# Supplementary material for: Long-term stability of RNA nucleoside standards for accurate LC–MS quantification
Source: Nucleic Acids Res. 2026 Jun 8;54(11):gkag444. doi: 10.1093/nar/gkag444 (PMC13244150; doi:10.1093/nar/gkag444)
Supplement: gkag444_Supplemental_Files [file gkag444_supplemental_files.zip › Kerkhoff SI_2_SO_20260319.pdf]

Supporting Information (Part II)

Long-term stability of RNA nucleoside standards for accurate LC-MS quantification

Kira Kerkhoff[1,§], Hagen Wesseling[1,§], Yuyang Qi[1,§], Sofia Obersteiner[1], Kuangjie Liu[2], Maximilian Berg[1], Leona Rusling[1], Hendrik Zipse[2] and Stefanie Kaiser[1,*]

[§] these authors contributed equally

* please address your correspondence to stefanie.kaiser@pharmchem.uni-frankfurt.de

[1] Goethe University Frankfurt, Institute of Pharmaceutical Chemistry, Max-von-Laue-Str. 9, 60438 Frankfurt, Germany

[2] Department of Chemistry, LMU Munich, Butenandtstrasse 5-13, 81377 Munich, Germany

Table of Contents

S1 Computational Details ···· **3**

S1.1 Method Development for Conformational Search ···· 3

S2 Theoretical Study ···· **4**

S2.1 Calculation Method ···· 4

S2.1.1 Complete Basis Set ···· 4

S2.1.2 Reaction Energy ···· 5

S2.2 Conformer Analysis ···· 9

S3 Results and Discussion ···· **104**

S3.1 Four Reaction Categories ···· 104

S3.1.1 Deglycosylation ···· 105

S3.1.2 Deamination ···· 107

S3.1.3 Deacetylation ···· 109

S3.1.4 Desulfurization ···· 111

S3.2 Corresponding Computational Results to Experimental Findings ···· 113

S3.2.1 Degradation of 4-thiouridine ···· 113

S3.2.2 Degradation of 3-methylcytidine ···· 114

S3.2.3 Degradation of N^6-isoprenyladenosine ···· 115

S3.2.4 Degradation of N^4-acetylcytidine ···· 116

S4 Reference ···· **117**

S1 Computational Details

S1.1 Method Development for Conformational Search

Appropriate conformational sampling is important to identify the conformer with the lowest energy. Cytidine was selected as the model compound to explore the conformational space using different computational techniques. The initial conformer search employed GOAT (Global Optimization Automated Toolkit) module in ORCA 6.0.0[1] and CREST 2.12[2-3] (Conformer-Rotamer Ensemble Sampling Tool) to have a robust assessment of the conformational space covered by different approaches. GOAT was applied at the GFN2-xTB[4] level to map the initial conformers of cytidine, while CREST was used here with both GFN2-xTB and GFNFF[5], yielding two additional sets of conformers for cytidine. Before conducting the conformational search, the input structure was pre-optimzed at the same level serving as a reference for topology check. The default energy window 6 kcal/mol was applied as a sorting threshold. The xTB method from GOAT and CREST yielded similar conformer sets, while GFNFF from CREST produced a distinct set. To evaluate each method's coverage of the conformational space, two key structural parameters were analyzed, the O1'-C1'-N1-C2 and C1'-C2'-C3'-C4' dihedral angle. The former determines whether the carbonyl group of cytosine is in an anti or gauche orientation, while the latter determines whether the ribose ring's C3' position is endo (positive) or exo (negative dihedral angle). After confirming the validity of this method, we chose the conformational search with CREST at GFN2-xTB level.

S2 Theoretical Study

S2.1 Calculation Method

S2.1.1Complete Basis Set

Following the conformer sampling, all conformers were further optimized using Gaussian 09[6] at the (U)B3LYP[7]-D3[8]/def2-TZVPP[9] level of theory in the gas phase, followed by single-point calculation at the SMD(H_2O)[10]/(U)B3LYP-D3/def2-TZVPP level of theory in the aqueous phase. Single point energies have subsequently been calculated with the DLPNO-CCSD(T)[11-12] method as implemented in ORCA 6.0.0 in combination with the cc-pVTZ and cc-pVQZ basis sets[13-15], followed by extrapolation to the complete basis set (CBS) limit to DLPNO-CCSD(T)/CBS total energies.[13] Each of the DLPNO is separated into E_{HF} as the Hartree-Fock reference energy and E_C as the final correlation energy. For the cc-pVTZ and cc-pVQZ basis sets, the 3/4 extrapolation scheme with n = 3 for triple- and m = 4 for quadruple-zeta (eq. (2.1)), and the constants with α = 5.46 and β = 3.05 were used (eq. (2.2)).[11, 16] $E_{CBS,HF}$ was summed up with $E_{CBS,C}$ to get the correct extrapolated DLPNO energy E_{CBS} (eq. (2.3)).

$$\mathrm{E_{HF}^{CBS}} = \frac{\mathrm{E_{HF}^{\mathit{n}}} \cdot e^{-\alpha\sqrt{m}} - \mathrm{E_{HF}^{\mathit{m}}} \cdot e^{-\alpha\sqrt{n}}}{e^{-\alpha\sqrt{m}} - e^{-\alpha\sqrt{n}}} \tag{2.1}$$

$$\mathrm{E_{C}^{CBS}} = \frac{\mathrm{E_{C}^{\mathit{n}}} \cdot n^{\beta} - \mathrm{E_{C}^{\mathit{m}}} \cdot m^{\beta}}{n^{\beta} - m^{\beta}} \tag{2.2}$$

$$\mathrm{E_{CBS}} = \mathrm{E_{HF}^{CBS}} + \mathrm{E_{C}^{CBS}} \tag{2.3}$$

S2.1.2Reaction Energy

The Gibbs free energy of solvation in water ΔG_{Solv} was obtained as the difference of total energies without zero-point correction in the aqueous phase $E_{Tot,W}$ and in the gas phase E_{Tot} (eq. (2.4)). The Gibbs free energy at standard state in the aqueous phase $G_{298,W}$ was obtained as the sum of E_{Tot}, ΔG_{Solv}, thermal correction to Gibbs free energy δG, and standard state correction energy to 1 mol/L ΔG_{Corr} (+7.908 kJ/mol) (eq. (2.5)).

$$\Delta G_{Solv} = E_{Tot,W} - E_{Tot} \tag{2.4}$$

$$G_{298,W} = E_{Tot} + \Delta G_{Solv} + \delta G + \Delta G_{Corr} \tag{2.5}$$

The Gibbs free energy at standard state in the gas phase G_{CBS} at DLPNO-CCSD(T)/CBS level of theory was obtained as the sum of E_{CBS} and δG (eq. (2.6)), while the aqueous phase $G_{CBS,W}$ was obtained as the sum of G_{CBS}, ΔG_{Solv}, and ΔG_{Corr} (eq. (2.7)).

$$G_{CBS} = E_{CBS} + \delta G \tag{2.6}$$

$$G_{CBS,W} = G_{CBS} + \Delta G_{Solv} + \Delta G_{Corr} \tag{2.7}$$

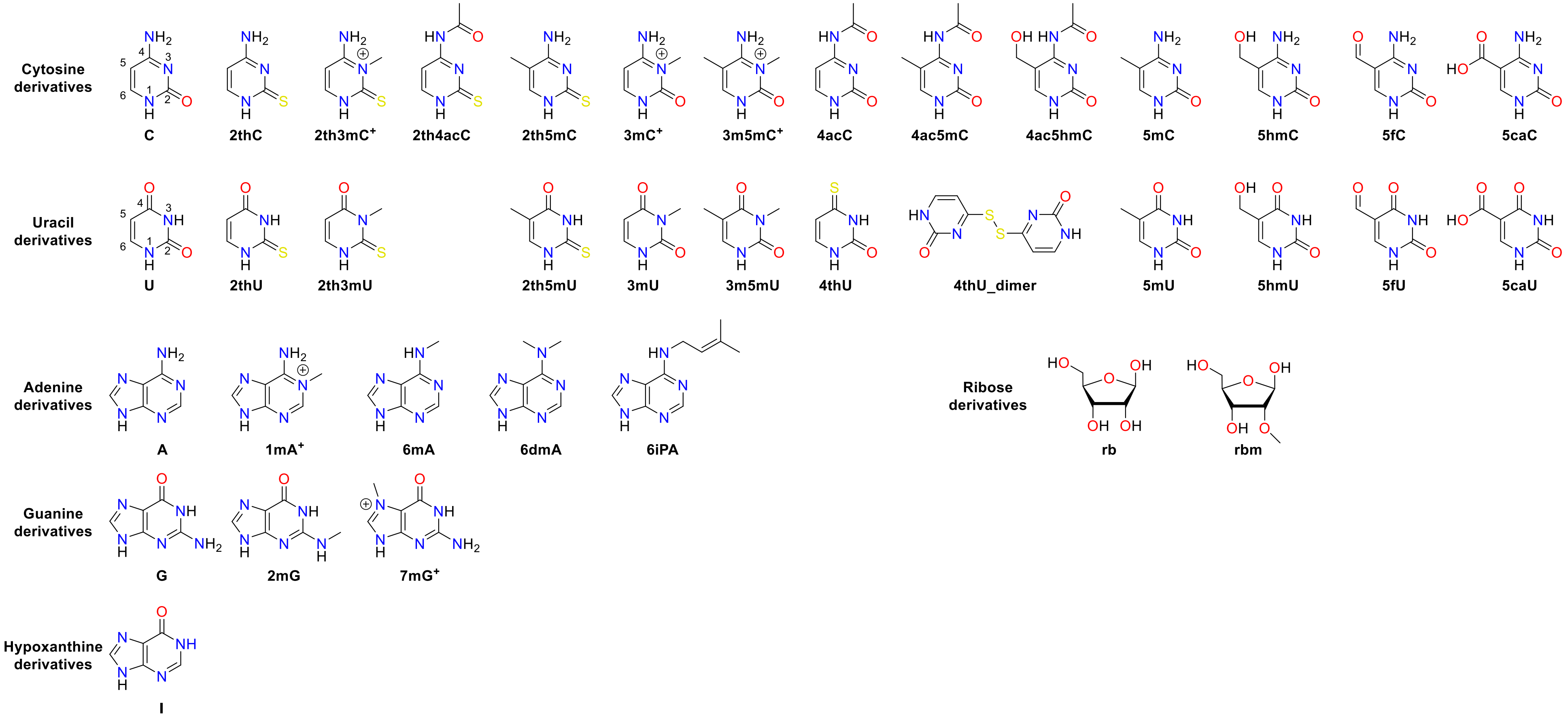

Figure S1. The 24 modified cytosine and uracil derivatives.

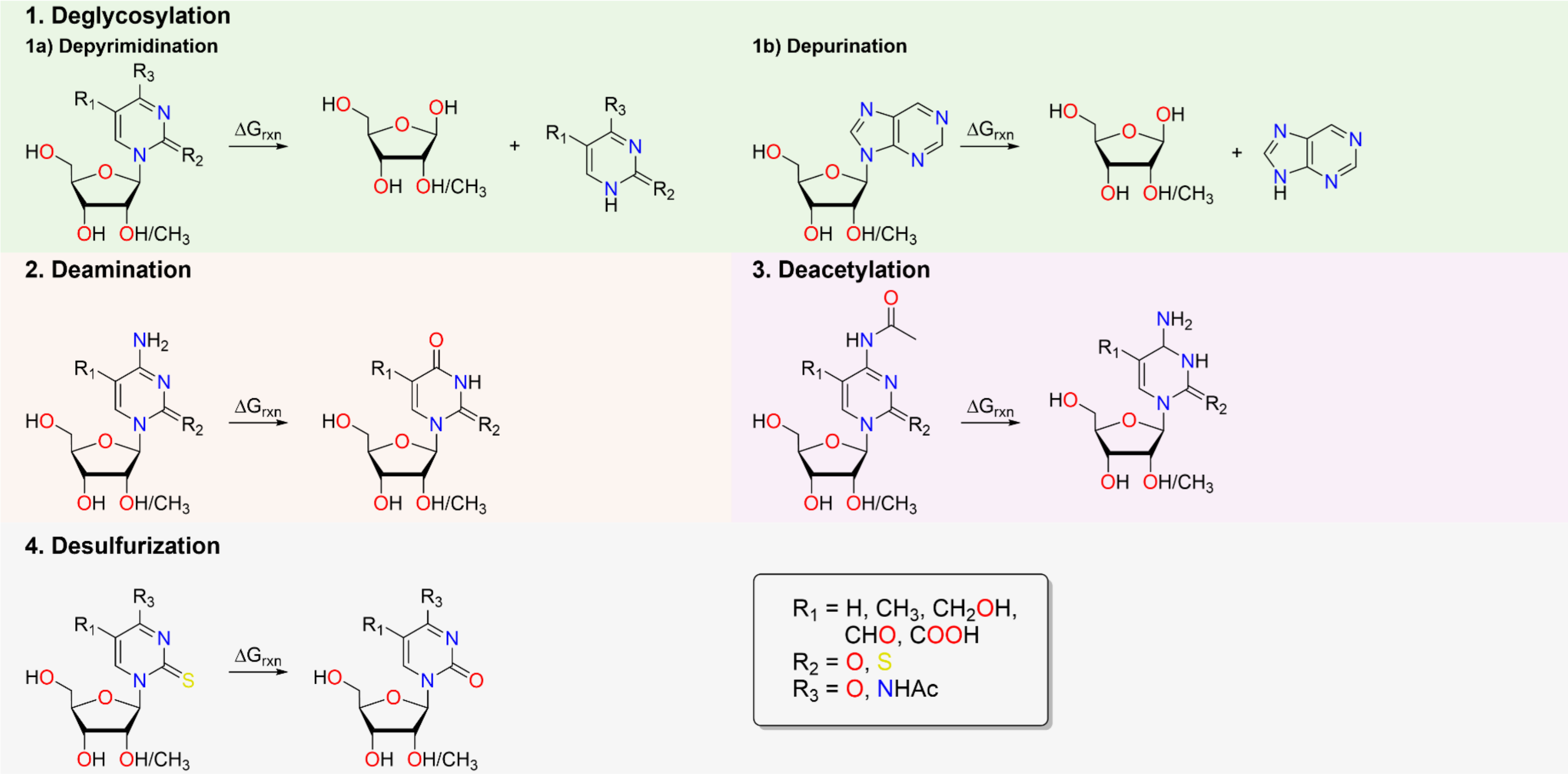

Figure S2. Overview of four potential decomposition reactions of modified cytidines and uridines: (1) Deglycosylation[17]; (2) Deamination[18]; (3) Deacetylation[19]; (4) Desulfurization[20].

The thermodynamic driving force for different reactions is reflected in the reaction free energies $\Delta E_{rxn,gas}$, $\Delta G_{rxn,gas}$, ΔG_{rxn} (eq. (2.8), eq. (2.9), eq. (2.10)).

$$\Delta E_{rxn,gas} = \sum E_{CBS}^{Product} - \sum E_{CBS}^{Reactant} \tag{2.8}$$

$$\Delta G_{rxn,gas} = \sum G_{CBS}^{Product} - \sum G_{CBS}^{Reactant} \tag{2.9}$$

$$\Delta G_{rxn} = \sum G_{CBS,W}^{Product} - \sum G_{CBS,W}^{Reactant} \tag{2.10}$$

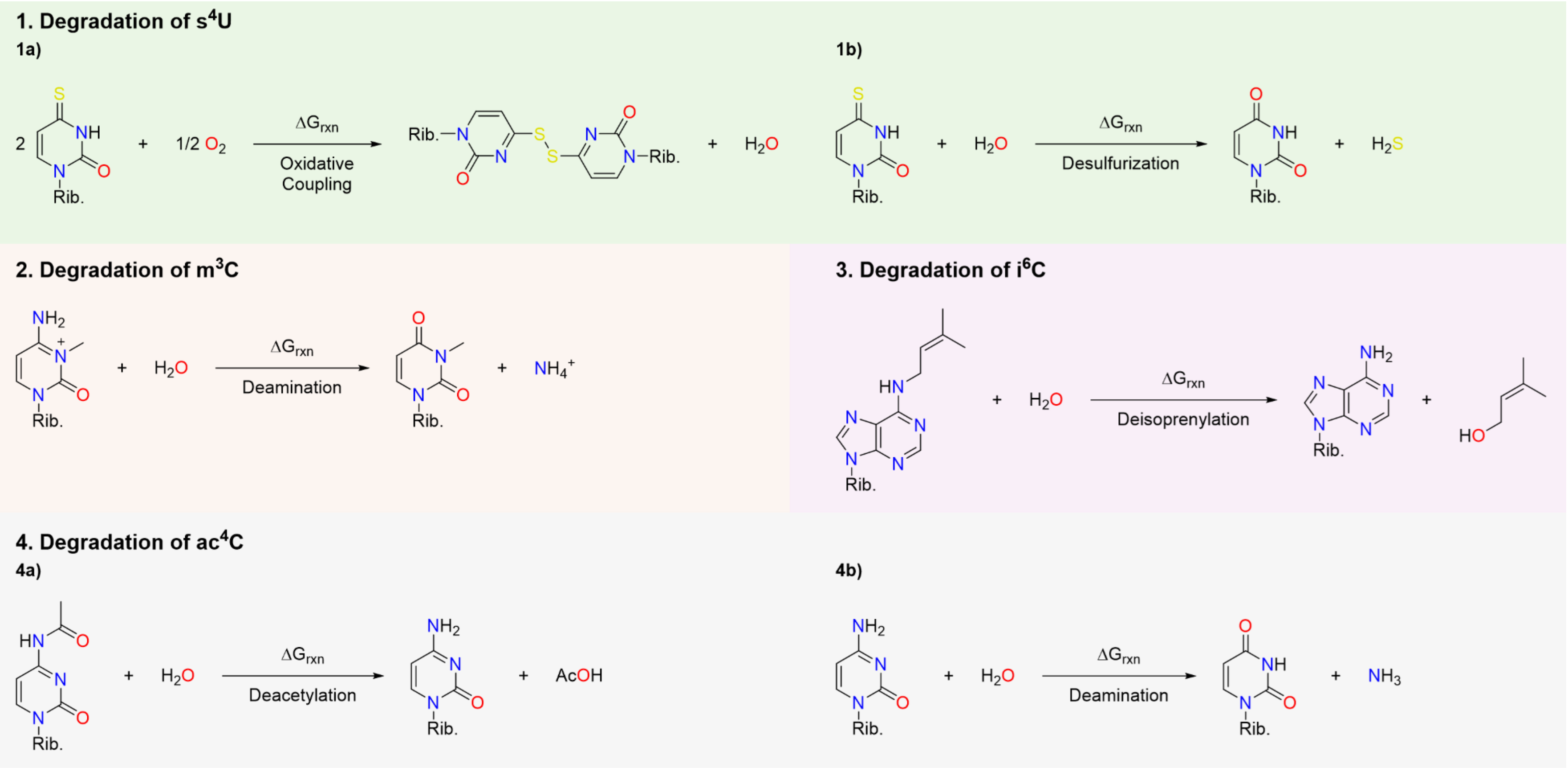

Figure S3. Corresponding four categories of reaction in **Figure 4**: (1) Degradation of 4-thiouridine; (2) Degradation of 3-methylcytidine; (3) Degradation of 6-isopentenyladenosine; (4) Degradation of *N*4-acetylcytidine.

The thermodynamic driving force for different reactions is reflected in the reaction free energies $\Delta E_{rxn,gas}$, $\Delta G_{rxn,gas}$, ΔG_{rxn} (eq. (2.8), eq. (2.9), eq. (2.10)).

$$\Delta E_{rxn,gas} = \sum E_{CBS}^{Product} - \sum E_{CBS}^{Reactant} \quad (2.11)$$

$$\Delta G_{rxn,gas} = \sum G_{CBS}^{Product} - \sum G_{CBS}^{Reactant} \quad (2.12)$$

$$\Delta G_{rxn} = \sum G_{CBS,W}^{Product} - \sum G_{CBS,W}^{Reactant} \quad (2.13)$$

S2.2 Conformer Analysis

Table S1. Gas-phase optimized basic molecules at the (U)B3LYP-D3/def2-TZVPP level of theory followed by aqueous phase single-point calculation. The columns display total energy without zero-point correction (E_{Tot}), Gibbs free energy (δG), total energy without zero-point correction ($E_{Tot,W}$), Gibbs free energy ($G_{298,W}$) in water (W), total single-point energy (E_{CBS}) calculated at DLPNO-CCSD(T)/CBS level of theory, and their corresponding free energy G_{CBS}. $G_{298,W}$ and $G_{CBS,W}$ have been corrected to the standard state of 1 mol/L by addition of +7.908 kJ/mol. ΔG_{Solv} represents the Gibbs free energy of solvation.

	(U)B3LYP-D3/def2-TZVPP			SMD(H_2O)/(U)B3LYP-D3/def2-TZVPP[a]				DLPNO-CCSD(T)/CBS		
**No.**	E_{Tot} (Hartree)	δH (Hartree)	δG (Hartree)	$E_{Tot,W}$ (Hartree)	ΔG_{Solv} (kJ/mol)	$H_{298,W}$ (Hartree)	$G_{298,W}$ (Hartree)	$E_{CBS,HF}$ (Hartree)	$E_{CBS,C}$ (Hartree)	$G_{CBS,W}$ (Hartree)
**H_2O**	-76.466681	0.025091	0.003672	-76.478486	-31.0	-76.453395	-76.471802	-76.066879	-0.309226	-76.381226
**NH_3**	-56.588660	0.038040	0.015162	-56.593896	-13.7	-56.555856	-56.575722	-56.224610	-0.280348	-56.492019
**NH_4^+**	-56.927233	0.053214	0.032115	-57.058092	-343.6	-57.004878	-57.022965	-56.569082	-0.273837	-56.938651
**H_2S**	-399.426820	0.018837	-0.004508	-399.428801	-5.2	-399.409964	-399.430297	-398.719353	-0.248411	-398.971242
**O_2**	-150.389771	0.007037	-0.016233	-150.390443	-1.8	-150.383406	-150.403664	-149.691643	-0.532628	-150.238165
**A**	-467.515091	0.120035	0.079489	-467.540992	-68.0	-467.420957	-467.458491	-464.721333	-2.041510	-466.706244
**1mA**	-506.811845	0.149407	0.106576	-506.852798	-107.5	-506.703391	-506.743210	-503.729300	-2.257015	-505.917680
**$1mA^+$**	-507.221283	0.163147	0.120183	-507.320812	-261.3	-507.157665	-507.197617	-504.149115	-2.241892	-506.367342
**6dmA**	-546.158371	0.179071	0.130722	-546.175493	-45.0	-546.041759	-546.172481	-542.797280	-2.461293	-545.141962
**I**	-487.391157	0.107798	0.068741	-487.423645	-85.3	-487.315847	-487.351892	-484.559106	-2.069309	-486.589150
**G**	-542.787393	0.125836	0.083717	-542.826154	-101.8	-542.700318	-542.739425	-539.635285	-2.309574	-541.896892
**7mG**	-582.116335	0.155488	0.108785	-582.150821	-90.5	-581.995333	-582.039024	-578.681077	-2.518395	-581.122161
**$7mG^+$**	-582.503931	0.168848	0.121988	-582.606659	-269.7	-582.437811	-582.481659	-579.073730	-2.507212	-581.558669
**C**	-395.107825	0.105973	0.067228	-395.138152	-79.6	-395.032179	-395.067912	-392.797146	-1.690307	-394.447539
**2thC**	-718.056461	0.104157	0.063605	-718.085841	-77.1	-717.981684	-718.019224	-715.431408	-1.640875	-717.035045
**2th5mC**	-757.392145	0.133521	0.089506	-757.421758	-77.8	-757.288237	-757.329240	-754.483796	-1.851279	-756.272171
**$3mC^+$**	-434.819005	0.149496	0.108303	-434.918911	-262.3	-434.769415	-434.807596	-432.230823	-1.890489	-434.109903
**5mC**	-434.442854	0.135362	0.093276	-434.473215	-79.7	-434.337853	-434.376927	-431.848606	-1.900906	-433.683584
**U**	-415.003094	0.094185	0.056709	-415.024647	-56.6	-414.930462	-414.964926	-412.658470	-1.715049	-414.335351
**2thU**	-737.951572	0.092349	0.053663	-737.971990	-53.6	-737.879641	-737.915315	-735.291119	-1.666870	-736.921732
**2th5mU**	-777.289446	0.121734	0.079397	-777.308811	-50.8	-777.187077	-777.226402	-774.346473	-1.876144	-776.159573
**3mU**	-454.328792	0.123500	0.081697	-454.347123	-48.1	-454.223623	-454.262414	-451.700717	-1.925575	-453.559914
**3m5mU**	-493.665904	0.152864	0.107839	-493.682978	-44.8	-493.530114	-493.572127	-490.755138	-2.135190	-492.796552
**5mU**	-454.340292	0.123509	0.082343	-454.360920	-54.2	-454.237411	-454.275565	-451.713007	-1.924502	-453.572781
**5ncmU**	-623.125333	0.154567	0.106331	-623.155382	-78.9	-623.000815	-623.046039	-619.578777	-2.602439	-622.101922

[a]: Single-point calculation in aqueous phase with SMD model.

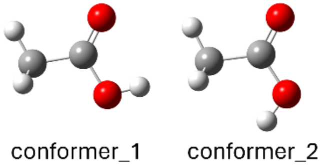

Figure S4. B3LYP-D3/def2-TZVPP optimized geometries of conformers for acetic acid (**AcOH**).

Table S2. Conformers of gas-phase optimized acetic acid (**AcOH**) at the B3LYP-D3/def2-TZVPP level of theory followed by aqueous phase single-point calculation. The columns display total energy without zero-point correction (E_{Tot}), thermal correction to enthalpy (δH), Gibbs free energy (δG), total energy without zero-point correction ($E_{Tot,W}$), Gibbs free energy ($G_{298,W}$) in water (W), total single-point energy (E_{CBS}) calculated at DLPNO-CCSD(T)/CBS level of theory, and their corresponding free energy $G_{CBS,W}$. $G_{298,W}$ and $G_{CBS,W}$ have been corrected to the standard state of 1 mol/L by addition of +7.908 kJ/mol. ΔG_{Solv} represents the Gibbs free energy of solvation. The data are arranged in the ascending numeric order of $E_{Tot,W}$. $\Delta G_{298,W}$ represents the respective energy difference to the lowest structure. Only conformers within the 24 kJ/mol (6 kcal/mol) energy window above the lowest in CREST are included in initial conformer sampling. Duplicates of the same structure are excluded. The overall optimum is marked bold.

AcOH No.	B3LYP-D3/def2-TZVPP			SMD(H_2O)/B3LYP-D3/def2-TZVPP[a]					DLPNO-CCSD(T)/CBS		
	E_{Tot} (Hartree)	δH (Hartree)	δG (Hartree)	$E_{Tot,W}$ (Hartree)	ΔG_{Solv} (kJ/mol)	$H_{298,W}$ (Hartree)	$G_{298,W}$ (Hartree)	$\Delta G_{298,W}$ (kJ/mol)	$E_{CBS,HF}$ (Hartree)	$E_{CBS,C}$ (Hartree)	$G_{CBS,W}$ (Hartree)
**conformer_1**	**-229.195033**	**0.067093**	**0.034344**	**-229.204206**	**-24.1**	**-229.137113**	**-229.166850**	**0**	**-227.924951**	**-0.941485**	**-228.838252**
conformer_2	-229.186670	0.066885	0.034463	-229.202476	-41.5	-229.135591	-229.165001	4.9	-227.915807	-0.942454	-228.836592

[a]: Single-point calculation in aqueous phase with SMD model.

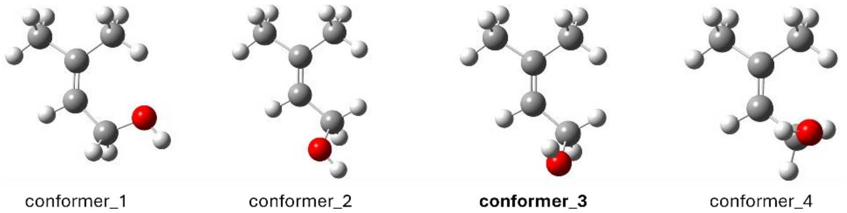

Figure S5. B3LYP-D3/def2-TZVPP optimized geometries of conformers for prenol (**iPOH**).

Table S3. Conformers of gas-phase optimized prenol (**iPOH**) at the B3LYP-D3/def2-TZVPP level of theory followed by aqueous phase single-point calculation. The columns display total energy without zero-point correction (E_{Tot}), thermal correction to enthalpy (δH), Gibbs free energy (δG), total energy without zero-point correction ($E_{Tot,W}$), Gibbs free energy ($G_{298,W}$) in water (W), total single-point energy (E_{CBS}) calculated at DLPNO-CCSD(T)/CBS level of theory, and their corresponding free energy $G_{CBS,W}$. $G_{298,W}$ and $G_{CBS,W}$ have been corrected to the standard state of 1 mol/L by addition of +7.908 kJ/mol. ΔG_{Solv} represents the Gibbs free energy of solvation. The data are arranged in the ascending numeric order of $E_{Tot,W}$. $\Delta G_{298,W}$ represents the respective energy difference to the lowest structure. Only conformers within the 24 kJ/mol (6 kcal/mol) energy window above the lowest in CREST are included in initial conformer sampling. Duplicates of the same structure are excluded. The overall optimum is marked bold.

	B3LYP-D3/def2-TZVPP			SMD(H_2O)/B3LYP-D3/def2-TZVPP[a]					DLPNO-CCSD(T)/CBS		
**iPOH No.**	**E_{Tot}** (Hartree)	**δH** (Hartree)	**δG** (Hartree)	**$E_{Tot,W}$** (Hartree)	**ΔG_{Solv}** (kJ/mol)	**$H_{298,W}$** (Hartree)	**$G_{298,W}$** (Hartree)	**$\Delta G_{298,W}$** (kJ/mol)	**$E_{CBS,HF}$** (Hartree)	**$E_{CBS,C}$** (Hartree)	**$G_{CBS,W}$** (Hartree)
**conformer_3**	**-271.880351**	**0.149829**	**0.109215**	**-271.886671**	**-16.6**	**-271.736842**	**-271.774444**	**0**	**-270.121870**	**-1.299424**	**-271.315388**
conformer_4	-271.879258	0.149690	0.108902	-271.884967	-15.0	-271.735277	-271.773053	3.7	-270.119787	-1.300259	-271.313842
conformer_2	-271.878153	0.149612	0.108563	-271.885463	-19.2	-271.735851	-271.773888	1.5	-270.120382	-1.299039	-271.315156
conformer_1	-271.875227	0.149587	0.107986	-271.880246	-13.2	-271.730659	-271.769248	13.6	-270.116702	-1.299543	-271.310266

[a]: Single-point calculation in aqueous phase with SMD model.

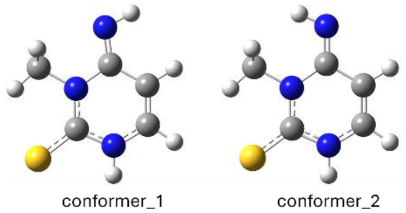

Figure S6. B3LYP-D3/def2-TZVPP optimized geometries of conformers for neutral 2-thio-3-methylcytosine (**2th3mC**).

Table S4. Conformers of gas-phase optimized neutral 2-thio-3-methylcytosine (**2th3mC**) at the B3LYP-D3/def2-TZVPP level of theory followed by aqueous phase single-point calculation. The columns display total energy without zero-point correction (E_{Tot}), thermal correction to enthalpy (δH), Gibbs free energy (δG), total energy without zero-point correction ($E_{Tot,W}$), Gibbs free energy ($G_{298,W}$) in water (W), total single-point energy (E_{CBS}) calculated at DLPNO-CCSD(T)/CBS level of theory, and their corresponding free energy $G_{CBS,W}$. $G_{298,W}$ and $G_{CBS,W}$ have been corrected to the standard state of 1 mol/L by addition of +7.908 kJ/mol. ΔG_{Solv} represents the Gibbs free energy of solvation. The data are arranged in the ascending numeric order of $E_{Tot,W}$. $\Delta G_{298,W}$ represents the respective energy difference to the lowest structure. Only conformers within the 24 kJ/mol (6 kcal/mol) energy window above the lowest in CREST are included in initial conformer sampling. Duplicates of the same structure are excluded. The overall optimum is marked bold.

2th3mC (neutral) No.	B3LYP-D3/def2-TZVPP E_{Tot} (Hartree)	δH (Hartree)	δG (Hartree)	SMD(H_2O)/B3LYP-D3/def2-TZVPP[a] $E_{Tot,W}$ (Hartree)	ΔG_{Solv} (kJ/mol)	$H_{298,W}$ (Hartree)	$G_{298,W}$ (Hartree)	$\Delta G_{298,W}$ (kJ/mol)	DLPNO-CCSD(T)/CBS $E_{CBS,HF}$ (Hartree)	$E_{CBS,C}$ (Hartree)	$G_{CBS,W}$ (Hartree)
**conformer_1**	**-757.376687**	**0.133987**	**0.091126**	**-757.3929946**	**-42.8**	**-757.259008**	**-757.298857**	**0**	**-754.465868**	**-1.856343**	**-756.244381**
conformer_2	-757.374004	0.133911	0.091407	-757.392241	-47.9	-757.258330	-757.297822	2.7	-754.462266	-1.857289	-756.243373

[a]: Single-point calculation in aqueous phase with SMD model.

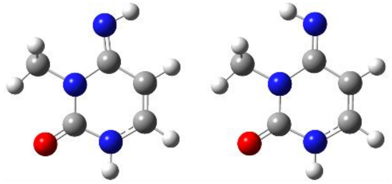

Figure S7. B3LYP-D3/def2-TZVPP optimized geometries of conformers for neutral 3-methylcytosine (**3mC**).

Table S5. Conformers of gas-phase optimized neutral 3-methylcytosine (**3mC**) at the B3LYP-D3/def2-TZVPP level of theory followed by aqueous phase single-point calculation. The columns display total energy without zero-point correction (E_{Tot}), thermal correction to enthalpy (δH), Gibbs free energy (δG), total energy without zero-point correction ($E_{Tot,W}$), Gibbs free energy ($G_{298,W}$) in water (W), total single-point energy (E_{CBS}) calculated at DLPNO-CCSD(T)/CBS level of theory, and their corresponding free energy $G_{CBS,W}$. $G_{298,W}$ and $G_{CBS,W}$ have been corrected to the standard state of 1 mol/L by addition of +7.908 kJ/mol. ΔG_{Solv} represents the Gibbs free energy of solvation. The data are arranged in the ascending numeric order of $E_{Tot,W}$. $\Delta G_{298,W}$ represents the respective energy difference to the lowest structure. Only conformers within the 24 kJ/mol (6 kcal/mol) energy window above the lowest in CREST are included in initial conformer sampling. Duplicates of the same structure are excluded. The overall optimum is marked bold.

	**B3LYP-D3/def2-TZVPP**			**SMD(H_2O)/B3LYP-D3/def2-TZVPP[a]**					**DLPNO-CCSD(T)/CBS**		
**3mC No.**	**E_{Tot}** (Hartree)	**δH** (Hartree)	**δG** (Hartree)	**$E_{Tot,W}$** (Hartree)	**ΔG_{Solv}** (kJ/mol)	**$H_{298,W}$** (Hartree)	**$G_{298,W}$** (Hartree)	**$\Delta G_{298,W}$** (kJ/mol)	**$E_{CBS,HF}$** (Hartree)	**$E_{CBS,C}$** (Hartree)	**$G_{CBS,W}$** (Hartree)
**conformer_1**	**-434.430332**	**0.135993**	**0.094736**	**-434.448131**	**-46.7**	**-434.312138**	**-434.350383**	**0**	**-431.836464**	**-1.902566**	**-433.659082**
conformer_2	-434.427760	0.135855	0.094797	-434.447343	-51.4	-434.311488	-434.349534	2.2	-431.833151	-1.903354	-433.658279

[a]: Single-point calculation in aqueous phase with SMD model.

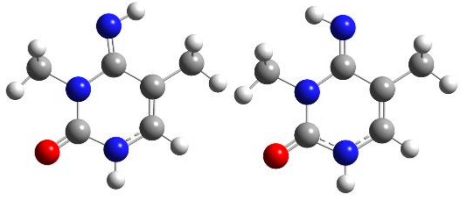

Figure S8. B3LYP-D3/def2-TZVPP optimized geometries of conformers for neutral 3,5-dimethylcytosine (**3m5mC**).

Table S6. Conformers of gas-phase optimized 3,5-dimethylcytosine (**3m5mC**) at the B3LYP-D3/def2-TZVPP level of theory followed by aqueous phase single-point calculation. The columns display total energy without zero-point correction (E_{Tot}), thermal correction to enthalpy (δH), Gibbs free energy (δG), total energy without zero-point correction ($E_{Tot,W}$), Gibbs free energy ($G_{298,W}$) in water (W), total single-point energy (E_{CBS}) calculated at DLPNO-CCSD(T)/CBS level of theory, and their corresponding free energy $G_{CBS,W}$. $G_{298,W}$ and $G_{CBS,W}$ have been corrected to the standard state of 1 mol/L by addition of +7.908 kJ/mol. ΔG_{Solv} represents the Gibbs free energy of solvation. The data are arranged in the ascending numeric order of $E_{Tot,W}$. $\Delta G_{298,W}$ represents the respective energy difference to the lowest structure. Only conformers within the 24 kJ/mol (6 kcal/mol) energy window above the lowest in CREST are included in initial conformer sampling. Duplicates of the same structure are excluded. The overall optimum is marked bold.

	**B3LYP-D3/def2-TZVPP**			**SMD(H_2O)/B3LYP-D3/def2-TZVPP[a]**					**DLPNO-CCSD(T)/CBS**		
**3m5mC No.**	**E_{Tot}** (Hartree)	**δH** (Hartree)	**δG** (Hartree)	**$E_{Tot,W}$** (Hartree)	**ΔG_{Solv}** (kJ/mol)	**$H_{298,W}$** (Hartree)	**$G_{298,W}$** (Hartree)	**$\Delta G_{298,W}$** (kJ/mol)	**$E_{CBS,HF}$** (Hartree)	**$E_{CBS,C}$** (Hartree)	**$G_{CBS,W}$** (Hartree)
**conformer_1**	**-473.766469**	**0.165386**	**0.120951**	**-473.783531**	**-44.8**	**-473.618145**	**-473.659568**	**0**	**-470.889491**	**-2.112950**	**-472.895541**
conformer_2	-473.764582	0.165271	0.120880	-473.782060	-45.9	-473.616789	-473.658168	3.7	-470.887268	-2.113253	-472.894107

[a]: Single-point calculation in aqueous phase with SMD model.

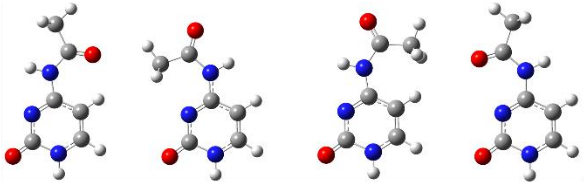

Figure S9. B3LYP-D3/def2-TZVPP optimized geometries of conformers for 4-acetylcytosine (**4acC**).

Table S7. Conformers of gas-phase optimized 4-acetylcytosine (**4acC**) at the B3LYP-D3/def2-TZVPP level of theory followed by aqueous phase single-point calculation. The columns display total energy without zero-point correction (E_{Tot}), thermal correction to enthalpy (δH), Gibbs free energy (δG), total energy without zero-point correction ($E_{Tot,W}$), Gibbs free energy ($G_{298,W}$) in water (W), total single-point energy (E_{CBS}) calculated at DLPNO-CCSD(T)/CBS level of theory, and their corresponding free energy $G_{CBS,W}$. $G_{298,W}$ and $G_{CBS,W}$ have been corrected to the standard state of 1 mol/L by addition of +7.908 kJ/mol. ΔG_{Solv} represents the Gibbs free energy of solvation. The data are arranged in the ascending numeric order of $E_{Tot,W}$. $\Delta G_{298,W}$ represents the respective energy difference to the lowest structure. Only conformers within the 24 kJ/mol (6 kcal/mol) energy window above the lowest in CREST are included in initial conformer sampling. Duplicates of the same structure are excluded. The overall optimum is marked bold.

	**B3LYP-D3/def2-TZVPP**			**SMD(H_2O)/B3LYP-D3/def2-TZVPP[a]**					**DLPNO-CCSD(T)/CBS**		
**4acC No.**	**E_{Tot}** (Hartree)	**δH** (Hartree)	**δG** (Hartree)	**$E_{Tot,W}$** (Hartree)	**ΔG_{Solv}** (kJ/mol)	**$H_{298,W}$** (Hartree)	**$G_{298,W}$** (Hartree)	**$\Delta G_{298,W}$** (kJ/mol)	**$E_{CBS,HF}$** (Hartree)	**$E_{CBS,C}$** (Hartree)	**$G_{CBS,W}$** (Hartree)
**conformer_1**	**-547.831137**	**0.147337**	**0.099975**	**-547.860219**	**-76.4**	**-547.712882**	**-547.757232**	**0**	**-544.644668**	**-2.330744**	**-546.901507**
conformer_2	-547.827390	0.147197	0.100795	-547.855859	-74.7	-547.708662	-547.752052	13.6	-544.640735	-2.330880	-546.896277
conformer_3	-547.822974	0.147389	0.099928	-547.855310	-84.9	-547.707921	-547.752370	12.8	-544.634064	-2.332871	-546.896331
conformer_4	-547.815264	0.146738	0.097880	-547.855236	-104.9	-547.708498	-547.754344	7.6	-544.628938	-2.331160	-546.899178

[a]: Single-point calculation in aqueous phase with SMD model.

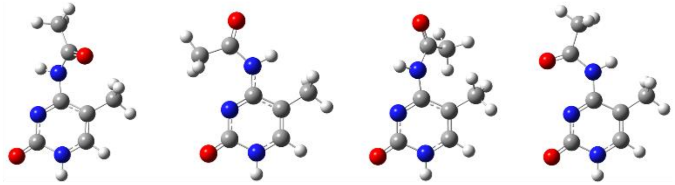

conformer_1 conformer_2 conformer_3 conformer_4

Figure S10. B3LYP-D3/def2-TZVPP optimized geometries of conformers for 4-acetylcytosine (**4ac5mC**).

Table S8. Conformers of gas-phase optimized 4-acetylcytosine (**4ac5mC**) at the B3LYP-D3/def2-TZVPP level of theory followed by aqueous phase single-point calculation. The columns display total energy without zero-point correction (E_{Tot}), thermal correction to enthalpy (δH), Gibbs free energy (δG), total energy without zero-point correction ($E_{Tot,W}$), Gibbs free energy ($G_{298,W}$) in water (W), total single-point energy (E_{CBS}) calculated at DLPNO-CCSD(T)/CBS level of theory, and their corresponding free energy $G_{CBS,W}$. $G_{298,W}$ and $G_{CBS,W}$ have been corrected to the standard state of 1 mol/L by addition of +7.908 kJ/mol. ΔG_{Solv} represents the Gibbs free energy of solvation. The data are arranged in the ascending numeric order of $E_{Tot,W}$. $\Delta G_{298,W}$ represents the respective energy difference to the lowest structure. Only conformers within the 24 kJ/mol (6 kcal/mol) energy window above the lowest in CREST are included in initial conformer sampling. Duplicates of the same structure are excluded. The overall optimum is marked bold.

	**B3LYP-D3/def2-TZVPP**			**SMD(H_2O)/B3LYP-D3/def2-TZVPP[a]**					**DLPNO-CCSD(T)/CBS**		
**4ac5mC No.**	**E_{Tot}** (Hartree)	**δH** (Hartree)	**δG** (Hartree)	**$E_{Tot,W}$** (Hartree)	**ΔG_{Solv}** (kJ/mol)	**$H_{298,W}$** (Hartree)	**$G_{298,W}$** (Hartree)	**$\Delta G_{298,W}$** (kJ/mol)	**$E_{CBS,HF}$** (Hartree)	**$E_{CBS,C}$** (Hartree)	**$G_{CBS,W}$** (Hartree)
conformer_2	-587.162198	0.176592	0.126910	-587.190234	-73.6	-587.013642	-587.060312	5.3	-583.691616	-2.542006	-586.131736
**conformer_4**	**-587.149641**	**0.176070**	**0.123965**	**-587.189295**	**-104.1**	**-587.013225**	**-587.062318**	**0**	**-583.679316**	**-2.542341**	**-586.134333**
conformer_1	-587.157924	0.176490	0.126200	-587.187525	-77.7	-587.011035	-587.058313	10.5	-583.685786	-2.543686	-586.129860
conformer_3	-587.150942	0.176474	0.126892	-587.183673	-85.9	-587.007199	-587.053769	22.4	-583.675770	-2.546055	-586.124653

[a]: Single-point calculation in aqueous phase with SMD model.

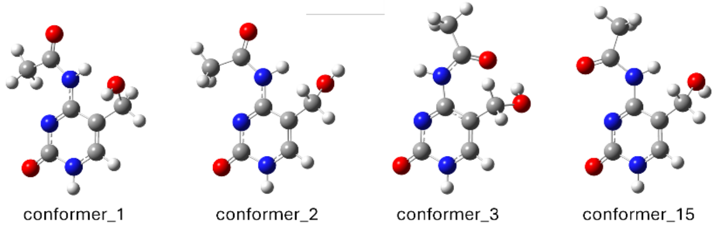

Figure S11. B3LYP-D3/def2-TZVPP optimized geometries of conformers for 4-acetylcytosine (**4ac5hmC**).

Table S9. Conformers of gas-phase optimized 4-acetylcytosine (**4ac5hmC**) at the B3LYP-D3/def2-TZVPP level of theory followed by aqueous phase single-point calculation. The columns display total energy without zero-point correction (E_{Tot}), thermal correction to enthalpy (δH), Gibbs free energy (δG), total energy without zero-point correction ($E_{Tot,W}$), Gibbs free energy ($G_{298,W}$) in water (W), total single-point energy (E_{CBS}) calculated at DLPNO-CCSD(T)/CBS level of theory, and their corresponding free energy $G_{CBS,W}$. $G_{298,W}$ and $G_{CBS,W}$ have been corrected to the standard state of 1 mol/L by addition of +7.908 kJ/mol. ΔG_{Solv} represents the Gibbs free energy of solvation. The data are arranged in the ascending numeric order of $E_{Tot,W}$. $\Delta G_{298,W}$ represents the respective energy difference to the lowest structure. Only conformers within the 24 kJ/mol (6 kcal/mol) energy window above the lowest in CREST are included in initial conformer sampling. Duplicates of the same structure are excluded. The overall optimum is marked bold.

	**B3LYP-D3/def2-TZVPP**			**SMD(H_2O)/B3LYP-D3/def2-TZVPP[a]**					**DLPNO-CCSD(T)/CBS**		
**4ac5hmC No.**	**E_{Tot}** (Hartree)	**δH** (Hartree)	**δG** (Hartree)	**$E_{Tot,W}$** (Hartree)	**ΔG_{Solv}** (kJ/mol)	**$H_{298,W}$** (Hartree)	**$G_{298,W}$** (Hartree)	**$\Delta G_{298,W}$** (kJ/mol)	**$E_{CBS,HF}$** (Hartree)	**$E_{CBS,C}$** (Hartree)	**$G_{CBS,W}$** (Hartree)
conformer_1	-662.412066	0.182943	0.131049	-662.445463	-87.7	-662.262520	-662.311402	3.5	-658.581907	-2.813391	-661.294634
**conformer_15**	**-662.402703**	**0.182621**	**0.129526**	**-662.445270**	**-111.8**	**-662.262649**	**-662.312732**	**0**	**-658.572657**	**-2.813795**	**-661.296480**
conformer_3	-662.410839	0.183186	0.131095	-662.444975	-89.6	-662.261789	-662.310868	4.9	-658.579563	-2.814871	-661.294463
conformer_2	-662.411005	0.182831	0.130877	-662.444484	-87.9	-662.261653	-662.310595	5.6	-658.581367	-2.813052	-661.294009
conformer_16	-662.400768	0.182439	0.129083	-662.443816	-113.0	-662.261377	-662.311721	2.7	-658.571231	-2.813508	-661.295691
conformer_5	-662.406978	0.182763	0.129772	-662.442723	-93.8	-662.259960	-662.309939	7.3	-658.577271	-2.813000	-661.293231
conformer_7	-662.406430	0.182845	0.130390	-662.442361	-94.3	-662.259516	-662.308959	9.9	-658.575843	-2.814425	-661.292797
conformer_4	-662.407181	0.182847	0.130097	-662.442123	-91.7	-662.259276	-662.309014	9.8	-658.578076	-2.812661	-661.292569
conformer_10	-662.404823	0.182702	0.129992	-662.441740	-96.9	-662.259038	-662.308736	10.5	-658.574089	-2.814485	-661.292487
conformer_6	-662.407561	0.182672	0.129464	-662.441242	-88.4	-662.258570	-662.308766	10.4	-658.579614	-2.812061	-661.292879
conformer_12	-662.395451	0.182244	0.127123	-662.441115	-119.9	-662.258871	-662.310980	4.6	-658.565494	-2.813909	-661.294932
conformer_8	-662.406348	0.182778	0.129973	-662.440633	-90.0	-662.257855	-662.307648	13.3	-658.576593	-2.813945	-661.291838
conformer_14	-662.394662	0.182109	0.127403	-662.440034	-119.1	-662.257925	-662.309619	8.2	-658.566571	-2.812655	-661.294182

[a]: Single-point calculation in aqueous phase with SMD model.

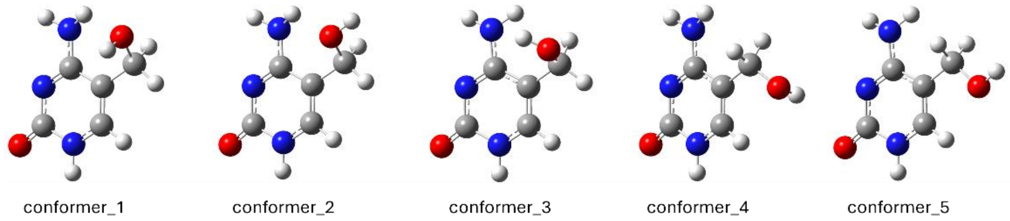

Figure S12. B3LYP-D3/def2-TZVPP optimized geometries of conformers for 5-hydroxymethylcytosine (**5hmC**).

Table S10. Conformers of gas-phase optimized 5-hydroxymethylcytosine (**5hmC**) at the B3LYP-D3/def2-TZVPP level of theory followed by aqueous phase single-point calculation. The columns display total energy without zero-point correction (E_{Tot}), thermal correction to enthalpy (δH), Gibbs free energy (δG), total energy without zero-point correction ($E_{Tot,W}$), Gibbs free energy ($G_{298,W}$) in water (W), total single-point energy (E_{CBS}) calculated at DLPNO-CCSD(T)/CBS level of theory, and their corresponding free energy $G_{CBS,W}$. $G_{298,W}$ and $G_{CBS,W}$ have been corrected to the standard state of 1 mol/L by addition of +7.908 kJ/mol. ΔG_{Solv} represents the Gibbs free energy of solvation. The data are arranged in the ascending numeric order of $E_{Tot,W}$. $\Delta G_{298,W}$ represents the respective energy difference to the lowest structure. Only conformers within the 24 kJ/mol (6 kcal/mol) energy window above the lowest in CREST are included in initial conformer sampling. Duplicates of the same structure are excluded. The overall optimum is marked bold.

	**B3LYP-D3/def2-TZVPP**			**SMD(H_2O)/B3LYP-D3/def2-TZVPP[a]**					**DLPNO-CCSD(T)/CBS**		
**5hmC No.**	**E_{Tot}** (Hartree)	**δH** (Hartree)	**δG** (Hartree)	**$E_{Tot,W}$** (Hartree)	**ΔG_{Solv}** (kJ/mol)	**$H_{298,W}$** (Hartree)	**$G_{298,W}$** (Hartree)	**$\Delta G_{298,W}$** (kJ/mol)	**$E_{CBS,HF}$** (Hartree)	**$E_{CBS,C}$** (Hartree)	**$G_{CBS,W}$** (Hartree)
**conformer_1**	**-509.694654**	**0.142043**	**0.098546**	**-509.728660**	**-89.3**	**-509.586617**	**-509.627102**	**0**	**-506.740818**	**-2.172386**	**-508.845652**
conformer_2	-509.692566	0.141943	0.098366	-509.727183	-90.9	-509.585240	-509.625805	3.4	-506.739355	-2.172144	-508.844739
conformer_3	-509.689268	0.141826	0.097581	-509.725824	-96.0	-509.583998	-509.625231	4.9	-506.735798	-2.172362	-508.844123
conformer_4	-509.688441	0.141776	0.097365	-509.725804	-98.1	-509.584028	-509.625427	4.4	-506.735622	-2.171677	-508.844285
conformer_5	-509.688129	0.141576	0.096764	-509.724763	-96.2	-509.583187	-509.624987	5.6	-506.736446	-2.171085	-508.844389

[a]: Single-point calculation in aqueous phase with SMD model.

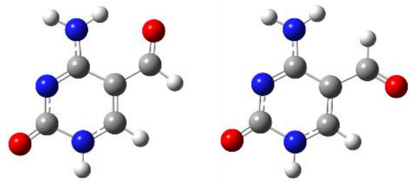

Figure S13. B3LYP-D3/def2-TZVPP optimized geometries of conformers for 5-formylcytosine (**5fC**).

Table S11. Conformers of gas-phase optimized 5-formylcytosine (**5fC**) at the B3LYP-D3/def2-TZVPP level of theory followed by aqueous phase single-point calculation. The columns display total energy without zero-point correction (E_{Tot}), thermal correction to enthalpy (δH), Gibbs free energy (δG), total energy without zero-point correction ($E_{Tot,W}$), Gibbs free energy ($G_{298,W}$) in water (W), total single-point energy (E_{CBS}) calculated at DLPNO-CCSD(T)/CBS level of theory, and their corresponding free energy $G_{CBS,W}$. $G_{298,W}$ and $G_{CBS,W}$ have been corrected to the standard state of 1 mol/L by addition of +7.908 kJ/mol. ΔG_{Solv} represents the Gibbs free energy of solvation. The data are arranged in the ascending numeric order of $E_{Tot,W}$. $\Delta G_{298,W}$ represents the respective energy difference to the lowest structure. Only conformers within the 24 kJ/mol (6 kcal/mol) energy window above the lowest in CREST are included in initial conformer sampling. Duplicates of the same structure are excluded. The overall optimum is marked bold.

	B3LYP-D3/def2-TZVPP			SMD(H_2O)/B3LYP-D3/def2-TZVPP[a]					DLPNO-CCSD(T)/CBS		
5fC No.	E_{Tot} (Hartree)	δH (Hartree)	δG (Hartree)	$E_{Tot,W}$ (Hartree)	ΔG_{Solv} (kJ/mol)	$H_{298,W}$ (Hartree)	$G_{298,W}$ (Hartree)	$\Delta G_{298,W}$ (kJ/mol)	$E_{CBS,HF}$ (Hartree)	$E_{CBS,C}$ (Hartree)	$G_{CBS,W}$ (Hartree)
**conformer_1**	**-508.491666**	**0.117684**	**0.075559**	**-508.521492**	**-78.3**	**-508.403808**	**-508.442921**	**0**	**-505.582485**	**-2.125234**	**-507.658974**
conformer_2	-508.481782	0.117328	0.073134	-508.514541	-86.0	-508.397213	-508.438395	11.9	-505.574374	-2.123434	-507.654422

[a]: Single-point calculation in aqueous phase with SMD model.

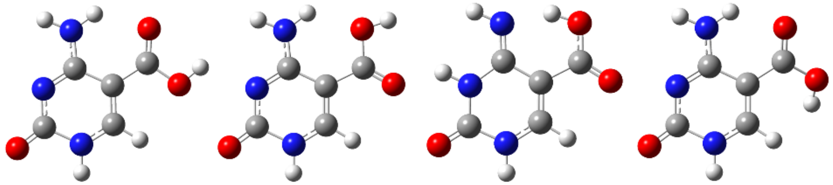

Figure S14. B3LYP-D3/def2-TZVPP optimized geometries of conformers for 5-carboxylcytosine (**5caC**).

Table S12. Conformers of gas-phase optimized 5-carboxylcytosine (**5caC**) at the B3LYP-D3/def2-TZVPP level of theory followed by aqueous phase single-point calculation. The columns display total energy without zero-point correction (E_{Tot}), thermal correction to enthalpy (δH), Gibbs free energy (δG), total energy without zero-point correction ($E_{Tot,W}$), Gibbs free energy ($G_{298,W}$) in water (W), total single-point energy (E_{CBS}) calculated at DLPNO-CCSD(T)/CBS level of theory, and their corresponding free energy $G_{CBS,W}$. $G_{298,W}$ and $G_{CBS,W}$ have been corrected to the standard state of 1 mol/L by addition of +7.908 kJ/mol. ΔG_{Solv} represents the Gibbs free energy of solvation. The data are arranged in the ascending numeric order of $E_{Tot,W}$. $\Delta G_{298,W}$ represents the respective energy difference to the lowest structure. Only conformers within the 24 kJ/mol (6 kcal/mol) energy window above the lowest in CREST are included in initial conformer sampling. Duplicates of the same structure are excluded. The overall optimum is marked bold.

	B3LYP-D3/def2-TZVPP			SMD(H_2O)/B3LYP-D3/def2-TZVPP[a]					DLPNO-CCSD(T)/CBS		
**5caC No.**	**E_{Tot}** (Hartree)	**δH** (Hartree)	**δG** (Hartree)	**$E_{Tot,W}$** (Hartree)	**ΔG_{Solv}** (kJ/mol)	**$H_{298,W}$** (Hartree)	**$G_{298,W}$** (Hartree)	**$\Delta G_{298,W}$** (kJ/mol)	**$E_{CBS,HF}$** (Hartree)	**$E_{CBS,C}$** (Hartree)	**$G_{CBS,W}$** (Hartree)
**conformer_1**	**-583.779532**	**0.124166**	**0.079604**	**-583.810296**	**-80.8**	**-583.686130**	**-583.727680**	**0**	**-580.514377**	**-2.394689**	**-582.857215**
conformer_2	-583.775473	0.124122	0.079178	-583.807575	-84.3	-583.683453	-583.725385	6.0	-580.510307	-2.394526	-582.854746
conformer_4	-583.765791	0.123745	0.078854	-583.804643	-102.0	-583.680898	-583.722777	12.9	-580.497909	-2.397918	-582.852814
conformer_3	-583.773088	0.124301	0.080409	-583.803086	-78.8	-583.678785	-583.719665	21.0	-580.505302	-2.399492	-582.851372

[a]: Single-point calculation in aqueous phase with SMD model.

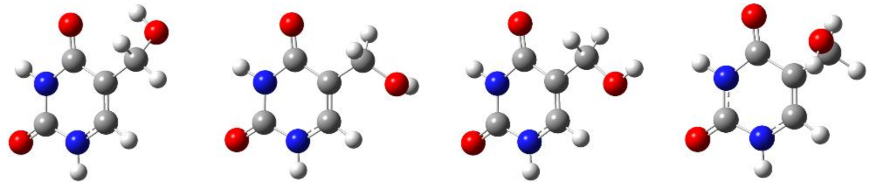

Figure S15. B3LYP-D3/def2-TZVPP optimized geometries of conformers for 5-hydroxymethyluracil (**5hmU**).

Table S13. Conformers of gas-phase optimized 5-hydroxymethyluracil (**5hmU**) at the B3LYP-D3/def2-TZVPP level of theory followed by aqueous phase single-point calculation. The columns display total energy without zero-point correction (E_{Tot}), thermal correction to enthalpy (δH), Gibbs free energy (δG), total energy without zero-point correction ($E_{Tot,W}$), Gibbs free energy ($G_{298,W}$) in water (W), total single-point energy (E_{CBS}) calculated at DLPNO-CCSD(T)/CBS level of theory, and their corresponding free energy $G_{CBS,W}$. $G_{298,W}$ and $G_{CBS,W}$ have been corrected to the standard state of 1 mol/L by addition of +7.908 kJ/mol. ΔG_{Solv} represents the Gibbs free energy of solvation. The data are arranged in the ascending numeric order of $E_{Tot,W}$. $\Delta G_{298,W}$ represents the respective energy difference to the lowest structure. Only conformers within the 24 kJ/mol (6 kcal/mol) energy window above the lowest in CREST are included in initial conformer sampling. Duplicates of the same structure are excluded. The overall optimum is marked bold.

	**B3LYP-D3/def2-TZVPP**			**SMD(H_2O)/B3LYP-D3/def2-TZVPP[a]**					**DLPNO-CCSD(T)/CBS**		
**5hmU No.**	**E_{Tot}** (Hartree)	**δH** (Hartree)	**δG** (Hartree)	**$E_{Tot,W}$** (Hartree)	**ΔG_{Solv}** (kJ/mol)	**$H_{298,W}$** (Hartree)	**$G_{298,W}$** (Hartree)	**$\Delta G_{298,W}$** (kJ/mol)	**$E_{CBS,HF}$** (Hartree)	**$E_{CBS,C}$** (Hartree)	**$G_{CBS,W}$** (Hartree)
**conformer_1**	**-529.590156**	**0.130046**	**0.086799**	**-529.616085**	**-68.1**	**-529.486039**	**-529.526274**	**0**	**-526.604259**	**-2.195477**	**-528.735853**
conformer_5	-529.584124	0.129789	0.085797	-529.615046	-81.2	-529.485257	-529.526237	0.1	-526.598368	-2.195413	-528.735893
conformer_2	-529.587412	0.129812	0.085955	-529.614210	-70.4	-529.484398	-529.525243	2.7	-526.601871	-2.194935	-528.734637
conformer_4	-529.588097	0.129726	0.085718	-529.613519	-66.7	-529.483793	-529.524789	3.9	-526.603498	-2.194382	-528.734572

[a]: Single-point calculation in aqueous phase with SMD model.

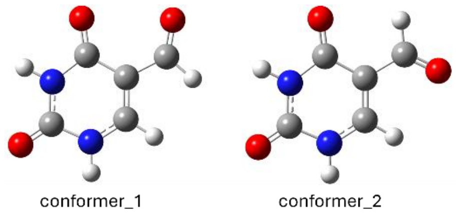

Figure S16. B3LYP-D3/def2-TZVPP optimized geometries of conformers for 5-formyluracil (**5fU**).

Table S14. Conformers of gas-phase optimized 5-formyluracil (**5fU**) at the B3LYP-D3/def2-TZVPP level of theory followed by aqueous phase single-point calculation. The columns display total energy without zero-point correction (E_{Tot}), thermal correction to enthalpy (δH), Gibbs free energy (δG), total energy without zero-point correction ($E_{Tot,W}$), Gibbs free energy ($G_{298,W}$) in water (W), total single-point energy (E_{CBS}) calculated at DLPNO-CCSD(T)/CBS level of theory, and their corresponding free energy $G_{CBS,W}$. $G_{298,W}$ and $G_{CBS,W}$ have been corrected to the standard state of 1 mol/L by addition of +7.908 kJ/mol. ΔG_{Solv} represents the Gibbs free energy of solvation. The data are arranged in the ascending numeric order of $E_{Tot,W}$. $\Delta G_{298,W}$ represents the respective energy difference to the lowest structure. Only conformers within the 24 kJ/mol (6 kcal/mol) energy window above the lowest in CREST are included in initial conformer sampling. Duplicates of the same structure are excluded. The overall optimum is marked bold.

	B3LYP-D3/def2-TZVPP			SMD(H_2O)/B3LYP-D3/def2-TZVPP[a]					DLPNO-CCSD(T)/CBS		
**5fU No.**	**E_{Tot}** (Hartree)	**δH** (Hartree)	**δG** (Hartree)	**$E_{Tot,W}$** (Hartree)	**ΔG_{Solv}** (kJ/mol)	**$H_{298,W}$** (Hartree)	**$G_{298,W}$** (Hartree)	**$\Delta G_{298,W}$** (kJ/mol)	**$E_{CBS,HF}$** (Hartree)	**$E_{CBS,C}$** (Hartree)	**$G_{CBS,W}$** (Hartree)
conformer 2	-528.377221	0.105589	0.063191	-528.401381	-63.4	-528.295792	-528.335178	0.8	-525.438085	-2.147268	-527.543310
**conformer_1**	**-528.369075**	**0.105223**	**0.062527**	**-528.401035**	**-83.9**	**-528.295812**	**-528.335496**	**0**	**-525.428418**	**-2.149230**	**-527.544069**

[a]: Single-point calculation in aqueous phase with SMD model.

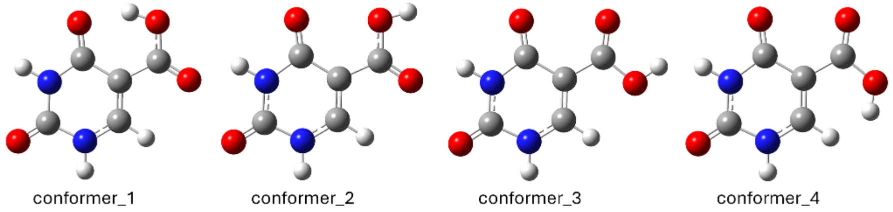

Figure S17. B3LYP-D3/def2-TZVPP optimized geometries of conformers for 5-carboxyluracil (**5caU**).

Table S15. Conformers of gas-phase optimized 5-carboxyluracil (**5caU**) at the B3LYP-D3/def2-TZVPP level of theory followed by aqueous phase single-point calculation. The columns display total energy without zero-point correction (E_{Tot}), thermal correction to enthalpy (δH), Gibbs free energy (δG), total energy without zero-point correction ($E_{Tot,W}$), Gibbs free energy ($G_{298,W}$) in water (W), total single-point energy (E_{CBS}) calculated at DLPNO-CCSD(T)/CBS level of theory, and their corresponding free energy $G_{CBS,W}$. $G_{298,W}$ and $G_{CBS,W}$ have been corrected to the standard state of 1 mol/L by addition of +7.908 kJ/mol. ΔG_{Solv} represents the Gibbs free energy of solvation. The data are arranged in the ascending numeric order of $E_{Tot,W}$. $\Delta G_{298,W}$ represents the respective energy difference to the lowest structure. Only conformers within the 24 kJ/mol (6 kcal/mol) energy window above the lowest in CREST are included in initial conformer sampling. Duplicates of the same structure are excluded. The overall optimum is marked bold.

	B3LYP-D3/def2-TZVPP			SMD(H_2O)/B3LYP-D3/def2-TZVPP[a]					DLPNO-CCSD(T)/CBS		
**5caU No.**	**E_{Tot}** (Hartree)	**δH** (Hartree)	**δG** (Hartree)	**$E_{Tot,W}$** (Hartree)	**ΔG_{Solv}** (kJ/mol)	**$H_{298,W}$** (Hartree)	**$G_{298,W}$** (Hartree)	**$\Delta G_{298,W}$** (kJ/mol)	**$E_{CBS,HF}$** (Hartree)	**$E_{CBS,C}$** (Hartree)	**$G_{CBS,W}$** (Hartree)
**conformer_1**	**-603.669696**	**0.112187**	**0.068539**	**-603.697177**	**-72.2**	**-603.584990**	**-603.625626**	**0**	**-600.369687**	**-2.421361**	**-602.746978**
conformer_2	-603.661029	0.112020	0.066968	-603.690814	-78.2	-603.578794	-603.620834	12.6	-600.363939	-2.418454	-602.742199
conformer_3	-603.658339	0.111849	0.066209	-603.690700	-85.0	-603.578851	-603.621479	10.9	-600.360465	-2.419575	-602.743180
conformer_4	-603.646065	0.111622	0.066374	-603.684591	-101.1	-603.572969	-603.615205	27.4	-600.345924	-2.422098	-602.737162

[a]: Single-point calculation in aqueous phase with SMD model.

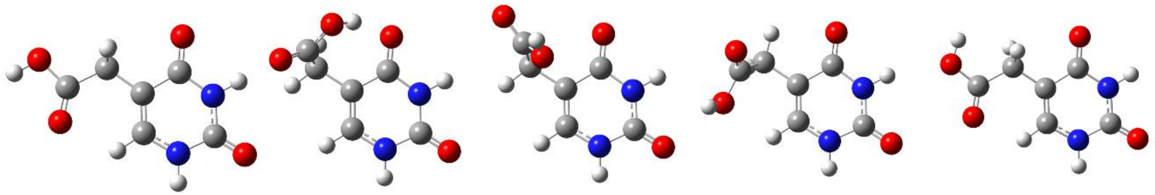

Figure S18. B3LYP-D3/def2-TZVPP optimized geometries of conformers for 5-carboxymethyluracil (**5cmU**).

Table S16. Conformers of gas-phase optimized 5-carboxymethyluracil (**5cmU**) at the B3LYP-D3/def2-TZVPP level of theory followed by aqueous phase single-point calculation. The columns display total energy without zero-point correction (E_{Tot}), thermal correction to enthalpy (δH), Gibbs free energy (δG), total energy without zero-point correction ($E_{Tot,W}$), Gibbs free energy ($G_{298,W}$) in water (W), total single-point energy (E_{CBS}) calculated at DLPNO-CCSD(T)/CBS level of theory, and their corresponding free energy $G_{CBS,W}$. $G_{298,W}$ and $G_{CBS,W}$ have been corrected to the standard state of 1 mol/L by addition of +7.908 kJ/mol. ΔG_{Solv} represents the Gibbs free energy of solvation. The data are arranged in the ascending numeric order of $E_{Tot,W}$. $\Delta G_{298,W}$ represents the respective energy difference to the lowest structure. Only conformers within the 24 kJ/mol (6 kcal/mol) energy window above the lowest in CREST are included in initial conformer sampling. Duplicates of the same structure are excluded. The overall optimum is marked bold.

	**B3LYP-D3/def2-TZVPP**			**SMD(H_2O)/B3LYP-D3/def2-TZVPP[a]**					**DLPNO-CCSD(T)/CBS**		
**5cmU No.**	**E_{Tot}** (Hartree)	**δH** (Hartree)	**δG** (Hartree)	**$E_{Tot,W}$** (Hartree)	**ΔG_{Solv}** (kJ/mol)	**$H_{298,W}$** (Hartree)	**$G_{298,W}$** (Hartree)	**$\Delta G_{298,W}$** (kJ/mol)	**$E_{CBS,HF}$** (Hartree)	**$E_{CBS,C}$** (Hartree)	**$G_{CBS,W}$** (Hartree)
conformer_2	-642.998192	0.142057	0.095002	-643.029047	-81.0	-642.886990	-642.931033	0.1	-639.415992	-2.630713	-641.979546
**conformer_3**	**-642.995056**	**0.141599**	**0.092742**	**-643.026842**	**-83.5**	**-642.885243**	**-642.931088**	**0.0**	**-639.416842**	**-2.627645**	**-641.980518**
conformer_1	-642.997261	0.141619	0.091287	-643.023508	-68.9	-642.881889	-642.929209	4.9	-639.419199	-2.627304	-641.978451
conformer_4	-642.993550	0.141864	0.091957	-643.022769	-76.7	-642.880905	-642.927800	8.6	-639.414254	-2.628366	-641.976870
conformer_5	-642.990022	0.141470	0.091590	-643.021644	-83.0	-642.880174	-642.927042	10.6	-639.411282	-2.628192	-641.976494

[a]: Single-point calculation in aqueous phase with SMD model.

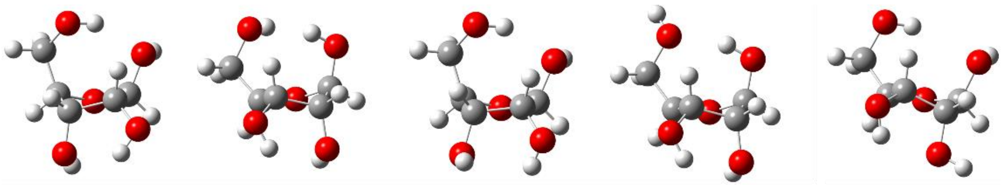

Figure S19. B3LYP-D3/def2-TZVPP optimized geometries of conformers for ribose (**rb**).

Table S17. Conformers of gas-phase optimized ribose (**rb**) at the B3LYP-D3/def2-TZVPP level of theory followed by aqueous phase single-point calculation. The columns display total energy without zero-point correction (E_{Tot}), thermal correction to enthalpy (δH), Gibbs free energy (δG), total energy without zero-point correction ($E_{Tot,W}$), Gibbs free energy ($G_{298,W}$) in water (W), total single-point energy (E_{CBS}) calculated at DLPNO-CCSD(T)/CBS level of theory, and their corresponding free energy $G_{CBS,W}$. $G_{298,W}$ and $G_{CBS,W}$ have been corrected to the standard state of 1 mol/L by addition of +7.908 kJ/mol. ΔG_{Solv} represents the Gibbs free energy of solvation. The data are arranged in the ascending numeric order of $E_{Tot,W}$. $\Delta G_{298,W}$ represents the respective energy difference to the lowest structure. Only conformers within the 24 kJ/mol (6 kcal/mol) energy window above the lowest in CREST are included in initial conformer sampling. Duplicates of the same structure are excluded. The overall optimum is marked bold.

	B3LYP-D3/def2-TZVPP			SMD(H_2O)/B3LYP-D3/def2-TZVPP[a]					DLPNO-CCSD(T)/CBS		
**rb No.**	**E_{Tot}** (Hartree)	**δH** (Hartree)	**δG** (Hartree)	**$E_{Tot,W}$** (Hartree)	**ΔG_{Solv}** (kJ/mol)	**$H_{298,W}$** (Hartree)	**$G_{298,W}$** (Hartree)	**$\Delta G_{298,W}$** (kJ/mol)	**$E_{CBS,HF}$** (Hartree)	**$E_{CBS,C}$** (Hartree)	**$G_{CBS,W}$** (Hartree)
conformer_6	-572.914169	0.175027	0.129285	-572.942252	-73.7	-572.767225	-572.809955	2.9	-569.719089	-2.389356	-572.004231
conformer_66	-572.915161	0.174926	0.128628	-572.942244	-71.1	-572.767318	-572.810604	1.2	-569.722553	-2.387387	-572.005384
**conformer_13**	**-572.913711**	**0.174756**	**0.127858**	**-572.941939**	**-74.1**	**-572.767183**	**-572.811069**	**0**	**-569.722387**	**-2.386200**	**-572.005945**
conformer_46	-572.913320	0.175143	0.129354	-572.941857	-74.9	-572.766714	-572.809491	4.1	-569.719682	-2.388424	-572.004277
conformer_7	-572.913779	0.175086	0.128953	-572.941314	-72.3	-572.766228	-572.809349	4.5	-569.720795	-2.387875	-572.004239
conformer_4	-572.914421	0.175282	0.129553	-572.941290	-70.5	-572.766008	-572.808725	6.2	-569.719054	-2.389879	-572.003236
conformer_26	-572.911705	0.174931	0.127248	-572.941013	-76.9	-572.766082	-572.810753	0.8	-569.721909	-2.385083	-572.006040
conformer_1	-572.914745	0.175313	0.130317	-572.940993	-68.9	-572.765680	-572.807664	8.9	-569.718074	-2.391333	-572.002326
conformer_3	-572.914494	0.175223	0.129700	-572.940917	-69.4	-572.765694	-572.808205	7.5	-569.718603	-2.390395	-572.002709
conformer_24	-572.913413	0.174921	0.129324	-572.940907	-72.2	-572.765986	-572.808571	6.6	-569.717582	-2.390331	-572.003072
conformer_11	-572.913026	0.174923	0.128617	-572.940813	-73.0	-572.765890	-572.809184	4.9	-569.720956	-2.387248	-572.004362
conformer_25	-572.909467	0.175115	0.129866	-572.940692	-82.0	-572.765577	-572.807814	8.5	-569.712574	-2.391117	-572.002038
conformer_5	-572.914557	0.175028	0.129666	-572.940628	-68.4	-572.765600	-572.807950	8.2	-569.719445	-2.389992	-572.002831
conformer_78	-572.912321	0.174825	0.128746	-572.940490	-74.0	-572.765665	-572.808732	6.1	-569.719661	-2.388030	-572.004102
conformer_62	-572.909504	0.174902	0.127432	-572.940339	-81.0	-572.765437	-572.809895	3.1	-569.718795	-2.385801	-572.004987
conformer_44	-572.912235	0.174700	0.127409	-572.940301	-73.7	-572.765601	-572.809880	3.1	-569.723221	-2.384577	-572.005443
conformer_35	-572.909060	0.174753	0.127925	-572.940204	-81.8	-572.765451	-572.809267	4.7	-569.715867	-2.387730	-572.003805
conformer_12	-572.913218	0.174733	0.128323	-572.940033	-70.4	-572.765300	-572.808698	6.2	-569.721289	-2.387371	-572.004140
conformer_31	-572.909719	0.174781	0.128136	-572.939872	-79.2	-572.765091	-572.808724	6.2	-569.717891	-2.387031	-572.003927
conformer_22	-572.910769	0.175143	0.129796	-572.939830	-76.3	-572.764687	-572.807022	10.6	-569.715178	-2.390372	-572.001803
conformer_14	-572.914090	0.174995	0.128615	-572.939518	-66.8	-572.764523	-572.807891	8.3	-569.721023	-2.388164	-572.002989
conformer_50	-572.908797	0.174476	0.126821	-572.939449	-80.5	-572.764973	-572.809616	3.8	-569.720011	-2.384338	-572.005168
conformer_85	-572.909534	0.174731	0.128028	-572.939448	-78.5	-572.764717	-572.808408	7.0	-569.718357	-2.386629	-572.003859
conformer_23	-572.910686	0.175052	0.128971	-572.939391	-75.4	-572.764339	-572.807408	9.6	-569.717058	-2.388981	-572.002762
conformer_53	-572.911157	0.174600	0.127001	-572.939308	-73.9	-572.764708	-572.809295	4.7	-569.722102	-2.384782	-572.005021
conformer_63	-572.907046	0.174802	0.128159	-572.939308	-84.7	-572.764506	-572.808137	7.7	-569.712567	-2.389177	-572.002835
conformer_52	-572.911235	0.174704	0.127979	-572.939005	-72.9	-572.764301	-572.808014	8.0	-569.720535	-2.385919	-572.003233
conformer_70	-572.908511	0.174754	0.128168	-572.938916	-79.8	-572.764162	-572.807736	8.8	-569.715922	-2.387724	-572.002872
conformer_42	-572.909719	0.174558	0.126928	-572.938898	-76.6	-572.764340	-572.808958	5.5	-569.719999	-2.384867	-572.004105
conformer_83	-572.907449	0.174717	0.126151	-572.938874	-82.5	-572.764157	-572.809711	3.6	-569.717792	-2.384824	-572.004878

conformer_19	-572.909783	0.174892	0.128346	-572.938746	-76.0	-572.763854	-572.807388	9.7	-569.717291	-2.387566	-572.002463
conformer_20	-572.910234	0.174709	0.127316	-572.938746	-74.9	-572.764037	-572.808418	7.0	-569.720284	-2.385502	-572.003970
conformer_75	-572.904562	0.174508	0.126383	-572.938668	-89.5	-572.764160	-572.809273	4.7	-569.712836	-2.386144	-572.003692
conformer_47	-572.908863	0.174674	0.127142	-572.938628	-78.1	-572.763954	-572.808474	6.8	-569.718449	-2.386140	-572.004200
conformer_91	-572.905779	0.174644	0.126283	-572.938613	-86.2	-572.763969	-572.809318	4.6	-569.716250	-2.384433	-572.004221
conformer_39	-572.912880	0.174808	0.128096	-572.938551	-67.4	-572.763743	-572.807443	9.5	-569.723299	-2.385080	-572.002943
conformer_17	-572.908222	0.174864	0.126862	-572.938547	-79.6	-572.763683	-572.808673	6.3	-569.717442	-2.385887	-572.003780
conformer_86	-572.903503	0.174355	0.126037	-572.938503	-91.9	-572.764148	-572.809454	4.2	-569.711862	-2.386032	-572.003846
conformer_37	-572.911295	0.174867	0.128196	-572.938475	-71.4	-572.763608	-572.807267	10.0	-569.719153	-2.386954	-572.002079
conformer_90	-572.907223	0.174546	0.127208	-572.938465	-82.0	-572.763919	-572.808245	7.4	-569.717515	-2.385012	-572.003549
conformer_38	-572.910571	0.175014	0.128804	-572.938459	-73.2	-572.763445	-572.806643	11.6	-569.715599	-2.389289	-572.000960
conformer_93	-572.907853	0.174386	0.126499	-572.938260	-79.8	-572.763874	-572.808749	6.1	-569.717978	-2.385061	-572.003935
conformer_21	-572.911210	0.175068	0.129831	-572.938256	-71.0	-572.763188	-572.805413	14.9	-569.714899	-2.390671	-571.999773
conformer_71	-572.911410	0.174740	0.127758	-572.938177	-70.3	-572.763437	-572.807407	9.6	-569.721638	-2.384909	-572.002545
conformer_15	-572.908803	0.174955	0.128540	-572.937960	-76.6	-572.763005	-572.806408	12.2	-569.715634	-2.388479	-572.001718
conformer_68	-572.910368	0.174773	0.128050	-572.937947	-72.4	-572.763174	-572.806885	11.0	-569.719436	-2.385848	-572.001802
conformer_57	-572.910502	0.174654	0.127445	-572.937946	-72.1	-572.763292	-572.807489	9.4	-569.721853	-2.384482	-572.003323
conformer_41	-572.905443	0.174647	0.127354	-572.937882	-85.2	-572.763235	-572.807516	9.3	-569.711638	-2.388301	-572.002013
conformer_30	-572.907921	0.174928	0.128347	-572.937823	-78.5	-572.762895	-572.806464	12.1	-569.714636	-2.388506	-572.001685
conformer_100	-572.906032	0.174481	0.126663	-572.937788	-83.4	-572.763307	-572.808113	7.8	-569.716872	-2.384440	-572.003393
conformer_79	-572.908241	0.174764	0.128091	-572.937721	-77.4	-572.762957	-572.806618	11.7	-569.716614	-2.387128	-572.002119
conformer_72	-572.909765	0.174603	0.127800	-572.937653	-73.2	-572.763050	-572.806841	11.1	-569.720177	-2.385087	-572.002339
conformer_64	-572.913360	0.174785	0.127995	-572.937592	-63.6	-572.762807	-572.806585	11.8	-569.724659	-2.384647	-572.002531
conformer_95	-572.904329	0.174530	0.126308	-572.937507	-87.1	-572.762977	-572.808187	7.6	-569.712884	-2.386139	-572.002880
conformer_32	-572.908053	0.175088	0.128960	-572.937467	-77.2	-572.762379	-572.805495	14.6	-569.712254	-2.390051	-571.999747
conformer_45	-572.908572	0.174566	0.127089	-572.937364	-75.6	-572.762798	-572.807263	10.0	-569.720304	-2.383855	-572.002850
conformer_73	-572.908136	0.174564	0.127491	-572.937258	-76.5	-572.762694	-572.806755	11.3	-569.718901	-2.384525	-572.002044
conformer_48	-572.909858	0.174759	0.128350	-572.937242	-71.9	-572.762483	-572.805880	13.6	-569.716023	-2.388520	-572.000564
conformer_54	-572.906434	0.174906	0.128356	-572.937074	-80.4	-572.762168	-572.805706	14.1	-569.713037	-2.388674	-572.000983
conformer_80	-572.910623	0.174714	0.127720	-572.936988	-69.2	-572.762274	-572.806256	12.6	-569.720636	-2.385403	-572.001672
conformer_34	-572.908829	0.174921	0.128357	-572.936843	-73.5	-572.761922	-572.805474	14.7	-569.716397	-2.388043	-572.001085
conformer_74	-572.906245	0.174535	0.126109	-572.936700	-80.0	-572.762165	-572.807579	9.2	-569.715953	-2.385913	-572.003200
conformer_88	-572.906497	0.174811	0.128150	-572.936615	-79.1	-572.761804	-572.805453	14.7	-569.713875	-2.388333	-572.001163
conformer_97	-572.907706	0.174469	0.126694	-572.936190	-74.8	-572.761721	-572.806484	12.0	-569.720033	-2.383568	-572.002379
conformer_61	-572.907744	0.174873	0.128234	-572.936108	-74.5	-572.761235	-572.804862	16.3	-569.714050	-2.388972	-572.000140
conformer_55	-572.907734	0.174620	0.126803	-572.936044	-74.3	-572.761424	-572.806229	12.7	-569.718095	-2.385541	-572.002131

[a]: Single-point calculation in aqueous phase with SMD model.

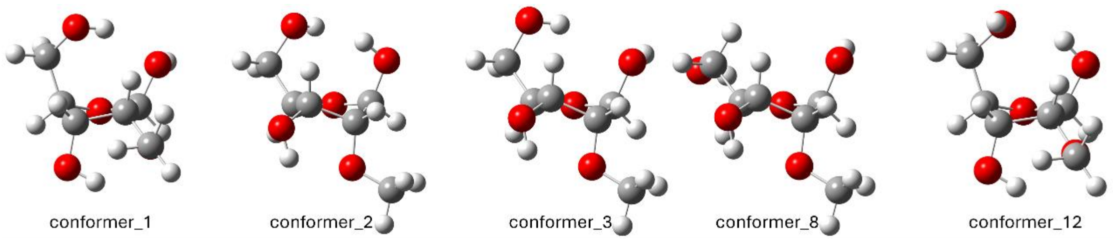

Figure S20. B3LYP-D3/def2-TZVPP optimized geometries of conformers for 2'-*O*-methylribose (**rbm**).

Table S18. Conformers of gas-phase optimized 2'-*O*-methylribose (**rbm**) at the B3LYP-D3/def2-TZVPP level of theory followed by aqueous phase single-point calculation. The columns display total energy without zero-point correction (E_{Tot}), thermal correction to enthalpy (δH), Gibbs free energy (δG), total energy without zero-point correction ($E_{Tot,W}$), Gibbs free energy ($G_{298,W}$) in water (W), total single-point energy (E_{CBS}) calculated at DLPNO-CCSD(T)/CBS level of theory, and their corresponding free energy $G_{CBS,W}$. $G_{298,W}$ and $G_{CBS,W}$ have been corrected to the standard state of 1 mol/L by addition of +7.908 kJ/mol. ΔG_{Solv} represents the Gibbs free energy of solvation. The data are arranged in the ascending numeric order of $E_{Tot,W}$. $\Delta G_{298,W}$ represents the respective energy difference to the lowest structure. Only conformers within the 24 kJ/mol (6 kcal/mol) energy window above the lowest in CREST are included in initial conformer sampling. Duplicates of the same structure are excluded. The overall optimum is marked bold.

	B3LYP-D3/def2-TZVPP			SMD(H2O)/B3LYP-D3/def2-TZVPP[a]					DLPNO-CCSD(T)/CBS		
**rbm No.**	**E_{Tot}** (Hartree)	**δH** (Hartree)	**δG** (Hartree)	**$E_{Tot,W}$** (Hartree)	**ΔG_{Solv}** (kJ/mol)	**$H_{298,W}$** (Hartree)	**$G_{298,W}$** (Hartree)	**$\Delta G_{298,W}$** (kJ/mol)	**$E_{CBS,HF}$** (Hartree)	**$E_{CBS,C}$** (Hartree)	**$G_{CBS,W}$** (Hartree)
conformer_3	-612.232256	0.204230	0.153791	-612.255416	-60.8	-612.051186	-612.098613	0.0	-608.759757	-2.591365	-611.217480
conformer_2	-612.232213	0.204604	0.155155	-612.254679	-59.0	-612.050075	-612.096512	5.5	-608.758142	-2.593059	-611.215500
conformer_1	-612.233033	0.204629	0.155613	-612.254433	-56.2	-612.049804	-612.095808	7.4	-608.756621	-2.595471	-611.214867
conformer_12	-612.227931	0.204349	0.155113	-612.253850	-68.0	-612.049501	-612.095725	7.6	-608.751023	-2.595572	-611.214390
conformer_8	-612.230756	0.204199	0.153604	-612.253699	-60.2	-612.049500	-612.097083	4.0	-608.760818	-2.589617	-611.216761
conformer_9	-612.230393	0.204259	0.153912	-612.253458	-60.6	-612.049199	-612.096534	5.5	-608.756635	-2.592836	-611.215611
conformer_5	-612.231589	0.204364	0.154772	-612.253353	-57.1	-612.048989	-612.095569	8.0	-608.758506	-2.592546	-611.215031
conformer_4	-612.232657	0.204487	0.155306	-612.253325	-54.3	-612.048838	-612.095007	9.5	-608.758408	-2.593578	-611.214336
conformer_27	-612.227279	0.203977	0.152838	-612.252959	-67.4	-612.048982	-612.097109	3.9	-608.757522	-2.589360	-611.216713
conformer_14	-612.227847	0.204226	0.153589	-612.252585	-64.9	-612.048359	-612.095984	6.9	-608.754953	-2.592696	-611.215786
conformer_18	-612.228175	0.204019	0.152720	-612.252401	-63.6	-612.048382	-612.096669	5.1	-608.757525	-2.589929	-611.215948
conformer_57	-612.226880	0.203918	0.152747	-612.252320	-66.8	-612.048402	-612.096561	5.4	-608.757641	-2.589094	-611.216416
conformer_36	-612.225548	0.203985	0.153241	-612.252032	-69.5	-612.048047	-612.095779	7.4	-608.754094	-2.590868	-611.215193
conformer_50	-612.226631	0.204418	0.155536	-612.251919	-66.4	-612.047501	-612.093371	13.8	-608.752037	-2.593606	-611.212383
conformer_24	-612.228306	0.204166	0.153669	-612.251584	-61.1	-612.047418	-612.094903	9.7	-608.756656	-2.590851	-611.214104
conformer_6	-612.229900	0.204202	0.153756	-612.251561	-56.9	-612.047359	-612.094793	10.0	-608.759222	-2.589946	-611.214062
conformer_25	-612.228226	0.204165	0.153223	-612.251532	-61.2	-612.047367	-612.095297	8.7	-608.757419	-2.590670	-611.215160
conformer_52	-612.223961	0.203840	0.152721	-612.251427	-72.1	-612.047587	-612.095694	7.7	-608.753036	-2.590245	-611.215015
conformer_44	-612.223961	0.203840	0.152721	-612.251427	-72.1	-612.047587	-612.095694	7.7	-608.753036	-2.590245	-611.215015
conformer_55	-612.227363	0.203962	0.152523	-612.250751	-61.4	-612.046789	-612.095216	8.9	-608.755674	-2.590677	-611.214204
conformer_54	-612.227363	0.203962	0.152523	-612.250751	-61.4	-612.046789	-612.095216	8.9	-608.755674	-2.590677	-611.214204
conformer_59	-612.225555	0.204160	0.153810	-612.250731	-66.1	-612.046571	-612.093909	12.4	-608.752151	-2.592198	-611.212703
conformer_10	-612.226269	0.204237	0.153541	-612.250658	-64.0	-612.046421	-612.094105	11.8	-608.753225	-2.592648	-611.213709
conformer_17	-612.227291	0.204509	0.155347	-612.250579	-61.1	-612.046070	-612.092220	16.8	-608.748563	-2.596798	-611.210290
conformer_51	-612.227291	0.204509	0.155347	-612.250579	-61.1	-612.046070	-612.092220	16.8	-608.748563	-2.596798	-611.210290
conformer_19	-612.226254	0.204099	0.152425	-612.250351	-63.3	-612.046252	-612.094914	9.7	-608.756049	-2.589990	-611.214699
conformer_42	-612.226865	0.204049	0.153417	-612.250270	-61.4	-612.046221	-612.093841	12.5	-608.756146	-2.590146	-611.213268
conformer_43	-612.226865	0.204049	0.153417	-612.250270	-61.4	-612.046221	-612.093841	12.5	-608.756146	-2.590146	-611.213268
conformer_33	-612.227604	0.204051	0.152640	-612.249984	-58.8	-612.045933	-612.094332	11.2	-608.756046	-2.590599	-611.213372

conformer_30	-612.225877	0.204381	0.155359	-612.249920	-63.1	-612.045539	-612.091549	18.5	-608.746770	-2.597398	-611.209840
conformer_15	-612.227063	0.204765	0.155922	-612.249860	-59.9	-612.045095	-612.090926	20.2	-608.747935	-2.597354	-611.209151
conformer_38	-612.225113	0.203891	0.152792	-612.249694	-64.5	-612.045803	-612.093890	12.4	-608.754882	-2.589578	-611.213237
conformer_40	-612.225113	0.203891	0.152792	-612.249694	-64.5	-612.045803	-612.093890	12.4	-608.754882	-2.589578	-611.213237
conformer_35	-612.225476	0.204140	0.153090	-612.249573	-63.3	-612.045433	-612.093471	13.5	-608.753167	-2.592229	-611.213391
conformer_20	-612.227048	0.204472	0.155706	-612.249477	-58.9	-612.045005	-612.090759	20.6	-608.748791	-2.596911	-611.209413
conformer_46	-612.225493	0.204008	0.152310	-612.249198	-62.2	-612.045190	-612.093876	12.4	-608.755872	-2.589727	-611.213982
conformer_45	-612.225493	0.204008	0.152310	-612.249198	-62.2	-612.045190	-612.093876	12.4	-608.755872	-2.589727	-611.213982
conformer_32	-612.225593	0.204304	0.154434	-612.249053	-61.6	-612.044749	-612.091607	18.4	-608.750300	-2.594408	-611.210721
conformer_48	-612.221904	0.204212	0.153980	-612.248810	-70.6	-612.044598	-612.091818	17.8	-608.745309	-2.594978	-611.210201
conformer_61	-612.223188	0.204004	0.152523	-612.248738	-67.1	-612.044734	-612.093203	14.2	-608.750420	-2.592632	-611.213067
conformer_49	-612.226525	0.204201	0.154027	-612.248662	-58.1	-612.044461	-612.091623	18.4	-608.755912	-2.589893	-611.210903
conformer_23	-612.224245	0.204171	0.153107	-612.248226	-63.0	-612.044055	-612.092107	17.1	-608.750535	-2.593110	-611.211506
conformer_28	-612.224245	0.204171	0.153106	-612.248225	-63.0	-612.044054	-612.092107	17.1	-608.750535	-2.593110	-611.211507
conformer_47	-612.222221	0.204132	0.153929	-612.248029	-67.8	-612.043897	-612.091088	19.8	-608.747220	-2.594164	-611.210251
conformer_60	-612.226515	0.204113	0.153689	-612.247500	-55.1	-612.043387	-612.090799	20.5	-608.756934	-2.589396	-611.210614
conformer_37	-612.222963	0.204316	0.154460	-612.247476	-64.4	-612.043160	-612.090004	22.6	-608.747413	-2.594462	-611.208916
conformer_53	-612.223600	0.204237	0.153532	-612.247338	-62.3	-612.043101	-612.090794	20.5	-608.748309	-2.594081	-611.209584
conformer_29	-612.223600	0.204237	0.153532	-612.247338	-62.3	-612.043101	-612.090794	20.5	-608.748309	-2.594081	-611.209584
conformer_56	-612.218636	0.204106	0.153580	-612.247259	-75.1	-612.043153	-612.090667	20.9	-608.741829	-2.595133	-611.208993

[a]: Single-point calculation in aqueous phase with SMD model.

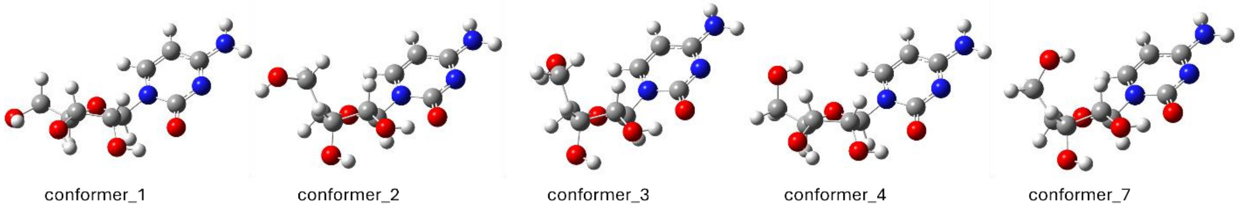

Figure S21. B3LYP-D3/def2-TZVPP optimized geometries of conformers for cytidine (**1rbC**).

Table S19. Conformers of gas-phase optimized cytidine (**1rbC**) at the B3LYP-D3/def2-TZVPP level of theory followed by aqueous phase single-point calculation. The columns display total energy without zero-point correction (E_{Tot}), thermal correction to enthalpy (δH), Gibbs free energy (δG), total energy without zero-point correction ($E_{Tot,W}$), Gibbs free energy ($G_{298,W}$) in water (W), total single-point energy (E_{CBS}) calculated at DLPNO-CCSD(T)/CBS level of theory, and their corresponding free energy $G_{CBS,W}$. $G_{298,W}$ and $G_{CBS,W}$ have been corrected to the standard state of 1 mol/L by addition of +7.908 kJ/mol. ΔG_{Solv} represents the Gibbs free energy of solvation. The data are arranged in the ascending numeric order of $E_{Tot,W}$. $\Delta G_{298,W}$ represents the respective energy difference to the lowest structure. Only conformers within the 24 kJ/mol (6 kcal/mol) energy window above the lowest in CREST are included in initial conformer sampling. Duplicates of the same structure are excluded. The overall optimum is marked bold.

	B3LYP-D3/def2-TZVPP			SMD(H_2O)/B3LYP-D3/def2-TZVPP[a]					DLPNO-CCSD(T)/CBS		
**1rbC No.**	**E_{Tot}** (Hartree)	**δH** (Hartree)	**δG** (Hartree)	**$E_{Tot,W}$** (Hartree)	**ΔG_{Solv}** (kJ/mol)	**$H_{298,W}$** (Hartree)	**$G_{298,W}$** (Hartree)	**$\Delta G_{298,W}$** (kJ/mol)	**$E_{CBS,HF}$** (Hartree)	**$E_{CBS,C}$** (Hartree)	**$G_{CBS,W}$** (Hartree)
**conformer_7**	**-891.565221**	**0.254695**	**0.194103**	**-891.604799**	**-103.9**	**-891.350104**	**-891.407684**	**0**	**-886.451845**	**-3.782145**	**-890.076454**
conformer_25	-891.563627	0.254916	0.195898	-891.605731	-110.5	-891.350815	-891.406821	2.3	-886.451786	-3.780856	-890.075836
conformer_15	-891.562518	0.254768	0.195391	-891.605471	-112.8	-891.350703	-891.407068	1.6	-886.450539	-3.780712	-890.075802
conformer_6	-891.564129	0.254640	0.194280	-891.603856	-104.3	-891.349216	-891.406564	2.9	-886.451808	-3.781278	-890.075520
conformer_19	-891.560777	0.254783	0.195480	-891.605474	-117.3	-891.350691	-891.406982	1.8	-886.446882	-3.782128	-890.075214
conformer_2	-891.563042	0.254626	0.193970	-891.602942	-104.8	-891.348316	-891.405960	4.5	-886.452157	-3.780070	-890.075145
conformer_10	-891.562510	0.254511	0.193903	-891.602865	-106.0	-891.348354	-891.405950	4.6	-886.451199	-3.780450	-890.075090
conformer_8	-891.565219	0.254648	0.194880	-891.604165	-102.3	-891.349517	-891.406273	3.7	-886.452608	-3.781391	-890.075052
conformer_3	-891.564468	0.254761	0.195507	-891.603840	-103.4	-891.349079	-891.405321	6.2	-886.452584	-3.781361	-890.074799
conformer_23	-891.563068	0.254663	0.195473	-891.603220	-105.4	-891.348557	-891.404735	7.7	-886.451868	-3.781256	-890.074791
conformer_27	-891.560288	0.254794	0.195200	-891.604398	-115.8	-891.349604	-891.406186	3.9	-886.448300	-3.780572	-890.074770
conformer_4	-891.562886	0.254741	0.193160	-891.600626	-99.1	-891.345885	-891.404454	8.5	-886.453586	-3.779437	-890.074591
conformer_22	-891.561537	0.254892	0.194936	-891.603173	-109.3	-891.348281	-891.405225	6.5	-886.452502	-3.778189	-890.074379
conformer_18	-891.562491	0.254772	0.195367	-891.603569	-107.9	-891.348797	-891.405190	6.5	-886.450566	-3.780976	-890.074241
conformer_21	-891.562174	0.254581	0.194119	-891.601787	-104.0	-891.347206	-891.404656	8.0	-886.451848	-3.779867	-890.074197
conformer_12	-891.565774	0.254731	0.195169	-891.602963	-97.6	-891.348232	-891.404782	7.6	-886.454194	-3.780945	-890.074147
conformer_29	-891.556819	0.254811	0.193660	-891.601252	-116.7	-891.346441	-891.404580	8.2	-886.447329	-3.778958	-890.074048
conformer_31	-891.557991	0.254722	0.193771	-891.600721	-112.2	-891.345999	-891.403938	9.8	-886.448614	-3.779305	-890.073866
conformer_16	-891.561358	0.254802	0.193480	-891.600210	-102.0	-891.345408	-891.403718	10.4	-886.453484	-3.778010	-890.073855
conformer_5	-891.562654	0.254796	0.193869	-891.600275	-98.8	-891.345479	-891.403394	11.3	-886.454527	-3.778501	-890.073768
conformer_14	-891.560458	0.254607	0.193590	-891.599728	-103.1	-891.345121	-891.403126	12.0	-886.452730	-3.778297	-890.073695
conformer_24	-891.560039	0.254593	0.193870	-891.599090	-102.5	-891.344497	-891.402208	14.4	-886.452784	-3.778078	-890.073031
conformer_37	-891.556241	0.254715	0.194681	-891.601380	-118.5	-891.346665	-891.403687	10.5	-886.443629	-3.781716	-890.072791
conformer_32	-891.555475	0.254829	0.194120	-891.600200	-117.4	-891.345371	-891.403068	12.1	-886.446102	-3.778623	-890.072318
conformer_39	-891.556779	0.254641	0.193434	-891.598162	-108.7	-891.343521	-891.401716	15.7	-886.448210	-3.778440	-890.071588
conformer_34	-891.555395	0.254752	0.194254	-891.598692	-113.7	-891.343940	-891.401426	16.4	-886.445807	-3.779141	-890.070978
conformer_1	-891.562372	0.254923	0.194558	-891.598283	-94.3	-891.343360	-891.400713	18.3	-886.453828	-3.778664	-890.070833
conformer_28	-891.557771	0.254722	0.193273	-891.596922	-102.8	-891.342200	-891.400637	18.5	-886.449667	-3.778257	-890.070789

[a]: Single-point calculation in aqueous phase with SMD model.

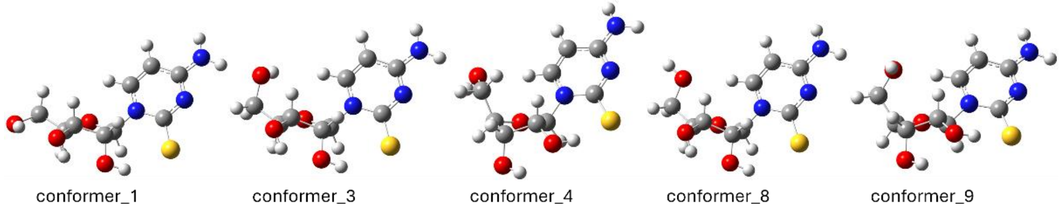

Figure S18. B3LYP-D3/def2-TZVPP optimized geometries of conformers for 2-thiocytidine (**1rb2thC**).

Table S20. Conformers of gas-phase optimized 2-thiocytidine (**1rb2thC**) at the B3LYP-D3/def2-TZVPP level of theory followed by aqueous phase single-point calculation. The columns display total energy without zero-point correction (E_{Tot}), thermal correction to enthalpy (δH), Gibbs free energy (δG), total energy without zero-point correction ($E_{Tot,W}$), Gibbs free energy ($G_{298,W}$) in water (W), total single-point energy (E_{CBS}) calculated at DLPNO-CCSD(T)/CBS level of theory, and their corresponding free energy $G_{CBS,W}$. $G_{298,W}$ and $G_{CBS,W}$ have been corrected to the standard state of 1 mol/L by addition of +7.908 kJ/mol. ΔG_{Solv} represents the Gibbs free energy of solvation. The data are arranged in the ascending numeric order of $E_{Tot,W}$. $\Delta G_{298,W}$ represents the respective energy difference to the lowest structure. Only conformers within the 24 kJ/mol (6 kcal/mol) energy window above the lowest in CREST are included in initial conformer sampling. Duplicates of the same structure are excluded. The overall optimum is marked bold.

1rb2thC No.	B3LYP-D3/def2-TZVPP E_{Tot} (Hartree)	δH (Hartree)	δG (Hartree)	SMD(H_2O)/B3LYP-D3/def2-TZVPP[a] $E_{Tot,W}$ (Hartree)	ΔG_{Solv} (kJ/mol)	$H_{298,W}$ (Hartree)	$G_{298,W}$ (Hartree)	$\Delta G_{298,W}$ (kJ/mol)	DLPNO-CCSD(T)/CBS $E_{CBS,HF}$ (Hartree)	$E_{CBS,C}$ (Hartree)	$G_{CBS,W}$ (Hartree)
conformer_9	-1214.510975	0.252236	0.191626	-1214.549342	-100.7	-1214.297106	-1214.354704	3.6	-1209.079501	-3.736723	-1212.659952
conformer_24	-1214.510682	0.252984	0.192007	-1214.548791	-100.1	-1214.295807	-1214.353772	6.0	-1209.084028	-3.733153	-1212.660269
conformer_14	-1214.508972	0.252245	0.191168	-1214.548783	-104.5	-1214.296538	-1214.354603	3.8	-1209.076947	-3.737707	-1212.660285
conformer_4	-1214.509693	0.252343	0.191912	-1214.548646	-102.3	-1214.296303	-1214.353722	6.1	-1209.078221	-3.737742	-1212.659992
**conformer_3**	**-1214.509607**	**0.252677**	**0.189552**	**-1214.548622**	**-102.4**	**-1214.295945**	**-1214.356058**	**0**	**-1209.082595**	**-3.733794**	**-1212.662840**
conformer_10	-1214.507229	0.252207	0.190580	-1214.548325	-107.9	-1214.296118	-1214.354733	3.5	-1209.077281	-3.735762	-1212.660549
conformer_13	-1214.511326	0.252249	0.191322	-1214.548210	-96.8	-1214.295961	-1214.353876	5.7	-1209.081053	-3.736160	-1212.659763
conformer_16	-1214.507703	0.252128	0.190570	-1214.547897	-105.5	-1214.295769	-1214.354315	4.6	-1209.073689	-3.739706	-1212.660007
conformer_18	-1214.508085	0.252333	0.192311	-1214.547876	-104.5	-1214.295543	-1214.352553	9.2	-1209.077065	-3.737860	-1212.659392
conformer_8	-1214.510845	0.252958	0.191910	-1214.547657	-96.6	-1214.294699	-1214.352735	8.7	-1209.085010	-3.732749	-1212.659649
conformer_28	-1214.509468	0.252698	0.191076	-1214.547409	-99.6	-1214.294711	-1214.353321	7.2	-1209.083437	-3.733350	-1212.660640
conformer_20	-1214.506164	0.252104	0.190374	-1214.547192	-107.7	-1214.295088	-1214.353806	5.9	-1209.076726	-3.735591	-1212.659959
conformer_12	-1214.507076	0.252496	0.190661	-1214.546732	-104.1	-1214.294236	-1214.353059	7.9	-1209.081543	-3.733135	-1212.660661
conformer_17	-1214.506570	0.252414	0.190536	-1214.546008	-103.5	-1214.293594	-1214.352460	9.4	-1209.081403	-3.733038	-1212.660330
conformer_30	-1214.496908	0.252422	0.189306	-1214.545832	-128.5	-1214.293410	-1214.353514	6.7	-1209.068711	-3.733176	-1212.658493
conformer_1	-1214.509534	0.252780	0.191549	-1214.545549	-94.6	-1214.292769	-1214.350988	13.3	-1209.082690	-3.734015	-1212.658158
conformer_27	-1214.500700	0.252775	0.191981	-1214.545105	-116.6	-1214.292330	-1214.350112	15.6	-1209.070416	-3.736136	-1212.655965
conformer_19	-1214.502268	0.252646	0.191667	-1214.543891	-109.3	-1214.291245	-1214.349212	18.0	-1209.071966	-3.736600	-1212.655511
conformer_21	-1214.502860	0.252357	0.189413	-1214.543673	-107.2	-1214.291316	-1214.351248	12.6	-1209.077998	-3.732389	-1212.658775
conformer_29	-1214.497583	0.252745	0.190194	-1214.542020	-116.7	-1214.289275	-1214.348814	19.0	-1209.069880	-3.734283	-1212.655394
conformer_26	-1214.498063	0.252624	0.190094	-1214.540866	-112.4	-1214.288242	-1214.347760	21.8	-1209.070084	-3.734985	-1212.654766
conformer_25	-1214.497880	0.252548	0.189949	-1214.536902	-102.5	-1214.284354	-1214.343941	31.8	-1209.071174	-3.733985	-1212.651219

[a]: Single-point calculation in aqueous phase with SMD model.

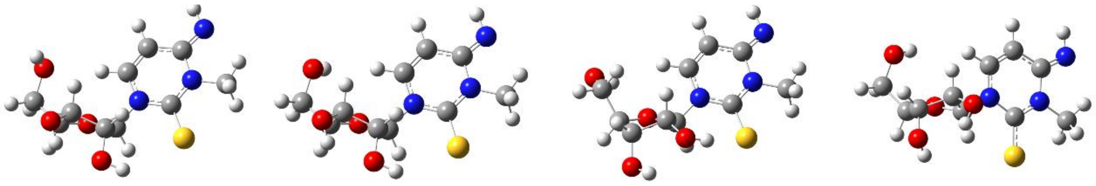

conformer_5 conformer_6 conformer_23 conformer_66

Figure S23. B3LYP-D3/def2-TZVPP optimized geometries of conformers for neutral 2-thio-3-methylcytidine (**1rb2th3mC**).

Table S21. Conformers of gas-phase optimized neutral 2-thio-3-methylcytidine (**1rb2th3mC**) at the B3LYP-D3/def2-TZVPP level of theory followed by aqueous phase single-point calculation. The columns display total energy without zero-point correction (E_{Tot}), thermal correction to enthalpy (δH), Gibbs free energy (δG), total energy without zero-point correction ($E_{Tot,W}$), Gibbs free energy ($G_{298,W}$) in water (W), total single-point energy (E_{CBS}) calculated at DLPNO-CCSD(T)/CBS level of theory, and their corresponding free energy $G_{CBS,W}$. $G_{298,W}$ and $G_{CBS,W}$ have been corrected to the standard state of 1 mol/L by addition of +7.908 kJ/mol. ΔG_{Solv} represents the Gibbs free energy of solvation. The data are arranged in the ascending numeric order of $E_{Tot,W}$. $\Delta G_{298,W}$ represents the respective energy difference to the lowest structure. Only conformers within the 24 kJ/mol (6 kcal/mol) energy window above the lowest in CREST are included in initial conformer sampling. Duplicates of the same structure are excluded. The overall optimum is marked bold.

	B3LYP-D3/def2-TZVPP			SMD(H2O)/B3LYP-D3/def2-TZVPP[a]					DLPNO-CCSD(T)/CBS		
1rb2th3mC No.	E_{Tot} (Hartree)	δH (Hartree)	δG (Hartree)	$E_{Tot,W}$ (Hartree)	ΔG_{Solv} (kJ/mol)	$H_{298,W}$ (Hartree)	$G_{298,W}$ (Hartree)	$\Delta G_{298,W}$ (kJ/mol)	$E_{CBS,HF}$ (Hartree)	$E_{CBS,C}$ (Hartree)	$G_{CBS,W}$ (Hartree)
conformer_5	-1253.826108	0.282751	0.219086	-1253.853401	-71.7	-1253.570650	-1253.631303	2.6	-1248.112991	-3.949351	-1251.867537
conformer_6	-1253.825184	0.282673	0.218367	-1253.853286	-73.8	-1253.570613	-1253.631907	1.1	-1248.111949	-3.949490	-1251.868162
conformer_23	-1253.823516	0.282230	0.218817	-1253.852742	-76.7	-1253.570512	-1253.630913	3.7	-1248.106931	-3.952554	-1251.866882
conformer_15	-1253.825468	0.282272	0.219363	-1253.852667	-71.4	-1253.570395	-1253.630292	5.3	-1248.108843	-3.952243	-1251.865911
conformer_7	-1253.826381	0.282709	0.219040	-1253.852273	-68.0	-1253.569564	-1253.630221	5.5	-1248.114135	-3.948886	-1251.866862
conformer_36	-1253.823119	0.282084	0.217889	-1253.852231	-76.4	-1253.570147	-1253.631330	2.6	-1248.106624	-3.952548	-1251.867382
conformer_87	-1253.818823	0.282508	0.218319	-1253.852150	-87.5	-1253.569642	-1253.630819	3.9	-1248.104289	-3.950717	-1251.867001
conformer_18	-1253.821847	0.282606	0.218431	-1253.852128	-79.5	-1253.569522	-1253.630685	4.3	-1248.107671	-3.950575	-1251.867084
conformer_17	-1253.821125	0.282500	0.217364	-1253.852075	-81.3	-1253.569575	-1253.631699	1.6	-1248.106970	-3.950608	-1251.868152
**conformer_66**	**-1253.815549**	**0.282359**	**0.216734**	**-1253.852054**	**-95.8**	**-1253.569695**	**-1253.632308**	**0**	**-1248.101619**	**-3.949974**	**-1251.868353**
conformer_3	-1253.824518	0.282653	0.218674	-1253.851863	-71.8	-1253.569210	-1253.630177	5.6	-1248.112526	-3.948977	-1251.867162
conformer_47	-1253.815634	0.282383	0.217981	-1253.851750	-94.8	-1253.569367	-1253.630757	4.1	-1248.100830	-3.949970	-1251.865923
conformer_14	-1253.826346	0.282354	0.219463	-1253.851694	-66.6	-1253.569340	-1253.629219	8.1	-1248.110807	-3.951592	-1251.865273
conformer_60	-1253.814734	0.282376	0.217234	-1253.851627	-96.9	-1253.569251	-1253.631381	2.4	-1248.100702	-3.949778	-1251.867127
conformer_9	-1253.823654	0.282238	0.219220	-1253.851600	-73.4	-1253.569362	-1253.629368	7.7	-1248.106431	-3.953458	-1251.865602
conformer_59	-1253.816703	0.282343	0.217637	-1253.851595	-91.6	-1253.569252	-1253.630946	3.6	-1248.101555	-3.951285	-1251.867083
conformer_45	-1253.819306	0.282123	0.218505	-1253.851467	-84.4	-1253.569344	-1253.629950	6.2	-1248.102207	-3.953195	-1251.866046
conformer_12	-1253.821900	0.282142	0.217645	-1253.851444	-77.6	-1253.569302	-1253.630787	4.0	-1248.106933	-3.950983	-1251.866803
conformer_13	-1253.821900	0.282142	0.217645	-1253.851444	-77.6	-1253.569302	-1253.630787	4.0	-1248.106933	-3.950983	-1251.866803
conformer_102	-1253.815714	0.282254	0.217568	-1253.851364	-93.6	-1253.569110	-1253.630784	4.0	-1248.102768	-3.948620	-1251.866458
conformer_26	-1253.821104	0.281998	0.217338	-1253.851353	-79.4	-1253.569355	-1253.631003	3.4	-1248.105648	-3.951398	-1251.866946
conformer_34	-1253.821083	0.282145	0.218773	-1253.851353	-79.5	-1253.569208	-1253.629568	7.2	-1248.103723	-3.953044	-1251.865252
conformer_43	-1253.816999	0.282440	0.218146	-1253.851157	-89.7	-1253.568717	-1253.629999	6.1	-1248.103204	-3.949484	-1251.865689
conformer_76	-1253.815311	0.282332	0.216992	-1253.851050	-93.8	-1253.568718	-1253.631046	3.3	-1248.103245	-3.948345	-1251.867325
conformer_21	-1253.822208	0.282573	0.218545	-1253.850977	-75.5	-1253.568404	-1253.629420	7.6	-1248.108900	-3.950103	-1251.866215
conformer_16	-1253.821900	0.282416	0.217912	-1253.850927	-76.2	-1253.568511	-1253.630003	6.1	-1248.110331	-3.948858	-1251.867292
conformer_112	-1253.821900	0.282416	0.217912	-1253.850927	-76.2	-1253.568511	-1253.630003	6.1	-1248.110331	-3.948858	-1251.867292
conformer_71	-1253.819025	0.282011	0.217365	-1253.850912	-83.7	-1253.568901	-1253.630535	4.7	-1248.101967	-3.953162	-1251.866639
conformer_116	-1253.819025	0.282011	0.217365	-1253.850912	-83.7	-1253.568901	-1253.630535	4.7	-1248.101967	-3.953162	-1251.866639
conformer_61	-1253.822341	0.282104	0.218825	-1253.850843	-74.8	-1253.568739	-1253.629006	8.7	-1248.105169	-3.953961	-1251.865795

conformer_110	-1253.811797	0.282274	0.216593	-1253.850796	-102.4	-1253.568522	-1253.631191	2.9	-1248.097265	-3.950683	-1251.867341
conformer_11	-1253.820372	0.282484	0.217906	-1253.850774	-79.8	-1253.568290	-1253.629856	6.4	-1248.107537	-3.949891	-1251.866912
conformer_83	-1253.817277	0.282288	0.217493	-1253.850682	-87.7	-1253.568394	-1253.630177	5.6	-1248.105449	-3.948034	-1251.866383
conformer_55	-1253.812871	0.282260	0.216614	-1253.850681	-99.3	-1253.568421	-1253.631055	3.3	-1248.100031	-3.948637	-1251.866853
conformer_94	-1253.811902	0.282163	0.216230	-1253.850524	-101.4	-1253.568361	-1253.631282	2.7	-1248.098927	-3.948685	-1251.866992
conformer_29	-1253.822215	0.282250	0.218956	-1253.850436	-74.1	-1253.568186	-1253.628468	10.1	-1248.105896	-3.952539	-1251.864688
conformer_81	-1253.818147	0.282292	0.218059	-1253.850419	-84.7	-1253.568127	-1253.629348	7.8	-1248.104470	-3.950357	-1251.866028
conformer_96	-1253.812911	0.282272	0.217166	-1253.850396	-98.4	-1253.568124	-1253.630218	5.5	-1248.096813	-3.952420	-1251.866540
conformer_104	-1253.811023	0.282293	0.216396	-1253.850369	-103.3	-1253.568076	-1253.630961	3.5	-1248.096322	-3.950631	-1251.866891
conformer_65	-1253.820984	0.282061	0.217634	-1253.850320	-77.0	-1253.568259	-1253.629674	6.9	-1248.106408	-3.950872	-1251.865970
conformer_40	-1253.821637	0.282366	0.217887	-1253.850273	-75.2	-1253.567907	-1253.629374	7.7	-1248.110449	-3.948694	-1251.866881
conformer_27	-1253.822930	0.282125	0.219026	-1253.850183	-71.6	-1253.568058	-1253.628145	10.9	-1248.102519	-3.956382	-1251.864116
conformer_32	-1253.817715	0.282027	0.217030	-1253.850108	-85.0	-1253.568081	-1253.630066	5.9	-1248.102130	-3.951810	-1251.866292
conformer_28	-1253.819007	0.282125	0.218717	-1253.850062	-81.5	-1253.567937	-1253.628333	10.4	-1248.100957	-3.954322	-1251.864604
conformer_1	-1253.824421	0.282645	0.218760	-1253.849870	-66.8	-1253.567225	-1253.628098	11.1	-1248.111623	-3.949608	-1251.864907
conformer_75	-1253.814192	0.282152	0.215879	-1253.849816	-93.5	-1253.567664	-1253.630925	3.6	-1248.103565	-3.947087	-1251.867386
conformer_33	-1253.817593	0.282260	0.217285	-1253.849804	-84.6	-1253.567544	-1253.629507	7.4	-1248.105139	-3.949780	-1251.866833
conformer_117	-1253.811661	0.282234	0.216211	-1253.849753	-100.0	-1253.567519	-1253.630530	4.7	-1248.098868	-3.949183	-1251.866920
conformer_79	-1253.813261	0.282340	0.217810	-1253.849724	-95.7	-1253.567384	-1253.628902	8.9	-1248.098670	-3.950391	-1251.864702
conformer_44	-1253.817740	0.282606	0.219033	-1253.849554	-83.5	-1253.566948	-1253.627509	12.6	-1248.101555	-3.952528	-1251.863852
conformer_115	-1253.812553	0.282182	0.216301	-1253.849553	-97.1	-1253.567371	-1253.630240	5.4	-1248.100302	-3.948333	-1251.866323
conformer_113	-1253.817916	0.281995	0.218310	-1253.849406	-82.7	-1253.567411	-1253.628084	11.1	-1248.099786	-3.955040	-1251.864994
conformer_98	-1253.809115	0.282157	0.215797	-1253.849353	-105.6	-1253.567196	-1253.630544	4.6	-1248.095559	-3.949483	-1251.866472
conformer_91	-1253.818395	0.282262	0.217958	-1253.849317	-81.2	-1253.567055	-1253.628347	10.4	-1248.105497	-3.950038	-1251.865486
conformer_111	-1253.817451	0.282226	0.217331	-1253.849198	-83.4	-1253.566972	-1253.628855	9.1	-1248.105397	-3.949602	-1251.866404
conformer_122	-1253.814578	0.282176	0.217161	-1253.849167	-90.8	-1253.566991	-1253.628994	8.7	-1248.099984	-3.951349	-1251.865750
conformer_114	-1253.816889	0.281938	0.216976	-1253.849026	-84.4	-1253.567088	-1253.629038	8.6	-1248.101603	-3.951812	-1251.865565
conformer_99	-1253.814497	0.282182	0.216108	-1253.848883	-90.3	-1253.566701	-1253.629763	6.7	-1248.104466	-3.946674	-1251.866405
conformer_4	-1253.820236	0.282522	0.218106	-1253.848754	-74.9	-1253.566232	-1253.627636	12.3	-1248.106530	-3.950522	-1251.864452
conformer_52	-1253.818953	0.282029	0.218782	-1253.848697	-78.1	-1253.566668	-1253.626903	14.2	-1248.097717	-3.957394	-1251.863061
conformer_64	-1253.818218	0.282231	0.216738	-1253.848462	-79.4	-1253.566231	-1253.628712	9.4	-1248.107290	-3.947978	-1251.865762
conformer_118	-1253.810513	0.282005	0.215103	-1253.848459	-99.6	-1253.566454	-1253.630344	5.2	-1248.099101	-3.948037	-1251.866969
conformer_30	-1253.819044	0.282506	0.218958	-1253.848268	-76.7	-1253.565762	-1253.626298	15.8	-1248.102735	-3.953092	-1251.863081
conformer_85	-1253.813848	0.282473	0.218686	-1253.848235	-90.3	-1253.565762	-1253.626537	15.2	-1248.097001	-3.953395	-1251.863085
conformer_48	-1253.815211	0.282682	0.218304	-1253.847624	-85.1	-1253.564942	-1253.626308	15.8	-1248.101242	-3.950750	-1251.863088
conformer_105	-1253.814201	0.282100	0.216173	-1253.847481	-87.4	-1253.565381	-1253.628296	10.5	-1248.102282	-3.949110	-1251.865486
conformer_62	-1253.815003	0.282314	0.218490	-1253.846989	-84.0	-1253.564675	-1253.625487	17.9	-1248.097961	-3.954015	-1251.862460
conformer_42	-1253.815339	0.282494	0.218093	-1253.846418	-81.6	-1253.563924	-1253.625313	18.4	-1248.101035	-3.951530	-1251.862539
conformer_93	-1253.811283	0.282591	0.218110	-1253.846280	-91.9	-1253.563689	-1253.625158	18.8	-1248.096612	-3.951553	-1251.862041
conformer_54	-1253.813704	0.282651	0.218254	-1253.845817	-84.3	-1253.563166	-1253.624551	20.4	-1248.099263	-3.950988	-1251.861098
conformer_82	-1253.811282	0.282411	0.217978	-1253.845062	-88.7	-1253.562651	-1253.624072	21.6	-1248.096233	-3.952379	-1251.861402
conformer_100	-1253.809800	0.282593	0.218270	-1253.844495	-91.1	-1253.561902	-1253.623213	23.9	-1248.094630	-3.951838	-1251.859882
conformer_73	-1253.812542	0.282502	0.218069	-1253.844119	-82.9	-1253.561617	-1253.623038	24.3	-1248.097646	-3.951690	-1251.859831
conformer_50	-1253.814769	0.282478	0.218015	-1253.843477	-75.4	-1253.560999	-1253.622450	25.9	-1248.101055	-3.950886	-1251.859623
conformer_119	-1253.808503	0.282477	0.218188	-1253.842907	-90.3	-1253.560430	-1253.621707	27.8	-1248.092869	-3.952549	-1251.858621
conformer_37	-1253.815635	0.282513	0.218157	-1253.842452	-70.4	-1253.559939	-1253.621283	28.9	-1248.102587	-3.950591	-1251.858826
conformer_95	-1253.810816	0.282405	0.217938	-1253.842245	-82.5	-1253.559840	-1253.621295	28.9	-1248.096394	-3.951770	-1251.858643
conformer_101	-1253.811610	0.282420	0.218037	-1253.841191	-77.7	-1253.558771	-1253.620142	31.9	-1248.097838	-3.951513	-1251.857882

[a]: Single-point calculation in aqueous phase with SMD model.

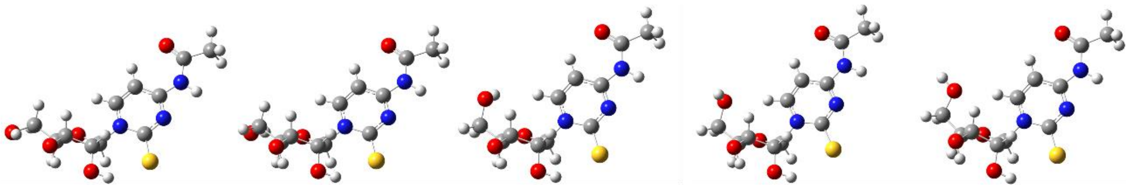

conformer_1 conformer_3 conformer_4 conformer_5 conformer_6

Figure S24. B3LYP-D3/def2-TZVPP optimized geometries of conformers for 2-thio-4-acetylcytidine (**1rb2th4acC**).

Table S22. Conformers of gas-phase optimized 2-thio-4-acetylcytidine (**1rb2th4acC**) at the B3LYP-D3/def2-TZVPP level of theory followed by aqueous phase single-point calculation. The columns display total energy without zero-point correction (E_{Tot}), thermal correction to enthalpy (δH), Gibbs free energy (δG), total energy without zero-point correction ($E_{Tot,W}$), Gibbs free energy ($G_{298,W}$) in water (W), total single-point energy (E_{CBS}) calculated at DLPNO-CCSD(T)/CBS level of theory, and their corresponding free energy $G_{CBS,W}$. $G_{298,W}$ and $G_{CBS,W}$ have been corrected to the standard state of 1 mol/L by addition of +7.908 kJ/mol. ΔG_{Solv} represents the Gibbs free energy of solvation. The data are arranged in the ascending numeric order of $E_{Tot,W}$. $\Delta G_{298,W}$ represents the respective energy difference to the lowest structure. Only conformers within the 24 kJ/mol (6 kcal/mol) energy window above the lowest in CREST are included in initial conformer sampling. Duplicates of the same structure are excluded. The overall optimum is marked bold.

1rb2th4acC No.	B3LYP-D3/def2-TZVPP E_{Tot} (Hartree)	δH (Hartree)	δG (Hartree)	SMD(H2O)/B3LYP-D3/def2-TZVPP[a] $E_{Tot,W}$ (Hartree)	ΔG_{Solv} (kJ/mol)	$H_{298,W}$ (Hartree)	$G_{298,W}$ (Hartree)	$\Delta G_{298,W}$ (kJ/mol)	DLPNO-CCSD(T)/CBS $E_{CBS,HF}$ (Hartree)	$E_{CBS,C}$ (Hartree)	$G_{CBS,W}$ (Hartree)
conformer_10	-1367.233762	0.293511	0.224834	-1367.270901	-97.5	-1366.977390	-1367.043055	3.7	-1360.926460	-4.377346	-1365.113098
conformer_5	-1367.233622	0.294078	0.224399	-1367.270859	-97.8	-1366.976781	-1367.043448	2.6	-1360.931148	-4.373674	-1365.114649
**conformer_4**	**-1367.232625**	**0.293980**	**0.223236**	**-1367.270699**	**-100.0**	**-1366.976719**	**-1367.044451**	**0.0**	**-1360.929917**	**-4.373991**	**-1365.115733**
conformer_15	-1367.231425	0.293350	0.223641	-1367.270644	-103.0	-1366.977294	-1367.043991	1.2	-1360.924197	-4.377620	-1365.114383
conformer_7	-1367.232286	0.293536	0.224645	-1367.270224	-99.6	-1366.976688	-1367.042567	4.9	-1360.925135	-4.378085	-1365.113501
conformer_8	-1367.234720	0.293576	0.224874	-1367.269906	-92.4	-1366.976330	-1367.042020	6.4	-1360.928483	-4.376791	-1365.112575
conformer_11	-1367.230066	0.293404	0.223628	-1367.269884	-104.5	-1366.976480	-1367.043244	3.2	-1360.924431	-4.376200	-1365.113809
conformer_6	-1367.234106	0.294035	0.224218	-1367.269878	-93.9	-1366.975843	-1367.042648	4.7	-1360.932380	-4.373323	-1365.114245
conformer_16	-1367.229353	0.293272	0.223450	-1367.269841	-106.3	-1366.976569	-1367.043379	2.8	-1360.923178	-4.376614	-1365.113818
conformer_19	-1367.231238	0.293527	0.224881	-1367.269741	-101.1	-1366.976214	-1367.041848	6.8	-1360.924438	-4.378229	-1365.113277
conformer_3	-1367.232360	0.293951	0.224020	-1367.269448	-97.4	-1366.975497	-1367.042416	5.3	-1360.930581	-4.373658	-1365.114295
conformer_21	-1367.229237	0.293317	0.223311	-1367.268764	-103.8	-1366.975447	-1367.042441	5.3	-1360.924063	-4.376044	-1365.113311
conformer_14	-1367.229902	0.293749	0.223486	-1367.268742	-102.0	-1366.974993	-1367.042244	5.8	-1360.928567	-4.373474	-1365.114384
conformer_18	-1367.229674	0.293706	0.223473	-1367.268122	-100.9	-1366.974416	-1367.041637	7.4	-1360.928704	-4.373304	-1365.113972
conformer_1	-1367.232279	0.293929	0.224239	-1367.267503	-92.5	-1366.973574	-1367.040252	11.0	-1360.929793	-4.374221	-1365.111988
conformer_43	-1367.219868	0.293708	0.221889	-1367.267408	-124.8	-1366.973700	-1367.042507	5.1	-1360.916799	-4.373512	-1365.112949
conformer_32	-1367.220183	0.293632	0.222495	-1367.267347	-123.8	-1366.973715	-1367.041840	6.9	-1360.916103	-4.373770	-1365.111529
conformer_41	-1367.219037	0.293609	0.221573	-1367.266986	-125.9	-1366.973377	-1367.042401	5.4	-1360.915943	-4.373284	-1365.112591
conformer_26	-1367.221682	0.293703	0.222807	-1367.266803	-118.5	-1366.973100	-1367.040984	9.1	-1360.918558	-4.373339	-1365.111199
conformer_30	-1367.223641	0.293966	0.224697	-1367.266514	-112.6	-1366.972548	-1367.038805	14.8	-1360.917861	-4.376347	-1365.109371
conformer_38	-1367.226172	0.294126	0.224522	-1367.266142	-104.9	-1366.972016	-1367.038608	15.3	-1360.921023	-4.375767	-1365.109227
conformer_36	-1367.217392	0.293550	0.221575	-1367.266026	-127.7	-1366.972476	-1367.041439	7.9	-1360.915195	-4.372448	-1365.111690
conformer_23	-1367.225971	0.293523	0.222107	-1367.265792	-104.6	-1366.972269	-1367.040673	9.9	-1360.925446	-4.372635	-1365.112784
conformer_44	-1367.224667	0.294101	0.223981	-1367.265783	-108.0	-1366.971682	-1367.038790	14.9	-1360.918963	-4.376280	-1365.109367
conformer_40	-1367.224933	0.293677	0.225374	-1367.265528	-106.6	-1366.971851	-1367.037142	19.2	-1360.915125	-4.380299	-1365.107634
conformer_20	-1367.225131	0.293841	0.224530	-1367.265263	-105.4	-1366.971422	-1367.037721	17.7	-1360.919341	-4.376800	-1365.108731
conformer_34	-1367.220844	0.294046	0.223868	-1367.263801	-112.8	-1366.969755	-1367.036921	19.8	-1360.917400	-4.374519	-1365.107996
conformer_28	-1367.221253	0.293871	0.223621	-1367.262732	-108.9	-1366.968861	-1367.036099	21.9	-1360.917583	-4.375124	-1365.107553

[a]: Single-point calculation in aqueous phase with SMD model.

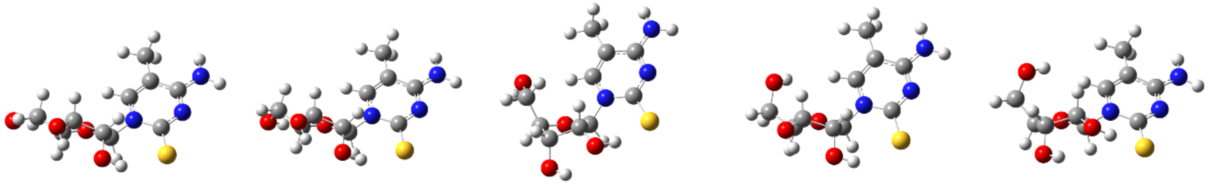

Figure S25. B3LYP-D3/def2-TZVPP optimized geometries of conformers for 2-thio-5-methylcytidine (**1rb2th5mC**).

Table S23. Conformers of gas-phase optimized 2-thio-5-methylcytidine (**1rb2th5mC**) at the B3LYP-D3/def2-TZVPP level of theory followed by aqueous phase single-point calculation. The columns display total energy without zero-point correction (E_{Tot}), thermal correction to enthalpy (δH), Gibbs free energy (δG), total energy without zero-point correction ($E_{Tot,W}$), Gibbs free energy ($G_{298,W}$) in water (W), total single-point energy (E_{CBS}) calculated at DLPNO-CCSD(T)/CBS level of theory, and their corresponding free energy $G_{CBS,W}$. $G_{298,W}$ and $G_{CBS,W}$ have been corrected to the standard state of 1 mol/L by addition of +7.908 kJ/mol. ΔG_{Solv} represents the Gibbs free energy of solvation. The data are arranged in the ascending numeric order of $E_{Tot,W}$. $\Delta G_{298,W}$ represents the respective energy difference to the lowest structure. Only conformers within the 24 kJ/mol (6 kcal/mol) energy window above the lowest in CREST are included in initial conformer sampling. Duplicates of the same structure are excluded. The overall optimum is marked bold.

1rb2th5mC	B3LYP-D3/def2-TZVPP			SMD(H_2O)/B3LYP-D3/def2-TZVPP[a]					DLPNO-CCSD(T)/CBS		
No.	E_{Tot} (Hartree)	δH (Hartree)	δG (Hartree)	$E_{Tot,W}$ (Hartree)	ΔG_{Solv} (kJ/mol)	$H_{298,W}$ (Hartree)	$G_{298,W}$ (Hartree)	$\Delta G_{298,W}$ (kJ/mol)	$E_{CBS,HF}$ (Hartree)	$E_{CBS,C}$ (Hartree)	$G_{CBS,W}$ (Hartree)
conformer_7	-1253.847325	0.281596	0.217315	-1253.885714	-100.8	-1253.604118	-1253.665387	1.3	-1248.132147	-3.947727	-1251.897936
**conformer_23**	**-1253.844576**	**0.281477**	**0.216475**	**-1253.885356**	**-107.1**	**-1253.603879**	**-1253.665869**	**0**	**-1248.129485**	**-3.947838**	**-1251.898615**
conformer_3	-1253.846379	0.281824	0.218143	-1253.885086	-101.6	-1253.603262	-1253.663931	5.1	-1248.130948	-3.948812	-1251.897312
conformer_4	-1253.845920	0.282221	0.216711	-1253.885051	-102.7	-1253.602830	-1253.665328	1.4	-1248.135179	-3.944613	-1251.899200
conformer_15	-1253.845603	0.281718	0.217481	-1253.885043	-103.5	-1253.603325	-1253.664550	3.5	-1248.129635	-3.948944	-1251.897525
conformer_5	-1253.846940	0.282277	0.217983	-1253.884995	-99.9	-1253.602718	-1253.664000	4.9	-1248.136531	-3.944193	-1251.897783
conformer_9	-1253.843419	0.281458	0.216111	-1253.884486	-107.8	-1253.603028	-1253.665363	1.3	-1248.129966	-3.946482	-1251.898393
conformer_19	-1253.847447	0.281637	0.217356	-1253.884482	-97.2	-1253.602845	-1253.664114	4.6	-1248.133474	-3.947103	-1251.897245
conformer_18	-1253.842909	0.281367	0.216200	-1253.884400	-108.9	-1253.603033	-1253.665188	1.8	-1248.128895	-3.946942	-1251.898117
conformer_25	-1253.844533	0.281720	0.218230	-1253.884380	-104.6	-1253.602660	-1253.663138	7.2	-1248.129497	-3.948958	-1251.897060
conformer_10	-1253.846946	0.282221	0.217624	-1253.883833	-96.8	-1253.601612	-1253.663197	7.0	-1248.137426	-3.943748	-1251.897426
conformer_2	-1253.845666	0.281983	0.216319	-1253.883600	-99.6	-1253.601617	-1253.664269	4.2	-1248.136011	-3.944102	-1251.898717
conformer_27	-1253.842270	0.281345	0.215819	-1253.883322	-107.8	-1253.601977	-1253.664491	3.6	-1248.129275	-3.946382	-1251.897877
conformer_14	-1253.843323	0.281843	0.216436	-1253.882908	-103.9	-1253.601065	-1253.663460	6.3	-1248.134054	-3.944030	-1251.898222
conformer_39	-1253.833072	0.281940	0.216356	-1253.882278	-129.2	-1253.600338	-1253.662910	7.8	-1248.121283	-3.943909	-1251.895031
conformer_24	-1253.842745	0.281810	0.216572	-1253.882245	-103.7	-1253.600435	-1253.662661	8.4	-1248.133816	-3.943926	-1251.897658
conformer_1	-1253.845755	0.282143	0.217171	-1253.881757	-94.5	-1253.599614	-1253.661574	11.3	-1248.135370	-3.944666	-1251.895854
conformer_35	-1253.836812	0.282028	0.217516	-1253.881288	-116.8	-1253.599260	-1253.660760	13.4	-1248.123142	-3.946766	-1251.893856
conformer_30	-1253.838968	0.281700	0.214893	-1253.880048	-107.9	-1253.598348	-1253.662143	9.8	-1248.130513	-3.943115	-1251.896802
conformer_26	-1253.838440	0.282051	0.217575	-1253.879961	-109.0	-1253.597910	-1253.659374	17.1	-1248.124805	-3.947113	-1251.892852
conformer_38	-1253.833615	0.282071	0.216187	-1253.877915	-116.3	-1253.595844	-1253.658716	18.8	-1248.122433	-3.944852	-1251.892385
conformer_33	-1253.834110	0.281953	0.215829	-1253.876881	-112.3	-1253.594928	-1253.658040	20.6	-1248.122784	-3.945436	-1251.892149
conformer_34	-1253.833907	0.281975	0.215700	-1253.872864	-102.3	-1253.590889	-1253.654152	30.8	-1248.123784	-3.944391	-1251.888421

[a]: Single-point calculation in aqueous phase with SMD model.

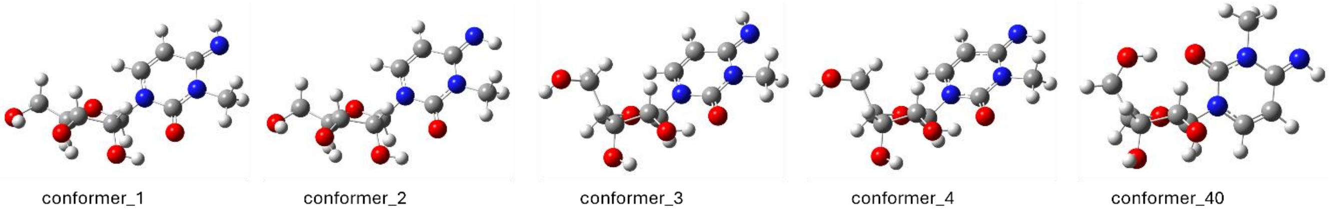

Figure S26. B3LYP-D3/def2-TZVPP optimized geometries of conformers for neutral 3-methylcytidine (**1rb3mC**).

Table S24. Conformers of gas-phase optimized 3-methylcytidine (**1rb3mC**) at the B3LYP-D3/def2-TZVPP level of theory followed by aqueous phase single-point calculation. The columns display total energy without zero-point correction (E_{Tot}), thermal correction to enthalpy (δH), Gibbs free energy (δG), total energy without zero-point correction ($E_{Tot,W}$), Gibbs free energy ($G_{298,W}$) in water (W), total single-point energy (E_{CBS}) calculated at DLPNO-CCSD(T)/CBS level of theory, and their corresponding free energy $G_{CBS,W}$. $G_{298,W}$ and $G_{CBS,W}$ have been corrected to the standard state of 1 mol/L by addition of +7.908 kJ/mol. ΔG_{Solv} represents the Gibbs free energy of solvation. The data are arranged in the ascending numeric order of $E_{Tot,W}$. $\Delta G_{298,W}$ represents the respective energy difference to the lowest structure. Only conformers within the 24 kJ/mol (6 kcal/mol) energy window above the lowest in CREST are included in initial conformer sampling. Duplicates of the same structure are excluded. The overall optimum is marked bold.

	B3LYP-D3/def2-TZVPP			SMD(H2O)/B3LYP-D3/def2-TZVPP[a]					DLPNO-CCSD(T)/CBS		
1rb3mC No.	E_{Tot} (Hartree)	δH (Hartree)	δG (Hartree)	$E_{Tot,W}$ (Hartree)	ΔG_{Solv} (kJ/mol)	$H_{298,W}$ (Hartree)	$G_{298,W}$ (Hartree)	$\Delta G_{298,W}$ (kJ/mol)	$E_{CBS,HF}$ (Hartree)	$E_{CBS,C}$ (Hartree)	$G_{CBS,W}$ (Hartree)
**conformer_40**	**-930.881573**	**0.284888**	**0.222273**	**-930.913892**	**-84.9**	**-930.629004**	**-930.688607**	**0**	**-925.486069**	**-3.993637**	**-929.286740**
conformer_58	-930.882733	0.284854	0.223028	-930.913458	-80.7	-930.628604	-930.687418	3.1	-925.487858	-3.993498	-929.286042
conformer_25	-930.879962	0.284787	0.222822	-930.913423	-87.9	-930.628636	-930.687589	2.7	-925.483129	-3.994674	-929.285430
conformer_14	-930.881893	0.284773	0.222710	-930.913315	-82.5	-930.628542	-930.687593	2.7	-925.486934	-3.993335	-929.285969
conformer_63	-930.882375	0.284783	0.222606	-930.913296	-81.2	-930.628513	-930.687678	2.4	-925.487419	-3.993450	-929.286172
conformer_34	-930.879642	0.284713	0.222445	-930.913250	-88.2	-930.628537	-930.687793	2.1	-925.482721	-3.994643	-929.285515
conformer_20	-930.881572	0.284707	0.222386	-930.913148	-82.9	-930.628441	-930.687750	2.2	-925.486526	-3.993303	-929.286007
conformer_29	-930.877010	0.284666	0.222789	-930.912780	-93.9	-930.628114	-930.686979	4.3	-925.479425	-3.995446	-929.284840
conformer_59	-930.879643	0.284728	0.222959	-930.912754	-86.9	-930.628026	-930.686783	4.8	-925.483966	-3.994365	-929.285471
conformer_68	-930.879737	0.284895	0.222643	-930.912670	-86.5	-930.627775	-930.687015	4.2	-925.484688	-3.993140	-929.285106
conformer_19	-930.878905	0.284654	0.222681	-930.912635	-88.6	-930.627981	-930.686942	4.4	-925.483175	-3.994151	-929.285363
conformer_175	-930.879160	0.284825	0.221847	-930.912282	-87.0	-930.627457	-930.687423	3.1	-925.484038	-3.993055	-929.285356
conformer_96	-930.876771	0.284760	0.222549	-930.912009	-92.5	-930.627249	-930.686448	5.7	-925.480994	-3.993922	-929.284593
conformer_26	-930.881653	0.284714	0.222303	-930.911775	-79.1	-930.627061	-930.686460	5.6	-925.486619	-3.993569	-929.284995
conformer_170	-930.873809	0.284590	0.220239	-930.911710	-99.5	-930.627120	-930.688459	0.4	-925.480208	-3.992148	-929.287006
conformer_173	-930.880329	0.284832	0.222066	-930.911403	-81.6	-930.626571	-930.686325	6.0	-925.487842	-3.990865	-929.284702
conformer_48	-930.879352	0.284847	0.220375	-930.911197	-83.6	-930.626350	-930.687810	2.1	-925.486776	-3.990994	-929.286228
conformer_133	-930.878538	0.284572	0.222269	-930.911082	-85.4	-930.626510	-930.685801	7.4	-925.482731	-3.994400	-929.284394
conformer_9	-930.882961	0.284608	0.221920	-930.911063	-73.8	-930.626455	-930.686131	6.5	-925.486265	-3.995143	-929.284578
conformer_145	-930.871133	0.284509	0.220396	-930.911062	-104.8	-930.626553	-930.687654	2.5	-925.476822	-3.992853	-929.286195
conformer_140	-930.873334	0.284566	0.221351	-930.910958	-98.8	-930.626392	-930.686595	5.3	-925.478755	-3.992223	-929.284239
conformer_185	-930.876403	0.284674	0.221099	-930.910826	-90.4	-930.626152	-930.686715	5.0	-925.482001	-3.992814	-929.285127
conformer_24	-930.881354	0.284719	0.221892	-930.910784	-77.3	-930.626065	-930.685880	7.2	-925.486095	-3.993670	-929.284291
conformer_49	-930.876885	0.284721	0.222135	-930.910745	-88.9	-930.626024	-930.685598	7.9	-925.483440	-3.991795	-929.283949
conformer_30	-930.882390	0.284642	0.221992	-930.910739	-74.4	-930.626097	-930.685735	7.5	-925.486945	-3.993573	-929.283864
conformer_5	-930.881671	0.284739	0.222627	-930.910692	-76.2	-930.625953	-930.685053	9.3	-925.486731	-3.993759	-929.283872
conformer_17	-930.882428	0.284551	0.221261	-930.910675	-74.2	-930.626124	-930.686402	5.8	-925.485808	-3.994964	-929.284746
conformer_31	-930.882428	0.284551	0.221261	-930.910675	-74.2	-930.626124	-930.686402	5.8	-925.485808	-3.994964	-929.284746
conformer_196	-930.876904	0.284843	0.221069	-930.910599	-88.5	-930.625756	-930.686518	5.5	-925.484852	-3.990953	-929.285419
conformer_103	-930.876150	0.284746	0.220708	-930.910532	-90.3	-930.625786	-930.686812	4.7	-925.482696	-3.991839	-929.285197

conformer_129	-930.870156	0.284384	0.219623	-930.910446	-105.8	-930.626062	-930.687811	2.1	-925.476030	-3.992488	-929.286173
conformer_179	-930.873457	0.284514	0.220346	-930.910423	-97.1	-930.625909	-930.687065	4.0	-925.482213	-3.990158	-929.285979
conformer_178	-930.873256	0.284619	0.219652	-930.910417	-97.6	-930.625798	-930.687753	2.2	-925.481938	-3.990012	-929.286448
conformer_128	-930.874766	0.284603	0.221494	-930.910325	-93.4	-930.625722	-930.685819	7.3	-925.481177	-3.991695	-929.283924
conformer_33	-930.880779	0.284640	0.220962	-930.910221	-77.3	-930.625581	-930.686247	6.2	-925.485553	-3.993493	-929.284514
conformer_205	-930.880541	0.284814	0.221946	-930.910215	-77.9	-930.625401	-930.685257	8.8	-925.488982	-3.990346	-929.284044
conformer_166	-930.873893	0.284596	0.221260	-930.910209	-95.3	-930.625613	-930.685937	7.0	-925.478837	-3.993486	-929.284367
conformer_142	-930.873346	0.284584	0.220949	-930.910185	-96.7	-930.625601	-930.686224	6.3	-925.478702	-3.993058	-929.284637
conformer_67	-930.880617	0.284657	0.222562	-930.910161	-77.6	-930.625504	-930.684587	10.6	-925.486248	-3.993640	-929.283859
conformer_27	-930.877966	0.284548	0.221672	-930.910148	-84.5	-930.625600	-930.685464	8.3	-925.481978	-3.994374	-929.283851
conformer_10	-930.879885	0.284463	0.222120	-930.910146	-79.4	-930.625683	-930.685014	9.4	-925.482244	-3.996078	-929.283451
conformer_200	-930.872898	0.284451	0.219242	-930.910141	-97.8	-930.625690	-930.687887	1.9	-925.481651	-3.990050	-929.286691
conformer_143	-930.870871	0.284443	0.219910	-930.910060	-102.9	-930.625617	-930.687138	3.9	-925.478326	-3.990830	-929.285423
conformer_207	-930.872814	0.284530	0.220175	-930.910045	-97.8	-930.625515	-930.686858	4.6	-925.480969	-3.990711	-929.285725
conformer_216	-930.875120	0.284469	0.220888	-930.910019	-91.6	-930.625550	-930.686119	6.5	-925.483534	-3.990185	-929.284717
conformer_192	-930.873959	0.284734	0.221074	-930.909972	-94.6	-930.625238	-930.685886	7.1	-925.481206	-3.991727	-929.284861
conformer_12	-930.877992	0.284589	0.222615	-930.909949	-83.9	-930.625360	-930.684322	11.3	-925.482135	-3.994644	-929.283109
conformer_37	-930.878785	0.284500	0.221919	-930.909932	-81.8	-930.625432	-930.685001	9.5	-925.482492	-3.994432	-929.283140
conformer_106	-930.875569	0.284817	0.222534	-930.909910	-90.2	-930.625093	-930.684364	11.1	-925.479782	-3.994262	-929.282839
conformer_13	-930.883495	0.284667	0.222006	-930.909893	-69.3	-930.625226	-930.684875	9.8	-925.488940	-3.993269	-929.283589
conformer_99	-930.876336	0.284741	0.222279	-930.909888	-88.1	-930.625147	-930.684597	10.5	-925.482687	-3.992544	-929.283493
conformer_88	-930.874841	0.284599	0.220553	-930.909872	-92.0	-930.625273	-930.686307	6.0	-925.479259	-3.993477	-929.284201
conformer_169	-930.870890	0.284416	0.220437	-930.909850	-102.3	-930.625434	-930.686401	5.8	-925.478918	-3.990900	-929.285328
conformer_164	-930.870719	0.284540	0.219870	-930.909806	-102.6	-930.625266	-930.686924	4.4	-925.478742	-3.990766	-929.285713
conformer_210	-930.869836	0.284272	0.218835	-930.909762	-104.8	-930.625490	-930.687915	1.8	-925.477447	-3.990709	-929.286236
conformer_3	-930.880364	0.284592	0.221255	-930.909753	-77.2	-930.625161	-930.685486	8.2	-925.486624	-3.992482	-929.284228
conformer_77	-930.877853	0.284730	0.221262	-930.909659	-83.5	-930.624929	-930.685385	8.5	-925.485617	-3.991487	-929.284636
conformer_180	-930.871702	0.284447	0.221084	-930.909636	-99.6	-930.625189	-930.685540	8.1	-925.475739	-3.993892	-929.283469
conformer_131	-930.875854	0.284672	0.221452	-930.909612	-88.6	-930.624940	-930.685148	9.1	-925.482165	-3.992471	-929.283930
conformer_32	-930.879590	0.284431	0.220657	-930.909610	-78.8	-930.625179	-930.685941	7.0	-925.485448	-3.992826	-929.284625
conformer_126	-930.875090	0.284750	0.221829	-930.909609	-90.6	-930.624859	-930.684768	10.1	-925.479254	-3.994200	-929.283132
conformer_193	-930.877186	0.284712	0.222045	-930.909547	-85.0	-930.624835	-930.684490	10.8	-925.484661	-3.991242	-929.283207
conformer_137	-930.870437	0.284465	0.220961	-930.909513	-102.6	-930.625048	-930.685540	8.1	-925.475027	-3.993821	-929.283951
conformer_73	-930.877182	0.284519	0.222597	-930.909473	-84.8	-930.624954	-930.683864	12.5	-925.481834	-3.994572	-929.283089
conformer_213	-930.869874	0.284425	0.220358	-930.909392	-103.8	-930.624967	-930.686022	6.8	-925.477296	-3.991501	-929.284946
conformer_90	-930.871980	0.284501	0.220834	-930.909391	-98.2	-930.624890	-930.685545	8.0	-925.475669	-3.994227	-929.283460
conformer_204	-930.872519	0.284390	0.221058	-930.909337	-96.7	-930.624947	-930.685267	8.8	-925.480097	-3.991026	-929.283870
conformer_69	-930.877331	0.284645	0.220658	-930.909301	-83.9	-930.624656	-930.685631	7.8	-925.485088	-3.991417	-929.284806
conformer_6	-930.879800	0.284494	0.219939	-930.909284	-77.4	-930.624790	-930.686333	6.0	-925.486090	-3.992361	-929.284983
conformer_100	-930.872555	0.284708	0.222574	-930.909239	-96.3	-930.624531	-930.683653	13.0	-925.476001	-3.995038	-929.282138
conformer_104	-930.873309	0.284626	0.222316	-930.909195	-94.2	-930.624569	-930.683867	12.4	-925.478878	-3.993351	-929.282787
conformer_87	-930.875822	0.284927	0.221811	-930.909133	-87.5	-930.624206	-930.684310	11.3	-925.483248	-3.991212	-929.282949
conformer_42	-930.879031	0.284333	0.219314	-930.909129	-79.0	-930.624796	-930.686803	4.7	-925.484918	-3.992677	-929.285368
conformer_4	-930.877015	0.284483	0.221333	-930.909107	-84.3	-930.624624	-930.684762	10.1	-925.482414	-3.993367	-929.283528
conformer_194	-930.872311	0.284328	0.219160	-930.909090	-96.6	-930.624762	-930.686918	4.4	-925.482221	-3.989123	-929.285952
conformer_199	-930.874041	0.284464	0.220542	-930.908979	-91.7	-930.624515	-930.685425	8.4	-925.482028	-3.991182	-929.284594
conformer_161	-930.874757	0.284622	0.221321	-930.908975	-89.8	-930.624353	-930.684642	10.4	-925.481747	-3.992328	-929.283959
conformer_41	-930.876153	0.284296	0.220271	-930.908936	-86.1	-930.624640	-930.685653	7.8	-925.481171	-3.993662	-929.284333
conformer_50	-930.879659	0.284540	0.221262	-930.908753	-76.4	-930.624213	-930.684479	10.8	-925.486431	-3.992282	-929.283531
conformer_168	-930.875271	0.284832	0.221025	-930.908717	-87.8	-930.623885	-930.684680	10.3	-925.482680	-3.991113	-929.283203
conformer_187	-930.869712	0.284243	0.219300	-930.908470	-101.8	-930.624227	-930.686158	6.4	-925.478865	-3.989903	-929.285214
conformer_156	-930.869512	0.284195	0.218745	-930.908464	-102.3	-930.624269	-930.686707	5.0	-925.476205	-3.991934	-929.285334
conformer_83	-930.872880	0.284826	0.221937	-930.908449	-93.4	-930.623623	-930.683500	13.4	-925.479541	-3.992059	-929.282220
conformer_113	-930.872878	0.284612	0.221131	-930.908392	-93.2	-930.623780	-930.684249	11.4	-925.478017	-3.992976	-929.282365
conformer_8	-930.880519	0.284769	0.221344	-930.908313	-73.0	-930.623544	-930.683957	12.2	-925.488467	-3.991531	-929.283437
conformer_195	-930.870936	0.284352	0.220613	-930.908307	-98.1	-930.623955	-930.684682	10.3	-925.478133	-3.992039	-929.283918

conformer_190	-930.869649	0.284536	0.219761	-930.908192	-101.2	-930.623656	-930.685419	8.4	-925.476718	-3.991362	-929.283849
conformer_53	-930.876388	0.284446	0.221405	-930.908125	-83.3	-930.623679	-930.683708	12.9	-925.482277	-3.993173	-929.282770
conformer_209	-930.875431	0.284498	0.220965	-930.908011	-85.5	-930.623513	-930.684034	12.0	-925.483463	-3.991253	-929.283318
conformer_135	-930.871781	0.284564	0.221032	-930.907970	-95.0	-930.623406	-930.683926	12.3	-925.477506	-3.992727	-929.282378
conformer_115	-930.869932	0.284539	0.221321	-930.907811	-99.5	-930.623272	-930.683478	13.5	-925.474260	-3.993819	-929.281624
conformer_189	-930.874415	0.284514	0.220092	-930.907740	-87.5	-930.623226	-930.684636	10.4	-925.482056	-3.991151	-929.283427
conformer_162	-930.873194	0.284399	0.219684	-930.907720	-90.6	-930.623321	-930.685024	9.4	-925.481223	-3.990919	-929.283972
conformer_163	-930.880203	0.284769	0.221312	-930.907710	-72.2	-930.622941	-930.683386	13.7	-925.489353	-3.990528	-929.283064
conformer_147	-930.871331	0.284506	0.220148	-930.907684	-95.4	-930.623178	-930.684524	10.7	-925.477024	-3.992652	-929.282868
conformer_18	-930.879880	0.284703	0.220233	-930.907638	-72.9	-930.622935	-930.684393	11.1	-925.487753	-3.991545	-929.283812
conformer_15	-930.877208	0.284615	0.221278	-930.907635	-79.9	-930.623020	-930.683345	13.8	-925.484341	-3.992318	-929.282796
conformer_157	-930.872532	0.284756	0.221170	-930.907600	-92.1	-930.622844	-930.683418	13.6	-925.479036	-3.991862	-929.281784
conformer_92	-930.875438	0.284723	0.221617	-930.907480	-84.1	-930.622757	-930.682851	15.1	-925.482598	-3.991764	-929.281775
conformer_153	-930.872164	0.284641	0.221644	-930.907373	-92.4	-930.622732	-930.682717	15.5	-925.478148	-3.992477	-929.281178
conformer_152	-930.870737	0.284366	0.220732	-930.907339	-96.1	-930.622973	-930.683595	13.2	-925.478027	-3.991759	-929.282643
conformer_74	-930.877437	0.284709	0.221450	-930.907309	-78.4	-930.622600	-930.682847	15.1	-925.485590	-3.991078	-929.282079
conformer_22	-930.879528	0.284678	0.218944	-930.907139	-72.5	-930.622461	-930.685183	9.0	-925.488633	-3.990505	-929.284794
conformer_98	-930.874945	0.284646	0.220943	-930.907122	-84.5	-930.622476	-930.683167	14.3	-925.482073	-3.991645	-929.281940
conformer_174	-930.871916	0.284425	0.220227	-930.907088	-92.3	-930.622663	-930.683849	12.5	-925.478903	-3.991883	-929.282720
conformer_16	-930.876871	0.284652	0.221327	-930.907049	-79.2	-930.622397	-930.682710	15.5	-925.485194	-3.991310	-929.282343
conformer_101	-930.876918	0.284632	0.220624	-930.906994	-79.0	-930.622362	-930.683358	13.8	-925.485036	-3.990985	-929.282461
conformer_116	-930.874512	0.284540	0.220733	-930.906964	-85.2	-930.622424	-930.683219	14.1	-925.483329	-3.990389	-929.282425
conformer_45	-930.877879	0.284622	0.221090	-930.906890	-76.2	-930.622268	-930.682788	15.3	-925.487356	-3.990457	-929.282722
conformer_134	-930.874085	0.284482	0.219907	-930.906693	-85.6	-930.622211	-930.683774	12.7	-925.482881	-3.990304	-929.282874
conformer_144	-930.869356	0.284553	0.221787	-930.906693	-98.0	-930.622140	-930.681894	17.6	-925.474596	-3.993347	-929.280480
conformer_201	-930.874715	0.284473	0.219954	-930.906677	-83.9	-930.622204	-930.683711	12.9	-925.483215	-3.990751	-929.282962
conformer_78	-930.874495	0.284602	0.221545	-930.906619	-84.3	-930.622017	-930.682062	17.2	-925.481852	-3.991950	-929.281370
conformer_172	-930.872216	0.284493	0.220428	-930.906606	-90.3	-930.622113	-930.683166	14.3	-925.478838	-3.992144	-929.281932
conformer_177	-930.868266	0.284278	0.219436	-930.906493	-100.4	-930.622215	-930.684045	12.0	-925.473515	-3.993230	-929.282524
conformer_80	-930.877690	0.284574	0.220993	-930.906353	-75.3	-930.621779	-930.682348	16.4	-925.487622	-3.990148	-929.282427
conformer_208	-930.878132	0.284689	0.221258	-930.906346	-74.1	-930.621657	-930.682076	17.1	-925.487037	-3.990718	-929.281700
conformer_182	-930.871790	0.284436	0.219596	-930.906319	-90.7	-930.621883	-930.683711	12.9	-925.478380	-3.992047	-929.282348
conformer_202	-930.871490	0.284441	0.220912	-930.906311	-91.4	-930.621870	-930.682387	16.3	-925.479538	-3.991166	-929.281601
conformer_186	-930.872293	0.284397	0.220118	-930.906027	-88.6	-930.621630	-930.682897	15.0	-925.480117	-3.991486	-929.282207
conformer_1	-930.879960	0.284909	0.222021	-930.906023	-68.4	-930.621114	-930.680990	20.0	-925.488460	-3.990830	-929.280320
conformer_181	-930.869229	0.284404	0.220657	-930.905976	-96.5	-930.621572	-930.682307	16.5	-925.475081	-3.992938	-929.281097
conformer_89	-930.874281	0.284437	0.220930	-930.905671	-82.4	-930.621234	-930.681729	18.1	-925.483342	-3.990979	-929.281769
conformer_71	-930.875090	0.284577	0.221360	-930.905648	-80.2	-930.621071	-930.681276	19.2	-925.483214	-3.991566	-929.280966
conformer_2	-930.876555	0.284740	0.221867	-930.905353	-75.6	-930.620613	-930.680474	21.4	-925.484199	-3.991660	-929.279777

[a]: Single-point calculation in aqueous phase with SMD model.

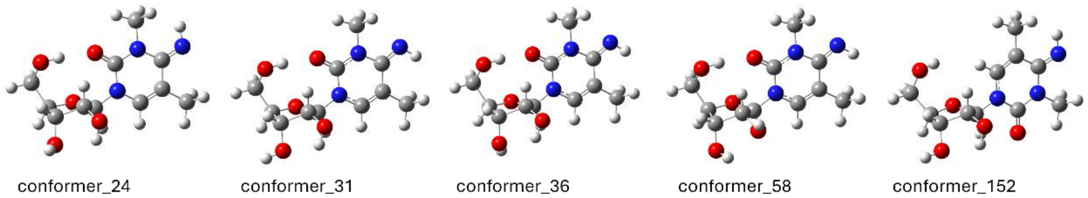

Figure S27. B3LYP-D3/def2-TZVPP optimized geometries of conformers for neutral 3,5-dimethylcytidine (**1rb3m5mC**).

Table S25. Conformers of gas-phase optimized 3,5-dimethylcytidine (**1rb3m5mC**) at the B3LYP-D3/def2-TZVPP level of theory followed by aqueous phase single-point calculation. The columns display total energy without zero-point correction (E_{Tot}), thermal correction to enthalpy (δH), Gibbs free energy (δG), total energy without zero-point correction ($E_{Tot,W}$), Gibbs free energy ($G_{298,W}$) in water (W), total single-point energy (E_{CBS}) calculated at DLPNO-CCSD(T)/CBS level of theory, and their corresponding free energy $G_{CBS,W}$. $G_{298,W}$ and $G_{CBS,W}$ have been corrected to the standard state of 1 mol/L by addition of +7.908 kJ/mol. ΔG_{Solv} represents the Gibbs free energy of solvation. The data are arranged in the ascending numeric order of $E_{Tot,W}$. $\Delta G_{298,W}$ represents the respective energy difference to the lowest structure. Only conformers within the 24 kJ/mol (6 kcal/mol) energy window above the lowest in CREST are included in initial conformer sampling. Duplicates of the same structure are excluded. The overall optimum is marked bold.

1rb3m5mC No.	B3LYP-D3/def2-TZVPP E_{Tot} (Hartree)	δH (Hartree)	δG (Hartree)	SMD(H_2O)/B3LYP-D3/def2-TZVPP[a] $E_{Tot,W}$ (Hartree)	ΔG_{Solv} (kJ/mol)	$H_{298,W}$ (Hartree)	$G_{298,W}$ (Hartree)	$\Delta G_{298,W}$ (kJ/mol)	DLPNO-CCSD(T)/CBS $E_{CBS,HF}$ (Hartree)	$E_{CBS,C}$ (Hartree)	$G_{CBS,W}$ (Hartree)
conformer_58	-970.219369	0.314433	0.249772	-970.249598	-79.4	-969.935165	-969.996814	5.0	-964.541091	-4.204130	-968.522666
conformer_36	-970.216587	0.314333	0.249458	-970.249576	-86.6	-969.935243	-969.997106	4.2	-964.536406	-4.205269	-968.522194
conformer_31	-970.218466	0.314301	0.249363	-970.249505	-81.5	-969.935204	-969.997130	4.2	-964.540150	-4.203924	-968.522738
conformer_24	-970.215818	0.314249	0.248898	-970.248884	-86.8	-969.934635	-969.996974	4.6	-964.536610	-4.204624	-968.522389
conformer_106	-970.216237	0.314301	0.248900	-970.248581	-84.9	-969.934280	-969.996669	5.4	-964.537845	-4.203777	-968.522053
conformer_20	-970.214280	0.314195	0.249314	-970.248154	-88.9	-969.933959	-969.995828	7.6	-964.533771	-4.205631	-968.520950
conformer_50	-970.216895	0.314280	0.249587	-970.248122	-82.0	-969.933842	-969.995523	8.4	-964.538234	-4.204527	-968.521390
conformer_11	-970.216104	0.314158	0.249194	-970.248045	-83.9	-969.933887	-969.995839	7.6	-964.537447	-4.204302	-968.521483
conformer_37	-970.218199	0.314185	0.248865	-970.247643	-77.3	-969.933458	-969.995766	7.7	-964.539748	-4.204247	-968.521562
conformer_18	-970.219543	0.314029	0.248309	-970.247283	-72.8	-969.933254	-969.995962	7.2	-964.539132	-4.206124	-968.521674
conformer_47	-970.216728	0.314197	0.248114	-970.247236	-80.1	-969.933039	-969.996110	6.8	-964.540537	-4.201934	-968.521853
conformer_48	-970.215869	0.314234	0.247433	-970.247160	-82.2	-969.932926	-969.996715	5.3	-964.539638	-4.201931	-968.522415
conformer_124	-970.209370	0.313907	0.246329	-970.247065	-99.0	-969.933158	-969.997724	2.6	-964.532154	-4.202905	-968.523413
conformer_33	-970.213893	0.314123	0.248661	-970.247057	-87.1	-969.932934	-969.995384	8.8	-964.535191	-4.204171	-968.520852
**conformer_152**	**-970.210896**	**0.313880**	**0.245269**	**-970.246998**	**-94.8**	**-969.933118**	**-969.998717**	**0**	**-964.535453**	**-4.201630**	**-968.524904**
conformer_34	-970.218858	0.314108	0.248449	-970.246732	-73.2	-969.932624	-969.995271	9.0	-964.539844	-4.204454	-968.520712
conformer_30	-970.217973	0.314024	0.247947	-970.246666	-75.3	-969.932642	-969.995707	7.9	-964.539248	-4.204514	-968.521497
conformer_10	-970.218302	0.314142	0.248887	-970.246652	-74.4	-969.932510	-969.994753	10.4	-964.539684	-4.204784	-968.520919
conformer_155	-970.209737	0.314001	0.245886	-970.246589	-96.8	-969.932588	-969.997691	2.7	-964.535085	-4.200747	-968.523786
conformer_69	-970.213358	0.314330	0.247457	-970.246473	-86.9	-969.932143	-969.996004	7.1	-964.537981	-4.201553	-968.522181
conformer_156	-970.209879	0.313853	0.246263	-970.246435	-96.0	-969.932582	-969.997160	4.1	-964.535131	-4.201013	-968.523424
conformer_103	-970.208296	0.313878	0.246363	-970.246385	-100.0	-969.932507	-969.997010	4.5	-964.530954	-4.203199	-968.522868
conformer_133	-970.207322	0.313872	0.246151	-970.246314	-102.4	-969.932442	-969.997151	4.1	-964.531424	-4.201452	-968.522705
conformer_65	-970.217095	0.314018	0.248633	-970.246239	-76.5	-969.932221	-969.994594	10.8	-964.538923	-4.204808	-968.521231
conformer_21	-970.215697	0.314002	0.248655	-970.246122	-79.9	-969.932120	-969.994455	11.2	-964.536892	-4.204665	-968.520316
conformer_66	-970.216788	0.314145	0.247874	-970.245963	-76.6	-969.931818	-969.995077	9.6	-964.541595	-4.201312	-968.521197
conformer_43	-970.213765	0.313982	0.247791	-970.245755	-84.0	-969.931773	-969.994952	9.9	-964.537041	-4.202471	-968.520699
conformer_42	-970.213269	0.314045	0.247152	-970.245754	-85.3	-969.931709	-969.995590	8.2	-964.536607	-4.202384	-968.521312
conformer_107	-970.212044	0.314254	0.249030	-970.245675	-88.3	-969.931421	-969.993633	13.3	-964.532938	-4.204954	-968.519481
conformer_4	-970.217137	0.313940	0.248447	-970.245644	-74.8	-969.931704	-969.994185	11.9	-964.536054	-4.206838	-968.519941

conformer_87	-970.207418	0.313755	0.246332	-970.245639	-100.3	-969.931884	-969.996295	6.4	-964.529949	-4.203169	-968.521994
conformer_108	-970.212744	0.314167	0.248692	-970.245626	-86.3	-969.931459	-969.993922	12.6	-964.535764	-4.203251	-968.520192
conformer_62	-970.214252	0.314100	0.247064	-970.245619	-82.4	-969.931519	-969.995543	8.3	-964.538689	-4.202115	-968.522094
conformer_7	-970.216863	0.314123	0.247976	-970.245561	-75.3	-969.931438	-969.994573	10.9	-964.539640	-4.203197	-968.520547
conformer_116	-970.208976	0.313699	0.244964	-970.245547	-96.0	-969.931848	-969.997571	3.0	-964.533295	-4.201910	-968.523800
conformer_38	-970.216138	0.314003	0.247750	-970.245487	-77.1	-969.931484	-969.994725	10.5	-964.538484	-4.203581	-968.520650
conformer_101	-970.207423	0.313888	0.247620	-970.245387	-99.7	-969.931499	-969.994755	10.4	-964.529037	-4.203429	-968.519797
conformer_13	-970.215214	0.313898	0.247901	-970.245200	-78.7	-969.931302	-969.994287	11.6	-964.536188	-4.204818	-968.520080
conformer_119	-970.207893	0.313946	0.246132	-970.245172	-97.9	-969.931226	-969.996028	7.1	-964.533003	-4.201039	-968.522177
conformer_27	-970.215783	0.313912	0.248111	-970.245052	-76.8	-969.931140	-969.993929	12.6	-964.536321	-4.204921	-968.519387
conformer_5	-970.215115	0.313947	0.248583	-970.245052	-78.6	-969.931105	-969.993457	13.8	-964.536002	-4.205250	-968.519595
conformer_78	-970.212271	0.314369	0.248206	-970.245031	-86.0	-969.930662	-969.993813	12.9	-964.536376	-4.201812	-968.519731
conformer_121	-970.207974	0.313700	0.246175	-970.245020	-97.3	-969.931320	-969.995833	7.6	-964.532951	-4.201318	-968.522128
conformer_54	-970.211044	0.314189	0.247399	-970.245000	-89.2	-969.930811	-969.994589	10.8	-964.535381	-4.201879	-968.520806
conformer_19	-970.216148	0.314035	0.246662	-970.244989	-75.7	-969.930954	-969.995315	8.9	-964.538868	-4.203152	-968.521187
conformer_97	-970.205332	0.313733	0.246082	-970.244917	-103.9	-969.931184	-969.995823	7.6	-964.529053	-4.201918	-968.521461
conformer_95	-970.208970	0.313945	0.247780	-970.244790	-94.0	-969.930845	-969.993998	12.4	-964.531574	-4.202889	-968.519490
conformer_144	-970.204333	0.313643	0.245668	-970.244784	-106.2	-969.931141	-969.996104	6.9	-964.527965	-4.201933	-968.521669
conformer_59	-970.209290	0.313930	0.247012	-970.244781	-93.2	-969.930851	-969.994757	10.4	-964.529838	-4.204715	-968.520020
conformer_55	-970.214214	0.313851	0.248441	-970.244747	-80.2	-969.930896	-969.993294	14.2	-964.535413	-4.205424	-968.519917
conformer_51	-970.216076	0.314027	0.247661	-970.244514	-74.7	-969.930487	-969.993841	12.8	-964.539396	-4.202983	-968.520144
conformer_159	-970.209324	0.313799	0.247233	-970.244462	-92.3	-969.930663	-969.994217	11.8	-964.533931	-4.201337	-968.520161
conformer_56	-970.213941	0.313938	0.247593	-970.244449	-80.1	-969.930511	-969.993844	12.8	-964.538214	-4.201868	-968.519986
conformer_148	-970.206930	0.313912	0.246847	-970.244403	-98.4	-969.930491	-969.994544	11.0	-964.531411	-4.201686	-968.520711
conformer_14	-970.217069	0.313961	0.247969	-970.244259	-71.4	-969.930298	-969.993278	14.3	-964.538413	-4.204803	-968.519425
conformer_73	-970.209656	0.314107	0.248833	-970.244212	-90.7	-969.930105	-969.992367	16.7	-964.530212	-4.205290	-968.518213
conformer_88	-970.210331	0.314022	0.248547	-970.244123	-88.7	-969.930101	-969.992564	16.2	-964.533010	-4.203617	-968.518861
conformer_52	-970.211768	0.313937	0.246942	-970.244108	-84.9	-969.930171	-969.994154	12.0	-964.535898	-4.202487	-968.520771
conformer_2	-970.214134	0.313974	0.247789	-970.244063	-78.6	-969.930089	-969.993262	14.3	-964.536456	-4.203679	-968.519263
conformer_117	-970.209259	0.314036	0.247277	-970.244025	-91.3	-969.929989	-969.993736	13.1	-964.530810	-4.203921	-968.519208
conformer_26	-970.213317	0.313847	0.247529	-970.244015	-80.6	-969.930168	-969.993474	13.8	-964.535196	-4.204038	-968.519392
conformer_22	-970.216957	0.314186	0.247681	-970.243756	-70.4	-969.929570	-969.993063	14.8	-964.541414	-4.202366	-968.519886
conformer_93	-970.206813	0.313684	0.245771	-970.243720	-96.9	-969.930036	-969.994937	9.9	-964.530754	-4.202099	-968.520977
conformer_143	-970.209963	0.313939	0.247137	-970.243660	-88.5	-969.929721	-969.993511	13.7	-964.534700	-4.201580	-968.519828
conformer_129	-970.208148	0.314022	0.247503	-970.243562	-93.0	-969.929540	-969.993047	14.9	-964.530570	-4.203342	-968.518811
conformer_25	-970.216592	0.314199	0.247751	-970.243530	-70.7	-969.929331	-969.992767	15.6	-964.542318	-4.201314	-968.519807
conformer_63	-970.209951	0.314202	0.247987	-970.243446	-87.9	-969.929244	-969.992447	16.5	-964.533727	-4.202176	-968.518398
conformer_131	-970.207920	0.313675	0.246388	-970.243431	-93.2	-969.929756	-969.994031	12.3	-964.532265	-4.202209	-968.520585
conformer_94	-970.211875	0.314180	0.247969	-970.243414	-82.8	-969.929234	-969.992433	16.5	-964.535731	-4.202352	-968.518641
conformer_145	-970.208887	0.314183	0.247464	-970.243388	-90.6	-969.929205	-969.992912	15.2	-964.532119	-4.202508	-968.518651
conformer_130	-970.206824	0.313897	0.246045	-970.243384	-96.0	-969.929487	-969.994327	11.5	-964.530918	-4.201702	-968.520122
conformer_120	-970.208619	0.314012	0.247786	-970.243218	-90.8	-969.929206	-969.992420	16.5	-964.531368	-4.203095	-968.518264
conformer_75	-970.213798	0.314153	0.247787	-970.243133	-77.0	-969.928980	-969.992334	16.8	-964.538675	-4.201640	-968.518851
conformer_44	-970.213428	0.313882	0.247483	-970.243045	-77.8	-969.929163	-969.992550	16.2	-964.536274	-4.203461	-968.518857
conformer_158	-970.207077	0.313666	0.245518	-970.243030	-94.4	-969.929364	-969.994500	11.1	-964.533846	-4.199774	-968.521043
conformer_83	-970.206955	0.313922	0.247265	-970.242649	-93.7	-969.928727	-969.992372	16.7	-964.528089	-4.204330	-968.517835
conformer_126	-970.209005	0.313849	0.246860	-970.242340	-87.5	-969.928491	-969.992468	16.4	-964.533019	-4.202232	-968.518715
conformer_6	-970.214285	0.314058	0.247548	-970.242287	-73.5	-969.928229	-969.991727	18.4	-964.538335	-4.202735	-968.518513
conformer_112	-970.207685	0.313840	0.247205	-970.242250	-90.8	-969.928410	-969.992033	17.5	-964.532095	-4.201944	-968.518386
conformer_149	-970.208591	0.313948	0.246939	-970.242237	-88.3	-969.928289	-969.992286	16.9	-964.531876	-4.202780	-968.518350
conformer_96	-970.205945	0.313937	0.247620	-970.242154	-95.1	-969.928217	-969.991522	18.9	-964.528044	-4.203655	-968.517276
conformer_85	-970.213971	0.313916	0.247119	-970.242120	-73.9	-969.928204	-969.991989	17.7	-964.540510	-4.200957	-968.519485
conformer_8	-970.213877	0.314055	0.247597	-970.242044	-74.0	-969.927989	-969.991435	19.1	-964.539183	-4.201705	-968.518446
conformer_68	-970.214489	0.314125	0.247496	-970.242028	-72.3	-969.927903	-969.991520	18.9	-964.540058	-4.201317	-968.518406
conformer_71	-970.209413	0.314035	0.247832	-970.241839	-85.1	-969.927804	-969.990995	20.3	-964.532888	-4.202774	-968.517244
conformer_113	-970.206573	0.313995	0.247157	-970.241827	-92.6	-969.927832	-969.991658	18.5	-964.529495	-4.202856	-968.517435

conformer_86	-970.206456	0.313882	0.247668	-970.241756	-92.7	-969.927874	-969.991076	20.1	-964.528894	-4.203468	-968.516982
conformer_60	-970.211471	0.313991	0.247617	-970.241562	-79.0	-969.927571	-969.990933	20.4	-964.535992	-4.202060	-968.517514
conformer_3	-970.216358	0.314329	0.248280	-970.241465	-65.9	-969.927136	-969.990173	22.4	-964.541428	-4.201628	-968.516871
conformer_122	-970.205314	0.313793	0.246331	-970.241315	-94.5	-969.927522	-969.991972	17.7	-964.527626	-4.203445	-968.517728
conformer_99	-970.208439	0.313880	0.247216	-970.241239	-86.1	-969.927359	-969.991011	20.2	-964.533587	-4.201365	-968.517525
conformer_134	-970.209348	0.313833	0.246774	-970.241203	-83.6	-969.927370	-969.991417	19.2	-964.534246	-4.201799	-968.518114
conformer_41	-970.211301	0.313819	0.247036	-970.241197	-78.5	-969.927378	-969.991149	19.9	-964.536896	-4.201708	-968.518452
conformer_123	-970.206242	0.313857	0.247055	-970.240842	-90.8	-969.926985	-969.990775	20.9	-964.529189	-4.203131	-968.516852
conformer_67	-970.211151	0.313779	0.247005	-970.240655	-77.5	-969.926876	-969.990638	21.2	-964.537217	-4.201401	-968.518105
conformer_57	-970.212054	0.313953	0.247342	-970.240462	-74.6	-969.926509	-969.990108	22.6	-964.537287	-4.201721	-968.517061
conformer_1	-970.213561	0.314202	0.248225	-970.240035	-69.5	-969.925833	-969.988798	26.0	-964.538176	-4.202033	-968.515447
conformer_104	-970.204908	0.313988	0.247048	-970.238990	-89.5	-969.925002	-969.988930	25.7	-964.523530	-4.207586	-968.515138

[a]: Single-point calculation in aqueous phase with SMD model.

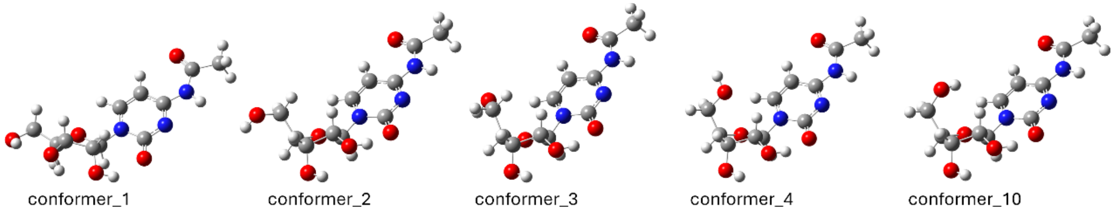

Figure S28. B3LYP-D3/def2-TZVPP optimized geometries of conformers for 4-acetylcytidine (**1rb4acC**).

Table S26. Conformers of gas-phase optimized 4-acetylcytidine (**1rb4acC**) at the B3LYP-D3/def2-TZVPP level of theory followed by aqueous phase single-point calculation. The columns display total energy without zero-point correction (E_{Tot}), thermal correction to enthalpy (δH), Gibbs free energy (δG), total energy without zero-point correction ($E_{Tot,W}$), Gibbs free energy ($G_{298,W}$) in water (W), total single-point energy (E_{CBS}) calculated at DLPNO-CCSD(T)/CBS level of theory, and their corresponding free energy $G_{CBS,W}$. $G_{298,W}$ and $G_{CBS,W}$ have been corrected to the standard state of 1 mol/L by addition of +7.908 kJ/mol. ΔG_{Solv} represents the Gibbs free energy of solvation. The data are arranged in the ascending numeric order of $E_{Tot,W}$. $\Delta G_{298,W}$ represents the respective energy difference to the lowest structure. Only conformers within the 24 kJ/mol (6 kcal/mol) energy window above the lowest in CREST are included in initial conformer sampling. Duplicates of the same structure are excluded. The overall optimum is marked bold.

	B3LYP-D3/def2-TZVPP			SMD(H_2O)/B3LYP-D3/def2-TZVPP[a]					DLPNO-CCSD(T)/CBS		
1rb4acC No.	E_{Tot} (Hartree)	δH (Hartree)	δG (Hartree)	$E_{Tot,W}$ (Hartree)	ΔG_{Solv} (kJ/mol)	$H_{298,W}$ (Hartree)	$G_{298,W}$ (Hartree)	$\Delta G_{298,W}$ (kJ/mol)	$E_{CBS,HF}$ (Hartree)	$E_{CBS,C}$ (Hartree)	$G_{CBS,W}$ (Hartree)
**conformer_10**	**-1044.287910**	**0.295905**	**0.227591**	**-1044.326571**	**-101.5**	**-1044.030666**	**-1044.095968**	**0**	**-1038.298891**	**-4.422627**	**-1042.529577**
conformer_20	-1044.285396	0.296110	0.228307	-1044.326934	-109.1	-1044.030824	-1044.095615	0.9	-1038.297802	-4.421114	-1042.529135
conformer_47	-1044.286261	0.296198	0.228596	-1044.327138	-107.3	-1044.030940	-1044.095530	1.2	-1038.298818	-4.421241	-1042.529329
conformer_38	-1044.284475	0.296038	0.227039	-1044.325576	-107.9	-1044.029538	-1044.095525	1.2	-1038.299448	-4.418858	-1042.529357
conformer_26	-1044.283498	0.296110	0.228404	-1044.326904	-114.0	-1044.030794	-1044.095488	1.3	-1038.294077	-4.422396	-1042.528464
conformer_11	-1044.286517	0.295853	0.227110	-1044.325503	-102.4	-1044.029650	-1044.095381	1.5	-1038.298782	-4.421450	-1042.529095
conformer_8	-1044.288024	0.295898	0.228080	-1044.326099	-100.0	-1044.030201	-1044.095007	2.5	-1038.299435	-4.422028	-1042.528445
conformer_3	-1044.286983	0.295902	0.228147	-1044.325895	-102.2	-1044.029993	-1044.094736	3.2	-1038.299180	-4.421984	-1042.528917
conformer_50	-1044.282861	0.296094	0.228224	-1044.325932	-113.1	-1044.029838	-1044.094696	3.3	-1038.295336	-4.420972	-1042.528143
conformer_2	-1044.285791	0.295889	0.227401	-1044.324652	-102.0	-1044.028763	-1044.094239	4.5	-1038.299238	-4.420423	-1042.528109
conformer_13	-1044.285101	0.295803	0.227413	-1044.324599	-103.7	-1044.028796	-1044.094174	4.7	-1038.298091	-4.420800	-1042.527964
conformer_39	-1044.286053	0.295855	0.228147	-1044.325305	-103.1	-1044.029450	-1044.094146	4.8	-1038.298792	-4.421898	-1042.528783
conformer_4	-1044.289178	0.295949	0.228119	-1044.325097	-94.3	-1044.029148	-1044.093966	5.3	-1038.301549	-4.421633	-1042.527970
conformer_32	-1044.285039	0.295762	0.226830	-1044.323658	-101.4	-1044.027896	-1044.093816	5.6	-1038.299038	-4.420248	-1042.528063
conformer_27	-1044.285027	0.296074	0.228521	-1044.325052	-105.1	-1044.028978	-1044.093519	6.4	-1038.297539	-4.421358	-1042.527389
conformer_30	-1044.284010	0.296029	0.226267	-1044.322702	-101.6	-1044.026673	-1044.093423	6.7	-1038.300277	-4.418449	-1042.528140
conformer_52	-1044.279615	0.296157	0.227057	-1044.323191	-114.4	-1044.027034	-1044.093122	7.5	-1038.294545	-4.419263	-1042.527314
conformer_7	-1044.285502	0.296055	0.227075	-1044.323069	-98.6	-1044.027014	-1044.092982	7.8	-1038.300608	-4.419760	-1042.527848
conformer_69	-1044.276033	0.295769	0.226291	-1044.322265	-121.4	-1044.026496	-1044.092962	7.9	-1038.288550	-4.421004	-1042.526483
conformer_48	-1044.280776	0.296031	0.226940	-1044.322559	-109.7	-1044.026528	-1044.092607	8.8	-1038.295687	-4.419698	-1042.527217
conformer_5	-1044.285191	0.295996	0.226965	-1044.322434	-97.8	-1044.026438	-1044.092457	9.2	-1038.301504	-4.418753	-1042.527523
conformer_67	-1044.279616	0.295879	0.227723	-1044.323045	-114.0	-1044.027166	-1044.092310	9.6	-1038.293521	-4.420499	-1042.526714
conformer_25	-1044.282933	0.295833	0.226517	-1044.321768	-102.0	-1044.025935	-1044.092239	9.8	-1038.299507	-4.418696	-1042.527510
conformer_43	-1044.282736	0.295771	0.226344	-1044.321248	-101.1	-1044.025477	-1044.091892	10.7	-1038.299796	-4.418424	-1042.527376
conformer_64	-1044.278946	0.295997	0.228219	-1044.323097	-115.9	-1044.027100	-1044.091866	10.8	-1038.290800	-4.422095	-1042.525815
conformer_56	-1044.278311	0.296204	0.227288	-1044.321998	-114.7	-1044.025794	-1044.091698	11.2	-1038.293212	-4.419005	-1042.525604
conformer_23	-1044.283790	0.295727	0.227808	-1044.322087	-100.5	-1044.026360	-1044.091267	12.3	-1038.294620	-4.422684	-1042.524781
conformer_31	-1044.281717	0.295910	0.228740	-1044.322522	-107.1	-1044.026612	-1044.090770	13.6	-1038.293874	-4.421270	-1042.524197
conformer_75	-1044.279961	0.295983	0.228019	-1044.321719	-109.6	-1044.025736	-1044.090688	13.9	-1038.288639	-4.424518	-1042.523884
conformer_55	-1044.280926	0.295979	0.227692	-1044.321349	-106.1	-1044.025370	-1044.090645	14.0	-1038.290173	-4.423749	-1042.523641

conformer_36	-1044.279625	0.295875	0.228687	-1044.322329	-112.1	-1044.026454	-1044.090630	14.0	-1038.289953	-4.422599	-1042.523557
conformer_44	-1044.282512	0.296014	0.229081	-1044.322622	-105.3	-1044.026608	-1044.090529	14.3	-1038.294799	-4.421356	-1042.524171
conformer_16	-1044.284586	0.295784	0.228230	-1044.321705	-97.5	-1044.025921	-1044.090463	14.5	-1038.295924	-4.421937	-1042.523739
conformer_57	-1044.279830	0.296000	0.226609	-1044.320036	-105.6	-1044.024036	-1044.090415	14.6	-1038.295579	-4.418803	-1042.524967
conformer_74	-1044.277380	0.295844	0.226514	-1044.319937	-111.7	-1044.024093	-1044.090411	14.6	-1038.293567	-4.418277	-1042.524875
conformer_63	-1044.278117	0.296023	0.227148	-1044.320561	-111.4	-1044.024538	-1044.090401	14.6	-1038.292839	-4.419506	-1042.524629
conformer_41	-1044.281395	0.296011	0.227981	-1044.321143	-104.4	-1044.025132	-1044.090150	15.3	-1038.296140	-4.418874	-1042.523769
conformer_83	-1044.274384	0.295879	0.226920	-1044.320065	-119.9	-1044.024186	-1044.090133	15.3	-1038.287995	-4.420166	-1042.523910
conformer_21	-1044.282661	0.295855	0.228317	-1044.321249	-101.3	-1044.025394	-1044.089920	15.9	-1038.294298	-4.421810	-1042.523367
conformer_28	-1044.281177	0.295632	0.227490	-1044.320346	-102.8	-1044.024714	-1044.089844	16.1	-1038.293947	-4.420943	-1042.523556
conformer_1	-1044.284929	0.296188	0.227700	-1044.320517	-93.4	-1044.024329	-1044.089805	16.2	-1038.300654	-4.419054	-1042.524584
conformer_12	-1044.281871	0.295741	0.227670	-1044.320353	-101.0	-1044.024612	-1044.089671	16.5	-1038.295070	-4.420585	-1042.523455
conformer_9	-1044.283707	0.295908	0.228902	-1044.321510	-99.3	-1044.025602	-1044.089596	16.7	-1038.295784	-4.421910	-1042.523582
conformer_17	-1044.285449	0.295855	0.228305	-1044.320788	-92.8	-1044.024933	-1044.089471	17.1	-1038.297726	-4.421584	-1042.523331
conformer_49	-1044.280727	0.296024	0.226446	-1044.318855	-100.1	-1044.022831	-1044.089397	17.3	-1038.297021	-4.418605	-1042.524296
conformer_46	-1044.282391	0.295777	0.228521	-1044.320854	-101.0	-1044.025077	-1044.089321	17.5	-1038.295167	-4.421733	-1042.523830
conformer_45	-1044.281723	0.295984	0.227863	-1044.320075	-100.7	-1044.024091	-1044.089200	17.8	-1038.297330	-4.418416	-1042.523223
conformer_62	-1044.281620	0.295991	0.228018	-1044.320190	-101.3	-1044.024199	-1044.089160	17.9	-1038.291847	-4.423332	-1042.522720
conformer_34	-1044.281190	0.295903	0.228681	-1044.320695	-103.7	-1044.024792	-1044.089002	18.3	-1038.293441	-4.421494	-1042.522746
conformer_71	-1044.275855	0.295945	0.226818	-1044.318809	-112.8	-1044.022864	-1044.088979	18.3	-1038.290582	-4.419329	-1042.523036
conformer_40	-1044.281101	0.295661	0.227412	-1044.319288	-100.3	-1044.023627	-1044.088864	18.7	-1038.294872	-4.420342	-1042.522977
conformer_35	-1044.280609	0.296014	0.227025	-1044.318423	-99.3	-1044.022409	-1044.088386	19.9	-1038.296692	-4.418229	-1042.522698
conformer_59	-1044.277118	0.295859	0.226821	-1044.318203	-107.9	-1044.022344	-1044.088370	19.9	-1038.291880	-4.419745	-1042.522878
conformer_68	-1044.277671	0.296150	0.227260	-1044.318271	-106.6	-1044.022121	-1044.087999	20.9	-1038.290491	-4.421604	-1042.522422
conformer_19	-1044.281503	0.295911	0.227434	-1044.318378	-96.8	-1044.022467	-1044.087932	21.1	-1038.296390	-4.419825	-1042.522644
conformer_18	-1044.281269	0.295910	0.227507	-1044.318039	-96.5	-1044.022129	-1044.087520	22.2	-1038.297279	-4.418911	-1042.522441
conformer_65	-1044.277385	0.296111	0.227330	-1044.317605	-105.6	-1044.021494	-1044.087263	22.9	-1038.291398	-4.420595	-1042.521871
conformer_33	-1044.279292	0.295762	0.227247	-1044.317378	-100.0	-1044.021616	-1044.087119	23.2	-1038.295694	-4.418730	-1042.522251
conformer_76	-1044.274624	0.296004	0.227561	-1044.317599	-112.8	-1044.021595	-1044.087026	23.5	-1038.289321	-4.419124	-1042.520847
conformer_51	-1044.279062	0.295734	0.227221	-1044.316818	-99.1	-1044.021084	-1044.086585	24.6	-1038.295909	-4.418455	-1042.521886
conformer_73	-1044.276293	0.295808	0.226978	-1044.315736	-103.6	-1044.019928	-1044.085746	26.8	-1038.291848	-4.418916	-1042.520217
conformer_81	-1044.274525	0.295877	0.227565	-1044.316082	-109.1	-1044.020205	-1044.085505	27.5	-1038.288994	-4.419604	-1042.519578
conformer_6	-1044.281063	0.296059	0.228184	-1044.316137	-92.1	-1044.020078	-1044.084941	29.0	-1038.296553	-4.419132	-1042.519563
conformer_58	-1044.277236	0.295843	0.226908	-1044.314691	-98.3	-1044.018848	-1044.084771	29.4	-1038.293337	-4.418721	-1042.519593

[a]: Single-point calculation in aqueous phase with SMD model.

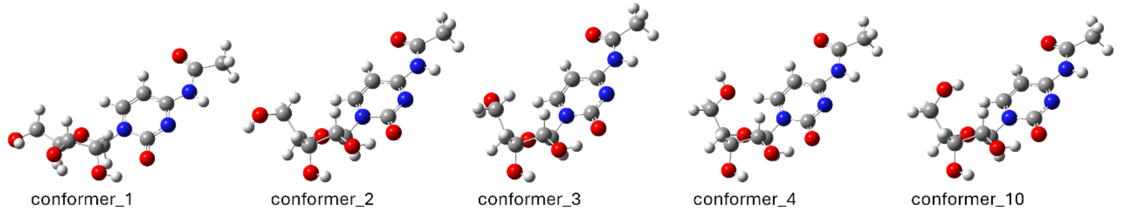

Figure S29. B3LYP-D3/def2-TZVPP optimized geometries of conformers for 4-acetyl-5-methylcytidine (**1rb4ac5mC**).

Table S27. Conformers of gas-phase optimized 4-acetyl-5-methylcytidine (**1rb4ac5mC**) at the B3LYP-D3/def2-TZVPP level of theory followed by aqueous phase single-point calculation. The columns display total energy without zero-point correction (E_{Tot}), thermal correction to enthalpy (δH), Gibbs free energy (δG), total energy without zero-point correction ($E_{Tot,W}$), Gibbs free energy ($G_{298,W}$) in water (W), total single-point energy (E_{CBS}) calculated at DLPNO-CCSD(T)/CBS level of theory, and their corresponding free energy $G_{CBS,W}$. $G_{298,W}$ and $G_{CBS,W}$ have been corrected to the standard state of 1 mol/L by addition of +7.908 kJ/mol. ΔG_{Solv} represents the Gibbs free energy of solvation. The data are arranged in the ascending numeric order of $E_{Tot,W}$. $\Delta G_{298,W}$ represents the respective energy difference to the lowest structure. Only conformers within the 24 kJ/mol (6 kcal/mol) energy window above the lowest in CREST are included in initial conformer sampling. Duplicates of the same structure are excluded. The overall optimum is marked bold.

	B3LYP-D3/def2-TZVPP			SMD(H2O)/B3LYP-D3/def2-TZVPP[a]					DLPNO-CCSD(T)/CBS		
1rb4ac5mC No.	E_{Tot} (Hartree)	δH (Hartree)	δG (Hartree)	$E_{Tot,W}$ (Hartree)	ΔG_{Solv} (kJ/mol)	$H_{298,W}$ (Hartree)	$G_{298,W}$ (Hartree)	$\Delta G_{298,W}$ (kJ/mol)	$E_{CBS,HF}$ (Hartree)	$E_{CBS,C}$ (Hartree)	$G_{CBS,W}$ (Hartree)
conformer_53	-1083.617803	0.325507	0.255524	-1083.657379	-103.9	-1083.331872	-1083.398843	1.7	-1077.345945	-4.632736	-1081.759720
conformer_25	-1083.614946	0.325331	0.254986	-1083.657186	-110.9	-1083.331855	-1083.399188	0.8	-1077.341136	-4.633917	-1081.759295
conformer_21	-1083.616949	0.325339	0.254918	-1083.657146	-105.5	-1083.331807	-1083.399216	0.7	-1077.344983	-4.632600	-1081.759851
**conformer_14**	**-1083.619047**	**0.325202**	**0.254281**	**-1083.656771**	**-99.0**	**-1083.331569**	**-1083.399478**	**0.0**	**-1077.345273**	**-4.634519**	**-1081.760223**
conformer_5	-1083.619737	0.325188	0.254263	-1083.656453	-96.4	-1083.331265	-1083.399178	0.8	-1077.346664	-4.633595	-1081.759700
conformer_1	-1083.619047	0.325264	0.254834	-1083.656228	-97.6	-1083.330964	-1083.398382	2.9	-1077.346626	-4.633716	-1081.759677
conformer_58	-1083.614213	0.325434	0.255023	-1083.656189	-110.2	-1083.330755	-1083.398154	3.5	-1077.342220	-4.632491	-1081.758653
conformer_13	-1083.617995	0.325152	0.254064	-1083.655953	-99.7	-1083.330801	-1083.398877	1.6	-1077.345182	-4.633668	-1081.759732
conformer_27	-1083.616489	0.325423	0.254148	-1083.65579	-103.2	-1083.330367	-1083.398630	2.2	-1077.346750	-4.630649	-1081.759540
conformer_35	-1083.617554	0.325163	0.254422	-1083.655607	-99.9	-1083.330444	-1083.398173	3.4	-1077.345788	-4.633584	-1081.759991
conformer_8	-1083.620377	0.325269	0.254438	-1083.655428	-92.0	-1083.330159	-1083.397978	3.9	-1077.348300	-4.633155	-1081.759056
conformer_23	-1083.616473	0.325351	0.255016	-1083.65537	-102.1	-1083.330019	-1083.397342	5.6	-1077.344549	-4.632869	-1081.758288
conformer_3	-1083.617021	0.325187	0.253934	-1083.654968	-99.6	-1083.329781	-1083.398022	3.8	-1077.345905	-4.632155	-1081.759060
conformer_18	-1083.616387	0.325081	0.253775	-1083.654934	-101.2	-1083.329853	-1083.398147	3.5	-1077.344839	-4.632551	-1081.759151
conformer_28	-1083.616636	0.32538	0.253926	-1083.654649	-99.8	-1083.329269	-1083.397711	4.6	-1077.347815	-4.630130	-1081.759020
conformer_71	-1083.612513	0.325183	0.254106	-1083.654538	-110.3	-1083.329355	-1083.397420	5.4	-1077.339001	-4.634320	-1081.758229
conformer_54	-1083.614897	0.325009	0.253361	-1083.654536	-104.1	-1083.329527	-1083.398163	3.5	-1077.339211	-4.636432	-1081.758908
conformer_42	-1083.615405	0.324971	0.252864	-1083.65438	-102.3	-1083.329409	-1083.398504	2.6	-1077.340184	-4.636260	-1081.759542
conformer_44	-1083.615656	0.325054	0.253693	-1083.654352	-101.6	-1083.329298	-1083.397647	4.8	-1077.340490	-4.635716	-1081.758196
conformer_49	-1083.614111	0.325037	0.253453	-1083.654175	-105.2	-1083.329138	-1083.397710	4.6	-1077.339225	-4.635768	-1081.758591
conformer_34	-1083.614665	0.325128	0.25428	-1083.653995	-103.3	-1083.328867	-1083.396703	7.3	-1077.340207	-4.635810	-1081.758054
conformer_37	-1083.614419	0.325131	0.254111	-1083.653971	-103.8	-1083.328840	-1083.396848	6.9	-1077.340321	-4.635657	-1081.758407
conformer_31	-1083.616177	0.325094	0.253612	-1083.65388	-99.0	-1083.328786	-1083.397256	5.8	-1077.345645	-4.631895	-1081.758619
conformer_48	-1083.615062	0.32497	0.253503	-1083.653869	-101.9	-1083.328899	-1083.397354	5.6	-1077.340317	-4.635572	-1081.758180
conformer_87	-1083.606847	0.325094	0.253339	-1083.65384	-123.4	-1083.328746	-1083.397489	5.2	-1077.335282	-4.631553	-1081.757478
conformer_51	-1083.613750	0.324973	0.25249	-1083.653511	-104.4	-1083.328538	-1083.398009	3.9	-1077.339673	-4.635151	-1081.759083
conformer_91	-1083.613744	0.325082	0.25411	-1083.653385	-104.1	-1083.328303	-1083.396263	8.4	-1077.340144	-4.635663	-1081.758326
conformer_63	-1083.610894	0.325368	0.253003	-1083.653275	-111.3	-1083.327907	-1083.397260	5.8	-1077.341584	-4.630642	-1081.758592
conformer_50	-1083.616434	0.325094	0.253573	-1083.653269	-96.7	-1083.328175	-1083.396684	7.3	-1077.342253	-4.635261	-1081.757765
conformer_78	-1083.61115	0.325263	0.254639	-1083.653242	-110.5	-1083.327979	-1083.395591	10.2	-1077.340617	-4.631956	-1081.757014

conformer_76	-1083.610366	0.325272	0.254771	-1083.653214	-112.5	-1083.327942	-1083.395431	10.6	-1077.337778	-4.633614	-1081.756457
conformer_89	-1083.607966	0.325119	0.253304	-1083.653177	-118.7	-1083.328058	-1083.396861	6.9	-1077.337483	-4.631055	-1081.757433
conformer_40	-1083.616365	0.325069	0.253969	-1083.653028	-96.3	-1083.327959	-1083.396047	9.0	-1077.342335	-4.635664	-1081.757680
conformer_77	-1083.612367	0.325205	0.25404	-1083.652968	-106.6	-1083.327763	-1083.395916	9.4	-1077.338871	-4.634661	-1081.757081
conformer_39	-1083.612949	0.324974	0.2529	-1083.65281	-104.7	-1083.327836	-1083.396898	6.8	-1077.339948	-4.634062	-1081.757959
conformer_61	-1083.612226	0.324862	0.252799	-1083.652727	-106.3	-1083.327865	-1083.396916	6.7	-1077.338756	-4.634470	-1081.757916
conformer_11	-1083.616639	0.325298	0.253593	-1083.652719	-94.7	-1083.327421	-1083.396114	8.8	-1077.347199	-4.631485	-1081.758159
conformer_56	-1083.612154	0.325252	0.253024	-1083.65268	-106.4	-1083.327428	-1083.396644	7.4	-1077.342768	-4.631154	-1081.758413
conformer_24	-1083.615635	0.325337	0.252832	-1083.65266	-97.2	-1083.327323	-1083.396816	7.0	-1077.347508	-4.629871	-1081.758560
conformer_12	-1083.616346	0.325318	0.253867	-1083.652513	-95.0	-1083.327195	-1083.395634	10.1	-1077.348111	-4.630479	-1081.757877
conformer_55	-1083.612375	0.324867	0.252338	-1083.652493	-105.3	-1083.327626	-1083.397143	6.1	-1077.339316	-4.634281	-1081.758364
conformer_38	-1083.612967	0.324972	0.252779	-1083.652471	-103.7	-1083.327499	-1083.396680	7.3	-1077.340315	-4.634008	-1081.758036
conformer_86	-1083.607405	0.325039	0.252515	-1083.652208	-117.6	-1083.327169	-1083.396681	7.3	-1077.335346	-4.632676	-1081.757298
conformer_66	-1083.609656	0.325385	0.253489	-1083.652077	-111.4	-1083.326692	-1083.395576	10.2	-1077.340330	-4.630416	-1081.756665
conformer_22	-1083.61438	0.325099	0.253241	-1083.651817	-98.3	-1083.326718	-1083.395564	10.3	-1077.346562	-4.630293	-1081.758039
conformer_84	-1083.612371	0.324917	0.252667	-1083.651703	-103.3	-1083.326786	-1083.396024	9.1	-1077.340241	-4.633897	-1081.757791
conformer_43	-1083.614055	0.325069	0.253269	-1083.651249	-97.7	-1083.326180	-1083.394968	11.8	-1077.346691	-4.630032	-1081.757636
conformer_47	-1083.612672	0.325236	0.253238	-1083.650796	-100.1	-1083.325560	-1083.394546	12.9	-1077.341459	-4.633270	-1081.756603
conformer_70	-1083.609586	0.325271	0.253585	-1083.650547	-107.5	-1083.325276	-1083.393950	14.5	-1077.340045	-4.630929	-1081.755338
conformer_2	-1083.616166	0.325452	0.254292	-1083.650376	-89.8	-1083.324924	-1083.393072	16.8	-1077.347413	-4.630711	-1081.755030
conformer_65	-1083.611263	0.325214	0.253248	-1083.650308	-102.5	-1083.325094	-1083.394048	14.3	-1077.342768	-4.630252	-1081.755805
conformer_85	-1083.608971	0.325147	0.253159	-1083.650267	-108.4	-1083.325120	-1083.394096	14.1	-1077.340827	-4.629734	-1081.755687
conformer_46	-1083.612372	0.325246	0.253316	-1083.650229	-99.4	-1083.324983	-1083.393901	14.6	-1077.342576	-4.632183	-1081.756287
conformer_75	-1083.611326	0.325182	0.252351	-1083.650208	-102.1	-1083.325026	-1083.394845	12.2	-1077.341245	-4.632028	-1081.756792
conformer_45	-1083.612371	0.32509	0.252585	-1083.65014	-99.2	-1083.325050	-1083.394543	13.0	-1077.342299	-4.632344	-1081.756815
conformer_72	-1083.610262	0.325014	0.252609	-1083.64955	-103.1	-1083.324536	-1083.393929	14.6	-1077.340816	-4.632074	-1081.756556
conformer_73	-1083.610057	0.324988	0.252278	-1083.649406	-103.3	-1083.324418	-1083.394116	14.1	-1077.340212	-4.632296	-1081.756567
conformer_57	-1083.612184	0.325234	0.252864	-1083.649192	-97.2	-1083.323958	-1083.393316	16.2	-1077.344240	-4.630025	-1081.755397
conformer_32	-1083.612192	0.325328	0.253456	-1083.64817	-94.5	-1083.322842	-1083.391702	20.4	-1077.341817	-4.632498	-1081.753826
conformer_33	-1083.612032	0.325324	0.253753	-1083.648074	-94.6	-1083.322750	-1083.391309	21.4	-1077.341437	-4.632673	-1081.753387

[a]: Single-point calculation in aqueous phase with SMD model.

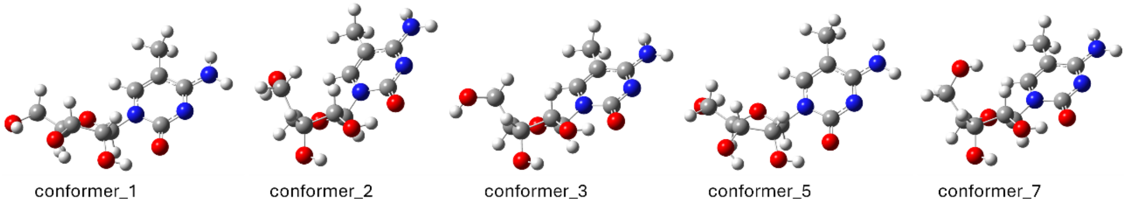

Figure S30. B3LYP-D3/def2-TZVPP optimized geometries of conformers for 5-methylcytidine (**1rb5mC**).

Table S28. Conformers of gas-phase optimized 5-methylcytidine (**1rb5mC**) at the B3LYP-D3/def2-TZVPP level of theory followed by aqueous phase single-point calculation. The columns display total energy without zero-point correction (E_{Tot}), Gibbs free energy (δG), total energy without zero-point correction ($E_{Tot,W}$), Gibbs free energy ($G_{298,W}$) in water (W), total single-point energy (E_{CBS}) calculated at DLPNO-CCSD(T)/CBS level of theory, and their corresponding free energy G_{CBS}. $G_{298,W}$ and G_{CBS} have been corrected to the standard state of 1 mol/L by addition of +7.908 kJ/mol. ΔG_{Solv} represents the Gibbs free energy of solvation. The data are arranged in the ascending numeric order of $E_{Tot,W}$. $\Delta G_{298,W}$ represents the respective energy difference to the lowest structure. Only conformers within the 24 kJ/mol (6 kcal/mol) energy window above the lowest in CREST are included in initial conformer sampling. Duplicates of the same structure are excluded.

	B3LYP-D3/def2-TZVPP			SMD(H2O)/B3LYP-D3/def2-TZVPP[a]					DLPNO-CCSD(T)/CBS		
**1rb5mC No.**	**E_{Tot}** (Hartree)	**δH** (Hartree)	**δG** (Hartree)	**$E_{Tot,W}$** (Hartree)	**ΔG_{Solv}** (kJ/mol)	**$H_{298,W}$** (Hartree)	**$G_{298,W}$** (Hartree)	**$\Delta G_{298,W}$** (kJ/mol)	**$E_{CBS,HF}$** (Hartree)	**$E_{CBS,C}$** (Hartree)	**$G_{CBS,W}$** (Hartree)
conformer_25	-930.899142	0.284342	0.222093	-930.940987	-109.9	-930.656645	-930.715882	2.4	-925.503549	-3.991700	-929.311988
conformer_19	-930.896257	0.284151	0.221356	-930.940726	-116.8	-930.656575	-930.716358	1.1	-925.498659	-3.992882	-929.311642
conformer_16	-930.897969	0.284165	0.221397	-930.940705	-112.2	-930.656540	-930.716296	1.3	-925.502261	-3.991533	-929.312122
**conformer_7**	**-930.900704**	**0.284058**	**0.220222**	**-930.940029**	**-103.2**	**-930.655971**	**-930.716795**	**0**	**-925.503321**	**-3.993280**	**-929.312691**
conformer_26	-930.895709	0.284302	0.221667	-930.939653	-115.4	-930.655351	-930.714974	4.8	-925.499965	-3.991451	-929.310681
conformer_9	-930.900665	0.284050	0.221014	-930.939451	-101.8	-930.655401	-930.715425	3.6	-925.503962	-3.992635	-929.311357
conformer_6	-930.899716	0.284063	0.219654	-930.939327	-104.0	-930.655264	-930.716661	0.4	-925.503283	-3.992563	-929.312792
conformer_12	-930.899716	0.284064	0.219653	-930.939327	-104.0	-930.655263	-930.716662	0.3	-925.503283	-3.992563	-929.312793
conformer_2	-930.900051	0.284158	0.221629	-930.939046	-102.4	-930.654888	-930.714405	6.3	-925.503986	-3.992759	-929.311100
conformer_18	-930.897849	0.284149	0.221430	-930.938762	-107.4	-930.654613	-930.714320	6.5	-925.502191	-3.991929	-929.310591
conformer_23	-930.898516	0.284113	0.221796	-930.938392	-104.7	-930.654279	-930.713584	8.4	-925.503094	-3.992593	-929.310754
conformer_22	-930.896801	0.284234	0.220851	-930.938325	-109.0	-930.654091	-930.714462	6.1	-925.503766	-3.989462	-929.310889
conformer_14	-930.901004	0.284074	0.221179	-930.938157	-97.5	-930.654083	-930.713966	7.4	-925.505389	-3.992128	-929.310479
conformer_3	-930.898445	0.284101	0.220236	-930.938120	-104.2	-930.654019	-930.714872	5.0	-925.503633	-3.991070	-929.311130
conformer_10	-930.897966	0.283963	0.219866	-930.938033	-105.2	-930.654070	-930.715155	4.3	-925.502707	-3.991488	-929.311384
conformer_20	-930.897475	0.283979	0.220169	-930.936961	-103.7	-930.652982	-930.713780	7.9	-925.503256	-3.990873	-929.310433
conformer_37	-930.891700	0.284227	0.221067	-930.936579	-117.8	-930.652352	-930.712500	11.3	-925.495350	-3.992602	-929.308751
conformer_40	-930.892134	0.284099	0.220668	-930.936497	-116.5	-930.652398	-930.712817	10.4	-925.497907	-3.991022	-929.309613
conformer_28	-930.892092	0.284297	0.220142	-930.936163	-115.7	-930.651866	-930.713009	9.9	-925.498907	-3.989802	-929.309625
conformer_33	-930.898264	0.284223	0.219894	-930.935967	-99.0	-930.651744	-930.713061	9.8	-925.505007	-3.990580	-929.310384
conformer_31	-930.893267	0.284133	0.219783	-930.935741	-111.5	-930.651608	-930.712946	10.1	-925.500212	-3.990120	-929.310011
conformer_5	-930.898021	0.284193	0.220229	-930.935579	-98.6	-930.651386	-930.712338	11.7	-925.505966	-3.989587	-929.309870
conformer_32	-930.890803	0.284350	0.220533	-930.935272	-116.8	-930.650922	-930.711727	13.3	-925.497698	-3.989443	-929.308065
conformer_17	-930.896689	0.284194	0.219632	-930.935217	-101.2	-930.651023	-930.712573	11.1	-925.504838	-3.989221	-929.309943
conformer_15	-930.895877	0.284031	0.219829	-930.934989	-102.7	-930.650958	-930.712148	12.2	-925.504178	-3.989388	-929.309836
conformer_24	-930.895365	0.283994	0.219995	-930.934402	-102.5	-930.650408	-930.711395	14.2	-925.504174	-3.989138	-929.309341
conformer_36	-930.891978	0.284082	0.219705	-930.933419	-108.8	-930.649337	-930.710702	16.0	-925.499799	-3.989161	-929.307684
conformer_1	-930.897735	0.284352	0.220592	-930.933259	-93.3	-930.648907	-930.709655	18.7	-925.505334	-3.989710	-929.306964

[a]: Single-point calculation in aqueous phase with SMD model.

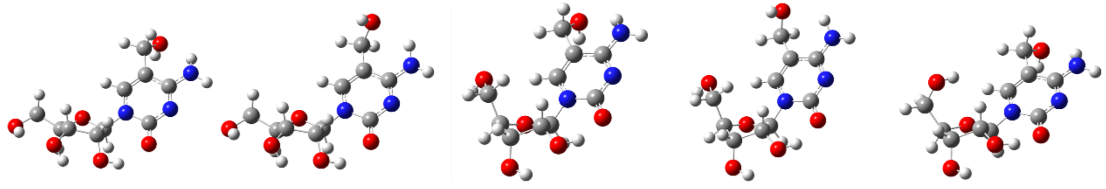

Figure S31. B3LYP-D3/def2-TZVPP optimized geometries of conformers for 5-hydroxymethylcytidine (**1rb5hmC**).

Table S29. Conformers of gas-phase optimized 5-hydroxymethylcytidine (**1rb5hmC**) at the B3LYP-D3/def2-TZVPP level of theory followed by aqueous phase single-point calculation. The columns display total energy without zero-point correction (E_{Tot}), thermal correction to enthalpy (δH), Gibbs free energy (δG), total energy without zero-point correction ($E_{Tot,W}$), Gibbs free energy ($G_{298,W}$) in water (W), total single-point energy (E_{CBS}) calculated at DLPNO-CCSD(T)/CBS level of theory, and their corresponding free energy $G_{CBS,W}$. $G_{298,W}$ and $G_{CBS,W}$ have been corrected to the standard state of 1 mol/L by addition of +7.908 kJ/mol. ΔG_{Solv} represents the Gibbs free energy of solvation. The data are arranged in the ascending numeric order of $E_{Tot,W}$. $\Delta G_{298,W}$ represents the respective energy difference to the lowest structure. Only conformers within the 24 kJ/mol (6 kcal/mol) energy window above the lowest in CREST are included in initial conformer sampling. Duplicates of the same structure are excluded. The overall optimum is marked bold.

1rb5hmC No.	B3LYP-D3/def2-TZVPP			SMD(H₂O)/B3LYP-D3/def2-TZVPP[a]					DLPNO-CCSD(T)/CBS		
	E_{Tot} (Hartree)	δH (Hartree)	δG (Hartree)	$E_{Tot,W}$ (Hartree)	ΔG_{Solv} (kJ/mol)	$H_{298,W}$ (Hartree)	$G_{298,W}$ (Hartree)	$\Delta G_{298,W}$ (kJ/mol)	$E_{CBS,HF}$ (Hartree)	$E_{CBS,C}$ (Hartree)	$G_{CBS,W}$ (Hartree)
conformer_66	-1006.150750	0.290989	0.227242	-1006.196126	-119.1	-1005.905137	-1005.965872	1.9	-1000.395757	-4.263029	-1004.473907
conformer_32	-1006.149729	0.290785	0.226664	-1006.195832	-121.0	-1005.905047	-1005.966156	1.1	-1000.394668	-4.262943	-1004.474039
conformer_35	-1006.149627	0.290857	0.226935	-1006.195810	-121.3	-1005.904953	-1005.965863	1.9	-1000.394465	-4.262883	-1004.473584
conformer_41	-1006.147733	0.290809	0.226815	-1006.195792	-126.2	-1005.904983	-1005.965965	1.6	-1000.390686	-4.264211	-1004.473129
conformer_15	-1006.152108	0.29068	0.225621	-1006.195185	-113.1	-1005.904505	-1005.966552	0.1	-1000.395307	-4.264741	-1004.474492
**conformer_16**	**-1006.152146**	**0.290599**	**0.225488**	**-1006.19509**	**-112.7**	**-1005.904491**	**-1005.966590**	**0**	**-1000.395800**	**-4.264276**	**-1004.474520**
conformer_6	-1006.152729	0.29073	0.226479	-1006.194924	-110.8	-1005.904194	-1005.965433	3.0	-1000.396734	-4.263954	-1004.473391
conformer_8	-1006.152830	0.290706	0.226309	-1006.194889	-110.4	-1005.904183	-1005.965568	2.7	-1000.396744	-4.263911	-1004.473392
conformer_120	-1006.147245	0.290965	0.226801	-1006.194846	-125.0	-1005.903881	-1005.965033	4.1	-1000.392090	-4.262744	-1004.472622
conformer_129	-1006.147131	0.290917	0.226554	-1006.194759	-125.0	-1005.903842	-1005.965193	3.7	-1000.391997	-4.262770	-1004.472830
conformer_3	-1006.152228	0.290904	0.227094	-1006.194739	-111.6	-1005.903835	-1005.964633	5.1	-1000.396750	-4.263974	-1004.473129
conformer_12	-1006.151418	0.290756	0.226071	-1006.19473	-113.7	-1005.903974	-1005.965647	2.5	-1000.395364	-4.264022	-1004.473615
conformer_150	-1006.148678	0.290776	0.226803	-1006.194614	-120.6	-1005.903838	-1005.964799	4.7	-1000.394310	-4.262868	-1004.473299
conformer_4	-1006.152013	0.290822	0.22675	-1006.194532	-111.6	-1005.903710	-1005.964770	4.8	-1000.396793	-4.263937	-1004.473486
conformer_119	-1006.145585	0.290655	0.226480	-1006.194335	-128.0	-1005.903680	-1005.964843	4.6	-1000.389110	-4.264206	-1004.472575
conformer_14	-1006.151043	0.290683	0.225666	-1006.194287	-113.5	-1005.903604	-1005.965609	2.6	-1000.395342	-4.263811	-1004.473719
conformer_110	-1006.147355	0.290645	0.226418	-1006.194285	-123.2	-1005.903640	-1005.964855	4.6	-1000.392826	-4.262831	-1004.473157
conformer_116	-1006.145722	0.290664	0.226522	-1006.194234	-127.4	-1005.903570	-1005.964700	5.0	-1000.389262	-4.264030	-1004.472271
conformer_106	-1006.147477	0.290706	0.226633	-1006.194226	-122.7	-1005.903520	-1005.964581	5.3	-1000.392906	-4.262661	-1004.472672
conformer_65	-1006.150746	0.290781	0.226792	-1006.194025	-113.6	-1005.903244	-1005.964221	6.2	-1000.395932	-4.263816	-1004.473223
conformer_38	-1006.149387	0.290791	0.226522	-1006.193962	-117.0	-1005.903171	-1005.964428	5.7	-1000.394375	-4.263158	-1004.472574
conformer_60	-1006.149229	0.290920	0.225951	-1006.193954	-117.4	-1005.903034	-1005.964991	4.2	-1000.396645	-4.260846	-1004.473253
conformer_37	-1006.149434	0.290813	0.226527	-1006.193939	-116.8	-1005.903126	-1005.964400	5.7	-1000.394428	-4.263173	-1004.472568
conformer_83	-1006.150142	0.290665	0.226384	-1006.193824	-114.7	-1005.903159	-1005.964428	5.7	-1000.395556	-4.263708	-1004.473550
conformer_73	-1006.148862	0.290927	0.225796	-1006.193816	-118.0	-1005.902889	-1005.965008	4.2	-1000.396443	-4.260738	-1004.473327
conformer_48	-1006.150457	0.290628	0.225809	-1006.193757	-113.7	-1005.903129	-1005.964936	4.3	-1000.394223	-4.264597	-1004.473299
conformer_20	-1006.153253	0.290723	0.226268	-1006.193684	-106.2	-1005.902961	-1005.964404	5.7	-1000.398203	-4.263435	-1004.472789
conformer_52	-1006.150112	0.290470	0.225343	-1006.193599	-114.2	-1005.903129	-1005.965244	3.5	-1000.394245	-4.264185	-1004.473561
conformer_23	-1006.152906	0.290744	0.226395	-1006.193572	-106.8	-1005.902828	-1005.964165	6.4	-1000.398003	-4.263359	-1004.472621
conformer_154	-1006.141637	0.290611	0.225783	-1006.193451	-136.0	-1005.902840	-1005.964656	5.1	-1000.385421	-4.263586	-1004.472025

conformer_136	-1006.141902	0.290499	0.225406	-1006.193423	-135.3	-1005.902924	-1005.965005	4.2	-1000.385722	-4.263567	-1004.472392
conformer_5	-1006.150140	0.290602	0.225213	-1006.193397	-113.6	-1005.902795	-1005.965172	3.7	-1000.396023	-4.262356	-1004.473410
conformer_17	-1006.14964	0.290512	0.225221	-1006.193384	-114.9	-1005.902872	-1005.965151	3.8	-1000.395078	-4.262733	-1004.473323
conformer_138	-1006.143359	0.290577	0.225656	-1006.193382	-131.3	-1005.902805	-1005.964714	4.9	-1000.389069	-4.262239	-1004.472663
conformer_50	-1006.150668	0.290602	0.226217	-1006.193350	-112.1	-1005.902748	-1005.964121	6.5	-1000.395069	-4.263816	-1004.472338
conformer_128	-1006.143504	0.290457	0.225263	-1006.193267	-130.7	-1005.902810	-1005.964992	4.2	-1000.389258	-4.262215	-1004.472960
conformer_54	-1006.150190	0.290586	0.226099	-1006.193211	-113.0	-1005.902625	-1005.964100	6.5	-1000.394734	-4.263744	-1004.472388
conformer_42	-1006.149577	0.290679	0.226000	-1006.193206	-114.5	-1005.902527	-1005.964194	6.3	-1000.393988	-4.263979	-1004.472585
conformer_167	-1006.142037	0.290621	0.225846	-1006.193152	-134.2	-1005.902531	-1005.964294	6.0	-1000.385494	-4.263989	-1004.471741
conformer_72	-1006.146500	0.290420	0.224806	-1006.193133	-122.4	-1005.902713	-1005.965315	3.3	-1000.390556	-4.264000	-1004.473371
conformer_28	-1006.149858	0.290713	0.226555	-1006.193116	-113.6	-1005.902403	-1005.963549	8.0	-1000.395132	-4.263804	-1004.472627
conformer_27	-1006.150095	0.290780	0.226904	-1006.193108	-112.9	-1005.902328	-1005.963192	8.9	-1000.395094	-4.263848	-1004.472039
conformer_7	-1006.149945	0.290571	0.225165	-1006.193107	-113.3	-1005.902536	-1005.964930	4.4	-1000.395935	-4.262311	-1004.473232
conformer_145	-1006.143865	0.290651	0.225901	-1006.193106	-129.3	-1005.902455	-1005.964193	6.3	-1000.389192	-4.262684	-1004.472204
conformer_177	-1006.141754	0.290469	0.225035	-1006.193103	-134.8	-1005.902634	-1005.965056	4.0	-1000.385241	-4.264023	-1004.472567
conformer_22	-1006.149390	0.290470	0.225257	-1006.192956	-114.4	-1005.902486	-1005.964687	5.0	-1000.394955	-4.262674	-1004.472926
conformer_82	-1006.145802	0.290327	0.223927	-1006.192941	-123.8	-1005.902614	-1005.966002	1.5	-1000.389246	-4.264528	-1004.473973
conformer_44	-1006.149199	0.290579	0.225500	-1006.192896	-114.7	-1005.902317	-1005.964384	5.8	-1000.394138	-4.263583	-1004.472905
conformer_89	-1006.146664	0.290437	0.224705	-1006.192796	-121.1	-1005.902359	-1005.965079	4.0	-1000.390139	-4.264696	-1004.473250
conformer_163	-1006.149268	0.290852	0.225577	-1006.192741	-114.1	-1005.901889	-1005.964152	6.4	-1000.397626	-4.260349	-1004.472859
conformer_140	-1006.147005	0.290790	0.225601	-1006.192700	-120.0	-1005.901910	-1005.964087	6.6	-1000.394990	-4.260716	-1004.472788
conformer_144	-1006.148628	0.290573	0.226205	-1006.192546	-115.3	-1005.901973	-1005.963329	8.6	-1000.394524	-4.263750	-1004.472975
conformer_26	-1006.147843	0.290702	0.227132	-1006.192538	-117.3	-1005.901836	-1005.962394	11.0	-1000.390878	-4.265602	-1004.471031
conformer_105	-1006.146198	0.290454	0.224922	-1006.192500	-121.6	-1005.902046	-1005.964566	5.3	-1000.389942	-4.264422	-1004.472732
conformer_112	-1006.147413	0.290666	0.226331	-1006.192425	-118.2	-1005.901759	-1005.963082	9.2	-1000.392969	-4.263054	-1004.471692
conformer_162	-1006.148233	0.290613	0.226436	-1006.192391	-115.9	-1005.901778	-1005.962943	9.6	-1000.393974	-4.263652	-1004.472337
conformer_146	-1006.146822	0.290774	0.225684	-1006.192378	-119.6	-1005.901604	-1005.963682	7.6	-1000.394849	-4.260662	-1004.472371
conformer_53	-1006.149260	0.290535	0.224883	-1006.192369	-113.2	-1005.901834	-1005.964474	5.6	-1000.395643	-4.262226	-1004.473083
conformer_39	-1006.147729	0.290721	0.226575	-1006.192165	-116.7	-1005.901444	-1005.962578	10.5	-1000.390205	-4.265195	-1004.470250
conformer_76	-1006.150871	0.290593	0.226038	-1006.192121	-108.3	-1005.901528	-1005.963071	9.2	-1000.396358	-4.263263	-1004.471821
conformer_71	-1006.150769	0.290600	0.225891	-1006.192104	-108.5	-1005.901504	-1005.963201	8.9	-1000.396265	-4.263464	-1004.472161
conformer_103	-1006.145187	0.290501	0.224822	-1006.192079	-123.1	-1005.901578	-1005.964245	6.2	-1000.389762	-4.263592	-1004.472411
conformer_156	-1006.145329	0.290581	0.226540	-1006.192051	-122.7	-1005.901470	-1005.962499	10.7	-1000.388012	-4.265991	-1004.471172
conformer_92	-1006.145521	0.290407	0.224821	-1006.192036	-122.1	-1005.901629	-1005.964203	6.3	-1000.390108	-4.263616	-1004.472405
conformer_67	-1006.148927	0.290445	0.224677	-1006.192011	-113.1	-1005.901566	-1005.964322	6.0	-1000.395485	-4.262091	-1004.472972
conformer_181	-1006.143827	0.290689	0.225297	-1006.191986	-126.4	-1005.901297	-1005.963677	7.6	-1000.389603	-4.262239	-1004.471692
conformer_95	-1006.144872	0.290258	0.223963	-1006.191976	-123.7	-1005.901718	-1005.965001	4.2	-1000.390366	-4.262753	-1004.473248
conformer_109	-1006.146494	0.290481	0.225060	-1006.191923	-119.3	-1005.901442	-1005.963851	7.2	-1000.390768	-4.263801	-1004.471927
conformer_88	-1006.146629	0.290463	0.224721	-1006.191901	-118.9	-1005.901438	-1005.964168	6.4	-1000.390992	-4.263775	-1004.472307
conformer_36	-1006.148046	0.290475	0.225130	-1006.191855	-115.0	-1005.901380	-1005.963713	7.6	-1000.394442	-4.262244	-1004.472354
conformer_59	-1006.147541	0.290374	0.225031	-1006.191803	-116.2	-1005.901429	-1005.963760	7.4	-1000.393480	-4.262643	-1004.472341
conformer_69	-1006.145962	0.290621	0.225734	-1006.191779	-120.3	-1005.901158	-1005.963033	9.3	-1000.390896	-4.263877	-1004.471844
conformer_179	-1006.143345	0.290820	0.226611	-1006.191759	-127.1	-1005.900939	-1005.962136	11.7	-1000.387584	-4.264028	-1004.470403
conformer_193	-1006.143247	0.290836	0.226839	-1006.191747	-127.3	-1005.900911	-1005.961896	12.3	-1000.387459	-4.263974	-1004.470082
conformer_34	-1006.148033	0.290459	0.224773	-1006.191715	-114.7	-1005.901256	-1005.963930	7.0	-1000.394595	-4.262203	-1004.472694
conformer_182	-1006.143936	0.290746	0.226295	-1006.191701	-125.4	-1005.900955	-1005.962394	11.0	-1000.390298	-4.262372	-1004.471128
conformer_137	-1006.143724	0.290913	0.225054	-1006.191680	-125.9	-1005.900767	-1005.963614	7.8	-1000.391252	-4.261063	-1004.472204
conformer_141	-1006.143750	0.290955	0.225408	-1006.191618	-125.7	-1005.900663	-1005.963198	8.9	-1000.391075	-4.261161	-1004.471683
conformer_57	-1006.147607	0.290366	0.224926	-1006.191604	-115.5	-1005.901238	-1005.963666	7.7	-1000.393769	-4.262527	-1004.472355
conformer_107	-1006.145149	0.290404	0.224523	-1006.191590	-121.9	-1005.901186	-1005.964055	6.7	-1000.390173	-4.263286	-1004.472364
conformer_131	-1006.144983	0.290156	0.224055	-1006.191565	-122.3	-1005.901409	-1005.964498	5.5	-1000.391212	-4.262421	-1004.473148
conformer_155	-1006.143121	0.290426	0.224883	-1006.191533	-127.1	-1005.901107	-1005.963638	7.7	-1000.388964	-4.262589	-1004.472071
conformer_148	-1006.143129	0.290552	0.225528	-1006.191449	-126.9	-1005.900897	-1005.962909	9.7	-1000.388879	-4.262609	-1004.471269
conformer_9	-1006.149823	0.290845	0.225548	-1006.191391	-109.1	-1005.900546	-1005.962831	9.9	-1000.397220	-4.261802	-1004.472030
conformer_94	-1006.146942	0.290488	0.225347	-1006.191296	-116.4	-1005.900808	-1005.962937	9.6	-1000.391722	-4.263672	-1004.471388
conformer_78	-1006.147042	0.290497	0.225255	-1006.191279	-116.1	-1005.900782	-1005.963012	9.4	-1000.392328	-4.263316	-1004.471614

conformer_11	-1006.149797	0.290857	0.225623	-1006.191253	-108.8	-1005.900396	-1005.962618	10.4	-1000.397352	-4.261732	-1004.471905
conformer_97	-1006.143267	0.290149	0.223457	-1006.191197	-125.8	-1005.901048	-1005.964728	4.9	-1000.389457	-4.262241	-1004.473159
conformer_125	-1006.145026	0.290836	0.225243	-1006.191173	-121.2	-1005.900337	-1005.962918	9.6	-1000.392485	-4.261438	-1004.471815
conformer_172	-1006.143381	0.290533	0.225343	-1006.191170	-125.5	-1005.900637	-1005.962815	9.9	-1000.388860	-4.262937	-1004.471231
conformer_79	-1006.147911	0.290514	0.225194	-1006.191159	-113.5	-1005.900645	-1005.962953	9.5	-1000.392950	-4.263726	-1004.471718
conformer_170	-1006.143440	0.290536	0.225316	-1006.191142	-125.2	-1005.900606	-1005.962814	9.9	-1000.388906	-4.262969	-1004.471248
conformer_123	-1006.144978	0.290773	0.224867	-1006.191079	-121.0	-1005.900306	-1005.963200	8.9	-1000.392568	-4.261459	-1004.472249
conformer_68	-1006.143600	0.290260	0.223601	-1006.191022	-124.5	-1005.900762	-1005.964409	5.7	-1000.390010	-4.262007	-1004.472825
conformer_185	-1006.144495	0.290187	0.224243	-1006.191018	-122.1	-1005.900831	-1005.963763	7.4	-1000.390178	-4.263042	-1004.472488
conformer_126	-1006.147222	0.290445	0.224860	-1006.190891	-114.7	-1005.900446	-1005.963019	9.4	-1000.394265	-4.262111	-1004.472173
conformer_13	-1006.149433	0.290793	0.225474	-1006.190885	-108.8	-1005.900092	-1005.962399	11.0	-1000.398215	-4.260745	-1004.471927
conformer_74	-1006.144277	0.290416	0.224513	-1006.190880	-122.4	-1005.900464	-1005.963355	8.5	-1000.390618	-4.262203	-1004.471900
conformer_111	-1006.142927	0.290161	0.223879	-1006.190864	-125.9	-1005.900703	-1005.963973	6.9	-1000.388914	-4.262309	-1004.472269
conformer_114	-1006.143700	0.290297	0.224478	-1006.190843	-123.8	-1005.900546	-1005.963353	8.5	-1000.389640	-4.262536	-1004.471830
conformer_159	-1006.142451	0.290976	0.225637	-1006.190808	-127.0	-1005.899832	-1005.962159	11.6	-1000.389852	-4.260801	-1004.470360
conformer_31	-1006.148671	0.290807	0.224787	-1006.190803	-110.6	-1005.899996	-1005.963004	9.4	-1000.397548	-4.260324	-1004.472204
conformer_135	-1006.147021	0.290372	0.224570	-1006.190742	-114.8	-1005.900370	-1005.963160	9.0	-1000.393937	-4.262084	-1004.472159
conformer_10	-1006.149575	0.290769	0.225311	-1006.190739	-108.1	-1005.899970	-1005.962416	11.0	-1000.398249	-4.260788	-1004.471877
conformer_33	-1006.148514	0.290881	0.225158	-1006.190704	-110.8	-1005.899823	-1005.962534	10.6	-1000.397499	-4.260256	-1004.471775
conformer_77	-1006.144036	0.290353	0.223966	-1006.190664	-122.4	-1005.900311	-1005.963686	7.6	-1000.390311	-4.262310	-1004.472271
conformer_149	-1006.142550	0.290981	0.225621	-1006.190648	-126.3	-1005.899667	-1005.962015	12.0	-1000.389976	-4.260804	-1004.470244
conformer_108	-1006.147252	0.290458	0.223151	-1006.190639	-113.9	-1005.900181	-1005.964476	5.6	-1000.392647	-4.263233	-1004.473104
conformer_122	-1006.143344	0.290201	0.223244	-1006.190583	-124.0	-1005.900382	-1005.964327	5.9	-1000.389234	-4.262623	-1004.472840
conformer_157	-1006.143156	0.290227	0.223801	-1006.190281	-123.7	-1005.900054	-1005.963468	8.2	-1000.390242	-4.261803	-1004.472357
conformer_30	-1006.147456	0.290590	0.224863	-1006.190242	-112.3	-1005.899652	-1005.962367	11.1	-1000.396641	-4.260559	-1004.472111
conformer_29	-1006.147643	0.290632	0.224977	-1006.190126	-111.5	-1005.899494	-1005.962137	11.7	-1000.396671	-4.260580	-1004.471746
conformer_191	-1006.142866	0.290119	0.223478	-1006.190099	-124.0	-1005.899980	-1005.963609	7.8	-1000.389122	-4.262981	-1004.472846
conformer_124	-1006.143201	0.290150	0.223542	-1006.190071	-123.1	-1005.899921	-1005.963517	8.1	-1000.390727	-4.261598	-1004.472641
conformer_180	-1006.143645	0.290388	0.224226	-1006.190042	-121.8	-1005.899654	-1005.962804	9.9	-1000.390443	-4.262123	-1004.471725
conformer_175	-1006.142652	0.289999	0.223012	-1006.190002	-124.3	-1005.900003	-1005.963978	6.9	-1000.389779	-4.261855	-1004.472959
conformer_45	-1006.147873	0.290751	0.225354	-1006.189924	-110.4	-1005.899173	-1005.961558	13.2	-1000.395798	-4.261710	-1004.471193
conformer_43	-1006.147902	0.290741	0.225330	-1006.189731	-109.8	-1005.898990	-1005.961389	13.7	-1000.395978	-4.261657	-1004.471122
conformer_102	-1006.147014	0.290561	0.224892	-1006.189704	-112.1	-1005.899143	-1005.961800	12.6	-1000.396669	-4.260306	-1004.471762
conformer_121	-1006.147055	0.290604	0.226247	-1006.189558	-111.6	-1005.898954	-1005.960299	16.5	-1000.389094	-4.266193	-1004.468530
conformer_87	-1006.147221	0.290572	0.224883	-1006.189541	-111.1	-1005.898969	-1005.961646	13.0	-1000.396763	-4.260333	-1004.471521
conformer_192	-1006.139465	0.290632	0.224292	-1006.189506	-131.4	-1005.898874	-1005.962202	11.5	-1000.384140	-4.263222	-1004.470099
conformer_47	-1006.147585	0.290706	0.225305	-1006.189438	-109.9	-1005.898732	-1005.961121	14.4	-1000.396953	-4.260594	-1004.471084
conformer_169	-1006.142391	0.290809	0.225394	-1006.189347	-123.3	-1005.898538	-1005.960941	14.8	-1000.389672	-4.261294	-1004.469517
conformer_101	-1006.146432	0.290754	0.224883	-1006.189309	-112.6	-1005.898555	-1005.961414	13.6	-1000.395989	-4.260092	-1004.471063
conformer_96	-1006.146529	0.290682	0.224790	-1006.189222	-112.1	-1005.898540	-1005.961420	13.6	-1000.395911	-4.260286	-1004.471088
conformer_46	-1006.147644	0.290649	0.225145	-1006.189219	-109.2	-1005.898570	-1005.961062	14.5	-1000.396829	-4.260698	-1004.470945
conformer_80	-1006.143615	0.290527	0.223997	-1006.189120	-119.5	-1005.898593	-1005.962111	11.8	-1000.392052	-4.260910	-1004.471457
conformer_161	-1006.142458	0.290770	0.225287	-1006.189074	-122.4	-1005.898304	-1005.960775	15.3	-1000.389761	-4.261319	-1004.469398
conformer_75	-1006.143795	0.290577	0.224403	-1006.189015	-118.7	-1005.898438	-1005.961600	13.1	-1000.392085	-4.261099	-1004.470990
conformer_171	-1006.143819	0.290699	0.224850	-1006.188979	-118.6	-1005.898280	-1005.961117	14.4	-1000.392187	-4.260588	-1004.470073
conformer_176	-1006.143890	0.290738	0.225031	-1006.188931	-118.3	-1005.898193	-1005.960888	15.0	-1000.392197	-4.260588	-1004.469783
conformer_189	-1006.143890	0.290738	0.225031	-1006.188931	-118.3	-1005.898193	-1005.960888	15.0	-1000.392196	-4.260589	-1004.469783
conformer_2	-1006.149277	0.290946	0.226081	-1006.188834	-103.9	-1005.897888	-1005.959741	18.0	-1000.397556	-4.261004	-1004.469024
conformer_84	-1006.145590	0.290510	0.224771	-1006.188825	-113.5	-1005.898315	-1005.961042	14.6	-1000.395344	-4.260416	-1004.471212
conformer_98	-1006.143931	0.290671	0.224677	-1006.188794	-117.8	-1005.898123	-1005.961105	14.4	-1000.391873	-4.261515	-1004.470562
conformer_1	-1006.149418	0.290962	0.226157	-1006.188671	-103.1	-1005.897709	-1005.959502	18.6	-1000.397626	-4.261000	-1004.468711
conformer_90	-1006.143924	0.290536	0.224148	-1006.188645	-117.4	-1005.898109	-1005.961485	13.4	-1000.391955	-4.261402	-1004.470918
conformer_86	-1006.145485	0.290486	0.224732	-1006.188555	-113.1	-1005.898069	-1005.960811	15.2	-1000.394989	-4.260527	-1004.470842
conformer_91	-1006.143704	0.290642	0.224747	-1006.188466	-117.5	-1005.897824	-1005.960707	15.4	-1000.392907	-4.260487	-1004.470397
conformer_153	-1006.141766	0.290459	0.223717	-1006.188308	-122.2	-1005.897849	-1005.961579	13.2	-1000.391254	-4.259968	-1004.471035
conformer_165	-1006.141425	0.290466	0.223748	-1006.188286	-123.0	-1005.897820	-1005.961526	13.3	-1000.390901	-4.259989	-1004.470990

conformer_187	-1006.145110	0.290478	0.224776	-1006.188280	-113.3	-1005.897802	-1005.960492	16.0	-1000.395341	-4.260159	-1004.470882
conformer_85	-1006.143079	0.290398	0.223873	-1006.188221	-118.5	-1005.897823	-1005.961336	13.8	-1000.392543	-4.260241	-1004.471041
conformer_81	-1006.143083	0.290425	0.223813	-1006.188191	-118.4	-1005.897766	-1005.961366	13.7	-1000.392618	-4.260180	-1004.471081
conformer_99	-1006.143586	0.290504	0.224175	-1006.188154	-117.0	-1005.897650	-1005.960967	14.8	-1000.392783	-4.260527	-1004.470691
conformer_127	-1006.143472	0.290340	0.223540	-1006.188008	-116.9	-1005.897668	-1005.961456	13.5	-1000.392866	-4.260572	-1004.471423
conformer 132	-1006.140993	0.290267	0.223541	-1006.187822	-122.9	-1005.897555	-1005.961269	14.0	-1000.390791	-4.259999	-1004.471066
conformer_152	-1006.141585	0.290459	0.224287	-1006.187754	-121.2	-1005.897295	-1005.960455	16.1	-1000.391181	-4.260304	-1004.470355
conformer 133	-1006.144794	0.290764	0.224702	-1006.187464	-112.0	-1005.896700	-1005.959750	18.0	-1000.393620	-4.260464	-1004.469040
conformer 134	-1006.144869	0.290779	0.224743	-1006.187457	-111.8	-1005.896678	-1005.959702	18.1	-1000.393607	-4.260494	-1004.468934
conformer 24	-1006.147434	0.290848	0.225896	-1006.187392	-104.9	-1005.896544	-1005.958484	21.3	-1000.396304	-4.260836	-1004.468191
conformer_164	-1006.141336	0.290359	0.223824	-1006.187355	-120.8	-1005.896996	-1005.960519	15.9	-1000.390834	-4.260401	-1004.470418
conformer 25	-1006.147355	0.290829	0.225884	-1006.187127	-104.4	-1005.896298	-1005.958231	21.9	-1000.396106	-4.260904	-1004.467886
conformer 142	-1006.142767	0.290270	0.223471	-1006.187017	-116.2	-1005.896747	-1005.960534	15.9	-1000.393349	-4.259691	-1004.470807
conformer_55	-1006.143474	0.290734	0.225000	-1006.186354	-112.6	-1005.895620	-1005.958342	21.7	-1000.392128	-4.260807	-1004.467803
conformer_49	-1006.142885	0.290640	0.224746	-1006.186294	-114.0	-1005.895654	-1005.958536	21.1	-1000.391926	-4.260388	-1004.467965
conformer_56	-1006.142621	0.290572	0.224637	-1006.186265	-114.6	-1005.895693	-1005.958616	20.9	-1000.391590	-4.260488	-1004.468073
conformer_61	-1006.143317	0.290749	0.225085	-1006.186046	-112.2	-1005.895297	-1005.957949	22.7	-1000.391965	-4.260799	-1004.467396
conformer_115	-1006.142215	0.290383	0.223981	-1006.185056	-112.5	-1005.894673	-1005.958063	22.4	-1000.392329	-4.259930	-1004.468106
conformer 183	-1006.136770	0.291004	0.227066	-1006.183038	-121.5	-1005.892034	-1005.952960	35.8	-1000.372909	-4.272514	-1004.461613

[a]: Single-point calculation in aqueous phase with SMD model.

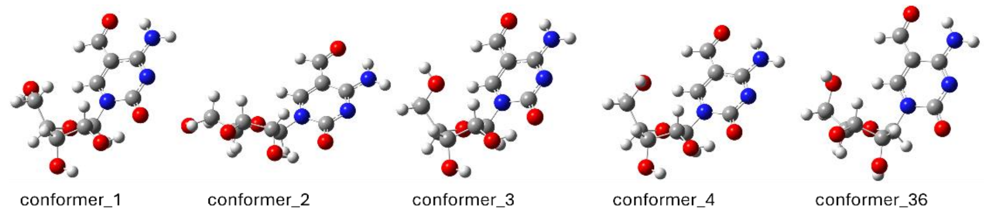

Figure S32. B3LYP-D3/def2-TZVPP optimized geometries of conformers for 5-formylcytidine (**1rb5fC**).

Table S30. Conformers of gas-phase optimized 5-formylcytidine (**1rb5fC**) at the B3LYP-D3/def2-TZVPP level of theory followed by aqueous phase single-point calculation. The columns display total energy without zero-point correction (E_{Tot}), thermal correction to enthalpy (δH), Gibbs free energy (δG), total energy without zero-point correction ($E_{Tot,W}$), Gibbs free energy ($G_{298,W}$) in water (W), total single-point energy (E_{CBS}) calculated at DLPNO-CCSD(T)/CBS level of theory, and their corresponding free energy $G_{CBS,W}$. $G_{298,W}$ and $G_{CBS,W}$ have been corrected to the standard state of 1 mol/L by addition of +7.908 kJ/mol. ΔG_{Solv} represents the Gibbs free energy of solvation. The data are arranged in the ascending numeric order of $E_{Tot,W}$. $\Delta G_{298,W}$ represents the respective energy difference to the lowest structure. Only conformers within the 24 kJ/mol (6 kcal/mol) energy window above the lowest in CREST are included in initial conformer sampling. Duplicates of the same structure are excluded. The overall optimum is marked bold.

	B3LYP-D3/def2-TZVPP			SMD(H2O)/B3LYP-D3/def2-TZVPP[a]					DLPNO-CCSD(T)/CBS		
1rb5fC No.	E_{Tot} (Hartree)	δH (Hartree)	δG (Hartree)	$E_{Tot,W}$ (Hartree)	ΔG_{Solv} (kJ/mol)	$H_{298,W}$ (Hartree)	$G_{298,W}$ (Hartree)	$\Delta G_{298,W}$ (kJ/mol)	$E_{CBS,HF}$ (Hartree)	$E_{CBS,C}$ (Hartree)	$G_{CBS,W}$ (Hartree)
conformer_23	-1004.946847	0.266590	0.204108	-1004.987772	-107.4	-1004.721182	-1004.780652	5.7	-999.237133	-4.215404	-1003.286341
conformer_9	-1004.948600	0.266272	0.202751	-1004.987700	-102.7	-1004.721428	-1004.781937	2.3	-999.237276	-4.217015	-1003.287628
conformer_15	-1004.946002	0.266458	0.203686	-1004.987558	-109.1	-1004.721100	-1004.780860	5.1	-999.236090	-4.215249	-1003.286197
conformer_18	-1004.943823	0.266405	0.203539	-1004.987487	-114.6	-1004.721082	-1004.780936	4.9	-999.232036	-4.216622	-1003.285771
conformer_4	-1004.950083	0.266369	0.203525	-1004.987484	-98.2	-1004.721115	-1004.780947	4.9	-999.239613	-4.216167	-1003.286643
conformer_1	-1004.949232	0.266508	0.204041	-1004.987387	-100.2	-1004.720879	-1004.780334	6.5	-999.239531	-4.216145	-1003.286778
conformer_14	-1004.947025	0.266472	0.202628	-1004.987309	-105.8	-1004.720837	-1004.781669	3.0	-999.239785	-4.213164	-1003.287594
conformer_8	-1004.947513	0.266328	0.202668	-1004.987144	-104.1	-1004.720816	-1004.781464	3.5	-999.237021	-4.216373	-1003.287345
conformer_32	-1004.943202	0.266535	0.203497	-1004.986725	-114.3	-1004.720190	-1004.780216	6.8	-999.233333	-4.215271	-1003.285618
conformer_21	-1004.948016	0.266339	0.203668	-1004.986718	-101.6	-1004.720379	-1004.780038	7.3	-999.238824	-4.216049	-1003.286896
conformer_3	-1004.951051	0.266417	0.203500	-1004.986546	-93.2	-1004.720129	-1004.780034	7.3	-999.241336	-4.215850	-1003.286170
conformer_19	-1004.947454	0.266454	0.202663	-1004.986328	-102.1	-1004.719874	-1004.780653	5.7	-999.240959	-4.212694	-1003.286852
conformer_5	-1004.946893	0.266260	0.202295	-1004.985930	-102.5	-1004.719670	-1004.780623	5.7	-999.238212	-4.214770	-1003.286711
**conformer_36**	**-1004.940798**	**0.266158**	**0.200088**	**-1004.985912**	**-118.4**	**-1004.719754**	**-1004.782812**	**0**	**-999.230738**	**-4.215435**	**-1003.288186**
conformer_10	-1004.946187	0.266168	0.202321	-1004.985903	-104.3	-1004.719735	-1004.780570	5.9	-999.237118	-4.215114	-1003.286616
conformer_17	-1004.945530	0.266366	0.203273	-1004.985891	-106.0	-1004.719525	-1004.779606	8.4	-999.235834	-4.215585	-1003.285495
conformer_20	-1004.946188	0.266193	0.202121	-1004.985001	-101.9	-1004.718808	-1004.779868	7.7	-999.237991	-4.214598	-1003.286269
conformer_7	-1004.946370	0.266478	0.202499	-1004.984342	-99.7	-1004.717864	-1004.778831	10.5	-999.239288	-4.213965	-1003.285714
conformer_42	-1004.938210	0.266229	0.202069	-1004.984123	-120.5	-1004.717894	-1004.779042	9.9	-999.229053	-4.214149	-1003.284034
conformer_30	-1004.940315	0.266581	0.202313	-1004.984067	-114.9	-1004.717486	-1004.778742	10.7	-999.232739	-4.213675	-1003.284842
conformer_44	-1004.940410	0.266323	0.203069	-1004.983838	-114.0	-1004.717515	-1004.777757	13.3	-999.231954	-4.214596	-1003.283896
conformer_46	-1004.939456	0.266365	0.203248	-1004.983721	-116.2	-1004.717356	-1004.777461	14.0	-999.228916	-4.216273	-1003.283194
conformer_41	-1004.937203	0.266146	0.201745	-1004.983695	-122.1	-1004.717549	-1004.778938	10.2	-999.226938	-4.215715	-1003.284388
conformer_6	-1004.946134	0.266443	0.202522	-1004.983641	-98.5	-1004.717198	-1004.778107	12.4	-999.240239	-4.213028	-1003.285240
conformer_26	-1004.941643	0.266433	0.202160	-1004.983564	-110.1	-1004.717131	-1004.778392	11.6	-999.234172	-4.214032	-1003.284953
conformer_34	-1004.939033	0.266580	0.202537	-1004.982977	-115.4	-1004.716397	-1004.777428	14.1	-999.231394	-4.213527	-1003.283316
conformer_13	-1004.944277	0.266276	0.201980	-1004.982959	-101.6	-1004.716683	-1004.777967	12.7	-999.238962	-4.212768	-1003.285420
conformer_24	-1004.944098	0.266240	0.201979	-1004.982452	-100.7	-1004.716212	-1004.777461	14.0	-999.239183	-4.212505	-1003.285052
conformer_2	-1004.946003	0.266574	0.202961	-1004.981818	-94.0	-1004.715244	-1004.775845	18.3	-999.239590	-4.213262	-1003.282693
conformer_40	-1004.938936	0.266391	0.202183	-1004.981550	-111.9	-1004.715159	-1004.776355	17.0	-999.231217	-4.213995	-1003.282631

conformer_33	-1004.940756	0.266350	0.201877	-1004.981187	-106.2	-1004.714837	-1004.776298	17.1	-999.234063	-4.213228	-1003.282832
conformer_48	-1004.938562	0.266185	0.201763	-1004.980946	-111.3	-1004.714761	-1004.776171	17.4	-999.232235	-4.212637	-1003.282481
conformer_50	-1004.938463	0.265951	0.201326	-1004.980805	-111.2	-1004.714854	-1004.776467	16.7	-999.229287	-4.214876	-1003.282167
conformer_29	-1004.941182	0.266096	0.201781	-1004.980126	-102.2	-1004.714030	-1004.775333	19.6	-999.232208	-4.214589	-1003.280949
conformer_25	-1004.941772	0.266368	0.201673	-1004.979966	-100.3	-1004.713598	-1004.775281	19.8	-999.235627	-4.212984	-1003.282119
conformer_38	-1004.936256	0.265909	0.200518	-1004.979772	-114.3	-1004.713863	-1004.776242	17.2	-999.229035	-4.213175	-1003.282196
conformer_37	-1004.934895	0.266121	0.200648	-1004.975167	-105.7	-1004.709046	-1004.771507	29.7	-999.230094	-4.211395	-1003.278100

[a]: Single-point calculation in aqueous phase with SMD model.

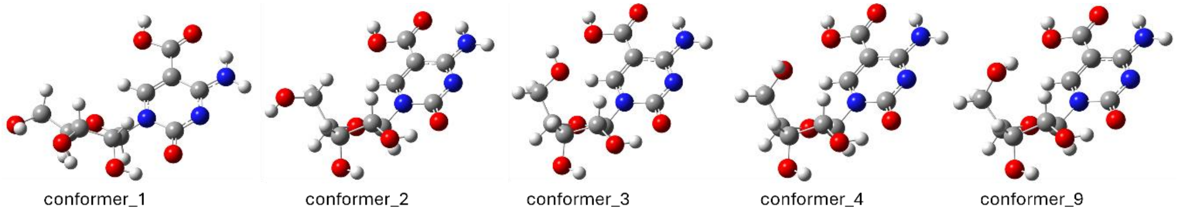

Figure S33. B3LYP-D3/def2-TZVPP optimized geometries of conformers for 5-carboxylcytidine (**1rb5caC**).

Table S31. Conformers of gas-phase optimized 5-carboxylcytidine (**1rb5caC**) at the B3LYP-D3/def2-TZVPP level of theory followed by aqueous phase single-point calculation. The columns display total energy without zero-point correction (E_{Tot}), thermal correction to enthalpy (δH), Gibbs free energy (δG), total energy without zero-point correction ($E_{Tot,W}$), Gibbs free energy ($G_{298,W}$) in water (W), total single-point energy (E_{CBS}) calculated at DLPNO-CCSD(T)/CBS level of theory, and their corresponding free energy $G_{CBS,W}$. $G_{298,W}$ and $G_{CBS,W}$ have been corrected to the standard state of 1 mol/L by addition of +7.908 kJ/mol. ΔG_{Solv} represents the Gibbs free energy of solvation. The data are arranged in the ascending numeric order of $E_{Tot,W}$. $\Delta G_{298,W}$ represents the respective energy difference to the lowest structure. Only conformers within the 24 kJ/mol (6 kcal/mol) energy window above the lowest in CREST are included in initial conformer sampling. Duplicates of the same structure are excluded. The overall optimum is marked bold.

	B3LYP-D3/def2-TZVPP			SMD(H_2O)/B3LYP-D3/def2-TZVPP[a]					DLPNO-CCSD(T)/CBS		
1rb5caC No.	E_{Tot} (Hartree)	δH (Hartree)	δG (Hartree)	$E_{Tot,W}$ (Hartree)	ΔG_{Solv} (kJ/mol)	$H_{298,W}$ (Hartree)	$G_{298,W}$ (Hartree)	$\Delta G_{298,W}$ (kJ/mol)	$E_{CBS,HF}$ (Hartree)	$E_{CBS,C}$ (Hartree)	$G_{CBS,W}$ (Hartree)
**conformer_9**	**-1080.236967**	**0.272731**	**0.206577**	**-1080.277181**	**-105.6**	**-1080.004450**	**-1080.067592**	**0**	**-1074.169345**	**-4.486648**	**-1078.486617**
conformer_25	-1080.234697	0.273021	0.208071	-1080.276977	-111.0	-1080.003956	-1080.065894	4.5	-1074.168779	-4.485109	-1078.485085
conformer_4	-1080.237163	0.272751	0.207350	-1080.276971	-104.5	-1080.004220	-1080.066609	2.6	-1074.170259	-4.486232	-1078.485937
conformer_17	-1080.231859	0.272940	0.207901	-1080.276813	-118.0	-1080.003873	-1080.065900	4.4	-1074.163817	-4.486336	-1078.484193
conformer_13	-1080.233811	0.272881	0.207645	-1080.276772	-112.8	-1080.003891	-1080.066115	3.9	-1074.167743	-4.484891	-1078.484938
conformer_7	-1080.235815	0.272782	0.207216	-1080.276698	-107.3	-1080.003916	-1080.066470	2.9	-1074.169279	-4.485737	-1078.485671
conformer_5	-1080.235417	0.272787	0.207565	-1080.276487	-107.8	-1080.003700	-1080.065910	4.4	-1074.169272	-4.486073	-1078.485838
conformer_26	-1080.234927	0.272763	0.207588	-1080.276382	-108.8	-1080.003619	-1080.065782	4.8	-1074.168338	-4.486707	-1078.485900
conformer_3	-1080.238632	0.272811	0.207565	-1080.276106	-98.4	-1080.003295	-1080.065529	5.4	-1074.172262	-4.485991	-1078.485150
conformer_27	-1080.232978	0.272855	0.206359	-1080.275996	-112.9	-1080.003141	-1080.066625	2.5	-1074.169452	-4.482987	-1078.486086
conformer_40	-1080.231255	0.273010	0.207743	-1080.275917	-117.3	-1080.002907	-1080.065162	6.4	-1074.165102	-4.484952	-1078.483961
conformer_2	-1080.234697	0.272782	0.206780	-1080.275342	-106.7	-1080.002560	-1080.065550	5.4	-1074.169600	-4.484657	-1078.485111
conformer_10	-1080.234002	0.272666	0.206493	-1080.275286	-108.4	-1080.002620	-1080.065781	4.8	-1074.168485	-4.485012	-1078.485275
conformer_62	-1080.225927	0.272570	0.205750	-1080.275172	-129.3	-1080.002602	-1080.066410	3.1	-1074.159285	-4.485578	-1078.485346
conformer_16	-1080.233487	0.272882	0.207494	-1080.275009	-109.0	-1080.002127	-1080.064503	8.1	-1074.167524	-4.485272	-1078.483812
conformer_47	-1080.229319	0.272952	0.206899	-1080.274913	-119.7	-1080.001961	-1080.065002	6.8	-1074.162617	-4.485075	-1078.483374
conformer_39	-1080.230911	0.273006	0.209483	-1080.274529	-114.5	-1080.001523	-1080.062034	14.6	-1074.159588	-4.491455	-1078.482165
conformer_42	-1080.227814	0.272916	0.207610	-1080.274347	-122.2	-1080.001431	-1080.063725	10.2	-1074.159785	-4.486132	-1078.481827
conformer_19	-1080.233969	0.272685	0.206371	-1080.274315	-105.9	-1080.001630	-1080.064932	7.0	-1074.169407	-4.484450	-1078.484820
conformer_38	-1080.229624	0.272837	0.207275	-1080.274241	-117.1	-1080.001404	-1080.063954	9.6	-1074.163551	-4.484772	-1078.482653
conformer_28	-1080.232449	0.272696	0.207091	-1080.274219	-109.7	-1080.001523	-1080.064116	9.1	-1074.165306	-4.486291	-1078.483264
conformer_30	-1080.231395	0.272703	0.206780	-1080.274069	-112.0	-1080.001366	-1080.064277	8.7	-1074.165055	-4.485286	-1078.483223
conformer_6	-1080.234569	0.272924	0.206639	-1080.273685	-102.7	-1080.000761	-1080.064034	9.3	-1074.171254	-4.483554	-1078.484274
conformer_64	-1080.222884	0.272453	0.204287	-1080.273636	-133.3	-1080.001183	-1080.066337	3.3	-1074.157567	-4.483975	-1078.484995
conformer_60	-1080.231705	0.272886	0.208485	-1080.273611	-110.0	-1080.000725	-1080.062114	14.4	-1074.162575	-4.488280	-1078.481265
conformer_20	-1080.234969	0.272846	0.207138	-1080.273533	-101.2	-1080.000687	-1080.063383	11.1	-1074.167975	-4.486028	-1078.482416
conformer_33	-1080.229935	0.272687	0.206950	-1080.273253	-113.7	-1080.000566	-1080.063291	11.3	-1074.163488	-4.486119	-1078.482963
conformer_56	-1080.227505	0.272957	0.207761	-1080.273157	-119.9	-1080.000200	-1080.062384	13.7	-1074.160702	-4.485916	-1078.481497
conformer_59	-1080.228274	0.272847	0.207315	-1080.273139	-117.8	-1080.000292	-1080.062812	12.5	-1074.163593	-4.484229	-1078.482359
conformer_41	-1080.228201	0.273056	0.206432	-1080.273105	-117.9	-1080.000049	-1080.063661	10.3	-1074.164302	-4.483412	-1078.483174

conformer_51	-1080.225255	0.272651	0.205843	-1080.273056	-125.5	-1080.000405	-1080.064201	8.9	-1074.158489	-4.485532	-1078.482966
conformer_52	-1080.225255	0.272651	0.205843	-1080.273056	-125.5	-1080.000405	-1080.064201	8.9	-1074.158489	-4.485531	-1078.482966
conformer_15	-1080.232711	0.272932	0.205993	-1080.272933	-105.6	-1080.000001	-1080.063928	9.6	-1074.170708	-4.482302	-1078.484227
conformer_8	-1080.234011	0.272902	0.206517	-1080.272821	-101.9	-1079.999919	-1080.063292	11.3	-1074.171838	-4.482777	-1078.483896
conformer_22	-1080.230326	0.272709	0.206264	-1080.272736	-111.3	-1080.000027	-1080.063460	10.8	-1074.165201	-4.484496	-1078.482830
conformer_36	-1080.229487	0.272964	0.206416	-1080.272648	-113.3	-1079.999684	-1080.063220	11.5	-1074.165693	-4.483752	-1078.483178
conformer_32	-1080.229425	0.272576	0.205894	-1080.272587	-113.3	-1080.000011	-1080.063681	10.3	-1074.163833	-4.484899	-1078.482989
conformer_45	-1080.229113	0.272822	0.207041	-1080.272410	-113.7	-1079.999588	-1080.062357	13.7	-1074.163114	-4.485114	-1078.481472
conformer_14	-1080.231697	0.272704	0.206052	-1080.272172	-106.3	-1079.999468	-1080.063108	11.8	-1074.170002	-4.482519	-1078.483933
conformer_49	-1080.226886	0.273069	0.206586	-1080.271998	-118.4	-1079.998929	-1080.062400	13.6	-1074.163117	-4.483031	-1078.481662
conformer_46	-1080.229755	0.272621	0.205879	-1080.271800	-110.4	-1079.999179	-1080.062909	12.3	-1074.165096	-4.484348	-1078.482597
conformer_31	-1080.231497	0.272679	0.206098	-1080.271613	-105.3	-1079.998934	-1080.062503	13.4	-1074.170248	-4.482231	-1078.483484
conformer_24	-1080.230334	0.272893	0.206292	-1080.271052	-106.9	-1079.998159	-1080.061748	15.3	-1074.167115	-4.483211	-1078.481741
conformer_1	-1080.233658	0.272961	0.206852	-1080.270946	-97.9	-1079.997985	-1080.061082	17.1	-1074.170966	-4.483051	-1078.481442
conformer_54	-1080.226771	0.272865	0.206445	-1080.270569	-115.0	-1079.997704	-1080.061112	17.0	-1074.162871	-4.483551	-1078.480764
conformer_29	-1080.229552	0.272841	0.206011	-1080.270147	-106.6	-1079.997306	-1080.061124	17.0	-1074.167371	-4.482563	-1078.481506
conformer_50	-1080.228440	0.272800	0.205896	-1080.270025	-109.2	-1079.997225	-1080.061117	17.0	-1074.165642	-4.482819	-1078.481138
conformer_61	-1080.226204	0.272711	0.206045	-1080.269910	-114.8	-1079.997199	-1080.060853	17.7	-1074.163771	-4.482159	-1078.480579
conformer_55	-1080.226712	0.272639	0.205552	-1080.269381	-112.0	-1079.996742	-1080.060817	17.8	-1074.164809	-4.482526	-1078.481441
conformer_37	-1080.229420	0.272774	0.205462	-1080.268732	-103.2	-1079.995958	-1080.060258	19.3	-1074.167147	-4.482604	-1078.480589
conformer_21	-1080.229002	0.272908	0.206396	-1080.268243	-103.0	-1079.995335	-1080.058835	23.0	-1074.166211	-4.482952	-1078.478996

[a]: Single-point calculation in aqueous phase with SMD model.

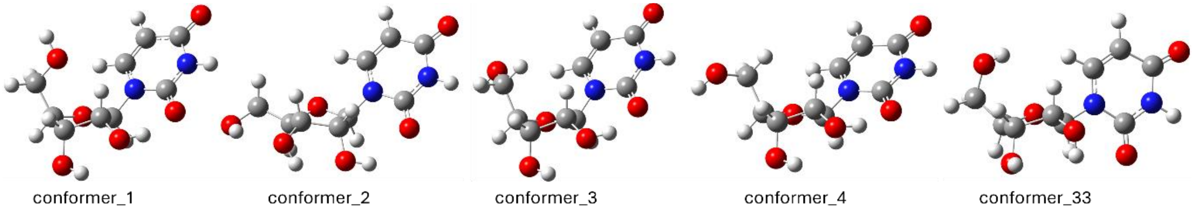

Figure S34. B3LYP-D3/def2-TZVPP optimized geometries of conformers for uridine (**1rbU**).

Table S32. Conformers of gas-phase optimized uridine (**1rbU**) at the B3LYP-D3/def2-TZVPP level of theory followed by aqueous phase single-point calculation. The columns display total energy without zero-point correction (E_{Tot}), thermal correction to enthalpy (δH), Gibbs free energy (δG), total energy without zero-point correction ($E_{Tot,W}$), Gibbs free energy ($G_{298,W}$) in water (W), total single-point energy (E_{CBS}) calculated at DLPNO-CCSD(T)/CBS level of theory, and their corresponding free energy $G_{CBS,W}$. $G_{298,W}$ and $G_{CBS,W}$ have been corrected to the standard state of 1 mol/L by addition of +7.908 kJ/mol. ΔG_{Solv} represents the Gibbs free energy of solvation. The data are arranged in the ascending numeric order of $E_{Tot,W}$. $\Delta G_{298,W}$ represents the respective energy difference to the lowest structure. Only conformers within the 24 kJ/mol (6 kcal/mol) energy window above the lowest in CREST are included in initial conformer sampling. Duplicates of the same structure are excluded. The overall optimum is marked bold.

	B3LYP-D3/def2-TZVPP			SMD(H2O)/B3LYP-D3/def2-TZVPP[a]					DLPNO-CCSD(T)/CBS		
1rbU No.	E_{Tot} (Hartree)	δH (Hartree)	δG (Hartree)	$E_{Tot,W}$ (Hartree)	ΔG_{Solv} (kJ/mol)	$H_{298,W}$ (Hartree)	$G_{298,W}$ (Hartree)	$\Delta G_{298,W}$ (kJ/mol)	$E_{CBS,HF}$ (Hartree)	$E_{CBS,C}$ (Hartree)	$G_{CBS,W}$ (Hartree)
conformer_26	-911.455617	0.243099	0.185156	-911.490299	-91.1	-911.247200	-911.302131	4.2	-906.310639	-3.805105	-909.962258
conformer_5	-911.454938	0.243014	0.184899	-911.490193	-92.6	-911.247179	-911.302282	3.8	-906.309889	-3.804903	-909.962135
conformer_10	-911.452744	0.243000	0.184896	-911.490152	-98.2	-911.247152	-911.302244	3.9	-906.305864	-3.806212	-909.961576
conformer_32	-911.451884	0.242992	0.184237	-911.489004	-97.5	-911.246012	-911.301755	5.2	-906.306904	-3.804647	-909.961422
**conformer_33**	**-911.449178**	**0.243039**	**0.182266**	**-911.488998**	**-104.5**	**-911.245959**	**-911.303720**	**0**	**-906.307035**	**-3.802388**	**-909.963965**
conformer_19	-911.453419	0.242897	0.183629	-911.488595	-92.4	-911.245698	-911.301954	4.6	-906.310414	-3.802802	-909.961751
conformer_52	-911.446833	0.242791	0.182355	-911.488425	-109.2	-911.245634	-911.303058	1.7	-906.303116	-3.803782	-909.963123
conformer_20	-911.452153	0.242904	0.182425	-911.488335	-95.0	-911.245431	-911.302898	2.2	-906.309089	-3.802877	-909.962711
conformer_12	-911.454067	0.242899	0.184298	-911.488153	-89.5	-911.245254	-911.300843	7.6	-906.309031	-3.805074	-909.960881
conformer_7	-911.455413	0.242791	0.183912	-911.487979	-85.5	-911.245188	-911.301055	7.0	-906.308729	-3.806549	-909.960920
conformer_41	-911.446889	0.242729	0.183019	-911.487973	-107.9	-911.245244	-911.301942	4.7	-906.302148	-3.803999	-909.961200
conformer_49	-911.445703	0.242719	0.182352	-911.487923	-110.8	-911.245204	-911.302559	3.1	-906.301873	-3.803664	-909.962393
conformer_77	-911.447275	0.242681	0.182849	-911.487808	-106.4	-911.245127	-911.301947	4.7	-906.304457	-3.802524	-909.961653
conformer_70	-911.449695	0.242851	0.182826	-911.487786	-100.0	-911.244935	-911.301948	4.7	-906.305190	-3.804430	-909.961873
conformer_3	-911.454347	0.242852	0.184546	-911.487702	-87.6	-911.244850	-911.300144	9.4	-906.309102	-3.805463	-909.960362
conformer_6	-911.455244	0.242784	0.183806	-911.487587	-84.9	-911.244803	-911.300769	7.8	-906.309488	-3.805466	-909.960480
conformer_22	-911.453859	0.242894	0.183607	-911.487554	-88.5	-911.244660	-911.300935	7.3	-906.311729	-3.802290	-909.961095
conformer_54	-911.448020	0.242732	0.183105	-911.487525	-103.7	-911.244793	-911.301408	6.1	-906.302466	-3.804908	-909.960762
conformer_31	-911.448534	0.242826	0.183410	-911.487517	-102.3	-911.244691	-911.301095	6.9	-906.304684	-3.803491	-909.960735
conformer_71	-911.446289	0.242793	0.181766	-911.487431	-108.0	-911.244638	-911.302653	2.8	-906.304669	-3.801865	-909.962897
conformer_83	-911.449577	0.242998	0.183147	-911.487397	-99.3	-911.244399	-911.301238	6.5	-906.307433	-3.802565	-909.961659
conformer_63	-911.446664	0.242656	0.182060	-911.487384	-106.9	-911.244728	-911.302312	3.7	-906.305005	-3.802048	-909.962701
conformer_13	-911.453456	0.242766	0.183602	-911.487283	-88.8	-911.244517	-911.300669	8.0	-906.308164	-3.805201	-909.960578
conformer_25	-911.453479	0.242721	0.184236	-911.487198	-88.5	-911.244477	-911.299950	9.9	-906.308752	-3.805445	-909.960668
conformer_55	-911.449205	0.242744	0.183001	-911.487197	-99.7	-911.244453	-911.301184	6.7	-906.307341	-3.801970	-909.961291
conformer_48	-911.444037	0.242628	0.181993	-911.487023	-112.9	-911.244395	-911.302018	4.5	-906.301332	-3.802569	-909.961883
conformer_1	-911.456743	0.242872	0.184196	-911.486969	-79.4	-911.244097	-911.299761	10.4	-906.311736	-3.805272	-909.960027
conformer_40	-911.447629	0.242849	0.182864	-911.486960	-103.3	-911.244111	-911.301084	6.9	-906.301735	-3.805279	-909.960468
conformer_85	-911.442958	0.242449	0.181253	-911.486875	-115.3	-911.244426	-911.302610	2.9	-906.300203	-3.802603	-909.962458
conformer_86	-911.445697	0.242801	0.182703	-911.486812	-107.9	-911.244011	-911.301097	6.9	-906.303631	-3.802445	-909.961476

conformer_38	-911.449125	0.242721	0.183741	-911.486701	-98.7	-911.243980	-911.299948	9.9	-906.305609	-3.804006	-909.960439
conformer_42	-911.448087	0.242801	0.184033	-911.486518	-100.9	-911.243717	-911.299473	11.2	-906.302505	-3.805632	-909.959522
conformer_16	-911.452099	0.242685	0.183213	-911.486458	-90.2	-911.243773	-911.300233	9.2	-906.307642	-3.804531	-909.960308
conformer_24	-911.450681	0.242854	0.182926	-911.486449	-93.9	-911.243595	-911.300511	8.4	-906.308295	-3.803167	-909.961292
conformer_4	-911.452986	0.242804	0.183402	-911.486442	-87.8	-911.243638	-911.300028	9.7	-906.308968	-3.804212	-909.960222
conformer_57	-911.448189	0.242641	0.182479	-911.486423	-100.4	-911.243782	-911.300932	7.3	-906.303715	-3.804243	-909.960701
conformer_64	-911.445848	0.242524	0.181252	-911.486227	-106.0	-911.243703	-911.301963	4.6	-906.305381	-3.800954	-909.962450
conformer_65	-911.444861	0.242512	0.181192	-911.485947	-107.9	-911.243435	-911.301743	5.2	-906.302039	-3.802995	-909.961916
conformer_73	-911.447066	0.242617	0.182403	-911.485874	-101.9	-911.243257	-911.300459	8.6	-906.304863	-3.802902	-909.961158
conformer_30	-911.448532	0.243021	0.183469	-911.485851	-98.0	-911.242830	-911.299370	11.4	-906.305735	-3.802960	-909.959533
conformer_90	-911.443899	0.242515	0.181540	-911.485815	-110.0	-911.243300	-911.301263	6.5	-906.301829	-3.802216	-909.961408
conformer_89	-911.443305	0.242600	0.182163	-911.485667	-111.2	-911.243067	-911.300492	8.5	-906.298344	-3.804871	-909.960403
conformer_50	-911.445454	0.242790	0.182973	-911.485505	-105.2	-911.242715	-911.299520	11.0	-906.300083	-3.804901	-909.959050
conformer_21	-911.452403	0.242724	0.183143	-911.485358	-86.5	-911.242634	-911.299203	11.9	-906.308940	-3.803973	-909.959712
conformer_80	-911.446253	0.242510	0.181115	-911.485270	-102.4	-911.242760	-911.301143	6.8	-906.306446	-3.800501	-909.961836
conformer_47	-911.447080	0.242739	0.183018	-911.485198	-100.1	-911.242459	-911.299168	12.0	-906.304966	-3.802583	-909.959637
conformer_8	-911.452832	0.242923	0.183175	-911.485051	-84.6	-911.242128	-911.298864	12.8	-906.310401	-3.803240	-909.959673
conformer_9	-911.452476	0.242924	0.183396	-911.484604	-84.4	-911.241680	-911.298196	14.5	-906.311183	-3.802274	-909.959177
conformer_69	-911.447760	0.242718	0.182272	-911.484550	-96.6	-911.241832	-911.299266	11.7	-906.305174	-3.802971	-909.959650
conformer_37	-911.448310	0.242908	0.183545	-911.484378	-94.7	-911.241470	-911.297821	15.5	-906.305209	-3.803442	-909.958162
conformer_35	-911.445187	0.242959	0.183142	-911.484238	-102.5	-911.241279	-911.298084	14.8	-906.301502	-3.803557	-909.957956
conformer_39	-911.448021	0.242814	0.183154	-911.484197	-95.0	-911.241383	-911.298031	14.9	-906.306668	-3.801963	-909.958641
conformer_58	-911.444660	0.242769	0.183402	-911.484165	-103.7	-911.241396	-911.297751	15.7	-906.300389	-3.804238	-909.957718
conformer_27	-911.450469	0.242823	0.183182	-911.484030	-88.1	-911.241207	-911.297836	15.5	-906.308367	-3.802858	-909.958591
conformer_17	-911.450286	0.242732	0.182858	-911.483837	-88.1	-911.241105	-911.297967	15.1	-906.309398	-3.802141	-909.959220
conformer_56	-911.445437	0.242715	0.182571	-911.483695	-100.4	-911.240980	-911.298112	14.7	-906.301989	-3.803668	-909.958333
conformer_72	-911.448176	0.242696	0.182161	-911.483513	-92.8	-911.240817	-911.298340	14.1	-906.306470	-3.802519	-909.959153
conformer_36	-911.450263	0.242717	0.182905	-911.483285	-86.7	-911.240568	-911.297368	16.7	-906.309800	-3.801856	-909.958761
conformer_23	-911.451279	0.242887	0.183170	-911.483101	-83.5	-911.240214	-911.296919	17.9	-906.309871	-3.802518	-909.958028
conformer_2	-911.452255	0.243051	0.183891	-911.482918	-80.5	-911.239867	-911.296015	20.2	-906.310261	-3.802623	-909.956644
conformer_92	-911.444352	0.242904	0.184266	-911.482755	-100.8	-911.239851	-911.295477	21.6	-906.294879	-3.809252	-909.955256
conformer_45	-911.444673	0.242899	0.184571	-911.482467	-99.2	-911.239568	-911.294884	23.2	-906.295687	-3.808378	-909.954276
conformer_78	-911.445803	0.242716	0.183122	-911.481269	-93.1	-911.238553	-911.295135	22.5	-906.300994	-3.805724	-909.956050
conformer_68	-911.443943	0.242821	0.184235	-911.480040	-94.8	-911.237219	-911.292793	28.7	-906.295929	-3.808281	-909.953060

[a]: Single-point calculation in aqueous phase with SMD model.

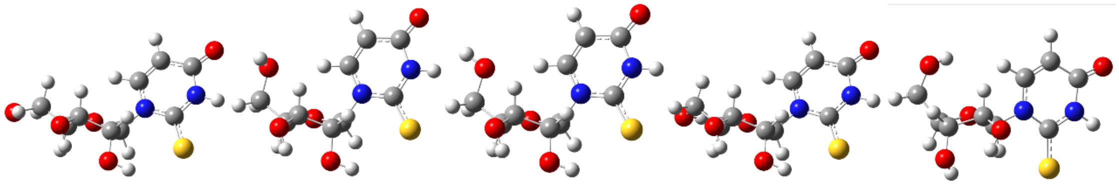

Figure S35. B3LYP-D3/def2-TZVPP optimized geometries of conformers for 2-thiouridine (**1rb2thU**).

Table S33. Conformers of gas-phase optimized 2-thiouridine (**1rb2thU**) at the B3LYP-D3/def2-TZVPP level of theory followed by aqueous phase single-point calculation. The columns display total energy without zero-point correction (E_{Tot}), thermal correction to enthalpy (δH), Gibbs free energy (δG), total energy without zero-point correction ($E_{Tot,W}$), Gibbs free energy ($G_{298,W}$) in water (W), total single-point energy (E_{CBS}) calculated at DLPNO-CCSD(T)/CBS level of theory, and their corresponding free energy $G_{CBS,W}$. $G_{298,W}$ and $G_{CBS,W}$ have been corrected to the standard state of 1 mol/L by addition of +7.908 kJ/mol. ΔG_{Solv} represents the Gibbs free energy of solvation. The data are arranged in the ascending numeric order of $E_{Tot,W}$. $\Delta G_{298,W}$ represents the respective energy difference to the lowest structure. Only conformers within the 24 kJ/mol (6 kcal/mol) energy window above the lowest in CREST are included in initial conformer sampling. Duplicates of the same structure are excluded. The overall optimum is marked bold.

	B3LYP-D3/def2-TZVPP			SMD(H2O)/B3LYP-D3/def2-TZVPP[a]					DLPNO-CCSD(T)/CBS		
**1rb2thU No.**	**E_{Tot}** (Hartree)	**δH** (Hartree)	**δG** (Hartree)	**$E_{Tot,W}$** (Hartree)	**ΔG_{Solv}** (kJ/mol)	**$H_{298,W}$** (Hartree)	**$G_{298,W}$** (Hartree)	**$\Delta G_{298,W}$** (kJ/mol)	**$E_{CBS,HF}$** (Hartree)	**$E_{CBS,C}$** (Hartree)	**$G_{CBS,W}$** (Hartree)
conformer_2	-1234.402284	0.241108	0.181132	-1234.433897	-83.0	-1234.192789	-1234.249753	2.7	-1228.940961	-3.758207	-1232.546637
conformer_3	-1234.402795	0.241136	0.181324	-1234.432967	-79.2	-1234.191831	-1234.248631	5.7	-1228.942261	-3.757781	-1232.545878
conformer_59	-1234.395512	0.240776	0.179573	-1234.432817	-97.9	-1234.192041	-1234.250232	1.5	-1228.933165	-3.759227	-1232.547112
conformer_68	-1234.396743	0.240699	0.180481	-1234.432477	-93.8	-1234.191778	-1234.248984	4.7	-1228.935246	-3.759061	-1232.546548
conformer_20	-1234.392587	0.240710	0.179910	-1234.432377	-104.5	-1234.191667	-1234.249455	3.5	-1228.930086	-3.758571	-1232.545526
conformer_47	-1234.392495	0.240676	0.179914	-1234.432372	-104.7	-1234.191696	-1234.249446	3.5	-1228.929182	-3.760177	-1232.546310
**conformer_43**	**-1234.391895**	**0.240652**	**0.178559**	**-1234.432357**	**-106.2**	**-1234.191705**	**-1234.250786**	**0**	**-1228.930364**	**-3.758424**	**-1232.547678**
conformer_8	-1234.401319	0.240535	0.181024	-1234.432273	-81.3	-1234.191738	-1234.248237	6.7	-1228.936807	-3.760818	-1232.544543
conformer_11	-1234.401319	0.240535	0.181025	-1234.432273	-81.3	-1234.191738	-1234.248236	6.7	-1228.936807	-3.760818	-1232.544542
conformer_41	-1234.390937	0.240681	0.179144	-1234.432154	-108.2	-1234.191473	-1234.249998	2.1	-1228.929379	-3.758183	-1232.546623
conformer_17	-1234.398750	0.240421	0.179922	-1234.432143	-87.7	-1234.191722	-1234.249209	4.1	-1228.934017	-3.761493	-1232.545968
conformer_4	-1234.400056	0.240853	0.180182	-1234.432139	-84.2	-1234.191286	-1234.248945	4.8	-1228.939932	-3.757853	-1232.546674
conformer_52	-1234.392988	0.240622	0.179550	-1234.432135	-102.8	-1234.191513	-1234.249573	3.2	-1228.932341	-3.757170	-1232.546096
conformer_6	-1234.399917	0.240586	0.181302	-1234.431978	-84.2	-1234.191392	-1234.247664	8.2	-1228.934616	-3.762273	-1232.544637
conformer_15	-1234.394190	0.240797	0.180237	-1234.431949	-99.1	-1234.191152	-1234.248700	5.5	-1228.932587	-3.758143	-1232.545240
conformer_19	-1234.398793	0.240375	0.179636	-1234.431815	-86.7	-1234.191440	-1234.249167	4.2	-1228.934471	-3.761043	-1232.545889
conformer_48	-1234.391904	0.240642	0.178967	-1234.431712	-104.5	-1234.191070	-1234.249733	2.8	-1228.932184	-3.756858	-1232.546871
conformer_74	-1234.392069	0.240398	0.178883	-1234.431698	-104.0	-1234.191300	-1234.249803	2.6	-1228.930993	-3.758638	-1232.547365
conformer_36	-1234.394845	0.240690	0.179690	-1234.431576	-96.4	-1234.190886	-1234.248874	5.0	-1228.935184	-3.756612	-1232.545826
conformer_5	-1234.402529	0.240583	0.181058	-1234.431514	-76.1	-1234.190931	-1234.247444	8.8	-1228.939018	-3.760323	-1232.544255
conformer_9	-1234.397681	0.240560	0.180199	-1234.431401	-88.5	-1234.190841	-1234.248190	6.8	-1228.934499	-3.760013	-1232.545021
conformer_37	-1234.398922	0.240563	0.181425	-1234.431390	-85.2	-1234.190827	-1234.246953	10.1	-1228.933914	-3.762458	-1232.544404
conformer_34	-1234.389381	0.240578	0.178531	-1234.431343	-110.2	-1234.190765	-1234.249800	2.6	-1228.928704	-3.757322	-1232.546444
conformer_65	-1234.389663	0.240416	0.178443	-1234.431187	-109.0	-1234.190771	-1234.249732	2.8	-1228.926948	-3.759830	-1232.546846
conformer_13	-1234.397683	0.240690	0.179786	-1234.431175	-87.9	-1234.190485	-1234.248377	6.3	-1228.938001	-3.757573	-1232.546269
conformer_61	-1234.388339	0.240418	0.177871	-1234.431164	-112.4	-1234.190746	-1234.250281	1.3	-1228.927605	-3.757279	-1232.546827
conformer_56	-1234.394801	0.240674	0.180164	-1234.431160	-95.5	-1234.190486	-1234.247984	7.4	-1228.933119	-3.758982	-1232.545283
conformer_66	-1234.389670	0.240396	0.177567	-1234.430658	-107.6	-1234.190262	-1234.250079	1.9	-1228.930693	-3.756010	-1232.547113
conformer_31	-1234.397589	0.240645	0.179752	-1234.430519	-86.5	-1234.189874	-1234.247755	8.0	-1228.938304	-3.757384	-1232.545854
conformer_42	-1234.391131	0.240501	0.177970	-1234.430490	-103.3	-1234.189989	-1234.249508	3.4	-1228.932733	-3.755671	-1232.546781

conformer_1	-1234.400024	0.240958	0.180701	-1234.430319	-79.5	-1234.189361	-1234.246606	11.0	-1228.939072	-3.758349	-1232.544003
conformer_44	-1234.396947	0.240459	0.179895	-1234.430282	-87.5	-1234.189823	-1234.247375	9.0	-1228.934242	-3.759872	-1232.544542
conformer_62	-1234.389224	0.240489	0.178183	-1234.430235	-107.7	-1234.189746	-1234.249040	4.6	-1228.929120	-3.757045	-1232.545981
conformer_29	-1234.394090	0.240960	0.181078	-1234.430229	-94.9	-1234.189269	-1234.246139	12.2	-1228.930158	-3.760868	-1232.543076
conformer_55	-1234.395192	0.240634	0.179998	-1234.430091	-91.6	-1234.189457	-1234.247081	9.7	-1228.934282	-3.758618	-1232.544790
conformer_57	-1234.391541	0.240526	0.178128	-1234.429602	-99.9	-1234.189076	-1234.248462	6.1	-1228.933740	-3.755242	-1232.545903
conformer_50	-1234.387918	0.240627	0.179070	-1234.429198	-108.4	-1234.188571	-1234.247116	9.6	-1228.924807	-3.759239	-1232.543244
conformer_21	-1234.393952	0.240749	0.180663	-1234.429042	-92.1	-1234.188293	-1234.245367	14.2	-1228.929067	-3.761629	-1232.542110
conformer_51	-1234.394177	0.240589	0.178865	-1234.429012	-91.5	-1234.188423	-1234.247135	9.6	-1228.935034	-3.756814	-1232.544806
conformer_71	-1234.395008	0.240851	0.181097	-1234.428996	-89.2	-1234.188145	-1234.244887	15.5	-1228.930601	-3.761597	-1232.542078
conformer_14	-1234.395632	0.240846	0.180956	-1234.428973	-87.5	-1234.188127	-1234.245005	15.2	-1228.931653	-3.761296	-1232.542323
conformer_30	-1234.391738	0.241008	0.180258	-1234.428637	-96.9	-1234.187629	-1234.245367	14.2	-1228.930043	-3.759102	-1232.542774
conformer_23	-1234.392126	0.240875	0.180231	-1234.427410	-92.6	-1234.186535	-1234.244167	17.4	-1228.930066	-3.759766	-1232.541874
conformer_38	-1234.390313	0.240997	0.180318	-1234.426760	-95.7	-1234.185763	-1234.243430	19.3	-1228.928060	-3.759286	-1232.540464
conformer_72	-1234.388263	0.240587	0.179577	-1234.426737	-101.0	-1234.186150	-1234.244148	17.4	-1228.926331	-3.759573	-1232.541789
conformer_49	-1234.387206	0.240804	0.179583	-1234.425492	-100.5	-1234.184688	-1234.242897	20.7	-1228.924484	-3.759718	-1232.539892
conformer_46	-1234.389257	0.240809	0.179984	-1234.425103	-94.1	-1234.184294	-1234.242107	22.8	-1228.926540	-3.760052	-1232.539443
conformer_64	-1234.388093	0.240664	0.179405	-1234.425102	-97.2	-1234.184438	-1234.242685	21.3	-1228.926611	-3.758857	-1232.540061
conformer_24	-1234.391759	0.240766	0.179936	-1234.424501	-86.0	-1234.183735	-1234.241553	24.2	-1228.930280	-3.759226	-1232.539300
conformer_16	-1234.392701	0.240808	0.180054	-1234.423540	-81.0	-1234.182732	-1234.240474	27.1	-1228.931896	-3.758932	-1232.538602

[a]: Single-point calculation in aqueous phase with SMD model.

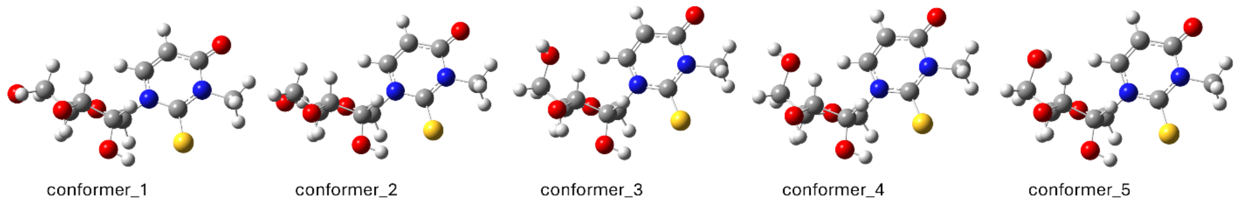

Figure S36. B3LYP-D3/def2-TZVPP optimized geometries of conformers for 2-thio-3-methyluridine (**1rb2th3mU**).

Table S34. Conformers of gas-phase optimized 2-thio-3-methyluridine (**1rb2th3mU**) at the B3LYP-D3/def2-TZVPP level of theory followed by aqueous phase single-point calculation. The columns display total energy without zero-point correction (E_{Tot}), thermal correction to enthalpy (δH), Gibbs free energy (δG), total energy without zero-point correction ($E_{Tot,W}$), Gibbs free energy ($G_{298,W}$) in water (W), total single-point energy (E_{CBS}) calculated at DLPNO-CCSD(T)/CBS level of theory, and their corresponding free energy $G_{CBS,W}$. $G_{298,W}$ and $G_{CBS,W}$ have been corrected to the standard state of 1 mol/L by addition of +7.908 kJ/mol. ΔG_{Solv} represents the Gibbs free energy of solvation. The data are arranged in the ascending numeric order of $E_{Tot,W}$. $\Delta G_{298,W}$ represents the respective energy difference to the lowest structure. Only conformers within the 24 kJ/mol (6 kcal/mol) energy window above the lowest in CREST are included in initial conformer sampling. Duplicates of the same structure are excluded. The overall optimum is marked bold.

	B3LYP-D3/def2-TZVPP			SMD(H_2O)/B3LYP-D3/def2-TZVPP[a]					DLPNO-CCSD(T)/CBS		
1rb2th3mU No.	E_{Tot} (Hartree)	δH (Hartree)	δG (Hartree)	$E_{Tot,W}$ (Hartree)	ΔG_{Solv} (kJ/mol)	$H_{298,W}$ (Hartree)	$G_{298,W}$ (Hartree)	$\Delta G_{298,W}$ (kJ/mol)	$E_{CBS,HF}$ (Hartree)	$E_{CBS,C}$ (Hartree)	$G_{CBS,W}$ (Hartree)
conformer_3	-1273.725581	0.270260	0.207133	-1273.753045	-72.1	-1273.482785	-1273.542900	1.4	-1267.977915	-3.972540	-1271.767774
**conformer_5**	**-1273.724437**	**0.270179**	**0.206255**	**-1273.752706**	**-74.2**	**-1273.482527**	**-1273.543439**	**0**	**-1267.976735**	**-3.972601**	**-1271.768337**
conformer_4	-1273.726031	0.270231	0.207156	-1273.752011	-68.2	-1273.481780	-1273.541843	4.2	-1267.979205	-3.972188	-1271.767206
conformer_62	-1273.718502	0.269950	0.205711	-1273.751768	-87.3	-1273.481818	-1273.543045	1.0	-1267.969726	-3.973795	-1271.768064
conformer_16	-1273.722234	0.269710	0.206641	-1273.751642	-77.2	-1273.481932	-1273.541989	3.8	-1267.971165	-3.975730	-1271.766651
conformer_10	-1273.724588	0.269769	0.207139	-1273.751553	-70.8	-1273.481784	-1273.541402	5.3	-1267.973508	-3.975274	-1271.765597
conformer_2	-1273.723634	0.270142	0.206616	-1273.751410	-72.9	-1273.481268	-1273.541782	4.4	-1267.977108	-3.972288	-1271.767543
conformer_41	-1273.715926	0.269935	0.206133	-1273.751333	-93.0	-1273.481398	-1273.542188	3.3	-1267.966254	-3.974536	-1271.767051
conformer_22	-1273.722059	0.269614	0.205882	-1273.751036	-76.1	-1273.481422	-1273.542142	3.4	-1267.971225	-3.975471	-1271.766780
conformer_7	-1273.722848	0.269752	0.207235	-1273.750938	-73.7	-1273.481186	-1273.540691	7.2	-1267.971111	-3.976704	-1271.765658
conformer_45	-1273.714590	0.269861	0.204638	-1273.750886	-95.3	-1273.481025	-1273.543236	0.5	-1267.966511	-3.972902	-1271.768059
conformer_24	-1273.715065	0.269912	0.205959	-1273.750797	-93.8	-1273.480885	-1273.541826	4.2	-1267.965972	-3.973013	-1271.765747
conformer_6	-1273.725744	0.269875	0.207357	-1273.750694	-65.5	-1273.480819	-1273.540325	8.2	-1267.975684	-3.974777	-1271.765043
conformer_40	-1273.713742	0.269910	0.205246	-1273.750594	-96.8	-1273.480684	-1273.542336	2.9	-1267.965470	-3.972832	-1271.766896
conformer_15	-1273.721115	0.269933	0.205973	-1273.750501	-77.2	-1273.480568	-1273.541516	5.0	-1267.975014	-3.972042	-1271.767456
conformer_60	-1273.715354	0.269799	0.205580	-1273.750493	-92.3	-1273.480694	-1273.541901	4.0	-1267.968116	-3.971736	-1271.766399
conformer_11	-1273.720924	0.269708	0.206017	-1273.750457	-77.5	-1273.480749	-1273.541428	5.3	-1267.971296	-3.974438	-1271.766238
conformer_19	-1273.716636	0.269993	0.206242	-1273.750325	-88.5	-1273.480332	-1273.541071	6.2	-1267.968473	-3.972610	-1271.765518
conformer_36	-1273.721803	0.269651	0.207008	-1273.750195	-74.5	-1273.480544	-1273.540175	8.6	-1267.970093	-3.977304	-1271.765770
conformer_49	-1273.714561	0.269854	0.204942	-1273.750118	-93.4	-1273.480264	-1273.542164	3.3	-1267.968208	-3.971394	-1271.767205
conformer_51	-1273.717865	0.269842	0.206145	-1273.750076	-84.6	-1273.480234	-1273.540919	6.6	-1267.969799	-3.973550	-1271.766404
conformer_38	-1273.717156	0.269856	0.205630	-1273.749900	-86.0	-1273.480044	-1273.541258	5.7	-1267.970953	-3.971102	-1271.766157
conformer_25	-1273.720993	0.269892	0.205965	-1273.749868	-75.8	-1273.479976	-1273.540891	6.7	-1267.975283	-3.971840	-1271.767022
conformer_33	-1273.712068	0.269796	0.204596	-1273.749701	-98.8	-1273.479905	-1273.542093	3.5	-1267.964878	-3.971772	-1271.766675
conformer_64	-1273.711087	0.269692	0.204235	-1273.749552	-101.0	-1273.479860	-1273.542305	3.0	-1267.963783	-3.971771	-1271.766772
conformer_1	-1273.723552	0.270184	0.206866	-1273.749406	-67.9	-1273.479222	-1273.539528	10.3	-1267.976227	-3.972818	-1271.765021
conformer_44	-1273.720143	0.269621	0.205862	-1273.749340	-76.7	-1273.479719	-1273.540466	7.8	-1267.970969	-3.974281	-1271.765572
conformer_59	-1273.718238	0.269820	0.206068	-1273.749023	-80.8	-1273.479203	-1273.539943	9.2	-1267.970945	-3.973226	-1271.765877
conformer_68	-1273.712330	0.269653	0.203961	-1273.748940	-96.1	-1273.479287	-1273.541967	3.9	-1267.966714	-3.970561	-1271.766912
conformer_66	-1273.711887	0.269731	0.204342	-1273.748603	-96.4	-1273.478872	-1273.541249	5.7	-1267.965274	-3.971476	-1271.766112

conformer_30	-1273.716747	0.270136	0.207033	-1273.748466	-83.3	-1273.478330	-1273.538421	13.2	-1267.966283	-3.975601	-1271.763559
conformer_43	-1273.717605	0.269762	0.204832	-1273.748108	-80.1	-1273.478346	-1273.540264	8.3	-1267.972115	-3.971197	-1271.765971
conformer_61	-1273.714081	0.269733	0.204256	-1273.747989	-89.0	-1273.478256	-1273.540721	7.1	-1267.969687	-3.969787	-1271.766114
conformer_53	-1273.710585	0.269841	0.204919	-1273.747487	-96.9	-1273.477646	-1273.539556	10.2	-1267.960881	-3.973893	-1271.763746
conformer_18	-1273.718097	0.270027	0.206935	-1273.747215	-76.4	-1273.477188	-1273.537268	16.2	-1267.967494	-3.976174	-1271.762839
conformer_34	-1273.714357	0.270216	0.206365	-1273.746863	-85.3	-1273.476647	-1273.537486	15.6	-1267.966031	-3.973887	-1271.763046
conformer_28	-1273.714517	0.270023	0.206126	-1273.745696	-81.9	-1273.475673	-1273.536558	18.1	-1267.965842	-3.974638	-1271.762521
conformer_37	-1273.712840	0.270203	0.206371	-1273.744942	-84.3	-1273.474739	-1273.535559	20.7	-1267.964069	-3.974145	-1271.760934
conformer_52	-1273.711681	0.270053	0.206155	-1273.743322	-83.1	-1273.473269	-1273.534155	24.4	-1267.962449	-3.974790	-1271.759713
conformer_29	-1273.714090	0.270022	0.206059	-1273.742657	-75.0	-1273.472635	-1273.533586	25.9	-1267.966080	-3.973976	-1271.759552
conformer_21	-1273.714971	0.270057	0.206220	-1273.741660	-70.1	-1273.471603	-1273.532428	28.9	-1267.967620	-3.973696	-1271.758773

[a]: Single-point calculation in aqueous phase with SMD model.

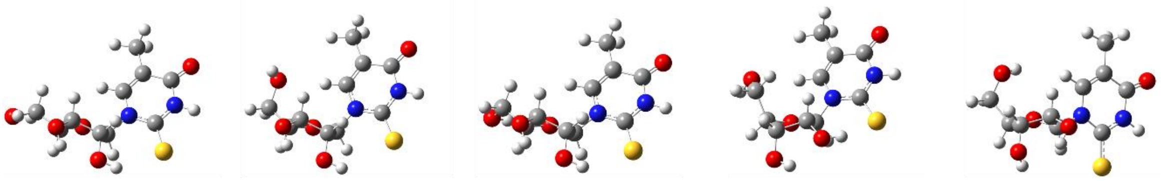

conformer_1 conformer_2 conformer_3 conformer_4 conformer_41

Figure S37. B3LYP-D3/def2-TZVPP optimized geometries of conformers for 2-thio-5-methyluridine (**1rb2th5mU**).

Table S35. Conformers of gas-phase optimized 2-thio-5-methyluridine (**1rb2th5mU**) at the B3LYP-D3/def2-TZVPP level of theory followed by aqueous phase single-point calculation. The columns display total energy without zero-point correction (E_{Tot}), thermal correction to enthalpy (δH), Gibbs free energy (δG), total energy without zero-point correction ($E_{Tot,W}$), Gibbs free energy ($G_{298,W}$) in water (W), total single-point energy (E_{CBS}) calculated at DLPNO-CCSD(T)/CBS level of theory, and their corresponding free energy $G_{CBS,W}$. $G_{298,W}$ and $G_{CBS,W}$ have been corrected to the standard state of 1 mol/L by addition of +7.908 kJ/mol. ΔG_{Solv} represents the Gibbs free energy of solvation. The data are arranged in the ascending numeric order of $E_{Tot,W}$. $\Delta G_{298,W}$ represents the respective energy difference to the lowest structure. Only conformers within the 24 kJ/mol (6 kcal/mol) energy window above the lowest in CREST are included in initial conformer sampling. Duplicates of the same structure are excluded. The overall optimum is marked bold.

	**B3LYP-D3/def2-TZVPP**			**SMD(H2O)/B3LYP-D3/def2-TZVPP**[a]					**DLPNO-CCSD(T)/CBS**		
**1rb2th5mU No.**	**E_{Tot}** (Hartree)	**δH** (Hartree)	**δG** (Hartree)	**$E_{Tot,W}$** (Hartree)	**ΔG_{Solv}** (kJ/mol)	**$H_{298,W}$** (Hartree)	**$G_{298,W}$** (Hartree)	**$\Delta G_{298,W}$** (kJ/mol)	**$E_{CBS,HF}$** (Hartree)	**$E_{CBS,C}$** (Hartree)	**$G_{CBS,W}$** (Hartree)
conformer_2	-1273.740402	0.270413	0.206841	-1273.771098	-80.6	-1273.500685	-1273.561245	2.9	-1267.995933	-3.968201	-1271.784976
conformer_5	-1273.739161	0.270386	0.206142	-1273.770981	-83.5	-1273.500595	-1273.561827	1.4	-1267.994619	-3.968218	-1271.785503
conformer_6	-1273.740728	0.270348	0.206607	-1273.770027	-76.9	-1273.499679	-1273.560408	5.1	-1267.997156	-3.967638	-1271.784474
conformer_54	-1273.733579	0.270203	0.205214	-1273.770004	-95.6	-1273.499801	-1273.561778	1.5	-1267.988031	-3.969228	-1271.785458
**conformer_41**	**-1273.730180**	**0.270061**	**0.204504**	**-1273.769872**	**-104.2**	**-1273.499811**	**-1273.562356**	**0**	**-1267.985754**	**-3.968056**	**-1271.785986**
conformer_10	-1273.739541	0.269888	0.206661	-1273.769801	-79.4	-1273.499913	-1273.560128	5.9	-1267.991993	-3.970612	-1271.783191
conformer_22	-1273.730697	0.270160	0.205861	-1273.769739	-102.5	-1273.499579	-1273.560866	3.9	-1267.985248	-3.968247	-1271.783663
conformer_37	-1273.729281	0.270066	0.204761	-1273.769672	-106.0	-1273.499606	-1273.561899	1.2	-1267.984679	-3.967929	-1271.785225
conformer_74	-1273.734496	0.270140	0.206338	-1273.769634	-92.3	-1273.499494	-1273.560284	5.4	-1267.989740	-3.969037	-1271.784565
conformer_53	-1273.730972	0.270087	0.205656	-1273.769556	-101.3	-1273.499469	-1273.560888	3.9	-1267.987399	-3.966889	-1271.784204
conformer_4	-1273.738346	0.270035	0.207120	-1273.769440	-81.6	-1273.499405	-1273.559308	8.0	-1267.989831	-3.972227	-1271.783019
conformer_3	-1273.738290	0.270266	0.206064	-1273.769421	-81.7	-1273.499155	-1273.560345	5.3	-1267.995236	-3.967417	-1271.784707
conformer_21	-1273.737133	0.269744	0.205349	-1273.769419	-84.8	-1273.499675	-1273.561058	3.4	-1267.989479	-3.971015	-1271.784420
conformer_19	-1273.732140	0.270217	0.206083	-1273.769181	-97.3	-1273.498964	-1273.560086	6.0	-1267.987656	-3.967749	-1271.783351
conformer_51	-1273.730064	0.269952	0.204335	-1273.769086	-102.5	-1273.499134	-1273.561739	1.6	-1267.987600	-3.966439	-1271.785714
conformer_7	-1273.740571	0.269961	0.206770	-1273.768943	-74.5	-1273.498982	-1273.559161	8.4	-1267.993979	-3.970114	-1271.782683
conformer_31	-1273.737202	0.269960	0.207139	-1273.768926	-83.3	-1273.498966	-1273.558775	9.4	-1267.988794	-3.972608	-1271.782974
conformer_42	-1273.732668	0.270109	0.205590	-1273.768863	-95.0	-1273.498754	-1273.560261	5.5	-1267.990156	-3.966277	-1271.784025
conformer_36	-1273.727609	0.270011	0.204544	-1273.768668	-107.8	-1273.498657	-1273.561112	3.3	-1267.984081	-3.966878	-1271.784462
conformer_8	-1273.735895	0.269752	0.205437	-1273.768651	-86.0	-1273.498899	-1273.560202	5.7	-1267.989904	-3.969647	-1271.783858
conformer_61	-1273.726597	0.269950	0.204245	-1273.768576	-110.2	-1273.498626	-1273.561319	2.7	-1267.982897	-3.966946	-1271.784565
conformer_16	-1273.735032	0.269658	0.205371	-1273.768575	-88.1	-1273.498917	-1273.560192	5.7	-1267.988450	-3.970107	-1271.783717
conformer_14	-1273.735868	0.270087	0.205546	-1273.768504	-85.7	-1273.498417	-1273.559946	6.3	-1267.993236	-3.967256	-1271.784569
conformer_55	-1273.732772	0.270213	0.206315	-1273.768340	-93.4	-1273.498127	-1273.559013	8.8	-1267.987998	-3.968993	-1271.783231
conformer_32	-1273.735690	0.270033	0.205449	-1273.767903	-84.6	-1273.497870	-1273.559442	7.7	-1267.993458	-3.967045	-1271.784255
conformer_68	-1273.727772	0.269865	0.203683	-1273.767898	-105.3	-1273.498033	-1273.561203	3.0	-1267.986011	-3.965561	-1271.785003
conformer_48	-1273.729192	0.269905	0.203730	-1273.767827	-101.4	-1273.497922	-1273.561085	3.3	-1267.987969	-3.965244	-1271.785106
conformer_40	-1273.735080	0.269632	0.204866	-1273.767449	-85.0	-1273.497817	-1273.559571	7.3	-1267.989492	-3.969545	-1271.783528
conformer_67	-1273.727391	0.269941	0.204275	-1273.767433	-105.1	-1273.497492	-1273.560146	5.8	-1267.984471	-3.966565	-1271.783792
conformer_28	-1273.732287	0.270265	0.206592	-1273.767431	-92.3	-1273.497166	-1273.557827	11.9	-1267.985691	-3.970436	-1271.781667

conformer_1	-1273.738277	0.270456	0.206814	-1273.767353	-76.3	-1273.496897	-1273.557527	12.7	-1267.994371	-3.968102	-1271.781723
conformer_59	-1273.733076	0.270136	0.206046	-1273.767295	-89.8	-1273.497159	-1273.558237	10.8	-1267.989119	-3.968572	-1271.782853
conformer_60	-1273.729553	0.269933	0.203990	-1273.766879	-98.0	-1273.496946	-1273.559877	6.5	-1267.988907	-3.964864	-1271.784095
conformer_73	-1273.729525	0.269993	0.206113	-1273.766354	-96.7	-1273.496361	-1273.557229	13.5	-1267.980252	-3.972486	-1271.780443
conformer_70	-1273.733300	0.270268	0.206852	-1273.766334	-86.7	-1273.496066	-1273.556470	15.5	-1267.986068	-3.971225	-1271.780464
conformer_23	-1273.732104	0.270076	0.206116	-1273.766243	-89.6	-1273.496167	-1273.557115	13.8	-1267.984547	-3.971198	-1271.780756
conformer_52	-1273.732298	0.270042	0.204808	-1273.766133	-88.8	-1273.496091	-1273.558313	10.6	-1267.990153	-3.966587	-1271.782754
conformer_17	-1273.733887	0.270271	0.206809	-1273.766130	-84.7	-1273.495859	-1273.556309	15.9	-1267.987137	-3.970903	-1271.780462
conformer_30	-1273.729835	0.270349	0.205788	-1273.765533	-93.7	-1273.495184	-1273.556733	14.8	-1267.985509	-3.968610	-1271.781017
conformer_33	-1273.730260	0.270246	0.205877	-1273.764357	-89.5	-1273.494111	-1273.555468	18.1	-1267.985602	-3.969293	-1271.780103
conformer_72	-1273.726435	0.270075	0.205540	-1273.763686	-97.8	-1273.493611	-1273.555134	19.0	-1267.981769	-3.969132	-1271.779600
conformer_34	-1273.728429	0.270369	0.206004	-1273.763656	-92.5	-1273.493287	-1273.554640	20.3	-1267.983550	-3.968819	-1271.778580
conformer_69	-1273.725374	0.270203	0.205268	-1273.762662	-97.9	-1273.492459	-1273.554382	20.9	-1267.979912	-3.969256	-1271.778177
conformer_43	-1273.727386	0.270197	0.205688	-1273.762027	-91.0	-1273.491830	-1273.553327	23.7	-1267.982093	-3.969528	-1271.777562
conformer_26	-1273.729802	0.270179	0.205603	-1273.761387	-82.9	-1273.491208	-1273.552772	25.2	-1267.985671	-3.968728	-1271.777369
conformer_18	-1273.730757	0.270211	0.205753	-1273.760511	-78.1	-1273.490300	-1273.551746	27.9	-1267.987291	-3.968422	-1271.776702

[a]: Single-point calculation in aqueous phase with SMD model.

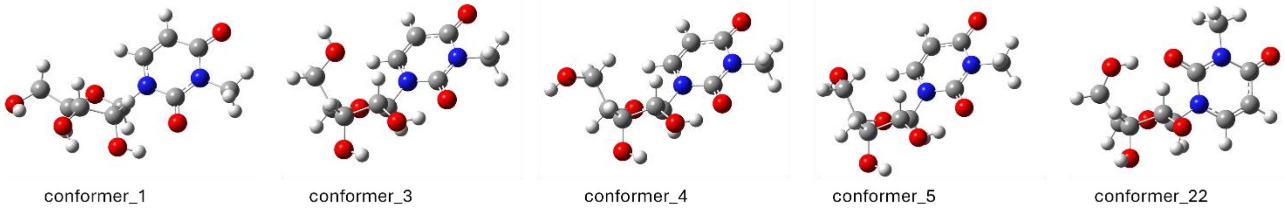

Figure S38. B3LYP-D3/def2-TZVPP optimized geometries of conformers for 3-methyluridine (**1rb3mU**).

Table S36. Conformers of gas-phase optimized 3-methyluridine (**1rb3mU**) at the B3LYP-D3/def2-TZVPP level of theory followed by aqueous phase single-point calculation. The columns display total energy without zero-point correction (E_{Tot}), thermal correction to enthalpy (δH), Gibbs free energy (δG), total energy without zero-point correction ($E_{Tot,W}$), Gibbs free energy ($G_{298,W}$) in water (W), total single-point energy (E_{CBS}) calculated at DLPNO-CCSD(T)/CBS level of theory, and their corresponding free energy $G_{CBS,W}$. $G_{298,W}$ and $G_{CBS,W}$ have been corrected to the standard state of 1 mol/L by addition of +7.908 kJ/mol. ΔG_{Solv} represents the Gibbs free energy of solvation. The data are arranged in the ascending numeric order of $E_{Tot,W}$. $\Delta G_{298,W}$ represents the respective energy difference to the lowest structure. Only conformers within the 24 kJ/mol (6 kcal/mol) energy window above the lowest in CREST are included in initial conformer sampling. Duplicates of the same structure are excluded. The overall optimum is marked bold.

	B3LYP-D3/def2-TZVPP			SMD(H2O)/B3LYP-D3/def2-TZVPP[a]					DLPNO-CCSD(T)/CBS		
1rb3mU No.	E_{Tot} (Hartree)	δH (Hartree)	δG (Hartree)	$E_{Tot,W}$ (Hartree)	ΔG_{Solv} (kJ/mol)	$H_{298,W}$ (Hartree)	$G_{298,W}$ (Hartree)	$\Delta G_{298,W}$ (kJ/mol)	$E_{CBS,HF}$ (Hartree)	$E_{CBS,C}$ (Hartree)	$G_{CBS,W}$ (Hartree)
**conformer_22**	**-950.780220**	**0.272354**	**0.209367**	**-950.812630**	**-85.1**	**-950.540276**	**-950.600251**	**0.0**	**-945.350498**	**-4.016546**	**-949.187075**
conformer_50	-950.781235	0.272338	0.210715	-950.812338	-81.7	-950.540000	-950.598611	4.3	-945.352141	-4.016367	-949.185884
conformer_19	-950.778483	0.272262	0.210464	-950.812278	-88.7	-950.540016	-950.598802	3.8	-945.347459	-4.017543	-949.185322
conformer_9	-950.780576	0.272266	0.210433	-950.812230	-83.1	-950.539964	-950.598785	3.8	-945.351397	-4.016237	-949.185843
conformer_54	-950.780772	0.272353	0.210147	-950.811960	-81.9	-950.539607	-950.598801	3.8	-945.351781	-4.016357	-949.186167
conformer_15	-950.780078	0.272281	0.209865	-950.811864	-83.5	-950.539583	-950.598987	3.3	-945.351005	-4.016195	-949.186108
conformer_53	-950.777960	0.272371	0.210130	-950.811468	-88.0	-950.539097	-950.598326	5.1	-945.348698	-4.016031	-949.185096
conformer_35	-950.779029	0.272260	0.209215	-950.810775	-83.3	-950.538515	-950.598548	4.5	-945.352207	-4.013776	-949.185502
conformer_37	-950.778886	0.272313	0.209257	-950.810652	-83.4	-950.538339	-950.598383	4.9	-945.352087	-4.013790	-949.185375
onformer_24	-950.779953	0.272183	0.209820	-950.810590	-80.4	-950.538407	-950.597758	6.5	-945.350698	-4.016454	-949.184957
conformer_39	-950.777824	0.272236	0.207621	-950.810408	-85.5	-950.538172	-950.599775	1.3	-945.351120	-4.013773	-949.186844
conformer_28	-950.779653	0.272216	0.209647	-950.810356	-80.6	-950.538140	-950.597697	6.7	-945.350515	-4.016441	-949.184999
conformer_104	-950.772219	0.272067	0.207707	-950.810328	-100.1	-950.538261	-950.599609	1.7	-945.344628	-4.014832	-949.186850
conformer_41	-950.777674	0.272261	0.207103	-950.810235	-85.5	-950.537974	-950.600120	0.3	-945.351078	-4.013716	-949.187241
conformer_11	-950.781362	0.272039	0.209350	-950.810220	-75.8	-950.538181	-950.597858	6.3	-945.350480	-4.017965	-949.184940
conformer_5	-950.780193	0.272197	0.210169	-950.810044	-78.4	-950.537847	-950.596863	8.9	-945.350865	-4.016724	-949.184258
conformer_16	-950.781032	0.272046	0.208730	-950.809987	-76.0	-950.537941	-950.598245	5.3	-945.350157	-4.018044	-949.185414
conformer_10	-950.781038	0.272096	0.209549	-950.809971	-76.0	-950.537875	-950.597410	7.5	-945.351172	-4.016656	-949.184200
conformer_8	-950.779942	0.272213	0.209758	-950.809810	-78.4	-950.537597	-950.597040	8.4	-945.350677	-4.016773	-949.184548
conformer_77	-950.772125	0.272035	0.208714	-950.809802	-98.9	-950.537767	-950.598076	5.7	-945.343466	-4.014973	-949.184390
conformer_20	-950.779541	0.272145	0.209224	-950.809754	-79.3	-950.537609	-950.597518	7.2	-945.350001	-4.016556	-949.184535
conformer_14	-950.780779	0.272121	0.209091	-950.809739	-76.0	-950.537618	-950.597636	6.9	-945.350989	-4.016679	-949.184525
conformer_132	-950.775073	0.272170	0.208609	-950.809733	-91.0	-950.537563	-950.598112	5.6	-945.346657	-4.015563	-949.185260
conformer_109	-950.773614	0.272015	0.208513	-950.809732	-94.8	-950.537717	-950.598207	5.4	-945.344236	-4.015926	-949.184755
conformer_98	-950.771141	0.272015	0.207703	-950.809721	-101.3	-950.537706	-950.599006	3.3	-945.343916	-4.014339	-949.186120
conformer_155	-950.772416	0.271919	0.208269	-950.809712	-97.9	-950.537793	-950.598431	4.8	-945.345708	-4.013521	-949.185245
conformer_45	-950.779426	0.272250	0.209156	-950.809691	-79.5	-950.537441	-950.597523	7.2	-945.353492	-4.013270	-949.184859
conformer_110	-950.771725	0.272005	0.208242	-950.809594	-99.4	-950.537589	-950.598340	5.0	-945.342876	-4.015931	-949.185422
conformer_55	-950.775290	0.272293	0.208498	-950.809588	-90.1	-950.537295	-950.598078	5.7	-945.349043	-4.013839	-949.185671
conformer_46	-950.779278	0.272306	0.209210	-950.809571	-79.5	-950.537265	-950.597349	7.6	-945.353368	-4.013284	-949.184723

conformer_121	-950.773442	0.272056	0.208535	-950.809561	-94.8	-950.537505	-950.598014	5.9	-945.344095	-4.015959	-949.184625
conformer_47	-950.779330	0.272114	0.210052	-950.809535	-79.3	-950.537421	-950.596471	9.9	-945.350552	-4.016601	-949.184294
conformer_3	-950.782480	0.272130	0.209600	-950.809481	-70.9	-950.537351	-950.596869	8.9	-945.353383	-4.016484	-949.184256
conformer_105	-950.770839	0.271993	0.206988	-950.809442	-101.4	-950.537449	-950.599442	2.1	-945.343672	-4.014362	-949.186637
conformer_144	-950.774771	0.272176	0.208279	-950.809420	-91.0	-950.537244	-950.598129	5.6	-945.346404	-4.015607	-949.185369
conformer_123	-950.771557	0.272035	0.207979	-950.809410	-99.4	-950.537375	-950.598419	4.8	-945.342721	-4.015991	-949.185574
conformer_57	-950.775010	0.272328	0.208383	-950.809337	-90.1	-950.537009	-950.597942	6.1	-945.348808	-4.013866	-949.185605
conformer_64	-950.773752	0.272096	0.208988	-950.809290	-93.3	-950.537194	-950.597290	7.8	-945.346014	-4.014509	-949.184061
conformer_122	-950.772049	0.272004	0.207901	-950.809265	-97.7	-950.537261	-950.598352	5.0	-945.346729	-4.012923	-949.185956
conformer_6	-950.782201	0.272174	0.209297	-950.809262	-71.0	-950.537088	-950.596953	8.7	-945.353171	-4.016499	-949.184422
conformer_133	-950.771673	0.272099	0.206980	-950.809219	-98.6	-950.537120	-950.599227	2.7	-945.346269	-4.012859	-949.186682
conformer_125	-950.774324	0.271976	0.208415	-950.809044	-91.2	-950.537068	-950.597617	6.9	-945.348588	-4.012995	-949.184876
conformer_72	-950.774917	0.272228	0.209925	-950.809020	-89.5	-950.536792	-950.596083	10.9	-945.347064	-4.015420	-949.183650
conformer_159	-950.771344	0.272007	0.207698	-950.809010	-98.9	-950.537003	-950.598300	5.1	-945.345271	-4.013625	-949.185853
conformer_4	-950.778851	0.272054	0.208851	-950.808959	-79.1	-950.536905	-950.597096	8.3	-945.350750	-4.015486	-949.184482
conformer_74	-950.773970	0.272291	0.210127	-950.808950	-91.8	-950.536659	-950.595811	11.7	-945.344027	-4.017065	-949.182933
conformer_145	-950.771403	0.272108	0.206711	-950.808905	-98.5	-950.536797	-950.599182	2.8	-945.346033	-4.012892	-949.186704
conformer_91	-950.769429	0.271928	0.207491	-950.808857	-103.5	-950.536929	-950.598354	5.0	-945.342785	-4.013608	-949.185317
conformer_27	-950.777974	0.271907	0.208432	-950.808855	-81.1	-950.536948	-950.597411	7.5	-945.349446	-4.015822	-949.184705
conformer_71	-950.773100	0.272061	0.208137	-950.808843	-93.8	-950.536782	-950.597694	6.7	-945.343367	-4.016347	-949.184307
conformer_137	-950.774014	0.271979	0.207930	-950.808765	-91.2	-950.536786	-950.597823	6.4	-945.348334	-4.013073	-949.185216
conformer_32	-950.777670	0.271928	0.207993	-950.808684	-81.4	-950.536756	-950.597679	6.8	-945.349193	-4.015881	-949.185082
conformer_49	-950.776299	0.272186	0.208753	-950.808658	-85.0	-950.536472	-950.596893	8.8	-945.349836	-4.014400	-949.184831
conformer_101	-950.769135	0.271949	0.207049	-950.808588	-103.6	-950.536639	-950.598527	4.5	-945.342532	-4.013625	-949.185549
conformer_156	-950.768367	0.271749	0.206206	-950.808588	-105.6	-950.536839	-950.599370	2.3	-945.341799	-4.013541	-949.186343
conformer_75	-950.772812	0.272061	0.207738	-950.808530	-93.8	-950.536469	-950.597780	6.5	-945.343160	-4.016311	-949.184439
conformer_52	-950.775981	0.272222	0.208605	-950.808403	-85.1	-950.536181	-950.596786	9.1	-945.349561	-4.014452	-949.184818
conformer_130	-950.774025	0.272019	0.208437	-950.808365	-90.2	-950.536346	-950.596916	8.8	-945.347073	-4.014505	-949.184468
conformer_164	-950.768075	0.271767	0.205894	-950.808354	-105.8	-950.536587	-950.599448	2.1	-945.341497	-4.013624	-949.186494
conformer_143	-950.773837	0.272047	0.208356	-950.808198	-90.2	-950.536151	-950.596830	9.0	-945.346899	-4.014554	-949.184447
conformer_170	-950.769043	0.271928	0.208139	-950.808077	-102.5	-950.536149	-950.596926	8.7	-945.340323	-4.015860	-949.184067
conformer_173	-950.768874	0.271964	0.208083	-950.808008	-102.7	-950.536044	-950.596913	8.8	-945.340197	-4.015883	-949.184118
conformer_138	-950.772625	0.271930	0.208020	-950.808004	-92.9	-950.536074	-950.596972	8.6	-945.346366	-4.014101	-949.184814
conformer_60	-950.774225	0.272387	0.209242	-950.807995	-88.7	-950.535608	-950.595741	11.8	-945.347461	-4.014090	-949.183067
conformer_38	-950.778251	0.271995	0.208757	-950.807952	-78.0	-950.535957	-950.596183	10.7	-945.350672	-4.015282	-949.183887
conformer_128	-950.771123	0.271837	0.206771	-950.807922	-96.6	-950.536085	-950.598139	5.5	-945.346907	-4.011942	-949.185866
conformer_44	-950.777948	0.272033	0.208398	-950.807785	-78.3	-950.535752	-950.596375	10.2	-945.350408	-4.015324	-949.184158
conformer_92	-950.771257	0.272064	0.208444	-950.807692	-95.7	-950.535628	-950.596236	10.5	-945.342148	-4.015849	-949.182976
conformer_167	-950.769289	0.271847	0.207199	-950.807674	-100.8	-950.535827	-950.597463	7.3	-945.343239	-4.013302	-949.184715
conformer_139	-950.770817	0.271843	0.206188	-950.807651	-96.7	-950.535808	-950.598451	4.7	-945.346640	-4.011966	-949.186240
conformer_18	-950.778612	0.272254	0.208585	-950.807546	-76.0	-950.535292	-950.595949	11.3	-945.352331	-4.014385	-949.184053
conformer_96	-950.771098	0.272096	0.208371	-950.807534	-95.7	-950.535438	-950.596151	10.8	-945.342014	-4.015881	-949.182948
conformer_12	-950.778829	0.272228	0.208793	-950.807484	-75.2	-950.535256	-950.595679	12.0	-945.352446	-4.014390	-949.183685
conformer_174	-950.768991	0.271864	0.206736	-950.807389	-100.8	-950.535525	-950.597641	6.9	-945.342988	-4.013318	-949.184956
conformer_88	-950.772519	0.271981	0.208306	-950.807140	-90.9	-950.535159	-950.595822	11.6	-945.346385	-4.013842	-949.183530
conformer_87	-950.770467	0.272068	0.208723	-950.807085	-96.1	-950.535017	-950.595350	12.9	-945.342040	-4.015550	-949.182473
conformer_152	-950.771534	0.271864	0.206924	-950.806964	-93.0	-950.535100	-950.597028	8.5	-945.347966	-4.011480	-949.184941
conformer_13	-950.778482	0.272235	0.208732	-950.806900	-74.6	-950.534665	-950.595156	13.4	-945.353274	-4.013413	-949.183362
conformer_17	-950.778293	0.272251	0.208375	-950.806829	-74.9	-950.534578	-950.595442	12.6	-945.353170	-4.013457	-949.183776
conformer_112	-950.770999	0.272234	0.208664	-950.806687	-93.7	-950.534453	-950.595011	13.8	-945.343295	-4.014729	-949.182037
conformer_160	-950.771226	0.271869	0.206330	-950.806685	-93.1	-950.534816	-950.597343	7.6	-945.347699	-4.011511	-949.185327
conformer_70	-950.773873	0.272183	0.209089	-950.806421	-85.5	-950.534238	-950.594320	15.6	-945.346826	-4.014650	-949.181923
conformer_56	-950.776053	0.272188	0.208949	-950.806267	-79.3	-950.534079	-950.594306	15.6	-945.350023	-4.013958	-949.182233
conformer_78	-950.773527	0.272204	0.208841	-950.806174	-85.7	-950.533970	-950.594321	15.6	-945.346548	-4.014649	-949.181991
conformer_113	-950.770420	0.272108	0.209094	-950.806163	-93.8	-950.534055	-950.594057	16.3	-945.342244	-4.015335	-949.181216
conformer_147	-950.773396	0.272055	0.208504	-950.806123	-85.9	-950.534068	-950.594607	14.8	-945.348025	-4.013253	-949.182488

conformer_34	-950.776229	0.272082	0.208500	-950.806050	-78.3	-950.533968	-950.594538	15.0	-945.351340	-4.013373	-949.183021
conformer_59	-950.775726	0.272212	0.208712	-950.805971	-79.4	-950.533759	-950.594247	15.8	-945.349771	-4.013986	-949.182278
conformer_36	-950.776019	0.272093	0.208163	-950.805956	-78.6	-950.533863	-950.594781	14.4	-945.351232	-4.013343	-949.183337
conformer_84	-950.772986	0.272073	0.207912	-950.805767	-86.1	-950.533694	-950.594843	14.2	-945.347671	-4.013246	-949.182774
conformer_120	-950.770926	0.271988	0.208060	-950.805691	-91.3	-950.533703	-950.594619	14.8	-945.343417	-4.014957	-949.182066
conformer_61	-950.776171	0.272039	0.208406	-950.805532	-77.1	-950.533493	-950.594114	16.1	-945.351738	-4.013056	-949.182737
conformer_65	-950.775962	0.272050	0.208060	-950.805443	-77.4	-950.533393	-950.594371	15.4	-945.351623	-4.013048	-949.183080
conformer_136	-950.773497	0.271968	0.206676	-950.805384	-83.7	-950.533416	-950.595696	12.0	-945.347868	-4.013604	-949.183671
conformer_69	-950.776772	0.272169	0.208789	-950.805350	-75.0	-950.533181	-950.593549	17.6	-945.351480	-4.013615	-949.181873
conformer_1	-950.778232	0.272347	0.209364	-950.805198	-70.8	-950.532851	-950.592822	19.5	-945.352361	-4.013732	-949.180683
conformer_2	-950.778064	0.272380	0.209231	-950.805154	-71.1	-950.532774	-950.592911	19.3	-945.352250	-4.013758	-949.180856
conformer_148	-950.773198	0.271997	0.206929	-950.805078	-83.7	-950.533081	-950.595137	13.4	-945.347622	-4.013619	-949.183180
conformer_51	-950.776444	0.272195	0.208523	-950.805035	-75.1	-950.532840	-950.593500	17.7	-945.351228	-4.013651	-949.181935
conformer_85	-950.770265	0.272230	0.209680	-950.804631	-90.2	-950.532401	-950.591939	21.8	-945.337106	-4.019688	-949.178468
conformer_168	-950.770054	0.272258	0.210095	-950.804610	-90.7	-950.532352	-950.591503	23.0	-945.336221	-4.020767	-949.178438
conformer_162	-950.770913	0.272042	0.209111	-950.803249	-84.9	-950.531207	-950.591126	24.0	-945.341806	-4.017144	-949.179163
conformer_142	-950.769238	0.272186	0.210119	-950.801896	-85.7	-950.529710	-950.588765	30.2	-945.336819	-4.019809	-949.176154
conformer_163	-950.766194	0.272508	0.210514	-950.797896	-83.2	-950.525388	-950.584370	41.7	-945.332101	-4.021482	-949.171759

[a]: Single-point calculation in aqueous phase with SMD model.

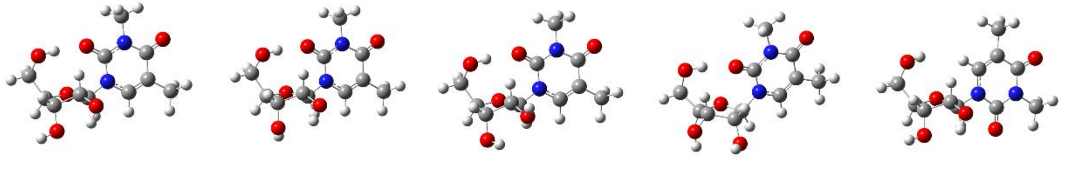

Figure S39. B3LYP-D3/def2-TZVPP optimized geometries of conformers for 3,5-dimethyluridine (**1rb3m5mU**).

Table S37. Conformers of gas-phase optimized 3,5-dimethyluridine (**1rb3m5mU**) at the B3LYP-D3/def2-TZVPP level of theory followed by aqueous phase single-point calculation. The columns display total energy without zero-point correction (E_{Tot}), thermal correction to enthalpy (δH), Gibbs free energy (δG), total energy without zero-point correction ($E_{Tot,W}$), Gibbs free energy ($G_{298,W}$) in water (W), total single-point energy (E_{CBS}) calculated at DLPNO-CCSD(T)/CBS level of theory, and their corresponding free energy $G_{CBS,W}$. $G_{298,W}$ and $G_{CBS,W}$ have been corrected to the standard state of 1 mol/L by addition of +7.908 kJ/mol. ΔG_{Solv} represents the Gibbs free energy of solvation. The data are arranged in the ascending numeric order of $E_{Tot,W}$. $\Delta G_{298,W}$ represents the respective energy difference to the lowest structure. Only conformers within the 24 kJ/mol (6 kcal/mol) energy window above the lowest in CREST are included in initial conformer sampling. Duplicates of the same structure are excluded. The overall optimum is marked bold.

	B3LYP-D3/def2-TZVPP			SMD(H₂O)/B3LYP-D3/def2-TZVPP[a]					DLPNO-CCSD(T)/CBS		
**1rb3m5mU No.**	**E_{Tot}** (Hartree)	**δH** (Hartree)	**δG** (Hartree)	**$E_{Tot,W}$** (Hartree)	**ΔG_{Solv}** (kJ/mol)	**$H_{298,W}$** (Hartree)	**$G_{298,W}$** (Hartree)	**$\Delta G_{298,W}$** (kJ/mol)	**$E_{CBS,HF}$** (Hartree)	**$E_{CBS,C}$** (Hartree)	**$G_{CBS,W}$** (Hartree)
conformer_25	-990.1158949	0.3017420	0.2365280	-990.1486787	-86.1	-989.846937	-989.909139	5.3	-984.4019192	-4.2274616	-988.4226245
conformer_13	-990.1179068	0.3017220	0.2364660	-990.1486549	-80.7	-989.846933	-989.909177	5.2	-984.4057778	-4.2261312	-988.4231790
conformer_51	-990.1182672	0.3018710	0.2363140	-990.1484638	-79.3	-989.846593	-989.909138	5.3	-984.4062832	-4.2261979	-988.4233517
conformer_52	-990.1152237	0.3016970	0.2357070	-990.1475623	-84.9	-989.845865	-989.908843	6.1	-984.4030316	-4.2259985	-988.4226496
conformer_39	-990.1160334	0.3015970	0.2349720	-990.1467957	-80.8	-989.845199	-989.908812	6.1	-984.4059058	-4.2241344	-988.4228184
conformer_123	-990.1095858	0.3014720	0.2335930	-990.1467755	-97.6	-989.845304	-989.910171	2.6	-984.3989274	-4.2248312	-988.4243433
conformer_41	-990.1159741	0.3016150	0.2349310	-990.1467420	-80.8	-989.845127	-989.908799	6.2	-984.4059028	-4.2241579	-988.4228856
conformer_28	-990.1172416	0.3015960	0.2357980	-990.1466830	-77.3	-989.845087	-989.907873	8.6	-984.4049816	-4.2264700	-988.4220830
conformer_38	-990.1150151	0.3015990	0.2339850	-990.1466659	-83.1	-989.845067	-989.909669	3.9	-984.4049346	-4.2241006	-988.4236890
conformer_130	-990.1093753	0.3014720	0.2332710	-990.1466342	-97.8	-989.845162	-989.910351	2.1	-984.3987917	-4.2248319	-988.4245996
conformer_40	-990.1149613	0.3016370	0.2341000	-990.1466013	-83.1	-989.844964	-989.909489	4.4	-984.4049346	-4.2240877	-988.4235503
conformer_10	-990.1186891	0.3014840	0.2355300	-990.1465634	-73.2	-989.845079	-989.908021	8.2	-984.4044791	-4.2282434	-988.4220548
conformer_14	-990.1184593	0.3014800	0.2352730	-990.1464979	-73.6	-989.845018	-989.908213	7.7	-984.4042703	-4.2282836	-988.4223075
conformer_142	-990.1123775	0.3015480	0.2343610	-990.1462098	-88.8	-989.844662	-989.908837	6.1	-984.4007501	-4.2257852	-988.4229945
conformer_3	-990.1173702	0.3015110	0.2356790	-990.1461395	-75.5	-989.844629	-989.907449	9.7	-984.4047943	-4.2270083	-988.4218809
conformer_17	-990.1180787	0.3014900	0.2351450	-990.1461219	-73.6	-989.844632	-989.907965	8.4	-984.4050717	-4.2268080	-988.4217658
conformer_80	-990.1092338	0.3014560	0.2346260	-990.1460398	-96.6	-989.844584	-989.908402	7.2	-984.3975292	-4.2250309	-988.4217281
conformer_95	-990.1085684	0.3013220	0.2333420	-990.1460350	-98.4	-989.844713	-989.909681	3.9	-984.3978404	-4.2247866	-988.4237396
conformer_5	-990.1172358	0.3015510	0.2355630	-990.1460336	-75.6	-989.844483	-989.907459	9.7	-984.4047343	-4.2270464	-988.4220035
conformer_23	-990.1179239	0.3015160	0.2350110	-990.1460125	-73.7	-989.844496	-989.907989	8.3	-984.4050155	-4.2267757	-988.4218568
conformer_136	-990.1103926	0.3013020	0.2321380	-990.1460070	-93.5	-989.844705	-989.910857	0.8	-984.4013646	-4.2235721	-988.4254012
conformer_117	-990.1083720	0.3013160	0.2327800	-990.1458754	-98.5	-989.844559	-989.910083	2.8	-984.3977015	-4.2247921	-988.4242051
conformer_182	-990.1094456	0.3013230	0.2342180	-990.1458719	-95.6	-989.844549	-989.908642	6.6	-984.3996873	-4.2236108	-988.4224944
conformer_22	-990.1168632	0.3014410	0.2350090	-990.1458652	-76.1	-989.844424	-989.907844	8.7	-984.4042630	-4.2267253	-988.4219693
conformer_27	-990.1166969	0.3014600	0.2346430	-990.1458487	-76.5	-989.844389	-989.908194	7.8	-984.4041603	-4.2266446	-988.4223017
**conformer_145**	**-990.1101764**	**0.3012990**	**0.2316880**	**-990.1458480**	**-93.7**	**-989.844549**	**-989.911148**	**0**	**-984.4012130**	**-4.2235888**	**-988.4257734**
conformer_87	-990.1090464	0.3014690	0.2344920	-990.1458443	-96.6	-989.844375	-989.908340	7.4	-984.3974172	-4.2250378	-988.4217489
conformer_47	-990.1164089	0.3014200	0.2355590	-990.1457434	-77.0	-989.844323	-989.907172	10.4	-984.4042178	-4.2271230	-988.4221043
conformer_50	-990.1162674	0.3014540	0.2355120	-990.1456878	-77.2	-989.844234	-989.907164	10.5	-984.4041348	-4.2271436	-988.4221749
conformer_191	-990.1092564	0.3013360	0.2341060	-990.1456658	-95.6	-989.844330	-989.908548	6.8	-984.3995643	-4.2236178	-988.4224735

conformer_55	-990.1125568	0.3017740	0.2346390	-990.1456636	-86.9	-989.843890	-989.908013	8.2	-984.4033558	-4.2236936	-988.4225051
conformer_132	-990.1089246	0.3013890	0.2343700	-990.1456531	-96.4	-989.844264	-989.908271	7.6	-984.3968549	-4.2261175	-988.4223188
conformer_45	-990.1162957	0.3015640	0.2348330	-990.1456118	-77.0	-989.844048	-989.907767	8.9	-984.4071136	-4.2235641	-988.4221488
conformer_149	-990.1090190	0.3014470	0.2329130	-990.1456074	-96.1	-989.844160	-989.909682	3.8	-984.4006692	-4.2228002	-988.4241328
conformer_120	-990.1106864	0.3014060	0.2341300	-990.1455943	-91.7	-989.844188	-989.908452	7.1	-984.3980054	-4.2262260	-988.4219972
conformer_133	-990.1088575	0.3014090	0.2343300	-990.1455670	-96.4	-989.844158	-989.908225	7.7	-984.3968297	-4.2261207	-988.4223179
conformer_146	-990.1162277	0.3015770	0.2347620	-990.1455582	-77.0	-989.843981	-989.907784	8.8	-984.4071102	-4.2235884	-988.4222551
conformer_57	-990.1123648	0.3017910	0.2343140	-990.1455240	-87.1	-989.843733	-989.908198	7.7	-984.4032709	-4.2236629	-988.4227671
conformer_77	-990.1107555	0.3015290	0.2348760	-990.1455167	-91.3	-989.843988	-989.907629	9.2	-984.4000287	-4.2244861	-988.4213880
conformer_125	-990.1106054	0.3014160	0.2338630	-990.1455083	-91.6	-989.844092	-989.908633	6.6	-984.3979927	-4.2262295	-988.4222502
conformer_143	-990.1093064	0.3012760	0.2332990	-990.1455060	-95.0	-989.844230	-989.909195	5.1	-984.4009293	-4.2230204	-988.4238384
conformer_155	-990.1088564	0.3014530	0.2326860	-990.1455012	-96.2	-989.844048	-989.909803	3.5	-984.4005602	-4.2228306	-988.4243376
conformer_110	-990.1067126	0.3013120	0.2331960	-990.1453798	-101.5	-989.844068	-989.909172	5.2	-984.3970947	-4.2235635	-988.4231174
conformer_148	-990.1091041	0.3012710	0.2328100	-990.1453311	-95.1	-989.844060	-989.909509	4.3	-984.4007946	-4.2230373	-988.4242370
conformer_84	-990.1105625	0.3015370	0.2346840	-990.1453282	-91.3	-989.843791	-989.907632	9.2	-984.3999116	-4.2244722	-988.4214535
conformer_9	-990.1193799	0.3015290	0.2353540	-990.1453250	-68.1	-989.843796	-989.906959	11.0	-984.4071552	-4.2266634	-988.4213977
conformer_61	-990.1106093	0.3015450	0.2341850	-990.1453048	-91.1	-989.843760	-989.908108	8.0	-984.3976916	-4.2264961	-988.4216862
conformer_11	-990.1191970	0.3015560	0.2350900	-990.1452720	-68.5	-989.843716	-989.907170	10.4	-984.4070792	-4.2266407	-988.4216929
conformer_122	-990.1065182	0.3013240	0.2329530	-990.1452524	-101.7	-989.843928	-989.909287	4.9	-984.3969586	-4.2235572	-988.4232851
conformer_163	-990.1112460	0.3013890	0.2343220	-990.1452482	-89.3	-989.843859	-989.907914	8.5	-984.4025136	-4.2229855	-988.4221673
conformer_174	-990.1056690	0.3012070	0.2326560	-990.1452252	-103.9	-989.844018	-989.909557	4.2	-984.3960031	-4.2235800	-988.4234713
conformer_63	-990.1104299	0.3015550	0.2338000	-990.1450944	-91.0	-989.843539	-989.908282	7.5	-984.3975995	-4.2264366	-988.4218886
conformer_64	-990.1104299	0.3015550	0.2338000	-990.1450943	-91.0	-989.843539	-989.908282	7.5	-984.3975994	-4.2264366	-988.4218885
conformer_186	-990.1054781	0.3012150	0.2324170	-990.1450929	-104.0	-989.843878	-989.909664	3.9	-984.3958668	-4.2235609	-988.4236136
conformer_172	-990.1085707	0.3015040	0.2340260	-990.1450911	-95.9	-989.843587	-989.908053	8.1	-984.3995328	-4.2234699	-988.4224851
conformer_169	-990.1110503	0.3013970	0.2341520	-990.1450391	-89.2	-989.843642	-989.907875	8.6	-984.4023845	-4.2229981	-988.4222073
conformer_82	-990.1120661	0.3016010	0.2357780	-990.1450260	-86.5	-989.843425	-989.906236	12.9	-984.4013060	-4.2254050	-988.4208809
conformer_72	-990.1112096	0.3016650	0.2360120	-990.1449817	-88.7	-989.843317	-989.905958	13.6	-984.3983492	-4.2270822	-988.4201795
conformer_83	-990.1112096	0.3016650	0.2360120	-990.1449816	-88.7	-989.843317	-989.905958	13.6	-984.3983492	-4.2270822	-988.4201794
conformer_4	-990.1160204	0.3015050	0.2348450	-990.1449292	-75.9	-989.843424	-989.907072	10.7	-984.4048498	-4.2255001	-988.4214017
conformer_31	-990.1151933	0.3013820	0.2346280	-990.1448949	-78.0	-989.843513	-989.907255	10.2	-984.4035774	-4.2258517	-988.4214906
conformer_7	-990.1158327	0.3015460	0.2346650	-990.1448855	-76.3	-989.843340	-989.907209	10.3	-984.4047260	-4.2254919	-988.4215937
conformer_33	-990.1150067	0.3014250	0.2344470	-990.1448627	-78.4	-989.843438	-989.907404	9.8	-984.4034549	-4.2258486	-988.4217006
conformer_48	-990.1134961	0.3015550	0.2343340	-990.1448564	-82.3	-989.843301	-989.907510	9.6	-984.4041130	-4.2242709	-988.4223981
conformer_88	-990.1109551	0.3016820	0.2357440	-990.1447273	-88.7	-989.843045	-989.905971	13.6	-984.3981931	-4.2270115	-988.4202208
conformer_53	-990.1132734	0.3015790	0.2339190	-990.1446742	-82.4	-989.843095	-989.907743	8.9	-984.4039533	-4.2242679	-988.4226910
conformer_151	-990.1082715	0.3012420	0.2325070	-990.1444505	-95.0	-989.843209	-989.908932	5.8	-984.4010716	-4.2218631	-988.4235947
conformer_150	-990.1097811	0.3012930	0.2336740	-990.1441840	-90.3	-989.842891	-989.907498	9.6	-984.4006386	-4.2239770	-988.4223325
conformer_202	-990.1065158	0.3012230	0.2328440	-990.1441354	-98.8	-989.842912	-989.908279	7.5	-984.3975241	-4.2232191	-988.4225069
conformer_60	-990.1114614	0.3017660	0.2350450	-990.1440696	-85.6	-989.842304	-989.906013	13.5	-984.4017724	-4.2239622	-988.4202858
conformer_65	-990.1112600	0.3017870	0.2349610	-990.1439582	-85.8	-989.842171	-989.905985	13.6	-984.4016686	-4.2239699	-988.4203637
conformer_43	-990.1153390	0.3013990	0.2344630	-990.1439154	-75.0	-989.842516	-989.906440	12.4	-984.4047124	-4.2252810	-988.4210948
conformer_44	-990.1151479	0.3014420	0.2343100	-990.1438903	-75.5	-989.842448	-989.906568	12.0	-984.4045776	-4.2252882	-988.4212862
conformer_91	-990.1084223	0.3014880	0.2343950	-990.1436203	-92.4	-989.842132	-989.906213	13.0	-984.3961155	-4.2261409	-988.4200473
conformer_100	-990.1083577	0.3015020	0.2342560	-990.1435516	-92.4	-989.842050	-989.906284	12.8	-984.3960921	-4.2261572	-988.4201753
conformer_185	-990.1086448	0.3012650	0.2326930	-990.1435412	-91.6	-989.842276	-989.907836	8.7	-984.4020539	-4.2214327	-988.4226779
conformer_180	-990.1061963	0.3013620	0.2333040	-990.1435337	-98.0	-989.842172	-989.907218	10.3	-984.3939943	-4.2263607	-988.4213765
conformer_192	-990.1084413	0.3012730	0.2324110	-990.1433859	-91.7	-989.842113	-989.907963	8.4	-984.4019080	-4.2214542	-988.4228838
conformer_20	-990.1158752	0.3016210	0.2342110	-990.1432382	-71.8	-989.841617	-989.906015	13.5	-984.4065261	-4.2245037	-988.4211698
conformer_15	-990.1159845	0.3015900	0.2345190	-990.1431671	-71.4	-989.841577	-989.905636	14.5	-984.4065271	-4.2245668	-988.4207455
conformer_21	-990.1155063	0.3016340	0.2346440	-990.1429482	-72.0	-989.841314	-989.905292	15.4	-984.4073804	-4.2234851	-988.4206514
conformer_16	-990.1155819	0.3015860	0.2345950	-990.1429294	-71.8	-989.841343	-989.905322	15.3	-984.4073591	-4.2235220	-988.4206216
conformer_92	-990.1076128	0.3014900	0.2347790	-990.1428825	-92.6	-989.841392	-989.905091	15.9	-984.3962914	-4.2254641	-988.4192342
conformer_128	-990.1092989	0.3014100	0.2340060	-990.1428561	-88.1	-989.841446	-989.905838	13.9	-984.4003440	-4.2237256	-988.4206088
conformer_152	-990.1103244	0.3013890	0.2337770	-990.1427538	-85.1	-989.841365	-989.905965	13.6	-984.4009416	-4.2239581	-988.4205400
conformer_121	-990.1073199	0.3014720	0.2342210	-990.1427051	-92.9	-989.841233	-989.905472	14.9	-984.3960919	-4.2254293	-988.4196734

conformer_134	-990.1081147	0.3015860	0.2341980	-990.1426800	-90.8	-989.841094	-989.905470	14.9	-984.3975577	-4.2246718	-988.4195848
conformer_158	-990.1101305	0.3014050	0.2335710	-990.1425844	-85.2	-989.841179	-989.906001	13.5	-984.4008084	-4.2239800	-988.4206593
conformer_144	-990.1079593	0.3016250	0.2342030	-990.1425271	-90.8	-989.840902	-989.905312	15.3	-984.3974869	-4.2246335	-988.4194732
conformer_70	-990.1110861	0.3015880	0.2349470	-990.1424620	-82.4	-989.840874	-989.904503	17.4	-984.4011234	-4.2245364	-988.4190767
conformer_75	-990.1108496	0.3016150	0.2347770	-990.1423549	-82.7	-989.840740	-989.904566	17.3	-984.4009716	-4.2245209	-988.4192088
conformer_56	-990.1131769	0.3015670	0.2346880	-990.1421973	-76.2	-989.840630	-989.904497	17.5	-984.4042630	-4.2238169	-988.4194003
conformer_101	-990.1076933	0.3014060	0.2346180	-990.1421841	-90.6	-989.840778	-989.904554	17.3	-984.3966942	-4.2252422	-988.4187971
conformer_37	-990.1131581	0.3014170	0.2340920	-990.1421000	-76.0	-989.840683	-989.904996	16.2	-984.4053886	-4.2234061	-988.4206326
conformer_36	-990.1132561	0.3013730	0.2341410	-990.1420945	-75.7	-989.840721	-989.904941	16.3	-984.4053712	-4.2234882	-988.4205447
conformer_59	-990.1129542	0.3015840	0.2345550	-990.1420593	-76.4	-989.840475	-989.904492	17.5	-984.4041354	-4.2237902	-988.4194638
conformer_81	-990.1104605	0.3014620	0.2344650	-990.1420396	-82.9	-989.840578	-989.904563	17.3	-984.4021701	-4.2231397	-988.4194120
conformer_89	-990.1101544	0.3014560	0.2340330	-990.1418499	-83.2	-989.840394	-989.904805	16.7	-984.4019401	-4.2231476	-988.4197382
conformer_159	-990.1106803	0.3013670	0.2336600	-990.1416453	-81.3	-989.840278	-989.904973	16.2	-984.4021821	-4.2235282	-988.4200034
conformer_137	-990.1080656	0.3014060	0.2341710	-990.1415539	-87.9	-989.840148	-989.904371	17.8	-984.3976162	-4.2249143	-988.4188359
conformer_67	-990.1130294	0.3013740	0.2340540	-990.1415523	-74.9	-989.840178	-989.904486	17.5	-984.4057095	-4.2231345	-988.4203010
conformer_167	-990.1104846	0.3013820	0.2334570	-990.1414736	-81.4	-989.840092	-989.905005	16.1	-984.4020464	-4.2235382	-988.4201045
conformer_49	-990.1138772	0.3015370	0.2343850	-990.1411516	-71.6	-989.839615	-989.903755	19.4	-984.4056521	-4.2235279	-988.4190573
conformer_85	-990.1078214	0.3016560	0.2359160	-990.1410160	-87.2	-989.839360	-989.902088	23.8	-984.3917390	-4.2296109	-988.4156165
conformer_54	-990.1136549	0.3015600	0.2342920	-990.1409983	-71.8	-989.839438	-989.903694	19.6	-984.4055256	-4.2235039	-988.4190688
conformer_2	-990.1152727	0.3017790	0.2351800	-990.1409531	-67.4	-989.839174	-989.902761	22.0	-984.4064470	-4.2238155	-988.4177508
conformer_178	-990.1075390	0.3015720	0.2356390	-990.1409382	-87.7	-989.839366	-989.902287	23.3	-984.3906288	-4.2308962	-988.4162733
conformer_1	-990.1153402	0.3017450	0.2352530	-990.1409187	-67.2	-989.839174	-989.902654	22.3	-984.4064351	-4.2238398	-988.4175884
conformer_193	-990.1079399	0.3014120	0.2347910	-990.1393051	-82.3	-989.837893	-989.901502	25.3	-984.3959091	-4.2271397	-988.4166110
conformer_161	-990.1063032	0.3014210	0.2356170	-990.1378944	-82.9	-989.836473	-989.899265	31.2	-984.3910168	-4.2298125	-988.4137915
conformer_189	-990.1027248	0.3015650	0.2341610	-990.1373023	-90.8	-989.835737	-989.900129	28.9	-984.3880212	-4.2292092	-988.4146348

[a]: Single-point calculation in aqueous phase with SMD model.

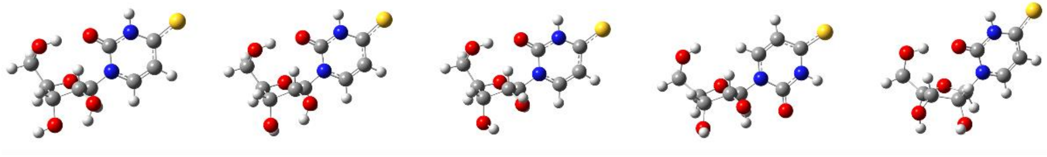

Figure S40. B3LYP-D3/def2-TZVPP optimized geometries of conformers for 4-thiouridine (**1rb4thU**).

Table S38. Conformers of gas-phase optimized 4-thiouridine (**1rb4thU**) at the B3LYP-D3/def2-TZVPP level of theory followed by aqueous phase single-point calculation. The columns display total energy without zero-point correction (E_{Tot}), thermal correction to enthalpy (δH), Gibbs free energy (δG), total energy without zero-point correction ($E_{Tot,W}$), Gibbs free energy ($G_{298,W}$) in water (W), total single-point energy (E_{CBS}) calculated at DLPNO-CCSD(T)/CBS level of theory, and their corresponding free energy $G_{CBS,W}$. $G_{298,W}$ and $G_{CBS,W}$ have been corrected to the standard state of 1 mol/L by addition of +7.908 kJ/mol. ΔG_{Solv} represents the Gibbs free energy of solvation. The data are arranged in the ascending numeric order of $E_{Tot,W}$. $\Delta G_{298,W}$ represents the respective energy difference to the lowest structure. Only conformers within the 24 kJ/mol (6 kcal/mol) energy window above the lowest in CREST are included in initial conformer sampling. Duplicates of the same structure are excluded. The overall optimum is marked bold.

1rb4thU No.	B3LYP-D3/def2-TZVPP E_{Tot} (Hartree)	δH (Hartree)	δG (Hartree)	SMD(H_2O)/B3LYP-D3/def2-TZVPP[a] $E_{Tot,W}$ (Hartree)	ΔG_{Solv} (kJ/mol)	$H_{298,W}$ (Hartree)	$G_{298,W}$ (Hartree)	$\Delta G_{298,W}$ (kJ/mol)	DLPNO-CCSD(T)/CBS $E_{CBS,HF}$ (Hartree)	$E_{CBS,C}$ (Hartree)	$G_{CBS,W}$ (Hartree)
conformer_18	-1234.407324	0.241368	0.182161	-1234.440501	-87.1	-1234.199133	-1234.255328	5.6	-1228.945926	-3.756954	-1232.550884
conformer_5	-1234.406673	0.241259	0.181818	-1234.440438	-88.6	-1234.199179	-1234.255608	4.9	-1228.945225	-3.756755	-1232.550915
conformer_8	-1234.404403	0.241229	0.181767	-1234.440388	-94.5	-1234.199159	-1234.255609	4.9	-1228.941051	-3.758138	-1232.550395
conformer_38	-1234.403474	0.241231	0.181190	-1234.439258	-93.9	-1234.198027	-1234.255056	6.3	-1228.942012	-3.756536	-1232.550129
**conformer_34**	**-1234.400951**	**0.241234**	**0.178759**	**-1234.439243**	**-100.5**	**-1234.198009**	**-1234.257472**	**0.0**	**-1228.942400**	**-3.754214**	**-1232.553135**
conformer_19	-1234.405427	0.241211	0.180841	-1234.439135	-88.5	-1234.197924	-1234.255282	5.8	-1228.946233	-3.754522	-1232.550610
conformer_23	-1234.404055	0.241182	0.179636	-1234.438883	-91.4	-1234.197701	-1234.256235	3.2	-1228.944709	-3.754638	-1232.551527
conformer_67	-1234.398583	0.241043	0.179253	-1234.438526	-104.9	-1234.197483	-1234.256261	3.2	-1228.938400	-3.755683	-1232.551761
conformer_7	-1234.407212	0.241043	0.181070	-1234.438483	-82.1	-1234.197440	-1234.254401	8.1	-1228.944174	-3.758469	-1232.549832
conformer_112	-1234.398839	0.240877	0.179726	-1234.438463	-104.0	-1234.197586	-1234.255725	4.6	-1228.939309	-3.755377	-1232.551572
conformer_12	-1234.405723	0.241200	0.181438	-1234.438416	-85.8	-1234.197216	-1234.253966	9.2	-1228.944247	-3.756909	-1232.549399
conformer_3	-1234.406160	0.241057	0.181328	-1234.438291	-84.4	-1234.197234	-1234.253951	9.2	-1228.944669	-3.757300	-1232.549759
conformer_75	-1234.401683	0.241164	0.180102	-1234.438211	-95.9	-1234.197047	-1234.255097	6.2	-1228.940776	-3.756290	-1232.550480
conformer_36	-1234.398876	0.240994	0.180036	-1234.438170	-103.2	-1234.197176	-1234.255122	6.2	-1228.937824	-3.755739	-1232.549808
conformer_21	-1234.405916	0.241188	0.180724	-1234.438139	-84.6	-1234.196951	-1234.254403	8.1	-1228.947634	-3.754004	-1232.550125
conformer_6	-1234.407120	0.241018	0.180665	-1234.438091	-81.3	-1234.197073	-1234.254414	8.0	-1228.945156	-3.757224	-1232.549675
conformer_60	-1234.397448	0.240897	0.179149	-1234.438072	-106.7	-1234.197175	-1234.255911	4.1	-1228.937294	-3.755478	-1232.551236
conformer_77	-1234.397710	0.240885	0.179502	-1234.438066	-106.0	-1234.197181	-1234.255552	5.0	-1228.936332	-3.756681	-1232.550855
conformer_81	-1234.399349	0.240885	0.179697	-1234.438017	-101.5	-1234.197132	-1234.255308	5.7	-1228.940329	-3.754179	-1232.550467
conformer_64	-1234.400023	0.240965	0.180137	-1234.437944	-99.6	-1234.196979	-1234.254795	7.0	-1228.938222	-3.756671	-1232.549665
conformer_31	-1234.400573	0.241098	0.180525	-1234.437815	-97.8	-1234.196717	-1234.254278	8.4	-1228.940393	-3.755216	-1232.549314
conformer_27	-1234.405384	0.241001	0.181383	-1234.437786	-85.1	-1234.196785	-1234.253391	10.7	-1228.944335	-3.757326	-1232.549668
conformer_30	-1234.401252	0.241218	0.180058	-1234.437707	-95.7	-1234.196489	-1234.254637	7.4	-1228.942663	-3.754432	-1232.550479
conformer_71	-1234.398526	0.240931	0.179232	-1234.437646	-102.7	-1234.196715	-1234.255402	5.4	-1228.940484	-3.753892	-1232.551252
conformer_13	-1234.405100	0.240949	0.180413	-1234.437606	-85.3	-1234.196657	-1234.254181	8.6	-1228.943484	-3.757050	-1232.549615
conformer_80	-1234.398039	0.241027	0.178527	-1234.437579	-103.8	-1234.196552	-1234.256040	3.8	-1228.940019	-3.753729	-1232.551749
conformer_56	-1234.401341	0.240964	0.179925	-1234.437532	-95.0	-1234.196568	-1234.254595	7.6	-1228.943268	-3.753641	-1232.550163
conformer_1	-1234.408726	0.241126	0.181353	-1234.437510	-75.6	-1234.196384	-1234.253145	11.4	-1228.947598	-3.756959	-1232.548976
conformer_43	-1234.399445	0.241108	0.179957	-1234.437375	-99.6	-1234.196267	-1234.254406	8.0	-1228.937046	-3.757184	-1232.549191
conformer_54	-1234.395874	0.240864	0.178880	-1234.437234	-108.6	-1234.196370	-1234.255342	5.6	-1228.936660	-3.754429	-1232.550558

conformer_97	-1234.397410	0.241015	0.179631	-1234.437100	-104.2	-1234.196085	-1234.254457	7.9	-1228.938938	-3.754279	-1232.550264
conformer_40	-1234.400927	0.240983	0.180789	-1234.437084	-94.9	-1234.196101	-1234.253283	11.0	-1228.941005	-3.755822	-1232.549183
conformer_94	-1234.394768	0.240683	0.178160	-1234.437058	-111.0	-1234.196375	-1234.255886	4.2	-1228.935571	-3.754468	-1232.551158
conformer_46	-1234.399797	0.241042	0.181024	-1234.436946	-97.5	-1234.195904	-1234.252910	12.0	-1228.937740	-3.757498	-1232.548351
conformer_62	-1234.400256	0.240903	0.179613	-1234.436940	-96.3	-1234.196037	-1234.254315	8.3	-1228.939547	-3.755973	-1232.549578
conformer_15	-1234.403872	0.241045	0.180581	-1234.436923	-86.8	-1234.195878	-1234.253330	10.9	-1228.943041	-3.756339	-1232.548838
conformer_4	-1234.404786	0.241142	0.180683	-1234.436890	-84.3	-1234.195748	-1234.253195	11.2	-1228.944434	-3.755977	-1232.548820
conformer_26	-1234.402388	0.241074	0.179911	-1234.436775	-90.3	-1234.195701	-1234.253852	9.5	-1228.943623	-3.754967	-1232.550054
conformer_109	-1234.396227	0.240670	0.177593	-1234.436539	-105.8	-1234.195869	-1234.255934	4.0	-1228.939050	-3.752898	-1232.551655
conformer_69	-1234.397801	0.240805	0.178389	-1234.436471	-101.5	-1234.195666	-1234.255070	6.3	-1228.940968	-3.752743	-1232.550981
conformer_86	-1234.398825	0.240860	0.179438	-1234.436152	-98.0	-1234.195292	-1234.253702	9.9	-1228.940246	-3.754703	-1232.549827
conformer_101	-1234.395183	0.240886	0.179240	-1234.436122	-107.5	-1234.195236	-1234.253870	9.5	-1228.933774	-3.756833	-1232.549295
conformer_98	-1234.395781	0.240764	0.178442	-1234.436044	-105.7	-1234.195280	-1234.254590	7.6	-1228.937233	-3.754057	-1232.550099
conformer_33	-1234.400195	0.241266	0.180435	-1234.436029	-94.1	-1234.194763	-1234.252582	12.8	-1228.940958	-3.754846	-1232.548191
conformer_58	-1234.397235	0.240961	0.179852	-1234.435956	-101.7	-1234.194995	-1234.253092	11.5	-1228.935450	-3.756824	-1232.548130
conformer_24	-1234.404222	0.241019	0.180183	-1234.435797	-82.9	-1234.194778	-1234.252602	12.8	-1228.944465	-3.755727	-1232.548572
conformer_85	-1234.398244	0.240798	0.178352	-1234.435550	-97.9	-1234.194752	-1234.254186	8.6	-1228.942068	-3.752291	-1232.550301
conformer_51	-1234.399021	0.241021	0.180142	-1234.435523	-95.8	-1234.194502	-1234.252369	13.4	-1228.940576	-3.754325	-1232.548249
conformer_53	-1234.396739	0.241057	0.180372	-1234.435422	-101.6	-1234.194365	-1234.252038	14.3	-1228.935954	-3.756087	-1232.547341
conformer_10	-1234.404528	0.241137	0.180148	-1234.435418	-81.1	-1234.194281	-1234.252258	13.7	-1228.945666	-3.755150	-1232.548545
conformer_9	-1234.404148	0.241115	0.180206	-1234.435224	-81.6	-1234.194109	-1234.252006	14.4	-1228.946419	-3.754168	-1232.548445
conformer_72	-1234.399617	0.240885	0.178330	-1234.434748	-92.2	-1234.193863	-1234.253406	10.7	-1228.940686	-3.754775	-1232.549250
conformer_41	-1234.399998	0.241151	0.180518	-1234.434598	-90.8	-1234.193447	-1234.251068	16.8	-1228.940484	-3.755326	-1232.546879
conformer_39	-1234.399978	0.241090	0.180252	-1234.434502	-90.6	-1234.193412	-1234.251238	16.4	-1228.942307	-3.753718	-1232.547285
conformer_20	-1234.402002	0.240964	0.179926	-1234.434422	-85.1	-1234.193458	-1234.251484	15.7	-1228.944744	-3.753970	-1232.548197
conformer_28	-1234.402292	0.241102	0.180156	-1234.434388	-84.3	-1234.193286	-1234.251220	16.4	-1228.943816	-3.754640	-1232.547384
conformer_73	-1234.396246	0.240977	0.180327	-1234.434252	-99.8	-1234.193275	-1234.250913	17.2	-1228.935455	-3.756198	-1232.546320
conformer_70	-1234.397303	0.240937	0.179218	-1234.433931	-96.2	-1234.192994	-1234.251701	15.2	-1228.937534	-3.755409	-1232.547342
conformer_42	-1234.402020	0.240931	0.179886	-1234.433831	-83.5	-1234.192900	-1234.250933	17.2	-1228.945219	-3.753673	-1232.547805
conformer_89	-1234.400075	0.240877	0.178179	-1234.433686	-88.2	-1234.192809	-1234.252495	13.1	-1228.942015	-3.754376	-1232.548810
conformer_25	-1234.403143	0.241180	0.180250	-1234.433398	-79.4	-1234.192218	-1234.250136	19.3	-1228.945375	-3.754267	-1232.546636
conformer_2	-1234.403962	0.241328	0.181043	-1234.433340	-77.1	-1234.192012	-1234.249285	21.5	-1228.945527	-3.754499	-1232.545349
conformer_108	-1234.393673	0.240884	0.177391	-1234.433223	-103.8	-1234.192339	-1234.252820	12.2	-1228.935920	-3.753508	-1232.548574
conformer_105	-1234.396284	0.241138	0.181311	-1234.433088	-96.6	-1234.191950	-1234.248765	22.9	-1228.930083	-3.761360	-1232.543925
conformer_52	-1234.396483	0.241159	0.181668	-1234.432842	-95.5	-1234.191683	-1234.248162	24.4	-1228.930865	-3.760429	-1232.542973
conformer_78	-1234.398160	0.240988	0.180437	-1234.431664	-88.0	-1234.190676	-1234.248215	24.3	-1228.936890	-3.757643	-1232.544589
conformer_106	-1234.396213	0.240873	0.179337	-1234.430780	-90.8	-1234.189907	-1234.248431	23.7	-1228.934521	-3.757718	-1232.544457
conformer_68	-1234.396247	0.241042	0.181226	-1234.430643	-90.3	-1234.189601	-1234.246405	29.1	-1228.931765	-3.760230	-1232.542153
conformer_107	-1234.392075	0.240995	0.179880	-1234.430140	-99.9	-1234.189145	-1234.247248	26.8	-1228.928415	-3.759610	-1232.543199
conformer_95	-1234.391921	0.241476	0.181851	-1234.425973	-89.4	-1234.184497	-1234.241110	43.0	-1228.926010	-3.761630	-1232.536829

[a]: Single-point calculation in aqueous phase with SMD model.

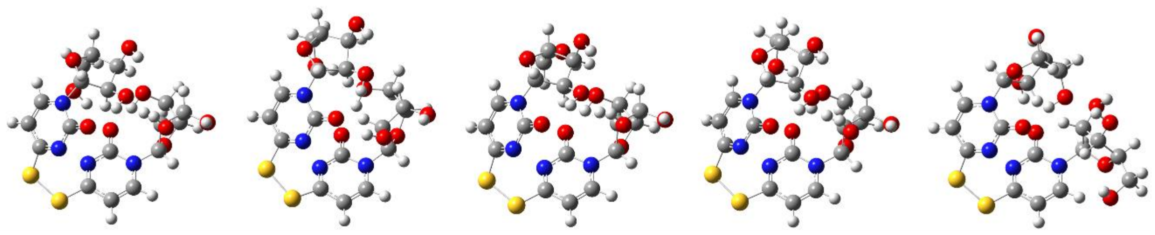

conformer_5 conformer_7 conformer_8 conformer_10 conformer_29

Figure S41. B3LYP-D3/def2-TZVPP optimized geometries of conformers for 4-thiouridine dimer (**1rb4thU_dimer**).

Table S39. Conformers of gas-phase optimized 4-thiouridine dimer (**1rb4thU_dimer**) at the B3LYP-D3/def2-TZVPP level of theory followed by aqueous phase single-point calculation. The columns display total energy without zero-point correction (E_{Tot}), thermal correction to enthalpy (δH), Gibbs free energy (δG), total energy without zero-point correction ($E_{Tot,W}$), Gibbs free energy ($G_{298,W}$) in water (W), total single-point energy (E_{CBS}) calculated at DLPNO-CCSD(T)/CBS level of theory, and their corresponding free energy $G_{CBS,W}$. $G_{298,W}$ and $G_{CBS,W}$ have been corrected to the standard state of 1 mol/L by addition of +7.908 kJ/mol. ΔG_{Solv} represents the Gibbs free energy of solvation. The data are arranged in the ascending numeric order of $E_{Tot,W}$. $\Delta G_{298,W}$ represents the respective energy difference to the lowest structure. Only conformers within the 24 kJ/mol (6 kcal/mol) energy window above the lowest in CREST are included in initial conformer sampling. Duplicates of the same structure are excluded. The overall optimum is marked bold.

1rb4thU_dimer No.	B3LYP-D3/def2-TZVPP E_{Tot} (Hartree)	δH (Hartree)	δG (Hartree)	SMD(H_2O)/B3LYP-D3/def2-TZVPP[a] $E_{Tot,W}$ (Hartree)	ΔG_{Solv} (kJ/mol)	$H_{298,W}$ (Hartree)	$G_{298,W}$ (Hartree)	$\Delta G_{298,W}$ (kJ/mol)	DLPNO-CCSD(T)/CBS $E_{CBS,HF}$ (Hartree)	$E_{CBS,C}$ (Hartree)	$G_{CBS,W}$ (Hartree)
conformer_5	-2467.604289	0.459548	0.369779	-2467.653235	-128.5	-2467.193687	-2467.280444	7.5	-2456.679038	-7.519464	-2463.874657
conformer_7	-2467.604109	0.459109	0.369192	-2467.653225	-129.0	-2467.194116	-2467.281021	6.0	-2456.678848	-7.519271	-2463.875032
**conformer_8**	**-2467.604035**	**0.459416**	**0.368826**	**-2467.655144**	**-134.2**	**-2467.195728**	**-2467.283306**	**0.0**	**-2456.681382**	**-7.517645**	**-2463.878299**
conformer_10	-2467.602774	0.459049	0.367842	-2467.651316	-127.4	-2467.192267	-2467.280462	7.5	-2456.681478	-7.516348	-2463.875515
conformer_29	-2467.602335	0.459231	0.367049	-2467.652630	-132.0	-2467.193399	-2467.282569	1.9	-2456.680866	-7.515477	-2463.876577
conformer_34	-2467.601902	0.459217	0.367015	-2467.651025	-129.0	-2467.191808	-2467.280998	6.1	-2456.681404	-7.515108	-2463.875608
conformer_4	-2467.601151	0.459290	0.367392	-2467.651139	-131.2	-2467.191849	-2467.280735	6.8	-2456.681722	-7.513477	-2463.874783
conformer_6	-2467.601133	0.459380	0.367908	-2467.652027	-133.6	-2467.192647	-2467.281107	5.8	-2456.680509	-7.514130	-2463.874612
conformer_1	-2467.601024	0.459300	0.367031	-2467.652780	-135.9	-2467.193480	-2467.282737	1.5	-2456.680467	-7.514495	-2463.876675
conformer_28	-2467.601006	0.459326	0.368763	-2467.651752	-133.2	-2467.192426	-2467.279977	8.7	-2456.676279	-7.519115	-2463.874366
conformer_30	-2467.599633	0.459310	0.366920	-2467.651913	-137.3	-2467.192603	-2467.281981	3.5	-2456.679293	-7.514699	-2463.876340
conformer_43	-2467.599633	0.459310	0.366920	-2467.651913	-137.3	-2467.192603	-2467.281981	3.5	-2456.679294	-7.514699	-2463.876340
conformer_21	-2467.599523	0.459372	0.367270	-2467.649693	-131.7	-2467.190321	-2467.279411	10.2	-2456.678351	-7.515376	-2463.873614
conformer_12	-2467.599090	0.459028	0.367391	-2467.647438	-126.9	-2467.188410	-2467.277035	16.5	-2456.676724	-7.516623	-2463.871292
conformer_81	-2467.599090	0.459028	0.367391	-2467.647438	-126.9	-2467.188410	-2467.277035	16.5	-2456.676724	-7.516623	-2463.871292
conformer_3	-2467.598798	0.459072	0.365974	-2467.651959	-139.6	-2467.192887	-2467.282973	0.9	-2456.680622	-7.512432	-2463.877230
conformer_2	-2467.598495	0.459180	0.366229	-2467.649643	-134.3	-2467.190463	-2467.280402	7.6	-2456.679236	-7.513240	-2463.874383
conformer_78	-2467.597974	0.459285	0.368486	-2467.653214	-145.0	-2467.193929	-2467.281716	4.2	-2456.675558	-7.517490	-2463.876788
conformer_15	-2467.597954	0.459418	0.367939	-2467.648582	-132.9	-2467.189164	-2467.277631	14.9	-2456.674921	-7.517632	-2463.872229
conformer_45	-2467.597812	0.459591	0.368680	-2467.650935	-139.5	-2467.191344	-2467.279243	10.7	-2456.676396	-7.516958	-2463.874784
conformer_16	-2467.597552	0.459325	0.368445	-2467.646255	-127.9	-2467.186930	-2467.274798	22.3	-2456.671623	-7.519597	-2463.868467
conformer_70	-2467.597364	0.459004	0.365783	-2467.651551	-142.3	-2467.192547	-2467.282756	1.4	-2456.677676	-7.514057	-2463.877125
conformer_90	-2467.597117	0.459417	0.369518	-2467.651116	-141.8	-2467.191699	-2467.278586	12.4	-2456.671565	-7.519509	-2463.872542
conformer_26	-2467.596928	0.458913	0.367351	-2467.649439	-137.9	-2467.190526	-2467.279076	11.1	-2456.677575	-7.513638	-2463.873361
conformer_17	-2467.596808	0.459358	0.367467	-2467.648235	-135.0	-2467.188877	-2467.277756	14.6	-2456.676413	-7.515628	-2463.872989
conformer_93	-2467.596486	0.459590	0.368099	-2467.649165	-138.3	-2467.189575	-2467.278054	13.8	-2456.673442	-7.516830	-2463.871839
conformer_54	-2467.596412	0.459251	0.367127	-2467.648596	-137.0	-2467.189345	-2467.278457	12.7	-2456.676338	-7.515158	-2463.873539

conformer_47	-2467.595961	0.459399	0.366843	-2467.645306	-129.6	-2467.185907	-2467.275451	20.6	-2456.680042	-7.511171	-2463.870704
conformer_24	-2467.595808	0.458562	0.365532	-2467.650442	-143.4	-2467.191880	-2467.281898	3.7	-2456.679569	-7.511062	-2463.876720
conformer_22	-2467.595657	0.458875	0.365313	-2467.651206	-145.8	-2467.192331	-2467.282881	1.1	-2456.677730	-7.512270	-2463.877224
conformer_55	-2467.595544	0.458845	0.365394	-2467.649271	-141.1	-2467.190426	-2467.280865	6.4	-2456.681550	-7.509550	-2463.876420
conformer_52	-2467.595527	0.458897	0.365548	-2467.650325	-143.9	-2467.191428	-2467.281765	4.0	-2456.680436	-7.510244	-2463.876918
conformer_73	-2467.595305	0.458830	0.365192	-2467.650600	-145.2	-2467.191770	-2467.282396	2.4	-2456.677997	-7.511987	-2463.877075
conformer_25	-2467.594663	0.458609	0.364375	-2467.641843	-123.9	-2467.183234	-2467.274456	23.2	-2456.681591	-7.509830	-2463.871215
conformer_18	-2467.594299	0.458602	0.364332	-2467.642846	-127.5	-2467.184244	-2467.275502	20.5	-2456.680409	-7.510239	-2463.871851
conformer_89	-2467.594286	0.459476	0.367465	-2467.648716	-142.9	-2467.189240	-2467.278239	13.3	-2456.672077	-7.516367	-2463.872396
conformer_98	-2467.594132	0.458933	0.364751	-2467.650208	-147.2	-2467.191275	-2467.282445	2.3	-2456.685965	-7.504814	-2463.879092
conformer_65	-2467.594099	0.459300	0.366937	-2467.648669	-143.3	-2467.189369	-2467.278720	12.0	-2456.671203	-7.517738	-2463.873562
conformer_75	-2467.593950	0.459576	0.367848	-2467.646891	-139.0	-2467.187315	-2467.276031	19.1	-2456.671160	-7.516717	-2463.869958
conformer_33	-2467.593937	0.458811	0.364830	-2467.640932	-123.4	-2467.182121	-2467.273090	26.8	-2456.675721	-7.513967	-2463.868842
conformer_48	-2467.593893	0.459021	0.365866	-2467.650654	-149.0	-2467.191633	-2467.281776	4.0	-2456.678523	-7.510557	-2463.876963
conformer_72	-2467.593535	0.459156	0.366451	-2467.647404	-141.4	-2467.188248	-2467.277941	14.1	-2456.677058	-7.511559	-2463.873024
conformer_84	-2467.593435	0.459208	0.367748	-2467.645058	-135.5	-2467.185850	-2467.274298	23.7	-2456.669672	-7.517576	-2463.868111
conformer_59	-2467.593288	0.458739	0.365720	-2467.649863	-148.5	-2467.191124	-2467.281131	5.7	-2456.676638	-7.512485	-2463.876965
conformer_37	-2467.592951	0.458882	0.365288	-2467.647694	-143.7	-2467.188812	-2467.279394	10.3	-2456.679314	-7.509046	-2463.874803
conformer_67	-2467.592816	0.458664	0.364602	-2467.649659	-149.2	-2467.190995	-2467.282045	3.3	-2456.679934	-7.508525	-2463.877688
conformer_41	-2467.592138	0.458500	0.363812	-2467.642904	-133.3	-2467.184404	-2467.276080	19.0	-2456.678018	-7.510411	-2463.872371
conformer_91	-2467.592032	0.458716	0.364113	-2467.648113	-147.2	-2467.189397	-2467.280988	6.1	-2456.674876	-7.511619	-2463.875451
conformer_86	-2467.591974	0.458875	0.368517	-2467.648512	-148.4	-2467.189637	-2467.276983	16.6	-2456.664808	-7.520857	-2463.870674
conformer_64	-2467.591752	0.459152	0.366091	-2467.644244	-137.8	-2467.185092	-2467.275141	21.4	-2456.671478	-7.515540	-2463.870407
conformer_97	-2467.591632	0.458514	0.365149	-2467.646213	-143.3	-2467.187699	-2467.278052	13.8	-2456.677057	-7.511101	-2463.874579
conformer_27	-2467.591496	0.458434	0.363137	-2467.642389	-133.6	-2467.183955	-2467.276240	18.6	-2456.679224	-7.509046	-2463.873012
conformer_14	-2467.591451	0.458579	0.363970	-2467.640164	-127.9	-2467.181585	-2467.273182	26.6	-2456.678842	-7.509270	-2463.869842
conformer_77	-2467.591263	0.459088	0.366111	-2467.640784	-130.0	-2467.181696	-2467.271661	30.6	-2456.672001	-7.514317	-2463.866715
conformer_94	-2467.591124	0.459147	0.367328	-2467.642190	-134.1	-2467.183043	-2467.271850	30.1	-2456.668252	-7.518722	-2463.867701
conformer_53	-2467.590905	0.458465	0.363841	-2467.643764	-138.8	-2467.185299	-2467.276911	16.8	-2456.675823	-7.510834	-2463.872663
conformer_69	-2467.590731	0.458961	0.367485	-2467.643135	-137.6	-2467.184174	-2467.272638	28.0	-2456.667202	-7.518115	-2463.867224
conformer_61	-2467.590563	0.459390	0.365215	-2467.644002	-140.3	-2467.184612	-2467.275775	19.8	-2456.677221	-7.509997	-2463.872430
conformer_95	-2467.590339	0.458711	0.366204	-2467.649972	-156.6	-2467.191261	-2467.280756	6.7	-2456.672456	-7.513255	-2463.876128
conformer_58	-2467.590197	0.458466	0.364178	-2467.644138	-141.6	-2467.185672	-2467.276948	16.7	-2456.671873	-7.513695	-2463.872319
conformer_100	-2467.590155	0.459303	0.366225	-2467.645346	-144.9	-2467.186043	-2467.276109	18.9	-2456.675078	-7.511276	-2463.872309
conformer_44	-2467.589562	0.459425	0.365642	-2467.642119	-138.0	-2467.182694	-2467.273465	25.8	-2456.676074	-7.509935	-2463.869912
conformer_71	-2467.589537	0.459346	0.366578	-2467.642917	-140.1	-2467.183571	-2467.273327	26.2	-2456.667501	-7.515425	-2463.866716
conformer_35	-2467.589378	0.458303	0.362503	-2467.641557	-137.0	-2467.183254	-2467.276042	19.1	-2456.677749	-7.508465	-2463.872878
conformer_36	-2467.589097	0.458850	0.366823	-2467.642268	-139.6	-2467.183418	-2467.272433	28.5	-2456.666272	-7.518139	-2463.867747
conformer_57	-2467.588461	0.458195	0.363342	-2467.639669	-134.4	-2467.181474	-2467.273315	26.2	-2456.672620	-7.512545	-2463.870020
conformer_46	-2467.588185	0.458330	0.362681	-2467.642362	-142.2	-2467.184032	-2467.276669	17.4	-2456.673210	-7.510860	-2463.872554
conformer_62	-2467.587610	0.458555	0.363556	-2467.640406	-138.6	-2467.181851	-2467.273838	24.9	-2456.673412	-7.511179	-2463.870818
conformer_60	-2467.587604	0.458786	0.368183	-2467.643919	-147.9	-2467.185133	-2467.272724	27.8	-2456.658690	-7.522818	-2463.866627
conformer_92	-2467.587204	0.458193	0.362043	-2467.642319	-144.7	-2467.184126	-2467.277264	15.9	-2456.671605	-7.511386	-2463.873050
conformer_32	-2467.587114	0.458611	0.362763	-2467.638593	-135.2	-2467.179982	-2467.272818	27.5	-2456.675070	-7.509085	-2463.869859
conformer_85	-2467.586863	0.459079	0.365709	-2467.639618	-138.5	-2467.180539	-2467.270897	32.6	-2456.666644	-7.513807	-2463.864487
conformer_68	-2467.586060	0.458282	0.363051	-2467.638812	-138.5	-2467.180530	-2467.272749	27.7	-2456.671270	-7.511763	-2463.869721
conformer_96	-2467.584492	0.458628	0.362990	-2467.638260	-141.2	-2467.179632	-2467.272258	29.0	-2456.672969	-7.508044	-2463.868780
conformer_87	-2467.583727	0.459059	0.365187	-2467.640332	-148.6	-2467.181273	-2467.272133	29.3	-2456.666239	-7.512750	-2463.867395
conformer_66	-2467.583575	0.458681	0.363112	-2467.635895	-137.4	-2467.177214	-2467.269771	35.5	-2456.673184	-7.507705	-2463.867086
conformer_83	-2467.582818	0.458967	0.366934	-2467.638971	-147.4	-2467.180004	-2467.269025	37.5	-2456.655243	-7.521340	-2463.862790
conformer_88	-2467.581236	0.458646	0.361526	-2467.635741	-143.1	-2467.177095	-2467.271203	31.8	-2456.671667	-7.507644	-2463.869278

[a]: Single-point calculation in aqueous phase with SMD model.

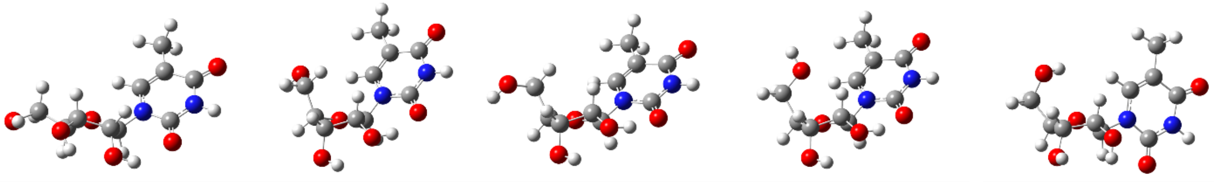

Figure S42. B3LYP-D3/def2-TZVPP optimized geometries of conformers for 5-methyluridine (**1rb5mU**).

Table S40. Conformers of gas-phase optimized 5-methyluridine (**1rb5mU**) at the B3LYP-D3/def2-TZVPP level of theory followed by aqueous phase single-point calculation. The columns display total energy without zero-point correction (E_{Tot}), thermal correction to enthalpy (δH), Gibbs free energy (δG), total energy without zero-point correction ($E_{Tot,W}$), Gibbs free energy ($G_{298,W}$) in water (W), total single-point energy (E_{CBS}) calculated at DLPNO-CCSD(T)/CBS level of theory, and their corresponding free energy $G_{CBS,W}$. $G_{298,W}$ and $G_{CBS,W}$ have been corrected to the standard state of 1 mol/L by addition of +7.908 kJ/mol. ΔG_{Solv} represents the Gibbs free energy of solvation. The data are arranged in the ascending numeric order of $E_{Tot,W}$. $\Delta G_{298,W}$ represents the respective energy difference to the lowest structure. Only conformers within the 24 kJ/mol (6 kcal/mol) energy window above the lowest in CREST are included in initial conformer sampling. Duplicates of the same structure are excluded. The overall optimum is marked bold.

	B3LYP-D3/def2-TZVPP			SMD(H_2O)/B3LYP-D3/def2-TZVPP[a]					DLPNO-CCSD(T)/CBS		
1rb5mU No.	E_{Tot} (Hartree)	δH (Hartree)	δG (Hartree)	$E_{Tot,W}$ (Hartree)	ΔG_{Solv} (kJ/mol)	$H_{298,W}$ (Hartree)	$G_{298,W}$ (Hartree)	$\Delta G_{298,W}$ (kJ/mol)	$E_{CBS,HF}$ (Hartree)	$E_{CBS,C}$ (Hartree)	$G_{CBS,W}$ (Hartree)
conformer_18	-950.793150	0.272517	0.210993	-950.826731	-88.2	-950.554214	-950.615738	4.9	-945.365246	-4.014791	-949.199613
conformer_6	-950.792416	0.272403	0.210632	-950.826572	-89.7	-950.554169	-950.615940	4.4	-945.364474	-4.014643	-949.199629
conformer_11	-950.790291	0.272357	0.210514	-950.826560	-95.2	-950.554203	-950.616046	4.1	-945.360529	-4.015951	-949.199223
**conformer_36**	**-950.786646**	**0.272385**	**0.207891**	**-950.825507**	**-102.0**	**-950.553122**	**-950.617616**	**0**	**-945.361490**	**-4.012247**	**-949.201695**
conformer_33	-950.789342	0.272419	0.210172	-950.825419	-94.7	-950.553000	-950.615247	6.2	-945.361485	-4.014397	-949.198775
conformer_70	-950.784326	0.272128	0.207981	-950.825097	-107.0	-950.552969	-950.617116	1.3	-945.357585	-4.013617	-949.200980
conformer_21	-950.790541	0.272266	0.209355	-950.824952	-90.3	-950.552686	-950.615597	5.3	-945.364367	-4.012928	-949.199339
conformer_19	-950.789543	0.272442	0.209082	-950.824893	-92.8	-950.552451	-950.615811	4.7	-945.363066	-4.013147	-949.199470
conformer_58	-950.783303	0.272113	0.208185	-950.824607	-108.4	-950.552494	-950.616422	3.1	-945.356286	-4.013718	-949.200111
conformer_80	-950.787197	0.272199	0.208671	-950.824548	-98.1	-950.552349	-950.615877	4.6	-945.359479	-4.014580	-949.199727
conformer_7	-950.792900	0.272179	0.209762	-950.824534	-83.1	-950.552355	-950.614772	7.5	-945.363006	-4.016564	-949.198430
conformer_13	-950.791459	0.272254	0.210015	-950.824532	-86.8	-950.552278	-950.614517	8.1	-945.363602	-4.014806	-949.198453
conformer_49	-950.784175	0.272084	0.208802	-950.824384	-105.6	-950.552300	-950.615582	5.3	-945.356381	-4.013864	-949.198640
conformer_76	-950.785242	0.272056	0.207153	-950.824348	-102.7	-950.552292	-950.617195	1.1	-945.360086	-4.012395	-949.201421
conformer_100	-950.784437	0.271991	0.208524	-950.824251	-104.5	-950.552260	-950.615727	5.0	-945.358609	-4.012468	-949.199355
conformer_81	-950.784085	0.272031	0.207849	-950.824032	-104.9	-950.552001	-950.616183	3.8	-945.359462	-4.011944	-949.200491
conformer_2	-950.791712	0.272230	0.210292	-950.823992	-84.8	-950.551762	-950.613700	10.3	-945.363126	-4.015737	-949.197839
conformer_8	-950.792444	0.272125	0.209491	-950.823927	-82.7	-950.551802	-950.614436	8.3	-945.363432	-4.015528	-949.197940
conformer_12	-950.790987	0.272129	0.208964	-950.823914	-86.5	-950.551785	-950.614950	7.0	-945.362322	-4.015523	-949.198796
conformer_45	-950.785679	0.272129	0.209000	-950.823900	-100.3	-950.551771	-950.614900	7.1	-945.358837	-4.013371	-949.198417
conformer_88	-950.783785	0.272182	0.207413	-950.823854	-105.2	-950.551672	-950.616441	3.1	-945.359384	-4.011589	-949.200617
conformer_24	-950.790810	0.272231	0.209110	-950.823811	-86.6	-950.551580	-950.614701	7.7	-945.365579	-4.012373	-949.198831
conformer_72	-950.783211	0.272034	0.208100	-950.823802	-106.6	-950.551768	-950.615702	5.0	-945.355261	-4.014745	-949.199484
conformer_31	-950.786918	0.272397	0.208845	-950.823762	-96.7	-950.551365	-950.614917	7.1	-945.361941	-4.012306	-949.199234
conformer_60	-950.781512	0.272032	0.207805	-950.823660	-110.7	-950.551628	-950.615855	4.6	-945.355828	-4.012360	-949.199519
conformer_67	-950.785232	0.272060	0.208248	-950.823615	-100.8	-950.551555	-950.615367	5.9	-945.356456	-4.015038	-949.198617
conformer_91	-950.786224	0.271995	0.208376	-950.823593	-98.1	-950.551598	-950.615217	6.3	-945.361412	-4.011876	-949.199269
conformer_35	-950.785310	0.272142	0.208352	-950.823586	-100.5	-950.551444	-950.615234	6.3	-945.356221	-4.015418	-949.198551
conformer_104	-950.780465	0.271928	0.207231	-950.823505	-113.0	-950.551577	-950.616274	3.5	-945.354744	-4.012349	-949.199889
conformer_29	-950.790763	0.272170	0.210297	-950.823488	-85.9	-950.551318	-950.613191	11.6	-945.362676	-4.015672	-949.197764

conformer_101	-950.782986	0.272095	0.208000	-950.823153	-105.5	-950.551058	-950.615153	6.5	-945.358085	-4.012148	-949.199389
conformer_5	-950.793760	0.272204	0.209830	-950.823144	-77.1	-950.550940	-950.613314	11.3	-945.365625	-4.015228	-949.197394
conformer_44	-950.786554	0.272255	0.209930	-950.822940	-95.5	-950.550685	-950.613010	12.1	-945.360087	-4.013770	-949.197301
conformer_27	-950.788017	0.272248	0.208694	-950.822870	-91.5	-950.550622	-950.614176	9.0	-945.362843	-4.012822	-949.198811
conformer_39	-950.785599	0.272294	0.209981	-950.822851	-97.8	-950.550557	-950.612870	12.5	-945.357055	-4.015422	-949.196737
conformer_85	-950.783177	0.271953	0.207057	-950.822772	-104.0	-950.550819	-950.615715	5.0	-945.359837	-4.010686	-949.200050
conformer_3	-950.790295	0.272228	0.209297	-950.822647	-84.9	-950.550419	-950.613350	11.2	-945.363271	-4.014005	-949.197319
conformer_15	-950.789471	0.272131	0.209201	-950.822613	-87.0	-950.550482	-950.613412	11.0	-945.361981	-4.014387	-949.197297
conformer_115	-950.781321	0.271948	0.207468	-950.822458	-108.0	-950.550510	-950.614990	6.9	-945.356265	-4.012024	-949.198945
conformer_83	-950.784358	0.271990	0.208105	-950.822248	-99.5	-950.550258	-950.614143	9.1	-945.359331	-4.012583	-949.198686
conformer_34	-950.785937	0.272488	0.209414	-950.822134	-95.0	-950.549646	-950.612720	12.9	-945.360262	-4.012663	-949.196696
conformer_105	-950.783553	0.271971	0.207201	-950.821725	-100.2	-950.549754	-950.614524	8.1	-945.360869	-4.010220	-949.199049
conformer_23	-950.789614	0.272093	0.208660	-950.821616	-84.0	-950.549523	-950.612956	12.2	-945.363131	-4.013844	-949.197304
conformer_61	-950.782713	0.272067	0.208514	-950.821510	-101.9	-950.549443	-950.612996	12.1	-945.354207	-4.015034	-949.196512
conformer_56	-950.784263	0.272075	0.208609	-950.821393	-97.5	-950.549318	-950.612784	12.7	-945.359341	-4.012298	-949.197148
conformer_55	-950.782181	0.272136	0.208907	-950.821233	-102.5	-950.549097	-950.612326	13.9	-945.354959	-4.014048	-949.196141
conformer_9	-950.790128	0.272241	0.208908	-950.821165	-81.5	-950.548924	-950.612257	14.1	-945.364670	-4.013228	-949.197015
conformer_89	-950.785168	0.272145	0.208181	-950.821008	-94.1	-950.548863	-950.612827	12.6	-945.359685	-4.012779	-949.197111
conformer_10	-950.789780	0.272244	0.208902	-950.820809	-81.5	-950.548565	-950.611907	15.0	-945.365498	-4.012179	-949.196792
conformer_40	-950.785660	0.272284	0.209220	-950.820607	-91.8	-950.548323	-950.611387	16.4	-945.359731	-4.013190	-949.195636
conformer_74	-950.782495	0.272349	0.208813	-950.820583	-100.0	-950.548234	-950.611770	15.3	-945.356001	-4.013244	-949.195508
conformer_30	-950.787740	0.272267	0.209092	-950.820425	-85.8	-950.548158	-950.611333	16.5	-945.362855	-4.012501	-949.195937
conformer_46	-950.785193	0.272094	0.208560	-950.820418	-92.5	-950.548324	-950.611858	15.1	-945.361032	-4.011708	-949.196393
conformer_57	-950.782121	0.272162	0.209067	-950.820359	-100.4	-950.548197	-950.611292	16.6	-945.355042	-4.013940	-949.195141
conformer_20	-950.787572	0.272091	0.208614	-950.819944	-85.0	-950.547853	-950.611330	16.5	-945.363606	-4.012152	-949.196504
conformer_103	-950.780656	0.272216	0.208601	-950.819926	-103.1	-950.547710	-950.611325	16.5	-945.351498	-4.016026	-949.195181
conformer_94	-950.785525	0.272091	0.207959	-950.819876	-90.2	-950.547785	-950.611917	15.0	-945.360936	-4.012334	-949.196650
conformer_71	-950.782704	0.272050	0.208262	-950.819875	-97.6	-950.547825	-950.611613	15.8	-945.356382	-4.013470	-949.195749
conformer_42	-950.787456	0.272024	0.208451	-950.819423	-83.9	-950.547399	-950.610972	17.4	-945.363956	-4.011852	-949.196312
conformer_107	-950.782077	0.272305	0.210158	-950.819416	-98.0	-950.547111	-950.609258	21.9	-945.349646	-4.019053	-949.192869
conformer_25	-950.788503	0.272271	0.208980	-950.819385	-81.1	-950.547114	-950.610405	18.9	-945.364329	-4.012168	-949.195387
conformer_51	-950.782350	0.272323	0.210406	-950.818957	-96.1	-950.546634	-950.608551	23.8	-945.350556	-4.018041	-949.191787
conformer_1	-950.789517	0.272419	0.209666	-950.818745	-76.7	-950.546326	-950.609079	22.4	-945.364588	-4.012536	-949.193673
conformer_79	-950.780744	0.272284	0.209069	-950.818029	-97.9	-950.545745	-950.608960	22.7	-945.350158	-4.017749	-949.193111
conformer_102	-950.783022	0.272136	0.208906	-950.817726	-91.1	-950.545590	-950.608820	23.1	-945.355630	-4.015200	-949.193616
conformer_90	-950.781234	0.272150	0.209861	-950.816444	-92.4	-950.544294	-950.606583	29.0	-945.350338	-4.018099	-949.190774
conformer_114	-950.777453	0.272239	0.208645	-950.815687	-100.4	-950.543448	-950.607042	27.8	-945.346927	-4.017728	-949.191232

[a]: Single-point calculation in aqueous phase with SMD model.

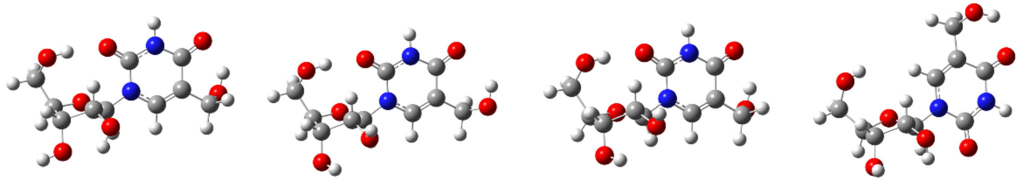

conformer_11 conformer_84 conformer_100 conformer_114

Figure S43. B3LYP-D3/def2-TZVPP optimized geometries of conformers for 5-hydroxymethyluridine (**1rb5hmU**).

Table S41. Conformers of gas-phase optimized 5-hydroxymethyluridine (**1rb5hmU**) at the B3LYP-D3/def2-TZVPP level of theory followed by aqueous phase single-point calculation. The columns display total energy without zero-point correction (E_{Tot}), thermal correction to enthalpy (δH), Gibbs free energy (δG), total energy without zero-point correction ($E_{Tot,W}$), Gibbs free energy ($G_{298,W}$) in water (W), total single-point energy (E_{CBS}) calculated at DLPNO-CCSD(T)/CBS level of theory, and their corresponding free energy $G_{CBS,W}$. $G_{298,W}$ and $G_{CBS,W}$ have been corrected to the standard state of 1 mol/L by addition of +7.908 kJ/mol. ΔG_{Solv} represents the Gibbs free energy of solvation. The data are arranged in the ascending numeric order of $E_{Tot,W}$. $\Delta G_{298,W}$ represents the respective energy difference to the lowest structure. Only conformers within the 24 kJ/mol (6 kcal/mol) energy window above the lowest in CREST are included in initial conformer sampling. Duplicates of the same structure are excluded. The overall optimum is marked bold.

1rb5hmU	B3LYP-D3/def2-TZVPP			SMD(H2O)/B3LYP-D3/def2-TZVPP[a]					DLPNO-CCSD(T)/CBS		
No.	E_{Tot} (Hartree)	δH (Hartree)	δG (Hartree)	$E_{Tot,W}$ (Hartree)	ΔG_{Solv} (kJ/mol)	$H_{298,W}$ (Hartree)	$G_{298,W}$ (Hartree)	$\Delta G_{298,W}$ (kJ/mol)	$E_{CBS,HF}$ (Hartree)	$E_{CBS,C}$ (Hartree)	$G_{CBS,W}$ (Hartree)
conformer_100	-1026.042518	0.278984	0.215252	-1026.081781	-103.1	-1025.802797	-1025.863517	7.1	-1020.256155	-4.285523	-1024.362676
conformer_84	-1026.042673	0.278974	0.215279	-1026.081630	-102.3	-1025.802656	-1025.863339	7.6	-1020.256358	-4.285478	-1024.362501
conformer_11	-1026.042060	0.278914	0.215084	-1026.081609	-103.8	-1025.802695	-1025.863513	7.1	-1020.255642	-4.285373	-1024.362469
conformer_19	-1026.039859	0.278869	0.214945	-1026.081566	-109.5	-1025.802697	-1025.863609	6.9	-1020.251631	-4.286675	-1024.362056
conformer_25	-1026.039633	0.278859	0.214937	-1026.081484	-109.9	-1025.802625	-1025.863535	7.0	-1020.251389	-4.286634	-1024.361925
conformer_13	-1026.041880	0.278870	0.214953	-1026.081449	-103.9	-1025.802579	-1025.863484	7.2	-1020.255485	-4.285347	-1024.362437
conformer_59	-1026.039101	0.278771	0.213787	-1026.080732	-109.3	-1025.801961	-1025.863933	6.0	-1020.252382	-4.285252	-1024.362467
conformer_115	-1026.036486	0.278866	0.212215	-1026.080594	-115.8	-1025.801728	-1025.865367	2.2	-1020.252880	-4.282948	-1024.364709
conformer_175	-1026.036480	0.278657	0.214063	-1026.080556	-115.7	-1025.801899	-1025.863481	7.2	-1020.250199	-4.285373	-1024.362573
conformer_234	-1026.034236	0.278671	0.214097	-1026.080527	-121.5	-1025.801856	-1025.863418	7.4	-1020.246097	-4.286693	-1024.361972
**conformer_114**	**-1026.036365**	**0.278841**	**0.211250**	**-1026.080481**	**-115.8**	**-1025.801640**	**-1025.866219**	**0**	**-1020.252731**	**-4.282912**	**-1024.365497**
conformer_128	-1026.038758	0.278897	0.214451	-1026.080479	-109.5	-1025.801582	-1025.863016	8.4	-1020.252408	-4.285097	-1024.361761
conformer_112	-1026.038874	0.278909	0.214359	-1026.080472	-109.2	-1025.801563	-1025.863101	8.2	-1020.252587	-4.285100	-1024.361915
conformer_43	-1026.041195	0.278884	0.214192	-1026.080441	-103.0	-1025.801557	-1025.863237	7.8	-1020.256117	-4.284004	-1024.362163
conformer_56	-1026.039319	0.278859	0.213055	-1026.080233	-107.4	-1025.801374	-1025.864166	5.4	-1020.254258	-4.283983	-1024.363088
conformer_203	-1026.039928	0.278675	0.214273	-1026.080150	-105.6	-1025.801475	-1025.862865	8.8	-1020.253827	-4.285126	-1024.361889
conformer_159	-1026.040110	0.278777	0.214555	-1026.080149	-105.1	-1025.801372	-1025.862582	9.5	-1020.253971	-4.285169	-1024.361613
conformer_54	-1026.040549	0.278775	0.213761	-1026.080079	-103.8	-1025.801304	-1025.863306	7.6	-1020.255756	-4.283837	-1024.362350
conformer_44	-1026.037577	0.278624	0.214135	-1026.080053	-111.5	-1025.801429	-1025.862906	8.7	-1020.249576	-4.286283	-1024.361188
conformer_89	-1026.039559	0.278647	0.213450	-1026.080039	-106.3	-1025.801392	-1025.863577	6.9	-1020.253823	-4.284713	-1024.362554
conformer_53	-1026.037306	0.278702	0.214349	-1026.080021	-112.1	-1025.801319	-1025.862660	9.3	-1020.249249	-4.286275	-1024.360877
conformer_41	-1026.039398	0.278665	0.214217	-1026.080005	-106.6	-1025.801340	-1025.862776	9.0	-1020.253215	-4.284958	-1024.361551
conformer_36	-1026.039544	0.278585	0.213938	-1026.079996	-106.2	-1025.801411	-1025.863046	8.3	-1020.253422	-4.284967	-1024.361891
conformer_49	-1026.039663	0.278867	0.212859	-1026.079986	-105.9	-1025.801119	-1025.864115	5.5	-1020.254688	-4.283879	-1024.363020
conformer_188	-1026.034292	0.278657	0.212196	-1026.079950	-119.9	-1025.801293	-1025.864742	3.9	-1020.248973	-4.284360	-1024.363783
conformer_201	-1026.033868	0.278588	0.212148	-1026.079820	-120.6	-1025.801232	-1025.864660	4.1	-1020.248672	-4.284351	-1024.363815
conformer_181	-1026.032990	0.278556	0.212342	-1026.079686	-122.6	-1025.801130	-1025.864332	5.0	-1020.247411	-4.284488	-1024.363241
conformer_111	-1026.034537	0.278707	0.213477	-1026.079599	-118.3	-1025.800892	-1025.863110	8.2	-1020.248037	-4.284796	-1024.361406
conformer_27	-1026.040980	0.278788	0.214386	-1026.079568	-101.3	-1025.800780	-1025.862170	10.6	-1020.254650	-4.285532	-1024.361372
conformer_157	-1026.040885	0.278734	0.214170	-1026.079547	-101.5	-1025.800813	-1025.862365	10.1	-1020.254586	-4.285482	-1024.361548

conformer_187	-1026.032982	0.278491	0.211823	-1026.079541	-122.2	-1025.801050	-1025.864706	4.0	-1020.247832	-4.284139	-1024.363695
conformer_82	-1026.038566	0.278708	0.214138	-1026.079514	-107.5	-1025.800806	-1025.862364	10.1	-1020.251240	-4.285565	-1024.360603
conformer_126	-1026.037412	0.278741	0.213182	-1026.079452	-110.4	-1025.800711	-1025.863258	7.8	-1020.251056	-4.285425	-1024.362326
conformer_207	-1026.037414	0.278752	0.213072	-1026.079423	-110.3	-1025.800671	-1025.863339	7.6	-1020.251014	-4.285380	-1024.362319
conformer_17	-1026.042333	0.278596	0.213779	-1026.079415	-97.4	-1025.800819	-1025.862624	9.4	-1020.253900	-4.287467	-1024.361659
conformer_81	-1026.037965	0.278581	0.213964	-1026.079357	-108.7	-1025.800776	-1025.862381	10.1	-1020.250878	-4.285734	-1024.361028
conformer_51	-1026.039602	0.278739	0.214105	-1026.079349	-104.4	-1025.800610	-1025.862232	10.5	-1020.252508	-4.285280	-1024.360418
conformer_219	-1026.040483	0.278616	0.214021	-1026.079338	-102.0	-1025.800722	-1025.862305	10.3	-1020.255299	-4.284661	-1024.361782
conformer_3	-1026.041581	0.278740	0.214750	-1026.079316	-99.1	-1025.800576	-1025.861554	12.2	-1020.254478	-4.286515	-1024.360966
conformer_153	-1026.035961	0.278648	0.213567	-1026.079306	-113.8	-1025.800658	-1025.862727	9.2	-1020.248272	-4.286118	-1024.361157
conformer_192	-1026.035345	0.278549	0.211352	-1026.079290	-115.4	-1025.800741	-1025.864926	3.4	-1020.251668	-4.283130	-1024.364379
conformer_52	-1026.041369	0.278805	0.213793	-1026.079245	-99.4	-1025.800440	-1025.862440	9.9	-1020.257278	-4.283434	-1024.361783
conformer_58	-1026.041193	0.278762	0.213665	-1026.079192	-99.8	-1025.800430	-1025.862515	9.7	-1020.257228	-4.283379	-1024.361930
conformer_113	-1026.034403	0.278678	0.213391	-1026.079182	-117.6	-1025.800504	-1025.862779	9.0	-1020.247929	-4.284809	-1024.361114
conformer_200	-1026.034049	0.278504	0.212112	-1026.079179	-118.5	-1025.800675	-1025.864055	5.7	-1020.250748	-4.282765	-1024.363519
conformer_4	-1026.041542	0.278797	0.214977	-1026.079168	-98.8	-1025.800371	-1025.861179	13.2	-1020.254446	-4.286470	-1024.360553
conformer_189	-1026.035236	0.278555	0.213009	-1026.079168	-115.3	-1025.800613	-1025.863147	8.1	-1020.247783	-4.286025	-1024.361718
conformer_96	-1026.036095	0.278768	0.213808	-1026.079149	-113.0	-1025.800381	-1025.862329	10.2	-1020.250419	-4.284364	-1024.361018
conformer_216	-1026.033237	0.278582	0.212911	-1026.079134	-120.5	-1025.800552	-1025.863211	7.9	-1020.246400	-4.285711	-1024.362085
conformer_29	-1026.040452	0.278722	0.213985	-1026.079098	-101.5	-1025.800376	-1025.862101	10.8	-1020.253372	-4.286007	-1024.361028
conformer_30	-1026.040469	0.278661	0.213446	-1026.079068	-101.3	-1025.800407	-1025.862610	9.5	-1020.253572	-4.285883	-1024.361596
conformer_241	-1026.034823	0.278576	0.212956	-1026.079062	-116.1	-1025.800486	-1025.863094	8.2	-1020.250269	-4.283360	-1024.361900
conformer_259	-1026.033452	0.278752	0.211965	-1026.079047	-119.7	-1025.800295	-1025.864070	5.6	-1020.250369	-4.282441	-1024.363427
conformer_238	-1026.037845	0.278622	0.213389	-1026.079046	-108.2	-1025.800424	-1025.862645	9.4	-1020.252712	-4.284506	-1024.362018
conformer_243	-1026.033057	0.278563	0.212975	-1026.078997	-120.6	-1025.800434	-1025.863010	8.4	-1020.246350	-4.285611	-1024.361914
conformer_211	-1026.034027	0.278510	0.212143	-1026.078981	-118.0	-1025.800471	-1025.863826	6.3	-1020.250949	-4.282579	-1024.363327
conformer_221	-1026.033739	0.278667	0.211514	-1026.078967	-118.7	-1025.800300	-1025.864441	4.7	-1020.250735	-4.282431	-1024.363867
conformer_93	-1026.036296	0.278761	0.213813	-1026.078964	-112.0	-1025.800203	-1025.862139	10.7	-1020.250649	-4.284314	-1024.360806
conformer_62	-1026.041465	0.278626	0.214491	-1026.078958	-98.4	-1025.800332	-1025.861455	12.5	-1020.254603	-4.286740	-1024.361333
conformer_12	-1026.042336	0.278666	0.214021	-1026.078953	-96.1	-1025.800287	-1025.861920	11.3	-1020.254862	-4.286369	-1024.360815
conformer_78	-1026.038353	0.278678	0.214116	-1026.078931	-106.5	-1025.800253	-1025.861803	11.6	-1020.251090	-4.285619	-1024.360160
conformer_205	-1026.036424	0.278685	0.213788	-1026.078907	-111.5	-1025.800222	-1025.862107	10.8	-1020.250295	-4.284716	-1024.360694
conformer_173	-1026.036864	0.278583	0.213063	-1026.078865	-110.3	-1025.800282	-1025.862790	9.0	-1020.253173	-4.282868	-1024.361967
conformer_97	-1026.036647	0.278880	0.213238	-1026.078861	-110.8	-1025.799981	-1025.862611	9.5	-1020.253088	-4.283077	-1024.362129
conformer_15	-1026.042308	0.278611	0.213858	-1026.078819	-95.9	-1025.800208	-1025.861949	11.2	-1020.254778	-4.286365	-1024.360784
conformer_101	-1026.036486	0.278840	0.213028	-1026.078780	-111.0	-1025.799940	-1025.862740	9.1	-1020.253057	-4.283009	-1024.362319
conformer_165	-1026.037024	0.278686	0.213402	-1026.078749	-109.5	-1025.800063	-1025.862335	10.2	-1020.253335	-4.282825	-1024.361470
conformer_1	-1026.044570	0.278691	0.214482	-1026.078686	-89.6	-1025.799995	-1025.861192	13.2	-1020.257324	-4.286671	-1024.360618
conformer_167	-1026.031341	0.278496	0.212132	-1026.078641	-124.2	-1025.800145	-1025.863497	7.1	-1020.246994	-4.283221	-1024.362371
conformer_171	-1026.031367	0.278497	0.211996	-1026.078596	-124.0	-1025.800099	-1025.863588	6.9	-1020.246995	-4.283274	-1024.362490
conformer_10	-1026.040657	0.278745	0.215692	-1026.078579	-99.6	-1025.799834	-1025.859875	16.7	-1020.251356	-4.288173	-1024.358747
conformer_279	-1026.030363	0.278388	0.211458	-1026.078533	-126.5	-1025.800145	-1025.864063	5.7	-1020.245989	-4.283238	-1024.362928
conformer_120	-1026.035320	0.278782	0.213346	-1026.078528	-113.4	-1025.799746	-1025.862170	10.6	-1020.247564	-4.286161	-1024.360576
conformer_88	-1026.040456	0.278653	0.214702	-1026.078522	-99.9	-1025.799869	-1025.860808	14.2	-1020.253858	-4.286424	-1024.360634
conformer_286	-1026.031615	0.278386	0.212430	-1026.078493	-123.1	-1025.800107	-1025.863051	8.3	-1020.244847	-4.284883	-1024.361166
conformer_125	-1026.034927	0.278696	0.213066	-1026.078484	-114.4	-1025.799788	-1025.862406	10.0	-1020.247178	-4.286217	-1024.360874
conformer_298	-1026.030202	0.278358	0.211327	-1026.078416	-126.6	-1025.800058	-1025.864077	5.6	-1020.245877	-4.283220	-1024.362973
conformer_2	-1026.043798	0.278721	0.214233	-1026.078322	-90.6	-1025.799601	-1025.861077	13.5	-1020.257089	-4.286099	-1024.360466
conformer_196	-1026.035004	0.278911	0.213989	-1026.078311	-113.7	-1025.799400	-1025.861310	12.9	-1020.245546	-4.287338	-1024.359191
conformer_285	-1026.032882	0.278619	0.212595	-1026.078306	-119.3	-1025.799687	-1025.862699	9.2	-1020.249446	-4.282930	-1024.362194
conformer_99	-1026.040091	0.278629	0.215381	-1026.078229	-100.1	-1025.799600	-1025.859836	16.8	-1020.249717	-4.289075	-1024.358537
conformer_185	-1026.036051	0.278862	0.214641	-1026.078203	-110.7	-1025.799341	-1025.860550	14.9	-1020.246331	-4.288039	-1024.358869
conformer_184	-1026.035679	0.278530	0.212698	-1026.078120	-111.4	-1025.799590	-1025.862410	10.0	-1020.249235	-4.285371	-1024.361338
conformer_139	-1026.036142	0.278720	0.214235	-1026.078111	-110.2	-1025.799391	-1025.860864	14.1	-1020.251148	-4.284533	-1024.360402
conformer_317	-1026.032509	0.278593	0.212420	-1026.078078	-119.6	-1025.799485	-1025.862646	9.4	-1020.249148	-4.282893	-1024.362177
conformer_174	-1026.035801	0.278563	0.212979	-1026.078071	-111.0	-1025.799508	-1025.862080	10.9	-1020.249265	-4.285447	-1024.360991

conformer_7	-1026.040057	0.278657	0.213251	-1026.078034	-99.7	-1025.799377	-1025.861771	11.7	-1020.254500	-4.284897	-1024.361111
conformer_130	-1026.036240	0.278737	0.214190	-1026.078013	-109.7	-1025.799276	-1025.860811	14.2	-1020.251301	-4.284490	-1024.360362
conformer_68	-1026.038416	0.278569	0.213696	-1026.077998	-103.9	-1025.799429	-1025.861290	12.9	-1020.252297	-4.285075	-1024.360246
conformer_136	-1026.035001	0.278734	0.214247	-1026.077996	-112.9	-1025.799262	-1025.860737	14.4	-1020.247974	-4.286131	-1024.359842
conformer_206	-1026.034128	0.278518	0.213704	-1026.077922	-115.0	-1025.799404	-1025.861206	13.2	-1020.244491	-4.287337	-1024.358905
conformer_85	-1026.037724	0.278782	0.213384	-1026.077902	-105.5	-1025.799120	-1025.861506	12.4	-1020.253941	-4.283627	-1024.361350
conformer_138	-1026.035160	0.278763	0.214351	-1026.077900	-112.2	-1025.799137	-1025.860537	14.9	-1020.248208	-4.286073	-1024.359658
conformer_163	-1026.035160	0.278763	0.214350	-1026.077900	-112.2	-1025.799137	-1025.860538	14.9	-1020.248208	-4.286073	-1024.359659
conformer_61	-1026.037091	0.278418	0.212424	-1026.077894	-107.1	-1025.799476	-1025.862458	9.9	-1020.250206	-4.286022	-1024.361595
conformer_33	-1026.039154	0.278511	0.212789	-1026.077877	-101.7	-1025.799366	-1025.862076	10.9	-1020.253264	-4.285119	-1024.361304
conformer_118	-1026.034464	0.278604	0.213236	-1026.077852	-113.9	-1025.799248	-1025.861604	12.1	-1020.247270	-4.285207	-1024.359616
conformer_9	-1026.039878	0.278692	0.213687	-1026.077851	-99.7	-1025.799159	-1025.861152	13.3	-1020.254266	-4.284933	-1024.360472
conformer_37	-1026.038884	0.278560	0.213355	-1026.077812	-102.2	-1025.799252	-1025.861445	12.5	-1020.252833	-4.285272	-1024.360666
conformer_284	-1026.034675	0.278591	0.213650	-1026.077802	-113.2	-1025.799211	-1025.861140	13.3	-1020.245045	-4.287349	-1024.358859
conformer_208	-1026.035920	0.278621	0.213089	-1026.077782	-109.9	-1025.799161	-1025.861681	11.9	-1020.251113	-4.284261	-1024.361134
conformer_86	-1026.037588	0.278697	0.212769	-1026.077731	-105.4	-1025.799034	-1025.861950	11.2	-1020.253928	-4.283586	-1024.361877
conformer_283	-1026.036041	0.278401	0.211958	-1026.077728	-109.5	-1025.799327	-1025.862758	9.1	-1020.251208	-4.283972	-1024.361898
conformer_227	-1026.035020	0.278453	0.212620	-1026.077674	-112.0	-1025.799221	-1025.862042	11.0	-1020.249991	-4.283574	-1024.360587
conformer_71	-1026.038236	0.278337	0.212450	-1026.077629	-103.4	-1025.799292	-1025.862167	10.6	-1020.251658	-4.285389	-1024.360978
conformer_106	-1026.036364	0.278767	0.213843	-1026.077619	-108.3	-1025.798852	-1025.860764	14.3	-1020.247979	-4.286628	-1024.359007
conformer_79	-1026.038064	0.278638	0.214374	-1026.077612	-103.8	-1025.798974	-1025.860226	15.7	-1020.247545	-4.288838	-1024.358545
conformer_75	-1026.040565	0.278645	0.214905	-1026.077593	-97.2	-1025.798948	-1025.859676	17.2	-1020.250614	-4.289098	-1024.358822
conformer_28	-1026.037991	0.278935	0.215414	-1026.077579	-103.9	-1025.798644	-1025.859153	18.6	-1020.247319	-4.289464	-1024.357945
conformer_300	-1026.030728	0.278284	0.211660	-1026.077560	-123.0	-1025.799276	-1025.862888	8.7	-1020.243687	-4.285732	-1024.361579
conformer_182	-1026.033832	0.278683	0.214452	-1026.077539	-114.8	-1025.798856	-1025.860075	16.1	-1020.243306	-4.288410	-1024.357958
conformer_45	-1026.039227	0.278411	0.213357	-1026.077490	-100.5	-1025.799079	-1025.861121	13.4	-1020.251497	-4.286516	-1024.359907
conformer_6	-1026.044312	0.278995	0.216416	-1026.077489	-87.1	-1025.798494	-1025.858061	21.4	-1020.250878	-4.291094	-1024.355721
conformer_305	-1026.031323	0.278434	0.211894	-1026.077479	-121.2	-1025.799045	-1025.862573	9.6	-1020.247572	-4.282895	-1024.361717
conformer_233	-1026.034212	0.278519	0.212703	-1026.077299	-113.1	-1025.798780	-1025.861584	12.2	-1020.250613	-4.283412	-1024.361397
conformer_293	-1026.028998	0.278258	0.211244	-1026.077291	-126.8	-1025.799033	-1025.863035	8.4	-1020.244711	-4.283082	-1024.361829
conformer_193	-1026.034049	0.278725	0.212602	-1026.077271	-113.5	-1025.798546	-1025.861657	12.0	-1020.250778	-4.282636	-1024.361021
conformer_50	-1026.039713	0.278599	0.213162	-1026.077261	-98.6	-1025.798662	-1025.861087	13.5	-1020.254562	-4.284850	-1024.360786
conformer_116	-1026.035465	0.278988	0.213915	-1026.077244	-109.7	-1025.798256	-1025.860317	15.5	-1020.251243	-4.283456	-1024.359551
conformer_18	-1026.041824	0.278504	0.213592	-1026.077225	-92.9	-1025.798721	-1025.860621	14.7	-1020.254601	-4.286197	-1024.359595
conformer_110	-1026.035690	0.278999	0.213870	-1026.077214	-109.0	-1025.798215	-1025.860332	15.5	-1020.251466	-4.283435	-1024.359543
conformer_140	-1026.036657	0.278444	0.211718	-1026.077212	-106.5	-1025.798768	-1025.862482	9.8	-1020.254213	-4.282072	-1024.362110
conformer_119	-1026.037980	0.278464	0.212535	-1026.077196	-103.0	-1025.798732	-1025.861649	12.0	-1020.253086	-4.283743	-1024.360496
conformer_303	-1026.038620	0.278545	0.213215	-1026.077177	-101.2	-1025.798632	-1025.860950	13.8	-1020.251632	-4.285276	-1024.359238
conformer_197	-1026.033903	0.278679	0.212525	-1026.077160	-113.6	-1025.798481	-1025.861623	12.1	-1020.250618	-4.282619	-1024.360956
conformer_179	-1026.032734	0.278597	0.212974	-1026.077154	-116.6	-1025.798557	-1025.861168	13.3	-1020.245726	-4.285701	-1024.359861
conformer_215	-1026.032431	0.278533	0.212852	-1026.077150	-117.4	-1025.798617	-1025.861286	13.0	-1020.245400	-4.285744	-1024.359999
conformer_204	-1026.040778	0.278487	0.213354	-1026.077146	-95.5	-1025.798659	-1025.860780	14.3	-1020.253785	-4.285929	-1024.359715
conformer_122	-1026.040696	0.278795	0.215858	-1026.077118	-95.6	-1025.798323	-1025.858248	20.9	-1020.247947	-4.291317	-1024.356816
conformer_149	-1026.033722	0.278532	0.212893	-1026.077083	-113.8	-1025.798551	-1025.861178	13.2	-1020.246668	-4.285061	-1024.359185
conformer_306	-1026.029017	0.278282	0.211312	-1026.077054	-126.1	-1025.798772	-1025.862730	9.2	-1020.244710	-4.283083	-1024.361506
conformer_270	-1026.033880	0.278441	0.212195	-1026.077052	-113.3	-1025.798611	-1025.861845	11.5	-1020.250381	-4.283350	-1024.361696
conformer_266	-1026.034389	0.278441	0.212421	-1026.076948	-111.7	-1025.798507	-1025.861515	12.4	-1020.249492	-4.283438	-1024.360056
conformer_152	-1026.033795	0.278825	0.214549	-1026.076926	-113.2	-1025.798101	-1025.859365	18.0	-1020.242503	-4.288888	-1024.356961
conformer_247	-1026.033777	0.278538	0.211803	-1026.076909	-113.2	-1025.798371	-1025.862094	10.8	-1020.252353	-4.281174	-1024.361845
conformer_220	-1026.036509	0.278592	0.213013	-1026.076805	-105.8	-1025.798213	-1025.860780	14.3	-1020.252431	-4.283953	-1024.360655
conformer_76	-1026.039238	0.278600	0.213391	-1026.076793	-98.6	-1025.798193	-1025.860390	15.3	-1020.254161	-4.284734	-1024.360047
conformer_262	-1026.033679	0.278422	0.211431	-1026.076782	-113.2	-1025.798360	-1025.862339	10.2	-1020.252334	-4.281178	-1024.362172
conformer_91	-1026.039142	0.278317	0.213095	-1026.076752	-98.7	-1025.798435	-1025.860645	14.6	-1020.252367	-4.285988	-1024.359857
conformer_32	-1026.037494	0.278452	0.212919	-1026.076653	-102.8	-1025.798201	-1025.860722	14.4	-1020.251967	-4.284650	-1024.359845
conformer_77	-1026.036560	0.278314	0.212575	-1026.076646	-105.2	-1025.798332	-1025.861059	13.5	-1020.250608	-4.284944	-1024.360050
conformer_22	-1026.039805	0.278805	0.213525	-1026.076639	-96.7	-1025.797834	-1025.860102	16.1	-1020.255870	-4.283796	-1024.359962

conformer_64	-1026.039744	0.278779	0.213178	-1026.076625	-96.8	-1025.797846	-1025.860435	15.2	-1020.255842	-4.283861	-1024.360395
conformer_170	-1026.034135	0.278608	0.213145	-1026.076577	-111.4	-1025.797969	-1025.860420	15.2	-1020.250582	-4.283132	-1024.359999
conformer_35	-1026.037117	0.278405	0.212605	-1026.076519	-103.5	-1025.798114	-1025.860902	14.0	-1020.251426	-4.284822	-1024.360032
conformer_249	-1026.033795	0.278544	0.213489	-1026.076497	-112.1	-1025.797953	-1025.859996	16.3	-1020.249023	-4.284130	-1024.359353
conformer_160	-1026.034220	0.278501	0.212820	-1026.076477	-110.9	-1025.797976	-1025.860645	14.6	-1020.250750	-4.283113	-1024.360288
conformer_269	-1026.033647	0.278565	0.213544	-1026.076450	-112.4	-1025.797885	-1025.859894	16.6	-1020.248859	-4.284128	-1024.359234
conformer_272	-1026.034561	0.278630	0.212271	-1026.076439	-110.0	-1025.797809	-1025.861156	13.3	-1020.252213	-4.282099	-1024.360907
conformer_261	-1026.032913	0.278606	0.213734	-1026.076438	-114.3	-1025.797832	-1025.859692	17.1	-1020.246087	-4.285758	-1024.358623
conformer_291	-1026.032023	0.278826	0.215199	-1026.076431	-116.6	-1025.797605	-1025.858220	21.0	-1020.239779	-4.290479	-1024.356455
conformer_178	-1026.031903	0.278685	0.213506	-1026.076421	-116.9	-1025.797736	-1025.859903	16.6	-1020.246080	-4.284814	-1024.358894
conformer_83	-1026.036055	0.278258	0.212268	-1026.076385	-105.9	-1025.798127	-1025.861105	13.4	-1020.249948	-4.285151	-1024.360149
conformer_280	-1026.032666	0.278645	0.213808	-1026.076337	-114.7	-1025.797692	-1025.859517	17.6	-1020.245811	-4.285761	-1024.358423
conformer_164	-1026.032041	0.278602	0.213237	-1026.076301	-116.2	-1025.797699	-1025.860052	16.2	-1020.246311	-4.284807	-1024.359129
conformer_109	-1026.037013	0.278240	0.212686	-1026.076274	-103.1	-1025.798034	-1025.860576	14.8	-1020.251059	-4.285565	-1024.360187
conformer_172	-1026.035003	0.278540	0.212371	-1026.076271	-108.3	-1025.797731	-1025.860888	14.0	-1020.251466	-4.283179	-1024.360530
conformer_20	-1026.039453	0.278782	0.213259	-1026.076258	-96.6	-1025.797476	-1025.859987	16.4	-1020.256696	-4.282812	-1024.360042
conformer_166	-1026.035043	0.278518	0.212304	-1026.076215	-108.1	-1025.797697	-1025.860899	14.0	-1020.251517	-4.283209	-1024.360583
conformer_264	-1026.034292	0.278426	0.212366	-1026.076106	-109.8	-1025.797680	-1025.860728	14.4	-1020.246921	-4.285684	-1024.359041
conformer_21	-1026.043533	0.278823	0.215263	-1026.076094	-85.5	-1025.797271	-1025.857819	22.1	-1020.252049	-4.289806	-1024.356141
conformer_26	-1026.039330	0.278768	0.213377	-1026.076074	-96.5	-1025.797306	-1025.859685	17.2	-1020.256590	-4.282874	-1024.359820
conformer_232	-1026.035112	0.278582	0.212241	-1026.075891	-107.1	-1025.797309	-1025.860638	14.7	-1020.250955	-4.283656	-1024.360136
conformer_281	-1026.030383	0.278362	0.212087	-1026.075847	-119.4	-1025.797485	-1025.860748	14.4	-1020.243259	-4.285592	-1024.359216
conformer_47	-1026.037804	0.278355	0.212562	-1026.075818	-99.8	-1025.797463	-1025.860244	15.7	-1020.253102	-4.284293	-1024.359836
conformer_107	-1026.037139	0.278387	0.212728	-1026.075813	-101.5	-1025.797426	-1025.860073	16.1	-1020.252088	-4.284466	-1024.359487
conformer_102	-1026.036873	0.278222	0.212277	-1026.075767	-102.1	-1025.797545	-1025.860478	15.1	-1020.251703	-4.284652	-1024.359960
conformer_134	-1026.035363	0.278826	0.213724	-1026.075724	-106.0	-1025.796898	-1025.858988	19.0	-1020.250867	-4.283960	-1024.358453
conformer_214	-1026.032298	0.278873	0.213290	-1026.075714	-114.0	-1025.796841	-1025.859412	17.9	-1020.247248	-4.284018	-1024.358379
conformer_146	-1026.035163	0.278754	0.213550	-1026.075624	-106.2	-1025.796870	-1025.859062	18.8	-1020.250709	-4.283962	-1024.358570
conformer_137	-1026.035055	0.278642	0.213157	-1026.075595	-106.4	-1025.796953	-1025.859426	17.8	-1020.252271	-4.282536	-1024.359179
conformer_218	-1026.033067	0.278726	0.212989	-1026.075593	-111.7	-1025.796867	-1025.859592	17.4	-1020.249178	-4.282992	-1024.358695
conformer_304	-1026.034666	0.278651	0.214716	-1026.075551	-107.3	-1025.796900	-1025.857823	22.0	-1020.242872	-4.289624	-1024.355653
conformer_295	-1026.029085	0.278637	0.214861	-1026.075532	-121.9	-1025.796895	-1025.857659	22.5	-1020.235614	-4.292031	-1024.356219
conformer_95	-1026.037485	0.278709	0.213197	-1026.075488	-99.8	-1025.796779	-1025.859279	18.2	-1020.254088	-4.283307	-1024.359190
conformer_94	-1026.037555	0.278779	0.213496	-1026.075468	-99.5	-1025.796689	-1025.858960	19.1	-1020.254096	-4.283286	-1024.358787
conformer_246	-1026.032818	0.278714	0.212860	-1026.075465	-112.0	-1025.796751	-1025.859593	17.4	-1020.248901	-4.282982	-1024.358657
conformer_131	-1026.035119	0.278555	0.212891	-1026.075440	-105.9	-1025.796885	-1025.859537	17.5	-1020.252411	-4.282520	-1024.359349
conformer_199	-1026.031767	0.278726	0.213699	-1026.075413	-114.6	-1025.796687	-1025.858702	19.7	-1020.246130	-4.284655	-1024.357720
conformer_240	-1026.035546	0.278461	0.212114	-1026.075413	-104.7	-1025.796952	-1025.860287	15.6	-1020.252901	-4.282714	-1024.360356
conformer_48	-1026.037517	0.278557	0.212507	-1026.075368	-99.4	-1025.796811	-1025.859849	16.7	-1020.253763	-4.283349	-1024.359444
conformer_57	-1026.037298	0.278608	0.212894	-1026.075355	-99.9	-1025.796747	-1025.859449	17.8	-1020.254925	-4.282733	-1024.359809
conformer_67	-1026.037060	0.278600	0.212961	-1026.075144	-100.0	-1025.796544	-1025.859171	18.5	-1020.254672	-4.282824	-1024.359607
conformer_212	-1026.032421	0.278587	0.212756	-1026.075057	-111.9	-1025.796470	-1025.859289	18.2	-1020.247520	-4.284215	-1024.358603
conformer_226	-1026.035650	0.278560	0.212222	-1026.074959	-103.2	-1025.796399	-1025.859725	17.0	-1020.252348	-4.283233	-1024.359656
conformer_169	-1026.037264	0.278283	0.212259	-1026.074914	-98.8	-1025.796631	-1025.859643	17.3	-1020.253003	-4.284152	-1024.359534
conformer_202	-1026.032535	0.278509	0.212515	-1026.074873	-111.2	-1025.796364	-1025.859346	18.0	-1020.247739	-4.284186	-1024.358736
conformer_132	-1026.037374	0.278597	0.212945	-1026.074845	-98.4	-1025.796248	-1025.858888	19.2	-1020.255424	-4.282479	-1024.359417
conformer_74	-1026.038213	0.278486	0.212264	-1026.074842	-96.2	-1025.796356	-1025.859566	17.5	-1020.255327	-4.282817	-1024.359497
conformer_301	-1026.031574	0.278398	0.212398	-1026.074814	-113.5	-1025.796416	-1025.859404	17.9	-1020.248343	-4.282650	-1024.358823
conformer_311	-1026.031438	0.278410	0.212411	-1026.074791	-113.8	-1025.796381	-1025.859368	18.0	-1020.248167	-4.282671	-1024.358768
conformer_294	-1026.029599	0.278476	0.212739	-1026.074731	-118.5	-1025.796255	-1025.858980	19.0	-1020.244076	-4.284353	-1024.357810
conformer_308	-1026.029374	0.278488	0.212789	-1026.074676	-118.9	-1025.796188	-1025.858875	19.3	-1020.243792	-4.284387	-1024.357680
conformer_299	-1026.030344	0.278610	0.212952	-1026.074575	-116.1	-1025.795965	-1025.858611	20.0	-1020.242259	-4.287315	-1024.357842
conformer_133	-1026.038254	0.278703	0.213088	-1026.074487	-95.1	-1025.795784	-1025.858387	20.6	-1020.255606	-4.282930	-1024.358669
conformer_80	-1026.038303	0.278774	0.213326	-1026.074453	-94.9	-1025.795679	-1025.858115	21.3	-1020.255546	-4.282981	-1024.358339
conformer_314	-1026.031661	0.278782	0.214290	-1026.074443	-112.3	-1025.795661	-1025.857141	23.8	-1020.240749	-4.289768	-1024.355997
conformer_5	-1026.039275	0.278943	0.214052	-1026.074422	-92.3	-1025.795479	-1025.857358	23.3	-1020.255803	-4.283189	-1024.357075

conformer_63	-1026.036625	0.278489	0.212234	-1026.074358	-99.1	-1025.795869	-1025.859112	18.7	-1020.254109	-4.282503	-1024.359098
conformer_73	-1026.036534	0.278471	0.212285	-1026.074275	-99.1	-1025.795804	-1025.858978	19.0	-1020.253994	-4.282605	-1024.359044
conformer_277	-1026.032553	0.278539	0.212835	-1026.074073	-109.0	-1025.795534	-1025.858226	21.0	-1020.248364	-4.283495	-1024.357531
conformer_8	-1026.039043	0.278873	0.213700	-1026.074026	-91.8	-1025.795153	-1025.857314	23.4	-1020.255625	-4.283259	-1024.357154
conformer_288	-1026.032530	0.278896	0.214929	-1026.073859	-108.5	-1025.794963	-1025.855918	27.0	-1020.240480	-4.290655	-1024.354523
conformer_123	-1026.034385	0.278328	0.212009	-1026.073827	-103.6	-1025.795499	-1025.858806	19.5	-1020.252170	-4.282346	-1024.358937
conformer_190	-1026.034793	0.278511	0.212623	-1026.073826	-102.5	-1025.795315	-1025.858191	21.1	-1020.251605	-4.282889	-1024.357892
conformer_191	-1026.034781	0.278509	0.212505	-1026.073811	-102.5	-1025.795302	-1025.858294	20.8	-1020.251590	-4.282909	-1024.358012
conformer_168	-1026.031638	0.278802	0.214822	-1026.073793	-110.7	-1025.794991	-1025.855959	26.9	-1020.241026	-4.289077	-1024.354424
conformer_257	-1026.032474	0.278445	0.212421	-1026.073781	-108.5	-1025.795336	-1025.858348	20.7	-1020.249987	-4.282070	-1024.357931
conformer_267	-1026.032388	0.278482	0.212570	-1026.073771	-108.7	-1025.795289	-1025.858189	21.1	-1020.249854	-4.282097	-1024.357752
conformer_254	-1026.032715	0.278576	0.212745	-1026.073673	-107.5	-1025.795097	-1025.857916	21.8	-1020.248503	-4.283589	-1024.357294
conformer_148	-1026.034330	0.278839	0.215383	-1026.073594	-103.1	-1025.794755	-1025.855199	28.9	-1020.239194	-4.292172	-1024.352235
conformer_87	-1026.037233	0.278435	0.212126	-1026.073507	-95.2	-1025.795072	-1025.858369	20.6	-1020.255629	-4.282012	-1024.358777
conformer_162	-1026.033776	0.278276	0.211886	-1026.073505	-104.3	-1025.795229	-1025.858607	20.0	-1020.251468	-4.282585	-1024.358884
conformer_268	-1026.034495	0.278326	0.212061	-1026.073301	-101.9	-1025.794975	-1025.858228	21.0	-1020.252738	-4.282069	-1024.358540
conformer_315	-1026.033843	0.278272	0.211878	-1026.072985	-102.8	-1025.794713	-1025.858095	21.3	-1020.251993	-4.282252	-1024.358497
conformer_156	-1026.035520	0.278510	0.212409	-1026.072891	-98.1	-1025.794381	-1025.857470	23.0	-1020.253057	-4.282538	-1024.357544
conformer_154	-1026.035526	0.278529	0.212512	-1026.072874	-98.1	-1025.794345	-1025.857350	23.3	-1020.253084	-4.282540	-1024.357448
conformer_255	-1026.030117	0.278770	0.213268	-1026.072837	-112.2	-1025.794067	-1025.856557	25.4	-1020.240979	-4.288471	-1024.355890
conformer_34	-1026.036384	0.278600	0.212684	-1026.072698	-95.3	-1025.794098	-1025.857002	24.2	-1020.253163	-4.282782	-1024.356563
conformer_225	-1026.034355	0.278215	0.211627	-1026.072686	-100.6	-1025.794471	-1025.858047	21.5	-1020.252971	-4.281983	-1024.358647
conformer_252	-1026.033109	0.278673	0.213720	-1026.072653	-103.8	-1025.793980	-1025.855921	27.0	-1020.246884	-4.286291	-1024.355987
conformer_42	-1026.036053	0.278576	0.212786	-1026.072564	-95.9	-1025.793988	-1025.856766	24.8	-1020.252779	-4.282990	-1024.356482
conformer_222	-1026.033364	0.278674	0.213751	-1026.072284	-102.2	-1025.793610	-1025.855521	28.1	-1020.246485	-4.286867	-1024.355509
conformer_239	-1026.033601	0.278515	0.211856	-1026.072071	-101.0	-1025.793556	-1025.857203	23.7	-1020.251704	-4.282302	-1024.357608
conformer_245	-1026.035908	0.278446	0.212206	-1026.072047	-94.9	-1025.793601	-1025.856829	24.7	-1020.254398	-4.282001	-1024.357319
conformer_217	-1026.029705	0.278668	0.214584	-1026.071881	-110.7	-1025.793213	-1025.854285	31.3	-1020.237670	-4.290823	-1024.353072
conformer_60	-1026.036684	0.278493	0.212385	-1026.071804	-92.2	-1025.793311	-1025.856407	25.8	-1020.254368	-4.282360	-1024.356451
conformer_253	-1026.033430	0.278476	0.211509	-1026.071701	-100.5	-1025.793225	-1025.857180	23.7	-1020.251422	-4.282549	-1024.357720
conformer_198	-1026.031326	0.278721	0.214368	-1026.071551	-105.6	-1025.792830	-1025.854171	31.6	-1020.241843	-4.288963	-1024.353651
conformer_282	-1026.027490	0.279146	0.214830	-1026.067167	-104.2	-1025.788021	-1025.849325	44.4	-1020.236427	-4.290465	-1024.348728
conformer_289	-1026.027464	0.279079	0.214888	-1026.067075	-104.0	-1025.787996	-1025.849175	44.7	-1020.236486	-4.290341	-1024.348538

[a]: Single-point calculation in aqueous phase with SMD model.

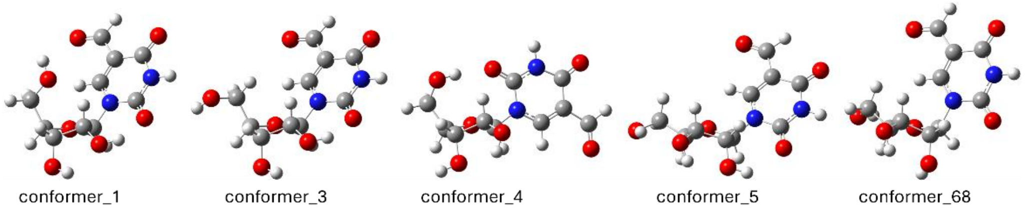

Figure S44. B3LYP-D3/def2-TZVPP optimized geometries of conformers for 5-formyluridine (**1rb5fU**).

Table S42. Conformers of gas-phase optimized 5-formyluridine (**1rb5fU**) at the B3LYP-D3/def2-TZVPP level of theory followed by aqueous phase single-point calculation. The columns display total energy without zero-point correction (E_{Tot}), Gibbs free energy (δG), total energy without zero-point correction ($E_{Tot,W}$), Gibbs free energy ($G_{298,W}$) in water (W), total single-point energy (E_{CBS}) calculated at DLPNO-CCSD(T)/CBS level of theory, and their corresponding free energy G_{CBS}. $G_{298,W}$ and G_{CBS} have been corrected to the standard state of 1 mol/L by addition of +7.908 kJ/mol. ΔG_{Solv} represents the Gibbs free energy of solvation. The data are arranged in the ascending numeric order of $E_{Tot,W}$. $\Delta G_{298,W}$ represents the respective energy difference to the lowest structure. Only conformers within the 24 kJ/mol (6 kcal/mol) energy window above the lowest in CREST are included in initial conformer sampling. Duplicates of the same structure are excluded.

	**B3LYP-D3/def2-TZVPP**			**SMD(H_2O)/B3LYP-D3/def2-TZVPP**[a]					**DLPNO-CCSD(T)/CBS**		
**1rb5fU No.**	**E_{Tot}** (Hartree)	**δH** (Hartree)	**δG** (Hartree)	**$E_{Tot,W}$** (Hartree)	**ΔG_{Solv}** (kJ/mol)	**$H_{298,W}$** (Hartree)	**$G_{298,W}$** (Hartree)	**$\Delta G_{298,W}$** (kJ/mol)	**$E_{CBS,HF}$** (Hartree)	**$E_{CBS,C}$** (Hartree)	**$G_{CBS,W}$** (Hartree)
conformer_30	-1024.828993	0.254407	0.191337	-1024.866338	-98.0	-1024.611931	-1024.671989	3.8	-1019.089791	-4.237052	-1023.169839
conformer_7	-1024.826487	0.254368	0.191228	-1024.866328	-104.6	-1024.611960	-1024.672088	3.5	-1019.085388	-4.238130	-1023.169120
conformer_4	-1024.828768	0.254343	0.191101	-1024.866310	-98.6	-1024.611967	-1024.672197	3.3	-1019.089528	-4.236767	-1023.169724
conformer_46	-1024.825397	0.254397	0.190763	-1024.865463	-105.2	-1024.611066	-1024.671688	4.6	-1019.086239	-4.236565	-1023.169094
**conformer_68**	**-1024.820937**	**0.253952**	**0.188885**	**-1024.865333**	**-116.6**	**-1024.611381**	**-1024.673436**	**0**	**-1019.080861**	**-4.237349**	**-1023.170709**
conformer_9	-1024.829692	0.254134	0.189643	-1024.865231	-93.3	-1024.611097	-1024.672576	2.3	-1019.090698	-4.235865	-1023.169447
conformer_69	-1024.825848	0.254177	0.188678	-1024.865115	-103.1	-1024.610938	-1024.673425	0.1	-1019.089602	-4.234018	-1023.171197
conformer_1	-1024.833143	0.254254	0.191000	-1024.864881	-83.3	-1024.610627	-1024.670869	6.7	-1019.092450	-4.237925	-1023.168100
conformer_14	-1024.827435	0.253997	0.189656	-1024.864751	-98.0	-1024.610754	-1024.672083	3.6	-1019.087875	-4.236941	-1023.169464
conformer_53	-1024.820333	0.253985	0.188402	-1024.864682	-116.4	-1024.610697	-1024.673268	0.4	-1019.081926	-4.235844	-1023.170706
conformer_12	-1024.827466	0.254217	0.190489	-1024.864503	-97.2	-1024.610286	-1024.671002	6.4	-1019.088318	-4.236955	-1023.168809
conformer_10	-1024.826574	0.254027	0.190524	-1024.864319	-99.1	-1024.610292	-1024.670783	7.0	-1019.086708	-4.237877	-1023.168794
conformer_52	-1024.821683	0.254002	0.188457	-1024.864299	-111.9	-1024.610297	-1024.672830	1.6	-1019.085314	-4.234243	-1023.170703
conformer_16	-1024.826835	0.254175	0.189977	-1024.864228	-98.2	-1024.610053	-1024.671239	5.8	-1019.088425	-4.235182	-1023.168011
conformer_6	-1024.825863	0.254257	0.190395	-1024.864222	-100.7	-1024.609965	-1024.670815	6.9	-1019.085196	-4.236973	-1023.167121
conformer_62	-1024.828080	0.254213	0.190996	-1024.864200	-94.8	-1024.609987	-1024.670192	8.5	-1019.085589	-4.238696	-1023.166398
conformer_20	-1024.829193	0.254116	0.191902	-1024.864019	-91.4	-1024.609903	-1024.669105	11.4	-1019.085950	-4.240626	-1023.166488
conformer_3	-1024.827047	0.254113	0.189880	-1024.863935	-96.9	-1024.609822	-1024.671043	6.3	-1019.088510	-4.236361	-1023.168867
conformer_28	-1024.823387	0.254330	0.189490	-1024.863929	-106.4	-1024.609599	-1024.671427	5.3	-1019.086738	-4.234678	-1023.169456
conformer_15	-1024.825685	0.253980	0.189579	-1024.863856	-100.2	-1024.609876	-1024.671265	5.7	-1019.086706	-4.236708	-1023.168995
conformer_49	-1024.824518	0.254113	0.189454	-1024.863727	-102.9	-1024.609614	-1024.671261	5.7	-1019.086319	-4.235963	-1023.169025
conformer_34	-1024.819030	0.253983	0.188357	-1024.863653	-117.2	-1024.609670	-1024.672284	3.0	-1019.081285	-4.235111	-1023.169651
conformer_74	-1024.818008	0.253814	0.187393	-1024.863580	-119.6	-1024.609766	-1024.673175	0.7	-1019.080281	-4.235099	-1023.170547
conformer_26	-1024.825555	0.254278	0.191495	-1024.863473	-99.6	-1024.609195	-1024.668966	11.7	-1019.080731	-4.240556	-1023.164698
conformer_36	-1024.823260	0.254231	0.190492	-1024.863332	-105.2	-1024.609101	-1024.669828	9.5	-1019.085320	-4.235911	-1023.167799
conformer_56	-1024.820122	0.254105	0.189489	-1024.863243	-113.2	-1024.609138	-1024.670742	7.1	-1019.079666	-4.237473	-1023.167759
conformer_45	-1024.822006	0.254267	0.190590	-1024.863180	-108.1	-1024.608913	-1024.669578	10.1	-1019.082052	-4.237508	-1023.167132
conformer_18	-1024.826822	0.254064	0.189756	-1024.863078	-95.2	-1024.609014	-1024.670310	8.2	-1019.088666	-4.236162	-1023.168316
conformer_42	-1024.821234	0.253962	0.187787	-1024.862951	-109.5	-1024.608989	-1024.672152	3.4	-1019.085964	-4.233254	-1023.170136
conformer_23	-1024.824424	0.254233	0.189533	-1024.862941	-101.1	-1024.608708	-1024.670396	8.0	-1019.087551	-4.235223	-1023.168746
conformer_39	-1024.825675	0.254146	0.189657	-1024.862823	-97.5	-1024.608677	-1024.670154	8.6	-1019.088065	-4.235661	-1023.168205

conformer_63	-1024.819597	0.253938	0.188191	-1024.862711	-113.2	-1024.608773	-1024.671508	5.1	-1019.082286	-4.234838	-1023.169035
conformer_37	-1024.822138	0.253978	0.187976	-1024.862348	-105.6	-1024.608370	-1024.671360	5.5	-1019.087327	-4.232967	-1023.169515
conformer_35	-1024.822358	0.254457	0.190060	-1024.862182	-104.6	-1024.607725	-1024.669110	11.4	-1019.085077	-4.235024	-1023.166854
conformer_11	-1024.826201	0.254229	0.189510	-1024.862060	-94.1	-1024.607831	-1024.669538	10.2	-1019.090569	-4.234028	-1023.167934
conformer_43	-1024.821638	0.254117	0.189484	-1024.861684	-105.1	-1024.607567	-1024.669188	11.2	-1019.085104	-4.234534	-1023.167189
conformer_51	-1024.819312	0.254178	0.189786	-1024.861628	-111.1	-1024.607450	-1024.668830	12.1	-1019.080504	-4.236263	-1023.166286
conformer_24	-1024.823343	0.254053	0.189112	-1024.861155	-99.3	-1024.607102	-1024.669031	11.6	-1019.087994	-4.234015	-1023.167697
conformer_61	-1024.823941	0.254068	0.189197	-1024.860753	-96.7	-1024.606685	-1024.668544	12.8	-1019.089077	-4.233564	-1023.167245
conformer_47	-1024.821973	0.254272	0.189939	-1024.860742	-101.8	-1024.606470	-1024.667791	14.8	-1019.084392	-4.235573	-1023.165782
conformer_32	-1024.822519	0.254168	0.189555	-1024.860658	-100.1	-1024.606490	-1024.668091	14.0	-1019.086739	-4.233941	-1023.166252
conformer_22	-1024.824534	0.254235	0.189637	-1024.860416	-94.2	-1024.606181	-1024.667767	14.9	-1019.088017	-4.234908	-1023.166159
conformer_5	-1024.825670	0.254343	0.189957	-1024.860340	-91.0	-1024.605997	-1024.667371	15.9	-1019.089252	-4.234483	-1023.165437
conformer_70	-1024.819795	0.254045	0.188611	-1024.860109	-105.8	-1024.606064	-1024.668486	13.0	-1019.081988	-4.235611	-1023.166290
conformer_21	-1024.825285	0.254241	0.189640	-1024.859519	-89.9	-1024.605278	-1024.666867	17.2	-1019.089523	-4.234501	-1023.165606
conformer_55	-1024.818525	0.254432	0.191634	-1024.858711	-105.5	-1024.604279	-1024.664065	24.6	-1019.074759	-4.240792	-1023.161091
conformer_72	-1024.820239	0.254081	0.189577	-1024.857657	-98.2	-1024.603576	-1024.665068	22.0	-1019.080689	-4.238025	-1023.163542

[a]: Single-point calculation in aqueous phase with SMD model.

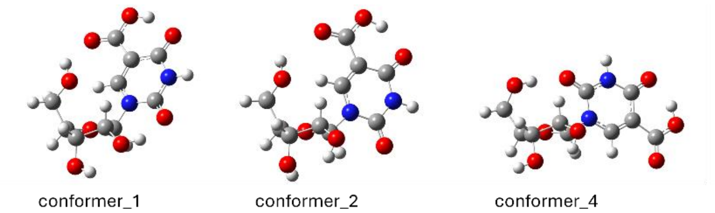

Figure S45. B3LYP-D3/def2-TZVPP optimized geometries of conformers for 5-carboxyluridine (**1rb5caU**).

Table S43. Conformers of gas-phase optimized 5-carboxyluridine (**1rb5caU**) at the B3LYP-D3/def2-TZVPP level of theory followed by aqueous phase single-point calculation. The columns display total energy without zero-point correction (E_{Tot}), Gibbs free energy (δG), total energy without zero-point correction ($E_{Tot,W}$), Gibbs free energy ($G_{298,W}$) in water (W), total single-point energy (E_{CBS}) calculated at DLPNO-CCSD(T)/CBS level of theory, and their corresponding free energy G_{CBS}. $G_{298,W}$ and G_{CBS} have been corrected to the standard state of 1 mol/L by addition of +7.908 kJ/mol. ΔG_{Solv} represents the Gibbs free energy of solvation. The data are arranged in the ascending numeric order of $E_{Tot,W}$. $\Delta G_{298,W}$ represents the respective energy difference to the lowest structure. Only conformers within the 24 kJ/mol (6 kcal/mol) energy window above the lowest in CREST are included in initial conformer sampling. Duplicates of the same structure are excluded.

	B3LYP-D3/def2-TZVPP			SMD(H_2O)/B3LYP-D3/def2-TZVPP[a]					DLPNO-CCSD(T)/CBS		
1rb5caU No.	E_{Tot} (Hartree)	δH (Hartree)	δG (Hartree)	$E_{Tot,W}$ (Hartree)	ΔG_{Solv} (kJ/mol)	$H_{298,W}$ (Hartree)	$G_{298,W}$ (Hartree)	$\Delta G_{298,W}$ (kJ/mol)	$E_{CBS,HF}$ (Hartree)	$E_{CBS,C}$ (Hartree)	$G_{CBS,W}$ (Hartree)
conformer_7	-1100.118831	0.260925	0.196433	-1100.161992	-113.3	-1099.901067	-1099.962547	4.6	-1094.016969	-4.512051	-1098.372736
conformer_4	-1100.121170	0.260887	0.196252	-1100.161954	-107.1	-1099.901067	-1099.962690	4.3	-1094.021174	-4.510702	-1098.373395
conformer_40	-1100.121241	0.260959	0.196565	-1100.161945	-106.9	-1099.900986	-1099.962368	5.1	-1094.021245	-4.510974	-1098.373347
**conformer_19**	**-1100.118230**	**0.260687**	**0.194306**	**-1100.161629**	**-113.9**	**-1099.900942**	**-1099.964311**	**0**	**-1094.020716**	**-4.508367**	**-1098.375163**
conformer_76	-1100.113566	0.260539	0.194353	-1100.161302	-125.3	-1099.900763	-1099.963937	1.0	-1094.012637	-4.511298	-1098.374307
conformer_70	-1100.117636	0.260956	0.195932	-1100.161161	-114.3	-1099.900205	-1099.962217	5.5	-1094.017679	-4.510405	-1098.372665
conformer_10	-1100.121148	0.260850	0.194894	-1100.161031	-104.7	-1099.900181	-1099.963125	3.1	-1094.021345	-4.509684	-1098.373005
conformer_1	-1100.126246	0.260993	0.196498	-1100.160804	-90.7	-1099.899811	-1099.961294	7.9	-1094.024057	-4.512352	-1098.371456
conformer_75	-1100.115003	0.260489	0.193584	-1100.160704	-120.0	-1099.900215	-1099.964108	0.5	-1094.017873	-4.508167	-1098.375145
conformer_12	-1100.123016	0.260729	0.195206	-1100.160642	-98.8	-1099.899913	-1099.962424	5.0	-1094.021552	-4.511134	-1098.372094
conformer_57	-1100.113095	0.260567	0.193703	-1100.160463	-124.4	-1099.899896	-1099.963748	1.5	-1094.013902	-4.509673	-1098.374229
conformer_14	-1100.119681	0.260790	0.195667	-1100.160167	-106.3	-1099.899377	-1099.961488	7.4	-1094.019731	-4.510840	-1098.372378
conformer_53	-1100.114639	0.260616	0.193952	-1100.160146	-119.5	-1099.899530	-1099.963182	3.0	-1094.017454	-4.508139	-1098.374136
conformer_17	-1100.118575	0.260657	0.196009	-1100.160015	-108.8	-1099.899358	-1099.960994	8.7	-1094.017692	-4.511967	-1098.372078
conformer_26	-1100.121923	0.260786	0.196563	-1100.159925	-99.8	-1099.899139	-1099.960350	10.4	-1094.018106	-4.513365	-1098.369898
conformer_16	-1100.122343	0.260772	0.197507	-1100.159919	-98.7	-1099.899147	-1099.959400	12.9	-1094.018084	-4.514695	-1098.369835
conformer_6	-1100.120667	0.260621	0.194911	-1100.159818	-102.8	-1099.899197	-1099.961895	6.3	-1094.020928	-4.509750	-1098.371906
conformer_5	-1100.119252	0.260653	0.194838	-1100.159790	-106.4	-1099.899137	-1099.961940	6.2	-1094.019879	-4.510392	-1098.372960
conformer_2	-1100.119665	0.260775	0.195490	-1100.159704	-105.1	-1099.898929	-1099.961202	8.2	-1094.017368	-4.511633	-1098.370538
conformer_37	-1100.115750	0.260950	0.194946	-1100.159682	-115.3	-1099.898732	-1099.961724	6.8	-1094.018248	-4.508616	-1098.372838
conformer_48	-1100.117551	0.260745	0.194984	-1100.159645	-110.5	-1099.898900	-1099.961649	7.0	-1094.018393	-4.510157	-1098.372648
conformer_36	-1100.111921	0.260606	0.193666	-1100.159583	-125.1	-1099.898977	-1099.962905	3.7	-1094.013204	-4.509151	-1098.373338
conformer_20	-1100.117750	0.260534	0.194723	-1100.159502	-109.6	-1099.898968	-1099.961767	6.7	-1094.017974	-4.510611	-1098.372602
conformer_66	-1100.112673	0.260727	0.194938	-1100.159275	-122.4	-1099.898548	-1099.961325	7.8	-1094.011285	-4.511516	-1098.371453
conformer_81	-1100.110927	0.260481	0.192788	-1100.159271	-126.9	-1099.898790	-1099.963471	2.2	-1094.012286	-4.509038	-1098.373867
conformer_18	-1100.119346	0.260970	0.197426	-1100.159200	-104.6	-1099.898230	-1099.958762	14.6	-1094.013293	-4.515062	-1098.367772
conformer_41	-1100.115713	0.260826	0.195792	-1100.159034	-113.7	-1099.898208	-1099.960230	10.7	-1094.016959	-4.509871	-1098.371347
conformer_22	-1100.119147	0.260578	0.194687	-1100.159032	-104.7	-1099.898454	-1099.961333	7.8	-1094.020027	-4.510378	-1098.372591
conformer_60	-1100.114372	0.260865	0.195928	-1100.158911	-116.9	-1099.898046	-1099.959971	11.4	-1094.013620	-4.511383	-1098.370602
conformer_38	-1100.114264	0.260583	0.193329	-1100.158901	-117.2	-1099.898318	-1099.962560	4.6	-1094.018048	-4.507368	-1098.373712
conformer_30	-1100.118929	0.260820	0.195317	-1100.158764	-104.6	-1099.897944	-1099.960435	10.2	-1094.020318	-4.509892	-1098.371716

conformer_31	-1100.116751	0.260835	0.194904	-1100.158685	-110.1	-1099.897850	-1099.960769	9.3	-1094.019012	-4.509168	-1098.372199
conformer_52	-1100.112733	0.260600	0.193713	-1100.158676	-120.6	-1099.898076	-1099.961951	6.2	-1094.014315	-4.509016	-1098.372549
conformer_33	-1100.115352	0.260631	0.193563	-1100.158366	-112.9	-1099.897735	-1099.961791	6.6	-1094.019579	-4.506958	-1098.372976
conformer_15	-1100.118379	0.260778	0.194815	-1100.158135	-104.4	-1099.897357	-1099.960308	10.5	-1094.021870	-4.507879	-1098.371679
conformer_84	-1100.112706	0.260850	0.193465	-1100.158023	-119.0	-1099.897173	-1099.961546	7.3	-1094.014394	-4.508774	-1098.372008
conformer_50	-1100.114723	0.261034	0.195367	-1100.157918	-113.4	-1099.896884	-1099.959539	12.5	-1094.016605	-4.508968	-1098.370389
conformer_83	-1100.114427	0.260780	0.196870	-1100.157820	-113.9	-1099.897040	-1099.957938	16.7	-1094.007310	-4.517026	-1098.367847
conformer_54	-1100.114284	0.260694	0.194780	-1100.157427	-113.3	-1099.896733	-1099.959635	12.3	-1094.016974	-4.508490	-1098.370816
conformer_61	-1100.111884	0.260742	0.195051	-1100.157378	-119.4	-1099.896636	-1099.959315	13.1	-1094.012292	-4.510185	-1098.369908
conformer_44	-1100.115360	0.260593	0.194323	-1100.157254	-110.0	-1099.896661	-1099.959919	11.5	-1094.019106	-4.507873	-1098.371539
conformer_69	-1100.114275	0.260825	0.195204	-1100.156435	-110.7	-1099.895610	-1099.958219	16.0	-1094.015865	-4.509536	-1098.369345
conformer_39	-1100.115129	0.260745	0.194872	-1100.156401	-108.4	-1099.895656	-1099.958517	15.2	-1094.018566	-4.507924	-1098.369878
conformer_55	-1100.112390	0.260840	0.194217	-1100.156382	-115.5	-1099.895542	-1099.959153	13.5	-1094.014583	-4.508381	-1098.369728
conformer_9	-1100.117724	0.260903	0.195196	-1100.156314	-101.3	-1099.895411	-1099.958106	16.3	-1094.020465	-4.508432	-1098.369278
conformer_27	-1100.117012	0.260799	0.194876	-1100.156118	-102.7	-1099.895319	-1099.958230	16.0	-1094.019681	-4.508885	-1098.369785
conformer_46	-1100.110546	0.260783	0.195046	-1100.155889	-119.0	-1099.895106	-1099.957831	17.0	-1094.011400	-4.509425	-1098.368111
conformer_32	-1100.112730	0.260732	0.194808	-1100.155852	-113.2	-1099.895120	-1099.958032	16.5	-1094.015487	-4.508059	-1098.368848
conformer_23	-1100.117743	0.260819	0.194947	-1100.155217	-98.4	-1099.894398	-1099.957258	18.5	-1094.021166	-4.508515	-1098.369197
conformer_43	-1100.114145	0.260687	0.194847	-1100.154872	-106.9	-1099.894185	-1099.957013	19.2	-1094.014910	-4.508788	-1098.366566
conformer_58	-1100.114314	0.260666	0.196394	-1100.154505	-105.5	-1099.893839	-1099.955099	24.2	-1094.012701	-4.512093	-1098.365578
conformer_56	-1100.111161	0.260950	0.196722	-1100.154497	-113.8	-1099.893547	-1099.954763	25.1	-1094.006432	-4.514861	-1098.364894
conformer_71	-1100.111519	0.260695	0.194465	-1100.154012	-111.6	-1099.893317	-1099.956535	20.4	-1094.014247	-4.508231	-1098.367493
conformer_11	-1100.117883	0.260990	0.196801	-1100.153865	-94.5	-1099.892875	-1099.954052	26.9	-1094.016381	-4.511080	-1098.363630
conformer_67	-1100.110452	0.260533	0.194586	-1100.153702	-113.6	-1099.893169	-1099.956104	21.5	-1094.012328	-4.509380	-1098.367360
conformer_78	-1100.113209	0.260746	0.195584	-1100.153561	-105.9	-1099.892815	-1099.954965	24.5	-1094.012342	-4.512374	-1098.366472
conformer_24	-1100.111051	0.260513	0.193276	-1100.153464	-111.4	-1099.892951	-1099.957176	18.7	-1094.014457	-4.507666	-1098.368247
conformer_45	-1100.110166	0.260652	0.194543	-1100.153392	-113.5	-1099.892740	-1099.955837	22.2	-1094.010277	-4.509195	-1098.365143
conformer_77	-1100.109784	0.260416	0.193307	-1100.153217	-114.0	-1099.892801	-1099.956898	19.5	-1094.012753	-4.507921	-1098.367788
conformer_62	-1100.110228	0.260616	0.193243	-1100.151673	-108.8	-1099.891057	-1099.955418	23.3	-1094.016560	-4.505216	-1098.366967
conformer_35	-1100.109623	0.260774	0.193806	-1100.149767	-105.4	-1099.888993	-1099.952949	29.8	-1094.015187	-4.505772	-1098.364285

[a]: Single-point calculation in aqueous phase with SMD model.

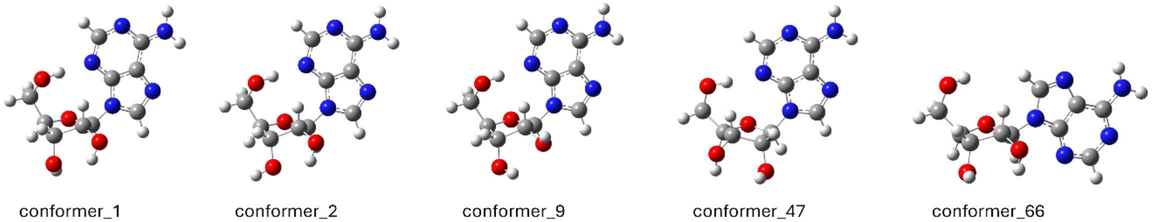

Figure S46. B3LYP-D3/def2-TZVPP optimized geometries of conformers for adenosine (**9rbA**).

Table S44. Conformers of gas-phase optimized adenosine (**9rbA**) at the B3LYP-D3/def2-TZVPP level of theory followed by aqueous phase single-point calculation. The columns display total energy without zero-point correction (E_{Tot}), Gibbs free energy (δG), total energy without zero-point correction ($E_{Tot,W}$), Gibbs free energy ($G_{298,W}$) in water (W), total single-point energy (E_{CBS}) calculated at DLPNO-CCSD(T)/CBS level of theory, and their corresponding free energy G_{CBS}. $G_{298,W}$ and G_{CBS} have been corrected to the standard state of 1 mol/L by addition of +7.908 kJ/mol. ΔG_{Solv} represents the Gibbs free energy of solvation. The data are arranged in the ascending numeric order of $E_{Tot,W}$. $\Delta G_{298,W}$ represents the respective energy difference to the lowest structure. Only conformers within the 24 kJ/mol (6 kcal/mol) energy window above the lowest in CREST are included in initial conformer sampling. Duplicates of the same structure are excluded.

	B3LYP-D3/def2-TZVPP			SMD(H2O)/B3LYP-D3/def2-TZVPP[a]					DLPNO-CCSD(T)/CBS		
9rbA No.	E_{Tot} (Hartree)	δH (Hartree)	δG (Hartree)	$E_{Tot,W}$ (Hartree)	ΔG_{Solv} (kJ/mol)	$H_{298,W}$ (Hartree)	$G_{298,W}$ (Hartree)	$\Delta G_{298,W}$ (kJ/mol)	$E_{CBS,HF}$ (Hartree)	$E_{CBS,C}$ (Hartree)	$G_{CBS,W}$ (Hartree)
conformer_1	-963.973397	0.269056	0.208337	-964.012956	-103.9	-963.743900	-963.801607	0.4	-958.374775	-4.133852	-962.336836
conformer_9	-963.975529	0.269187	0.208672	-964.012923	-98.2	-963.743736	-963.801239	1.4	-958.379083	-4.132597	-962.337390
**conformer_2**	**-963.974987**	**0.269013**	**0.208045**	**-964.012821**	**-99.3**	**-963.743808**	**-963.801764**	**0.0**	**-958.378397**	**-4.132508**	**-962.337683**
conformer_47	-963.969080	0.268992	0.206977	-964.008546	-103.6	-963.739554	-963.798557	8.4	-958.372979	-4.131756	-962.334212
conformer_66	-963.963456	0.269045	0.204913	-964.008210	-117.5	-963.739165	-963.800285	3.9	-958.373438	-4.127194	-962.337460
conformer_42	-963.965875	0.268796	0.207570	-964.008069	-110.8	-963.739273	-963.797487	11.2	-958.367925	-4.133306	-962.332842
conformer_5	-963.971403	0.268927	0.207503	-964.007910	-95.8	-963.738983	-963.797395	11.5	-958.375197	-4.132450	-962.333639
conformer_39	-963.967561	0.268747	0.206529	-964.007842	-105.8	-963.739095	-963.798301	9.1	-958.371660	-4.131759	-962.334159
conformer_82	-963.961587	0.268833	0.205142	-964.007828	-121.4	-963.738995	-963.799674	5.5	-958.369745	-4.128618	-962.336451
conformer_71	-963.964862	0.268921	0.206276	-964.007599	-112.2	-963.738678	-963.798311	9.1	-958.372481	-4.129303	-962.335232
conformer_11	-963.966299	0.268966	0.207636	-964.007506	-108.2	-963.738540	-963.796858	12.9	-958.369036	-4.133337	-962.332933
conformer_43	-963.965854	0.269070	0.206060	-964.007377	-109.0	-963.738307	-963.798305	9.1	-958.374386	-4.128373	-962.335210
conformer_4	-963.973802	0.269023	0.207113	-964.007309	-88.0	-963.738286	-963.797184	12.0	-958.378611	-4.131815	-962.333808
conformer_25	-963.966782	0.268897	0.207263	-964.007266	-106.3	-963.738369	-963.796991	12.5	-958.371803	-4.131753	-962.333765
conformer_79	-963.960074	0.268671	0.204322	-964.006937	-123.0	-963.738266	-963.799603	5.7	-958.367908	-4.128819	-962.336255
conformer_55	-963.962961	0.268992	0.205898	-964.006684	-114.8	-963.737692	-963.797774	10.5	-958.368671	-4.130406	-962.333890
conformer_7	-963.971673	0.268943	0.206909	-964.006260	-90.8	-963.737317	-963.796339	14.2	-958.377539	-4.130968	-962.333173
conformer_17	-963.970412	0.268831	0.206439	-964.006073	-93.6	-963.737242	-963.796622	13.5	-958.375419	-4.131326	-962.332955
conformer_81	-963.958173	0.268700	0.204534	-964.005875	-125.2	-963.737175	-963.798329	9.0	-958.367747	-4.127103	-962.335005
conformer_75	-963.965329	0.268906	0.206236	-964.005804	-106.3	-963.736898	-963.796556	13.7	-958.374755	-4.127910	-962.333892
conformer_6	-963.970371	0.268924	0.206549	-964.005556	-92.4	-963.736632	-963.795995	15.1	-958.376976	-4.130087	-962.332687
conformer_8	-963.969561	0.268813	0.206431	-964.005553	-94.5	-963.736740	-963.796110	14.8	-958.375640	-4.130492	-962.332680
conformer_84	-963.961664	0.268916	0.206542	-964.005503	-115.1	-963.736587	-963.795949	15.3	-958.369628	-4.128584	-962.332497
conformer_16	-963.968684	0.268800	0.206129	-964.005451	-96.5	-963.736651	-963.796310	14.3	-958.375233	-4.130131	-962.332990
conformer_19	-963.965022	0.268996	0.205416	-964.005345	-105.9	-963.736349	-963.796917	12.7	-958.374197	-4.128007	-962.334099
conformer_63	-963.965453	0.268880	0.205642	-964.005171	-104.3	-963.736291	-963.796517	13.8	-958.374095	-4.129061	-962.334220
conformer_10	-963.971788	0.268870	0.205934	-964.005170	-87.6	-963.736300	-963.796224	14.5	-958.378098	-4.130774	-962.333308
conformer_60	-963.960800	0.268742	0.205082	-964.004838	-115.6	-963.736096	-963.796744	13.2	-958.370035	-4.127961	-962.333941
conformer_86	-963.965649	0.268847	0.205793	-964.004776	-102.7	-963.735929	-963.795971	15.2	-958.375979	-4.127347	-962.333649
conformer_73	-963.968129	0.268731	0.205991	-964.004727	-96.1	-963.735996	-963.795724	15.9	-958.375252	-4.129998	-962.332846

conformer_89	-963.965639	0.268820	0.205246	-964.004706	-102.6	-963.735886	-963.796448	14.0	-958.375952	-4.127270	-962.334031
conformer_65	-963.959706	0.268652	0.206605	-964.004657	-118.0	-963.736005	-963.795040	17.7	-958.365653	-4.131527	-962.332514
conformer_31	-963.969647	0.268874	0.206413	-964.004424	-91.3	-963.735550	-963.794999	17.8	-958.376906	-4.129752	-962.332010
conformer_12	-963.966295	0.268913	0.205548	-964.004412	-100.1	-963.735499	-963.795852	15.5	-958.374904	-4.128835	-962.333295
conformer_76	-963.961133	0.269011	0.206000	-964.003961	-112.4	-963.734950	-963.794949	17.9	-958.367515	-4.130577	-962.331909
conformer_28	-963.964055	0.269098	0.206117	-964.003871	-104.5	-963.734773	-963.794742	18.4	-958.372447	-4.128533	-962.331668
conformer_53	-963.962377	0.268675	0.205337	-964.003847	-108.9	-963.735172	-963.795498	16.5	-958.371067	-4.128750	-962.332937
conformer_67	-963.963483	0.268822	0.205624	-964.003831	-105.9	-963.735009	-963.795195	17.2	-958.372793	-4.128292	-962.332797
conformer_36	-963.963237	0.268786	0.205707	-964.003798	-106.5	-963.735012	-963.795079	17.6	-958.372559	-4.128182	-962.332583
conformer_32	-963.962692	0.268877	0.206805	-964.003764	-107.8	-963.734887	-963.793947	20.5	-958.365006	-4.134334	-962.330596
conformer_15	-963.961623	0.268854	0.206119	-964.003636	-110.3	-963.734782	-963.794505	19.1	-958.368356	-4.130068	-962.331306
conformer_90	-963.955938	0.268796	0.204589	-964.002933	-123.4	-963.734137	-963.795332	16.9	-958.361542	-4.131246	-962.332182
conformer_30	-963.964044	0.268842	0.205772	-964.002803	-101.8	-963.733961	-963.794019	20.3	-958.374023	-4.127682	-962.331681
conformer_26	-963.960973	0.268754	0.204055	-964.002777	-109.8	-963.734023	-963.795710	15.9	-958.367720	-4.130042	-962.332500
conformer_64	-963.960563	0.269015	0.208021	-964.002718	-110.7	-963.733703	-963.791685	26.5	-958.361574	-4.135778	-962.328474
conformer_21	-963.964052	0.268916	0.205997	-964.002489	-100.9	-963.733573	-963.793480	21.7	-958.371900	-4.129257	-962.330585
conformer_27	-963.961930	0.268833	0.205725	-964.002397	-106.2	-963.733564	-963.793660	21.3	-958.369425	-4.129655	-962.330809
conformer_14	-963.961169	0.268819	0.205743	-964.002374	-108.2	-963.733555	-963.793619	21.4	-958.367906	-4.129804	-962.330160
conformer_72	-963.960095	0.268957	0.205756	-964.002343	-110.9	-963.733386	-963.793575	21.5	-958.366921	-4.129883	-962.330284
conformer_41	-963.963474	0.268846	0.205195	-964.002328	-102.0	-963.733482	-963.794121	20.1	-958.372036	-4.128571	-962.331255
conformer_22	-963.966187	0.268874	0.204714	-964.002229	-94.6	-963.733355	-963.794503	19.1	-958.374941	-4.128642	-962.331900
conformer_58	-963.958872	0.268904	0.205797	-964.002085	-113.5	-963.733181	-963.793276	22.3	-958.364832	-4.131331	-962.330566
conformer_20	-963.961415	0.268879	0.205170	-964.001841	-106.1	-963.732962	-963.793659	21.3	-958.365443	-4.133138	-962.330826
conformer_77	-963.960183	0.268932	0.204875	-964.001310	-108.0	-963.732378	-963.793423	21.9	-958.367668	-4.129121	-962.330029
conformer_18	-963.966836	0.268910	0.204016	-964.000825	-89.2	-963.731915	-963.793797	20.9	-958.376088	-4.128525	-962.331574
conformer_57	-963.960359	0.268785	0.206623	-963.999923	-103.9	-963.731138	-963.790288	30.1	-958.363879	-4.133977	-962.327786
conformer_38	-963.958127	0.268820	0.205618	-963.999898	-109.7	-963.731078	-963.791268	27.6	-958.361957	-4.133540	-962.328639

[a]: Single-point calculation in aqueous phase with SMD model.

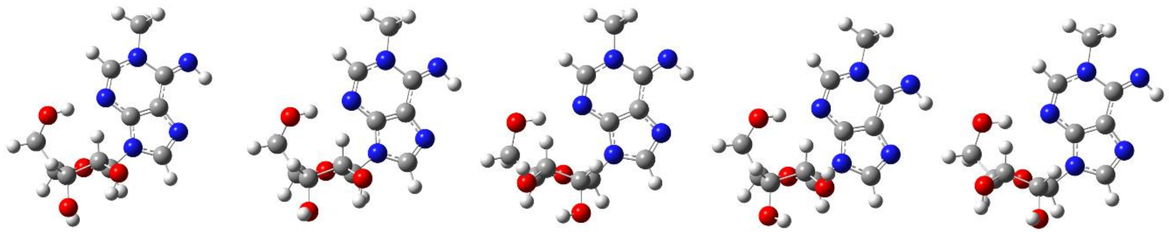

Figure S47. B3LYP-D3/def2-TZVPP optimized geometries of conformers for neutral *N*[1]-methyladenosine (**9rb1mA**).

Table S45. Conformers of gas-phase optimized neutral *N*[1]-methyladenosine (**9rb1mA**) at the B3LYP-D3/def2-TZVPP level of theory followed by aqueous phase single-point calculation. The columns display total energy without zero-point correction (E_{Tot}), Gibbs free energy (δG), total energy without zero-point correction ($E_{Tot,W}$), Gibbs free energy ($G_{298,W}$) in water (W), total single-point energy (E_{CBS}) calculated at DLPNO-CCSD(T)/CBS level of theory, and their corresponding free energy G_{CBS}. $G_{298,W}$ and G_{CBS} have been corrected to the standard state of 1 mol/L by addition of +7.908 kJ/mol. ΔG_{Solv} represents the Gibbs free energy of solvation. The data are arranged in the ascending numeric order of $E_{Tot,W}$. $\Delta G_{298,W}$ represents the respective energy difference to the lowest structure. Only conformers within the 24 kJ/mol (6 kcal/mol) energy window above the lowest in CREST are included in initial conformer sampling. Duplicates of the same structure are excluded.

9rb1mA No.	B3LYP-D3/def2-TZVPP E_{Tot} (Hartree)	δH (Hartree)	δG (Hartree)	SMD(H_2O)/B3LYP-D3/def2-TZVPP[a] $E_{Tot,W}$ (Hartree)	ΔG_{Solv} (kJ/mol)	$H_{298,W}$ (Hartree)	$G_{298,W}$ (Hartree)	$\Delta G_{298,W}$ (kJ/mol)	DLPNO-CCSD(T)/CBS $E_{CBS,HF}$ (Hartree)	$E_{CBS,C}$ (Hartree)	$G_{CBS,W}$ (Hartree)
**conformer_3**	**-1003.280596**	**0.298837**	**0.235148**	**-1003.319621**	**-102.5**	**-1003.020784**	**-1003.081461**	**0.0**	**-997.396958**	**-4.346969**	**-1001.544792**
conformer_1	-1003.279368	0.298730	0.235732	-1003.319149	-104.4	-1003.020419	-1003.080405	2.8	-997.394232	-4.347993	-1001.543263
conformer_13	-1003.281526	0.298854	0.235869	-1003.319026	-98.5	-1003.020172	-1003.080145	3.5	-997.398440	-4.346864	-1001.543923
conformer_75	-1003.275952	0.298800	0.234883	-1003.314917	-102.3	-1003.016117	-1003.077022	11.7	-997.393233	-4.345917	-1001.540219
conformer_10	-1003.277817	0.298597	0.234782	-1003.314293	-95.8	-1003.015696	-1003.076499	13.0	-997.394862	-4.346766	-1001.540310
conformer_50	-1003.274825	0.298455	0.234376	-1003.314203	-103.4	-1003.015748	-1003.076815	12.2	-997.392288	-4.346030	-1001.540307
conformer_91	-1003.272666	0.298703	0.233806	-1003.314014	-108.6	-1003.015311	-1003.077196	11.2	-997.394172	-4.342804	-1001.541507
conformer_5	-1003.279454	0.298743	0.234553	-1003.313502	-89.4	-1003.014759	-1003.075937	14.5	-997.397303	-4.346151	-1001.539937
conformer_20	-1003.272497	0.298557	0.234612	-1003.313396	-107.4	-1003.014839	-1003.075772	14.9	-997.388660	-4.347588	-1001.539524
conformer_45	-1003.273099	0.298495	0.234483	-1003.313353	-105.7	-1003.014858	-1003.075858	14.7	-997.391552	-4.346002	-1001.540314
conformer_16	-1003.276972	0.298664	0.234173	-1003.312286	-92.7	-1003.013622	-1003.075101	16.7	-997.396090	-4.345280	-1001.539500
conformer_40	-1003.271707	0.298646	0.232935	-1003.312076	-106.0	-1003.013430	-1003.076129	14.0	-997.394081	-4.342109	-1001.540612
conformer_66	-1003.275657	0.298458	0.233793	-1003.311838	-95.0	-1003.013380	-1003.075033	16.9	-997.393846	-4.345687	-1001.538909
conformer_61	-1003.273965	0.298544	0.233711	-1003.311621	-98.9	-1003.013077	-1003.074898	17.2	-997.393661	-4.344538	-1001.539132
conformer_12	-1003.275851	0.298679	0.234184	-1003.311554	-93.7	-1003.012875	-1003.074358	18.7	-997.395624	-4.344429	-1001.538559
conformer_93	-1003.271351	0.298569	0.232305	-1003.311361	-105.0	-1003.012792	-1003.076044	14.2	-997.395273	-4.340882	-1001.540848
conformer_28	-1003.272424	0.298552	0.233152	-1003.311085	-101.5	-1003.012533	-1003.074921	17.2	-997.394197	-4.343057	-1001.539752
conformer_22	-1003.277444	0.298555	0.233671	-1003.311077	-88.3	-1003.012522	-1003.074394	18.6	-997.396820	-4.345228	-1001.538999
conformer_99	-1003.270851	0.298409	0.231909	-1003.311044	-105.5	-1003.012635	-1003.076123	14.0	-997.393590	-4.341996	-1001.540858
conformer_62	-1003.270691	0.298747	0.233399	-1003.310614	-104.8	-1003.011867	-1003.074203	19.1	-997.392187	-4.342706	-1001.538405
conformer_63	-1003.269943	0.298432	0.233019	-1003.310535	-106.6	-1003.012103	-1003.074504	18.3	-997.392499	-4.342226	-1001.539286
conformer_89	-1003.275176	0.298608	0.233914	-1003.310426	-92.5	-1003.011818	-1003.073500	20.9	-997.395596	-4.344079	-1001.538000
conformer_34	-1003.270005	0.298559	0.234073	-1003.310252	-105.7	-1003.011693	-1003.073167	21.8	-997.385193	-4.348759	-1001.537114
conformer_23	-1003.268309	0.298530	0.233385	-1003.310152	-109.9	-1003.011622	-1003.073755	20.2	-997.388162	-4.344385	-1001.537994
conformer_56	-1003.267311	0.298408	0.232525	-1003.309847	-111.7	-1003.011439	-1003.074310	18.8	-997.387253	-4.344175	-1001.538428
conformer_86	-1003.270369	0.298489	0.233065	-1003.309575	-102.9	-1003.011086	-1003.073498	20.9	-997.393637	-4.341715	-1001.538481
conformer_48	-1003.267111	0.298516	0.232920	-1003.309366	-110.9	-1003.010850	-1003.073434	21.1	-997.387280	-4.343813	-1001.537415
conformer_81	-1003.270298	0.298529	0.232986	-1003.309104	-101.9	-1003.010575	-1003.073106	21.9	-997.392004	-4.342730	-1001.537542
conformer_41	-1003.267962	0.298546	0.233526	-1003.309102	-108.0	-1003.010556	-1003.072564	23.4	-997.387820	-4.343982	-1001.536404

conformer_65	-1003.268152	0.298452	0.232807	-1003.308942	-107.1	-1003.010490	-1003.073123	21.9	-997.388871	-4.343850	-1001.537692
conformer_33	-1003.272835	0.298571	0.232691	-1003.308933	-94.8	-1003.010362	-1003.073230	21.6	-997.394726	-4.342948	-1001.538068
conformer_31	-1003.268100	0.298557	0.233376	-1003.308242	-105.4	-1003.009685	-1003.071854	25.2	-997.385187	-4.347549	-1001.536489
conformer_54	-1003.273232	0.298549	0.232621	-1003.307869	-90.9	-1003.009320	-1003.072236	24.2	-997.395942	-4.342567	-1001.537513
conformer_55	-1003.265142	0.298571	0.233507	-1003.306205	-107.8	-1003.007634	-1003.069686	30.9	-997.381719	-4.348134	-1001.534398
conformer_32	-1003.265842	0.298507	0.231867	-1003.305542	-104.2	-1003.007035	-1003.070663	28.4	-997.383558	-4.346801	-1001.535180
conformer_29	-1003.265850	0.298562	0.232060	-1003.305538	-104.2	-1003.006976	-1003.070466	28.9	-997.383597	-4.346851	-1001.535064

[a]: Single-point calculation in aqueous phase with SMD model.

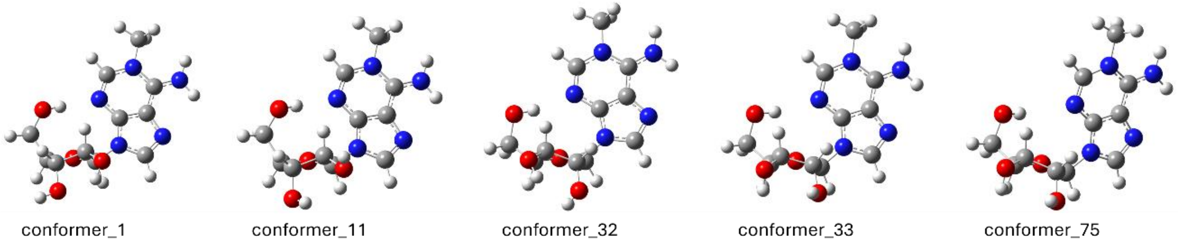

Figure S48. B3LYP-D3/def2-TZVPP optimized geometries of conformers for protonated N^1-methyladenosine cation (**9rb1mA⁺**).

Table S46. Conformers of gas-phase optimized protonated N^1-methyladenosine cation (**9rb1mA$^+$**) at the B3LYP-D3/def2-TZVPP level of theory followed by aqueous phase single-point calculation. The columns display total energy without zero-point correction (E_{Tot}), Gibbs free energy (δG), total energy without zero-point correction ($E_{Tot,W}$), Gibbs free energy ($G_{298,W}$) in water (W), total single-point energy (E_{CBS}) calculated at DLPNO-CCSD(T)/CBS level of theory, and their corresponding free energy G_{CBS}. $G_{298,W}$ and G_{CBS} have been corrected to the standard state of 1 mol/L by addition of +7.908 kJ/mol. ΔG_{Solv} represents the Gibbs free energy of solvation. The data are arranged in the ascending numeric order of $E_{Tot,W}$. $\Delta G_{298,W}$ represents the respective energy difference to the lowest structure. Only conformers within the 24 kJ/mol (6 kcal/mol) energy window above the lowest in CREST are included in initial conformer sampling. Duplicates of the same structure are excluded.

9rb1mA$^+$ No.	B3LYP-D3/def2-TZVPP E_{Tot} (Hartree)	δH (Hartree)	δG (Hartree)	SMD(H₂O)/B3LYP-D3/def2-TZVPP[a] $E_{Tot,W}$ (Hartree)	ΔG_{Solv} (kJ/mol)	$H_{298,W}$ (Hartree)	$G_{298,W}$ (Hartree)	$\Delta G_{298,W}$ (kJ/mol)	DLPNO-CCSD(T)/CBS $E_{CBS,HF}$ (Hartree)	$E_{CBS,C}$ (Hartree)	$G_{CBS,W}$ (Hartree)
conformer_11	-1003.678181	0.31198	0.247957	-1003.789057	-291.1	-1003.477077	-1003.538088	0.1	-997.805187	-4.331468	-1001.996561
**conformer_1**	**-1003.680379**	**0.311991**	**0.247877**	**-1003.789029**	**-285.3**	**-1003.477038**	**-1003.538140**	**0.0**	**-997.807155**	**-4.331489**	**-1001.996405**
conformer_33	-1003.672423	0.311879	0.245769	-1003.785382	-296.6	-1003.473503	-1003.536601	4.0	-997.799894	-4.330582	-1001.994655
conformer_70	-1003.672399	0.311768	0.245219	-1003.784659	-294.7	-1003.472891	-1003.536428	4.5	-997.801787	-4.329224	-1001.995040
conformer_32	-1003.667636	0.31171	0.246847	-1003.784616	-307.1	-1003.472906	-1003.534757	8.9	-997.792857	-4.332287	-1001.992264
conformer_9	-1003.67457	0.311705	0.246059	-1003.784376	-288.3	-1003.472671	-1003.535305	7.4	-997.801661	-4.331285	-1001.993680
conformer_22	-1003.66626	0.311403	0.244988	-1003.784317	-310.0	-1003.472914	-1003.536317	4.8	-997.795106	-4.329198	-1001.994360
conformer_35	-1003.669852	0.311729	0.245359	-1003.784162	-300.1	-1003.472433	-1003.535791	6.2	-997.800958	-4.327618	-1001.994514
conformer_45	-1003.674366	0.311546	0.24483	-1003.783664	-287.0	-1003.472118	-1003.535822	6.1	-997.803379	-4.329796	-1001.994630
conformer_19	-1003.673686	0.311646	0.245971	-1003.783384	-288.0	-1003.471738	-1003.534401	9.8	-997.802923	-4.329746	-1001.993384
conformer_23	-1003.670434	0.311501	0.245113	-1003.783254	-296.2	-1003.471753	-1003.535129	7.9	-997.801117	-4.328370	-1001.994182
conformer_31	-1003.674336	0.311607	0.245286	-1003.782658	-284.4	-1003.471051	-1003.534360	9.9	-997.805844	-4.327569	-1001.993436
conformer_12	-1003.669700	0.311595	0.245940	-1003.782593	-296.4	-1003.470998	-1003.533641	11.8	-997.798752	-4.329606	-1001.992299
conformer_68	-1003.672272	0.311876	0.246310	-1003.782464	-289.3	-1003.470588	-1003.533142	13.1	-997.798445	-4.332219	-1001.991534
conformer_18	-1003.668908	0.311528	0.245105	-1003.782441	-298.1	-1003.470913	-1003.534324	10.0	-997.799608	-4.327560	-1001.992584
conformer_60	-1003.669227	0.311826	0.245638	-1003.782424	-297.2	-1003.470598	-1003.533774	11.5	-997.799628	-4.328068	-1001.992243
conformer_21	-1003.665958	0.311504	0.245401	-1003.782393	-305.7	-1003.470889	-1003.533980	10.9	-997.794567	-4.329136	-1001.991725
conformer_3	-1003.677939	0.311605	0.246203	-1003.782259	-273.9	-1003.470654	-1003.533044	13.4	-997.806421	-4.330302	-1001.991828
conformer_57	-1003.672360	0.311481	0.246306	-1003.781864	-287.5	-1003.470383	-1003.532546	14.7	-997.800782	-4.330113	-1001.991081
conformer_72	-1003.672041	0.311596	0.245315	-1003.781732	-288.0	-1003.470136	-1003.533405	12.4	-997.798365	-4.332296	-1001.992025
conformer_14	-1003.671340	0.311644	0.246080	-1003.781663	-289.7	-1003.470019	-1003.532571	14.6	-997.800957	-4.328973	-1001.991161
conformer_80	-1003.674531	0.311602	0.245012	-1003.781565	-281.0	-1003.469963	-1003.533541	12.1	-997.807040	-4.326883	-1001.992935
conformer_36	-1003.664800	0.311655	0.245628	-1003.781234	-305.7	-1003.469579	-1003.532594	14.6	-997.793471	-4.329294	-1001.990560
conformer_100	-1003.663579	0.311499	0.245154	-1003.780677	-307.4	-1003.469178	-1003.532511	14.8	-997.787498	-4.334576	-1001.991006
conformer_51	-1003.674376	0.311599	0.244980	-1003.780402	-278.4	-1003.468803	-1003.532410	15.0	-997.805284	-4.328437	-1001.991755
conformer_63	-1003.670929	0.311557	0.245759	-1003.780384	-287.4	-1003.468827	-1003.531613	17.1	-997.801155	-4.328638	-1001.990476
conformer_28	-1003.672432	0.311785	0.246918	-1003.779734	-281.7	-1003.467949	-1003.529804	21.9	-997.797335	-4.333715	-1001.988421
conformer_85	-1003.674856	0.311633	0.245430	-1003.779679	-275.2	-1003.468046	-1003.531237	18.1	-997.806591	-4.327998	-1001.990969

[a]: Single-point calculation in aqueous phase with SMD model.

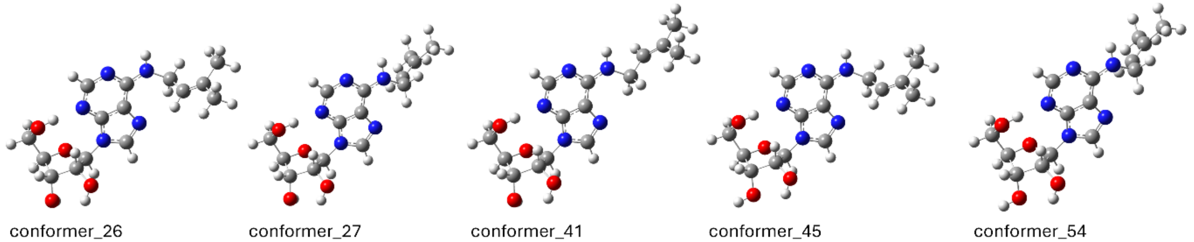

Figure S49. B3LYP-D3/def2-TZVPP optimized geometries of conformers for *N*[6]-isoprenyladenosine (**9rb6iPA**).

Table S47. Conformers of gas-phase optimized *N*[6]-isoprenyladenosine (**9rb6iPA**) at the B3LYP-D3/def2-TZVPP level of theory followed by aqueous phase single-point calculation. The columns display total energy without zero-point correction (E_{Tot}), Gibbs free energy (δG), total energy without zero-point correction ($E_{Tot,W}$), Gibbs free energy ($G_{298,W}$) in water (W), total single-point energy (E_{CBS}) calculated at DLPNO-CCSD(T)/CBS level of theory, and their corresponding free energy G_{CBS}. $G_{298,W}$ and G_{CBS} have been corrected to the standard state of 1 mol/L by addition of +7.908 kJ/mol. ΔG_{Solv} represents the Gibbs free energy of solvation. The data are arranged in the ascending numeric order of $E_{Tot,W}$. $\Delta G_{298,W}$ represents the respective energy difference to the lowest structure. Only conformers within the 24 kJ/mol (6 kcal/mol) energy window above the lowest in CREST are included in initial conformer sampling. Duplicates of the same structure are excluded.

9rb6iPA	B3LYP-D3/def2-TZVPP			SMD(H2O)/B3LYP-D3/def2-TZVPP[a]					DLPNO-CCSD(T)/CBS		
No.	E_{Tot} (Hartree)	δH (Hartree)	δG (Hartree)	$E_{Tot,W}$ (Hartree)	ΔG_{Solv} (kJ/mol)	$H_{298,W}$ (Hartree)	$G_{298,W}$ (Hartree)	$\Delta G_{298,W}$ (kJ/mol)	$E_{CBS,HF}$ (Hartree)	$E_{CBS,C}$ (Hartree)	$G_{CBS,W}$ (Hartree)
conformer_27	-1159.393204	0.393385	0.316412	-1159.428896	-93.7	-1159.035511	-1159.109472	3.2	-1152.430254	-5.133456	-1157.279977
conformer_26	-1159.393273	0.393367	0.315985	-1159.428820	-93.3	-1159.035453	-1159.109823	2.3	-1152.430356	-5.133478	-1157.280384
conformer_45	-1159.394815	0.393318	0.315781	-1159.428741	-89.1	-1159.035423	-1159.109948	2.0	-1152.433905	-5.132110	-1157.281148
conformer_54	-1159.394779	0.393350	0.316227	-1159.428730	-89.1	-1159.035380	-1159.109491	3.2	-1152.433839	-5.132111	-1157.280662
**conformer_41**	**-1159.393048**	**0.393246**	**0.314900**	**-1159.428604**	**-93.4**	**-1159.035358**	**-1159.110692**	**0.0**	**-1152.431364**	**-5.132154**	**-1157.281163**
conformer_55	-1159.394597	0.393225	0.314913	-1159.428464	-88.9	-1159.035239	-1159.110539	0.4	-1152.434917	-5.130805	-1157.281665
conformer_36	-1159.393061	0.393360	0.315476	-1159.428217	-92.3	-1159.034857	-1159.109729	2.5	-1152.430960	-5.132339	-1157.279967
conformer_20	-1159.394745	0.393450	0.315419	-1159.427954	-87.2	-1159.034504	-1159.109523	3.1	-1152.431957	-5.133783	-1157.280518
conformer_1	-1159.393793	0.393367	0.316283	-1159.427797	-89.3	-1159.034430	-1159.108502	5.7	-1152.429452	-5.134811	-1157.278973
conformer_5	-1159.395387	0.393398	0.316471	-1159.427726	-84.9	-1159.034328	-1159.108243	6.4	-1152.433035	-5.133499	-1157.279389
conformer_3	-1159.393635	0.393404	0.316910	-1159.427593	-89.2	-1159.034189	-1159.107671	7.9	-1152.429359	-5.134823	-1157.278217
conformer_7	-1159.395238	0.393393	0.316914	-1159.427515	-84.7	-1159.034122	-1159.107589	8.1	-1152.432970	-5.133467	-1157.278788
conformer_100	-1159.393646	0.393342	0.315618	-1159.425334	-83.2	-1159.031992	-1159.106704	10.5	-1152.433244	-5.132190	-1157.278493
conformer_79	-1159.391733	0.393355	0.316152	-1159.422854	-81.7	-1159.029499	-1159.103690	18.4	-1152.429824	-5.133500	-1157.275280
conformer_76	-1159.391798	0.393329	0.316329	-1159.422792	-81.4	-1159.029463	-1159.103451	19.0	-1152.429898	-5.133430	-1157.274982
conformer_12	-1159.394390	0.393342	0.315665	-1159.422521	-73.9	-1159.029179	-1159.103844	18.0	-1152.433393	-5.132797	-1157.275644
conformer_14	-1159.394427	0.393396	0.315823	-1159.422482	-73.7	-1159.029086	-1159.103647	18.5	-1152.433480	-5.132811	-1157.275512
conformer_88	-1159.392367	0.393289	0.315584	-1159.421291	-75.9	-1159.028002	-1159.102695	21.0	-1152.432426	-5.132056	-1157.274809
conformer_82	-1159.392333	0.393271	0.315637	-1159.421263	-76.0	-1159.027992	-1159.102614	21.2	-1152.432540	-5.131879	-1157.274700
conformer_66	-1159.390968	0.393189	0.314994	-1159.420541	-77.6	-1159.027352	-1159.102535	21.4	-1152.431966	-5.130887	-1157.274420
conformer_89	-1159.390258	0.393167	0.315065	-1159.420514	-79.4	-1159.027347	-1159.102437	21.7	-1152.430686	-5.131297	-1157.274162
conformer_86	-1159.390228	0.393124	0.315186	-1159.420509	-79.5	-1159.027385	-1159.102311	22.0	-1152.430673	-5.131424	-1157.274180
conformer_52	-1159.391002	0.393261	0.315121	-1159.420502	-77.5	-1159.027241	-1159.102369	21.9	-1152.431970	-5.130943	-1157.274280

[a]: Single-point calculation in aqueous phase with SMD model.

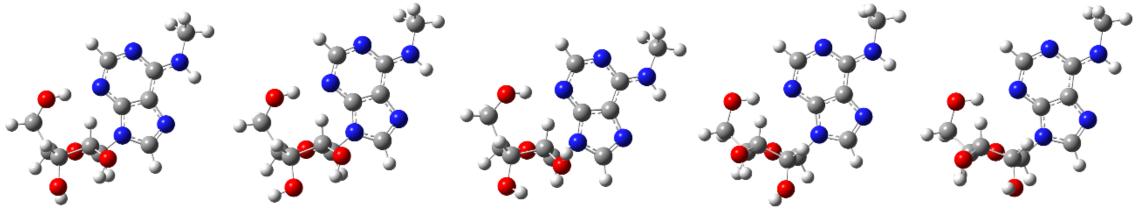

Figure S50. B3LYP-D3/def2-TZVPP optimized geometries of conformers for *N*[6]-methyladenosine (**9rb6mA**).

Table S48. Conformers of gas-phase optimized *N*[6]-methyladenosine (**9rb6mA**) at the B3LYP-D3/def2-TZVPP level of theory followed by aqueous phase single-point calculation. The columns display total energy without zero-point correction (E_{Tot}), Gibbs free energy (δG), total energy without zero-point correction ($E_{Tot,W}$), Gibbs free energy ($G_{298,W}$) in water (W), total single-point energy (E_{CBS}) calculated at DLPNO-CCSD(T)/CBS level of theory, and their corresponding free energy G_{CBS}. $G_{298,W}$ and G_{CBS} have been corrected to the standard state of 1 mol/L by addition of +7.908 kJ/mol. ΔG_{Solv} represents the Gibbs free energy of solvation. The data are arranged in the ascending numeric order of $E_{Tot,W}$. $\Delta G_{298,W}$ represents the respective energy difference to the lowest structure. Only conformers within the 24 kJ/mol (6 kcal/mol) energy window above the lowest in CREST are included in initial conformer sampling. Duplicates of the same structure are excluded.

**9rb6mA**	**B3LYP-D3/def2-TZVPP**			**SMD(H₂O)/B3LYP-D3/def2-TZVPP**[a]					**DLPNO-CCSD(T)/CBS**		
**No.**	**E_{Tot}** (Hartree)	**δH** (Hartree)	**δG** (Hartree)	**$E_{Tot,W}$** (Hartree)	**ΔG_{Solv}** (kJ/mol)	**$H_{298,W}$** (Hartree)	**$G_{298,W}$** (Hartree)	**$\Delta G_{298,W}$** (kJ/mol)	**$E_{CBS,HF}$** (Hartree)	**$E_{CBS,C}$** (Hartree)	**$G_{CBS,W}$** (Hartree)
conformer_9	-1003.299009	0.298998	0.234741	-1003.334234	-92.5	-1003.035236	-1003.096481	0.5	-997.421106	-4.339985	-1001.558564
conformer_1	-1003.296919	0.298895	0.234601	-1003.334227	-98.0	-1003.035332	-1003.096614	0.2	-997.416828	-4.341272	-1001.557795
**conformer_2**	**-1003.298459**	**0.298857**	**0.234414**	**-1003.334104**	**-93.6**	**-1003.035247**	**-1003.096678**	**0.0**	**-997.420406**	**-4.339910**	**-1001.558534**
conformer_49	-1003.292589	0.298872	0.233331	-1003.329928	-98.0	-1003.031056	-1003.093585	8.1	-997.414999	-4.339160	-1001.555155
conformer_47	-1003.289417	0.298615	0.233494	-1003.329376	-104.9	-1003.030761	-1003.092870	10.0	-997.410033	-4.340722	-1001.554209
conformer_5	-1003.294887	0.298788	0.233535	-1003.329324	-90.4	-1003.030536	-1003.092777	10.2	-997.417250	-4.339786	-1001.554925
conformer_73	-1003.286750	0.298863	0.230886	-1003.329255	-111.6	-1003.030392	-1003.095357	3.5	-997.415336	-4.334409	-1001.558353
conformer_40	-1003.291067	0.298574	0.232647	-1003.329222	-100.2	-1003.030648	-1003.093563	8.2	-997.413739	-4.339128	-1001.555363
conformer_87	-1003.284879	0.298601	0.230733	-1003.328931	-115.7	-1003.030330	-1003.095186	3.9	-997.411615	-4.335880	-1001.557801
conformer_76	-1003.288175	0.298761	0.232106	-1003.328840	-106.8	-1003.030079	-1003.093722	7.8	-997.414331	-4.336535	-1001.556413
conformer_12	-1003.289787	0.298785	0.233453	-1003.328671	-102.1	-1003.029886	-1003.092206	11.7	-997.411101	-4.340759	-1001.554279
conformer_3	-1003.297314	0.298854	0.233098	-1003.328492	-81.9	-1003.029638	-1003.092382	11.3	-997.420696	-4.339209	-1001.554973
conformer_26	-1003.290234	0.298725	0.233230	-1003.328473	-100.4	-1003.029748	-1003.092231	11.7	-997.413822	-4.339140	-1001.554960
conformer_50	-1003.289151	0.298821	0.232018	-1003.328415	-103.1	-1003.029594	-1003.093385	8.6	-997.416310	-4.335705	-1001.556249
conformer_83	-1003.283433	0.298554	0.230775	-1003.328092	-117.3	-1003.029538	-1003.094305	6.2	-997.409717	-4.336232	-1001.556821
conformer_62	-1003.286329	0.298839	0.232502	-1003.327919	-109.2	-1003.029080	-1003.092405	11.2	-997.410630	-4.337760	-1001.554465
conformer_7	-1003.295206	0.298763	0.232949	-1003.327392	-84.5	-1003.028629	-1003.091431	13.8	-997.419634	-4.338371	-1001.554230
conformer_18	-1003.293839	0.298685	0.232681	-1003.327325	-87.9	-1003.028640	-1003.091632	13.2	-997.417408	-4.338697	-1001.553898
conformer_82	-1003.288574	0.298702	0.231567	-1003.326974	-100.8	-1003.028272	-1003.092395	11.2	-997.416662	-4.335036	-1001.555519
conformer_85	-1003.281429	0.298483	0.230125	-1003.326909	-119.4	-1003.028426	-1003.093772	7.6	-997.409641	-4.334299	-1001.556283
conformer_6	-1003.293844	0.298759	0.232657	-1003.326751	-86.4	-1003.027992	-1003.091082	14.7	-997.419019	-4.337476	-1001.553732
conformer_8	-1003.293056	0.298637	0.232482	-1003.326706	-88.3	-1003.028069	-1003.091212	14.3	-997.417688	-4.337899	-1001.553743
conformer_16	-1003.292146	0.298624	0.232283	-1003.326649	-90.6	-1003.028025	-1003.091354	14.0	-997.417230	-4.337565	-1001.554004
conformer_19	-1003.288430	0.298859	0.231404	-1003.326531	-100.0	-1003.027672	-1003.092115	12.0	-997.416221	-4.335214	-1001.555119
conformer_11	-1003.295172	0.298720	0.232207	-1003.326370	-81.9	-1003.027650	-1003.091151	14.5	-997.420004	-4.338244	-1001.554227
conformer_10	-1003.295172	0.298720	0.232207	-1003.326370	-81.9	-1003.027650	-1003.091151	14.5	-997.420004	-4.338244	-1001.554227
conformer_66	-1003.284178	0.298577	0.230889	-1003.325981	-109.8	-1003.027404	-1003.092080	12.1	-997.411982	-4.335180	-1001.555064
conformer_80	-1003.291572	0.298550	0.232057	-1003.325917	-90.2	-1003.027367	-1003.090848	15.3	-997.417226	-4.337402	-1001.553904
conformer_74	-1003.291572	0.298550	0.232057	-1003.325917	-90.2	-1003.027367	-1003.090848	15.3	-997.417226	-4.337402	-1001.553904

conformer_32	-1003.293100	0.298707	0.232427	-1003.325624	-85.4	-1003.026917	-1003.090185	17.0	-997.418921	-4.337179	-1001.553185
conformer_33	-1003.289684	0.298737	0.231542	-1003.325439	-93.9	-1003.026702	-1003.090885	15.2	-997.416911	-4.336143	-1001.554255
conformer_13	-1003.289684	0.298737	0.231542	-1003.325439	-93.9	-1003.026702	-1003.090885	15.2	-997.416911	-4.336144	-1001.554255
conformer_30	-1003.287445	0.298887	0.231866	-1003.325096	-98.9	-1003.026209	-1003.090218	17.0	-997.414383	-4.335800	-1001.552956
conformer_71	-1003.286800	0.298619	0.231378	-1003.325028	-100.4	-1003.026409	-1003.090638	15.9	-997.414763	-4.335507	-1001.554109
conformer_35	-1003.286108	0.298695	0.232490	-1003.324982	-102.1	-1003.026287	-1003.089480	18.9	-997.407028	-4.341512	-1001.551912
conformer_41	-1003.286523	0.298554	0.231354	-1003.324957	-100.9	-1003.026403	-1003.090591	16.0	-997.414513	-4.335359	-1001.553940
conformer_59	-1003.285741	0.298473	0.230997	-1003.324892	-102.8	-1003.026419	-1003.090883	15.2	-997.413049	-4.335975	-1001.554167
conformer_84	-1003.284537	0.298770	0.231953	-1003.324854	-105.9	-1003.026084	-1003.089889	17.8	-997.409421	-4.337988	-1001.552762
conformer_78	-1003.284537	0.298770	0.231953	-1003.324854	-105.9	-1003.026084	-1003.089889	17.8	-997.409421	-4.337988	-1001.552762
conformer_17	-1003.284933	0.298615	0.231708	-1003.324789	-104.6	-1003.026174	-1003.090069	17.3	-997.410290	-4.337364	-1001.552791
conformer_60	-1003.281874	0.298690	0.232013	-1003.324137	-111.0	-1003.025447	-1003.089112	19.9	-997.406178	-4.338389	-1001.551805
conformer_34	-1003.287327	0.298620	0.231429	-1003.323992	-96.3	-1003.025372	-1003.089551	18.7	-997.415976	-4.334883	-1001.553083
conformer_69	-1003.284100	0.298859	0.233855	-1003.323880	-104.4	-1003.025021	-1003.087013	25.4	-997.403666	-4.343220	-1001.549799
conformer_24	-1003.284311	0.298534	0.230256	-1003.323875	-103.9	-1003.025341	-1003.090607	15.9	-997.409629	-4.337439	-1001.553365
conformer_22	-1003.287447	0.298726	0.231903	-1003.323731	-95.3	-1003.025005	-1003.088816	20.6	-997.413815	-4.336626	-1001.551810
conformer_45	-1003.286858	0.298632	0.230850	-1003.323596	-96.5	-1003.024964	-1003.089734	18.2	-997.414020	-4.335833	-1001.552729
conformer_46	-1003.286858	0.298632	0.230850	-1003.323596	-96.5	-1003.024964	-1003.089734	18.2	-997.414020	-4.335833	-1001.552729
conformer_31	-1003.285241	0.298589	0.231292	-1003.323509	-100.5	-1003.024920	-1003.089205	19.6	-997.411342	-4.336972	-1001.552278
conformer_27	-1003.289558	0.298715	0.231484	-1003.323485	-89.1	-1003.024770	-1003.088989	20.2	-997.416889	-4.335954	-1001.552274
conformer_23	-1003.289558	0.298715	0.231484	-1003.323485	-89.1	-1003.024770	-1003.088989	20.2	-997.416889	-4.335954	-1001.552274
conformer_75	-1003.283438	0.298778	0.232063	-1003.323351	-104.8	-1003.024573	-1003.088276	22.1	-997.409026	-4.337061	-1001.550925
conformer_68	-1003.282236	0.298728	0.231785	-1003.323209	-107.6	-1003.024481	-1003.088412	21.7	-997.406832	-4.338543	-1001.551552
conformer_63	-1003.282236	0.298728	0.231785	-1003.323209	-107.6	-1003.024481	-1003.088412	21.7	-997.406832	-4.338543	-1001.551552
conformer_20	-1003.284890	0.298711	0.231169	-1003.323005	-100.1	-1003.024294	-1003.088824	20.6	-997.407389	-4.340598	-1001.551921
conformer_79	-1003.283548	0.298718	0.230836	-1003.322354	-101.9	-1003.023636	-1003.088506	21.5	-997.409502	-4.336534	-1001.550994
conformer_21	-1003.290207	0.298751	0.231085	-1003.321983	-83.4	-1003.023232	-1003.087886	23.1	-997.417963	-4.335870	-1001.551511
conformer_44	-1003.290207	0.298751	0.231085	-1003.321983	-83.4	-1003.023232	-1003.087886	23.1	-997.417963	-4.335870	-1001.551511
conformer_65	-1003.290207	0.298751	0.231085	-1003.321983	-83.4	-1003.023232	-1003.087886	23.1	-997.417962	-4.335870	-1001.551511
conformer_15	-1003.282730	0.298588	0.228227	-1003.320263	-98.5	-1003.021675	-1003.089024	20.1	-997.406356	-4.339428	-1001.552078

[a]: Single-point calculation in aqueous phase with SMD model.

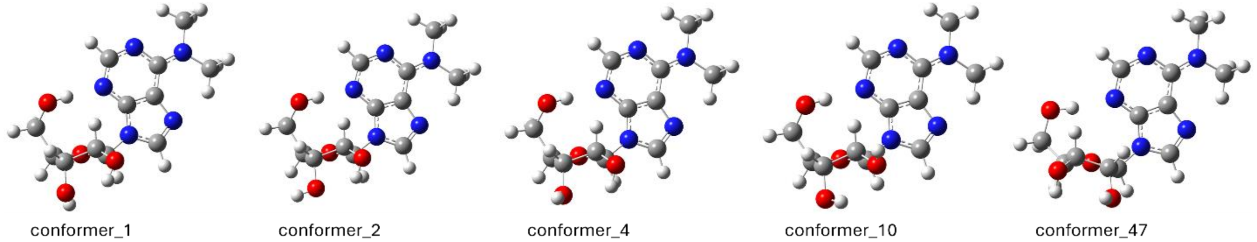

Figure S51. B3LYP-D3/def2-TZVPP optimized geometries of conformers for N^6,N^6-dimethyladenosine (**9rb6dmA**).

Table S49. Conformers of gas-phase optimized N^6,N^6-dimethyladenosine (**9rb6dmA**) at the B3LYP-D3/def2-TZVPP level of theory followed by aqueous phase single-point calculation. The columns display total energy without zero-point correction (E_{Tot}), Gibbs free energy (δG), total energy without zero-point correction ($E_{Tot,W}$), Gibbs free energy ($G_{298,W}$) in water (W), total single-point energy (E_{CBS}) calculated at DLPNO-CCSD(T)/CBS level of theory, and their corresponding free energy G_{CBS}. $G_{298,W}$ and G_{CBS} have been corrected to the standard state of 1 mol/L by addition of +7.908 kJ/mol. ΔG_{Solv} represents the Gibbs free energy of solvation. The data are arranged in the ascending numeric order of $E_{Tot,W}$. $\Delta G_{298,W}$ represents the respective energy difference to the lowest structure. Only conformers within the 24 kJ/mol (6 kcal/mol) energy window above the lowest in CREST are included in initial conformer sampling. Duplicates of the same structure are excluded.

9rb6dmA	B3LYP-D3/def2-TZVPP			SMD(H2O)/B3LYP-D3/def2-TZVPP[a]					DLPNO-CCSD(T)/CBS		
No.	E_{Tot} (Hartree)	δH (Hartree)	δG (Hartree)	$E_{Tot,W}$ (Hartree)	ΔG_{Solv} (kJ/mol)	$H_{298,W}$ (Hartree)	$G_{298,W}$ (Hartree)	$\Delta G_{298,W}$ (kJ/mol)	$E_{CBS,HF}$ (Hartree)	$E_{CBS,C}$ (Hartree)	$G_{CBS,W}$ (Hartree)
**conformer_4**	**-1042.617869**	**0.328098**	**0.259116**	**-1042.648194**	**-79.6**	**-1042.320096**	**-1042.386066**	**0.0**	**-1036.453239**	**-4.552960**	**-1040.774396**
conformer_10	-1042.618933	0.328163	0.259780	-1042.647901	-76.1	-1042.319738	-1042.385109	2.5	-1036.455027	-4.552667	-1040.773870
conformer_1	-1042.616819	0.328027	0.259911	-1042.647871	-81.5	-1042.319844	-1042.384948	2.9	-1036.450646	-4.554066	-1040.772842
conformer_2	-1042.618366	0.327993	0.259683	-1042.647717	-77.1	-1042.319724	-1042.385022	2.7	-1036.454222	-4.552666	-1040.773544
conformer_47	-1042.612633	0.328067	0.258515	-1042.643616	-81.3	-1042.315549	-1042.382089	10.4	-1036.449070	-4.551881	-1040.770406
conformer_88	-1042.606771	0.328016	0.256171	-1042.643014	-95.2	-1042.314998	-1042.383831	5.9	-1036.449399	-4.547165	-1040.773624
conformer_60	-1042.609293	0.327801	0.258777	-1042.642898	-88.2	-1042.315097	-1042.381109	13.0	-1036.443855	-4.553482	-1040.769153
conformer_7	-1042.614851	0.327986	0.258342	-1042.642824	-73.4	-1042.314838	-1042.381470	12.1	-1036.451081	-4.552613	-1040.770314
conformer_104	-1042.604942	0.327760	0.255880	-1042.642739	-99.2	-1042.314979	-1042.383847	5.8	-1036.445768	-4.548535	-1040.773207
conformer_3	-1042.617538	0.328019	0.258321	-1042.642473	-65.5	-1042.314454	-1042.381140	12.9	-1036.454723	-4.552057	-1040.770381
conformer_94	-1042.608148	0.327869	0.257438	-1042.642339	-89.8	-1042.314470	-1042.381889	11.0	-1036.448311	-4.549277	-1040.771330
conformer_14	-1042.609594	0.328001	0.259419	-1042.642073	-85.3	-1042.314072	-1042.379642	16.9	-1036.444930	-4.553451	-1040.768429
conformer_55	-1042.609346	0.328037	0.257738	-1042.641971	-85.7	-1042.313934	-1042.381221	12.7	-1036.450522	-4.548375	-1040.770772
conformer_32	-1042.610062	0.327942	0.259154	-1042.641737	-83.2	-1042.313795	-1042.379571	17.1	-1036.447624	-4.551889	-1040.769022
conformer_98	-1042.603516	0.327797	0.256239	-1042.641496	-99.7	-1042.313699	-1042.382245	10.0	-1036.443740	-4.548912	-1040.771380
conformer_71	-1042.606396	0.327956	0.257773	-1042.641482	-92.1	-1042.313526	-1042.380697	14.1	-1036.444748	-4.550447	-1040.769497
conformer_9	-1042.615438	0.327914	0.258456	-1042.641172	-67.6	-1042.313258	-1042.379704	16.7	-1036.453733	-4.551165	-1040.769164
conformer_17	-1042.614228	0.327897	0.258265	-1042.641162	-70.7	-1042.313265	-1042.379885	16.2	-1036.451638	-4.551509	-1040.768804
conformer_93	-1042.608915	0.327862	0.257385	-1042.640718	-83.5	-1042.312856	-1042.380321	15.1	-1036.450991	-4.547856	-1040.770252
conformer_15	-1042.612539	0.327817	0.258103	-1042.640668	-73.9	-1042.312851	-1042.379553	17.1	-1036.451500	-4.550362	-1040.768875
conformer_103	-1042.601450	0.327626	0.255198	-1042.640595	-102.8	-1042.312969	-1042.382385	9.7	-1036.443593	-4.547078	-1040.771606
conformer_6	-1042.614111	0.327938	0.258061	-1042.640556	-69.4	-1042.312618	-1042.379483	17.3	-1036.453148	-4.550297	-1040.768817
conformer_8	-1042.613342	0.327798	0.257765	-1042.640506	-71.3	-1042.312708	-1042.379729	16.6	-1036.451836	-4.550683	-1040.768905
conformer_11	-1042.615540	0.327950	0.257222	-1042.640339	-65.1	-1042.312389	-1042.380105	15.7	-1036.454161	-4.551036	-1040.769763
conformer_70	-1042.608400	0.328045	0.257317	-1042.640068	-83.1	-1042.312023	-1042.379739	16.6	-1036.450278	-4.547912	-1040.769529
conformer_80	-1042.611913	0.327727	0.257877	-1042.639948	-73.6	-1042.312221	-1042.379059	18.4	-1036.451437	-4.550220	-1040.768803
conformer_22	-1042.608398	0.328046	0.257110	-1042.639819	-82.5	-1042.311773	-1042.379697	16.7	-1036.450141	-4.547973	-1040.769413
conformer_84	-1042.608822	0.327928	0.257042	-1042.639656	-81.0	-1042.311728	-1042.379602	17.0	-1036.449888	-4.549185	-1040.769853
conformer_105	-1042.609209	0.327831	0.257179	-1042.639572	-79.7	-1042.311741	-1042.379381	17.6	-1036.452211	-4.547267	-1040.769650

conformer_28	-1042.613366	0.327889	0.257835	-1042.639422	-68.4	-1042.311533	-1042.378575	19.7	-1036.453029	-4.549955	-1040.768193
conformer_79	-1042.604134	0.327758	0.256159	-1042.639332	-92.4	-1042.311574	-1042.380161	15.5	-1036.445947	-4.547866	-1040.769840
conformer_16	-1042.609603	0.327907	0.256813	-1042.639159	-77.6	-1042.311252	-1042.379334	17.7	-1036.450939	-4.548761	-1040.769430
conformer_38	-1042.609608	0.327925	0.256729	-1042.638925	-77.0	-1042.311000	-1042.379184	18.1	-1036.450733	-4.548876	-1040.769185
conformer_31	-1042.607460	0.328096	0.257145	-1042.638515	-81.5	-1042.310419	-1042.378358	20.2	-1036.448339	-4.548528	-1040.767765
conformer_50	-1042.606415	0.327781	0.257078	-1042.638502	-84.2	-1042.310721	-1042.378412	20.1	-1036.448247	-4.548172	-1040.768417
conformer_92	-1042.604591	0.327943	0.257830	-1042.638473	-89.0	-1042.310530	-1042.377631	22.1	-1036.443447	-4.550654	-1040.767141
conformer_21	-1042.604833	0.327879	0.257445	-1042.638380	-88.1	-1042.310501	-1042.377923	21.4	-1036.444109	-4.550061	-1040.767261
conformer_43	-1042.605808	0.327857	0.258071	-1042.638250	-85.2	-1042.310393	-1042.377167	23.4	-1036.440418	-4.554466	-1040.766243
conformer_30	-1042.604194	0.327793	0.255549	-1042.637553	-87.6	-1042.309760	-1042.378992	18.6	-1036.443456	-4.550032	-1040.768286
conformer_40	-1042.607191	0.327840	0.257004	-1042.637493	-79.6	-1042.309653	-1042.377477	22.6	-1036.449674	-4.547680	-1040.767639
conformer_35	-1042.605105	0.327841	0.256914	-1042.637104	-84.0	-1042.309263	-1042.377178	23.3	-1036.445145	-4.549596	-1040.766814
conformer_58	-1042.606868	0.327877	0.256183	-1042.637054	-79.3	-1042.309177	-1042.377859	21.5	-1036.447939	-4.548525	-1040.767455
conformer_25	-1042.607386	0.327928	0.257318	-1042.637020	-77.8	-1042.309092	-1042.376690	24.6	-1036.447705	-4.549312	-1040.766321
conformer_27	-1042.609446	0.327922	0.256630	-1042.636863	-72.0	-1042.308941	-1042.377221	23.2	-1036.450722	-4.548639	-1040.767135
conformer_24	-1042.604665	0.327923	0.257637	-1042.636278	-83.0	-1042.308355	-1042.375629	27.4	-1036.440940	-4.553444	-1040.765347
conformer_99	-1042.603586	0.327862	0.255855	-1042.635928	-84.9	-1042.308066	-1042.377061	23.6	-1036.443668	-4.549033	-1040.766175
conformer_26	-1042.610075	0.327960	0.256653	-1042.635645	-67.1	-1042.307685	-1042.375980	26.5	-1036.451924	-4.548437	-1040.766266
conformer_39	-1042.610644	0.328017	0.257570	-1042.634622	-63.0	-1042.306605	-1042.374040	31.6	-1036.450651	-4.550008	-1040.764056

[a]: Single-point calculation in aqueous phase with SMD model.

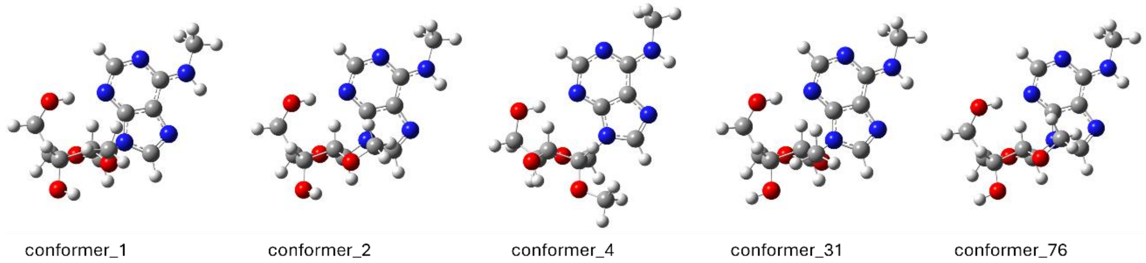

Figure S52. B3LYP-D3/def2-TZVPP optimized geometries of conformers for *N*[6]-2'-*O*-dimethyladenosine (**9rbm6mA**).

Table S50. Conformers of gas-phase optimized *N*[6]-2'-*O*-dimethyladenosine (**9rbm6mA**) at the B3LYP-D3/def2-TZVPP level of theory followed by aqueous phase single-point calculation. The columns display total energy without zero-point correction (E_{Tot}), Gibbs free energy (δG), total energy without zero-point correction ($E_{Tot,W}$), Gibbs free energy ($G_{298,W}$) in water (W), total single-point energy (E_{CBS}) calculated at DLPNO-CCSD(T)/CBS level of theory, and their corresponding free energy G_{CBS}. $G_{298,W}$ and G_{CBS} have been corrected to the standard state of 1 mol/L by addition of +7.908 kJ/mol. ΔG_{Solv} represents the Gibbs free energy of solvation. The data are arranged in the ascending numeric order of $E_{Tot,W}$. $\Delta G_{298,W}$ represents the respective energy difference to the lowest structure. Only conformers within the 24 kJ/mol (6 kcal/mol) energy window above the lowest in CREST are included in initial conformer sampling. Duplicates of the same structure are excluded.

9rbm6dmA	B3LYP-D3/def2-TZVPP			SMD(H2O)/B3LYP-D3/def2-TZVPP[a]					DLPNO-CCSD(T)/CBS		
No.	E_{Tot} (Hartree)	δH (Hartree)	δG (Hartree)	$E_{Tot,W}$ (Hartree)	ΔG_{Solv} (kJ/mol)	$H_{298,W}$ (Hartree)	$G_{298,W}$ (Hartree)	$\Delta G_{298,W}$ (kJ/mol)	$E_{CBS,HF}$ (Hartree)	$E_{CBS,C}$ (Hartree)	$G_{CBS,W}$ (Hartree)
**conformer_1**	**-1042.617138**	**0.328299**	**0.259892**	**-1042.647847**	**-80.6**	**-1042.319548**	**-1042.384943**	**0.0**	**-1036.457903**	**-4.545632**	**-1040.771340**
conformer_2	-1042.616405	0.328286	0.259523	-1042.646501	-79.0	-1042.318215	-1042.383966	2.6	-1036.457141	-4.545646	-1040.770348
conformer_31	-1042.612190	0.328131	0.259650	-1042.645632	-87.8	-1042.317501	-1042.382970	5.2	-1036.453990	-4.544504	-1040.769273
conformer_76	-1042.609321	0.328013	0.259099	-1042.644563	-92.5	-1042.316550	-1042.382452	6.5	-1036.449987	-4.545600	-1040.768717
conformer_4	-1042.610141	0.328061	0.259008	-1042.643045	-86.4	-1042.314984	-1042.381025	10.3	-1036.451643	-4.544698	-1040.767225
conformer_50	-1042.607941	0.328122	0.259115	-1042.642289	-90.2	-1042.314167	-1042.380162	12.6	-1036.450359	-4.544726	-1040.767306
conformer_8	-1042.608544	0.328326	0.258036	-1042.642166	-88.3	-1042.313840	-1042.381118	10.0	-1036.454111	-4.541487	-1040.768172
conformer_52	-1042.608371	0.328623	0.261109	-1042.641693	-87.5	-1042.313070	-1042.377572	19.4	-1036.447028	-4.546750	-1040.762979
conformer_59	-1042.607404	0.328196	0.257278	-1042.640383	-86.6	-1042.312187	-1042.380093	12.7	-1036.453538	-4.541051	-1040.767278
conformer_96	-1042.605111	0.327909	0.258579	-1042.640264	-92.3	-1042.312355	-1042.378673	16.5	-1036.446809	-4.544725	-1040.765095
conformer_15	-1042.607116	0.328087	0.257381	-1042.640093	-86.6	-1042.312006	-1042.379700	13.8	-1036.452805	-4.542091	-1040.767479
conformer_18	-1042.606355	0.328109	0.257402	-1042.639541	-87.1	-1042.311432	-1042.379127	15.3	-1036.453484	-4.540396	-1040.766651
conformer_13	-1042.606899	0.328229	0.257051	-1042.639489	-85.6	-1042.311260	-1042.379426	14.5	-1036.454189	-4.540118	-1040.766834
conformer_70	-1042.604403	0.327982	0.257269	-1042.639361	-91.8	-1042.311379	-1042.379080	15.4	-1036.449596	-4.542101	-1040.766375
conformer_64	-1042.607779	0.328185	0.257213	-1042.639271	-82.7	-1042.311086	-1042.379046	15.5	-1036.454731	-4.540602	-1040.766599
conformer_77	-1042.604417	0.328294	0.259765	-1042.63917	-91.2	-1042.310876	-1042.376393	22.5	-1036.441591	-4.547126	-1040.760692
conformer_78	-1042.602563	0.327854	0.256827	-1042.638833	-95.2	-1042.310979	-1042.378994	15.6	-1036.449685	-4.540435	-1040.766550
conformer_27	-1042.605047	0.328021	0.257077	-1042.638811	-88.6	-1042.310790	-1042.378722	16.3	-1036.451379	-4.541316	-1040.766370
conformer_16	-1042.605495	0.328152	0.257267	-1042.638733	-87.3	-1042.310581	-1042.378454	17.0	-1036.452393	-4.540336	-1040.765688
conformer_88	-1042.605053	0.327973	0.257945	-1042.638697	-88.3	-1042.310724	-1042.377740	18.9	-1036.449255	-4.542643	-1040.764586
conformer_30	-1042.606940	0.328203	0.257812	-1042.638661	-83.3	-1042.310458	-1042.377837	18.7	-1036.452383	-4.542233	-1040.765512
conformer_67	-1042.603563	0.327966	0.255931	-1042.638544	-91.8	-1042.310578	-1042.379601	14.0	-1036.451133	-4.540063	-1040.767234
conformer_44	-1042.607684	0.328006	0.258466	-1042.638406	-80.7	-1042.310400	-1042.376928	21.0	-1036.449509	-4.544493	-1040.763246
conformer_65	-1042.601668	0.327858	0.256942	-1042.638352	-96.3	-1042.310494	-1042.378398	17.2	-1036.447450	-4.541844	-1040.766023
conformer_66	-1042.602614	0.327911	0.256731	-1042.638114	-93.2	-1042.310203	-1042.378371	17.3	-1036.449222	-4.540698	-1040.765678
conformer_92	-1042.601305	0.327803	0.256590	-1042.637879	-96.0	-1042.310076	-1042.378277	17.5	-1036.447378	-4.541127	-1040.765477
conformer_9	-1042.605838	0.328160	0.257918	-1042.637846	-84.0	-1042.309686	-1042.376916	21.1	-1036.451948	-4.541121	-1040.764147
conformer_33	-1042.604798	0.328111	0.257108	-1042.637663	-86.3	-1042.309552	-1042.377543	19.4	-1036.450956	-4.541369	-1040.765071
conformer_62	-1042.603442	0.327991	0.256582	-1042.637662	-89.8	-1042.309671	-1042.378068	18.1	-1036.449213	-4.541654	-1040.765493

conformer_63	-1042.604468	0.327959	0.256717	-1042.637621	-87.0	-1042.309662	-1042.377892	18.5	-1036.451403	-4.541021	-1040.765848
conformer_5	-1042.606449	0.328273	0.257271	-1042.637506	-81.5	-1042.309233	-1042.377223	20.3	-1036.452902	-4.540698	-1040.764373
conformer_29	-1042.604753	0.328295	0.257781	-1042.637052	-84.8	-1042.308757	-1042.376259	22.8	-1036.450564	-4.540981	-1040.763051
conformer_85	-1042.601565	0.327924	0.256879	-1042.636694	-92.2	-1042.308770	-1042.376803	21.4	-1036.447283	-4.541870	-1040.764391
conformer_84	-1042.604236	0.328043	0.256771	-1042.636453	-84.6	-1042.308410	-1042.376670	21.7	-1036.450943	-4.541123	-1040.764501

[a]: Single-point calculation in aqueous phase with SMD model.

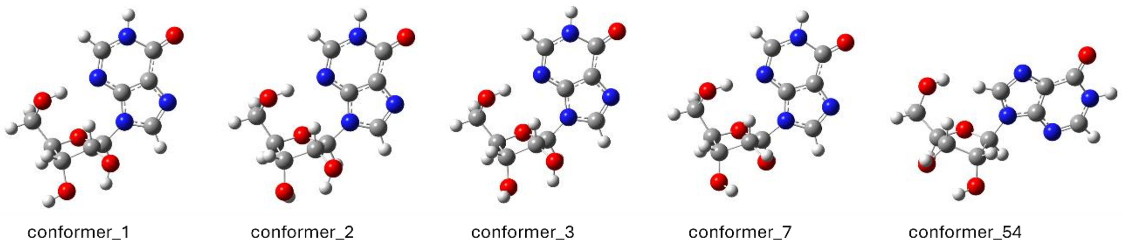

Figure S53. B3LYP-D3/def2-TZVPP optimized geometries of conformers for inosine (**9rbI**).

Table S51. Conformers of gas-phase optimized inosine (**9rbI**) at the B3LYP-D3/def2-TZVPP level of theory followed by aqueous phase single-point calculation. The columns display total energy without zero-point correction (E_{Tot}), Gibbs free energy (δG), total energy without zero-point correction ($E_{Tot,W}$), Gibbs free energy ($G_{298,W}$) in water (W), total single-point energy (E_{CBS}) calculated at DLPNO-CCSD(T)/CBS level of theory, and their corresponding free energy G_{CBS}. $G_{298,W}$ and G_{CBS} have been corrected to the standard state of 1 mol/L by addition of +7.908 kJ/mol. ΔG_{Solv} represents the Gibbs free energy of solvation. The data are arranged in the ascending numeric order of $E_{Tot,W}$. $\Delta G_{298,W}$ represents the respective energy difference to the lowest structure. Only conformers within the 24 kJ/mol (6 kcal/mol) energy window above the lowest in CREST are included in initial conformer sampling. Duplicates of the same structure are excluded.

	B3LYP-D3/def2-TZVPP			SMD(H2O)/B3LYP-D3/def2-TZVPP[a]					DLPNO-CCSD(T)/CBS		
9rbI No.	E_{Tot} (Hartree)	δH (Hartree)	δG (Hartree)	$E_{Tot,W}$ (Hartree)	ΔG_{Solv} (kJ/mol)	$H_{298,W}$ (Hartree)	$G_{298,W}$ (Hartree)	$\Delta G_{298,W}$ (kJ/mol)	$E_{CBS,HF}$ (Hartree)	$E_{CBS,C}$ (Hartree)	$G_{CBS,W}$ (Hartree)
**conformer_3**	**-983.847915**	**0.256891**	**0.196751**	**-983.894365**	**-122.0**	**-983.637474**	**-983.694602**	**0.0**	**-978.212967**	**-4.160448**	**-982.220102**
conformer_2	-983.846536	0.256772	0.197146	-983.893930	-124.4	-983.637158	-983.693772	2.2	-978.210062	-4.161528	-982.218825
conformer_7	-983.848772	0.256886	0.197343	-983.893808	-118.2	-983.636922	-983.693453	3.0	-978.214328	-4.160374	-982.219382
conformer_1	-983.848327	0.256737	0.196967	-983.893740	-119.2	-983.637003	-983.693761	2.2	-978.213787	-4.160229	-982.219450
conformer_54	-983.840995	0.256633	0.195172	-983.889866	-128.3	-983.633233	-983.691682	7.7	-978.210383	-4.156998	-982.218068
conformer_79	-983.837131	0.256552	0.193923	-983.889849	-138.4	-983.633297	-983.692914	4.4	-978.206105	-4.157072	-982.218960
conformer_51	-983.843278	0.256775	0.196102	-983.889806	-122.2	-983.633031	-983.690692	10.3	-978.209370	-4.159420	-982.216205
conformer_45	-983.840784	0.256721	0.195285	-983.889573	-128.1	-983.632852	-983.691276	8.7	-978.210734	-4.156484	-982.217710
conformer_80	-983.838431	0.256439	0.193609	-983.889534	-134.2	-983.633095	-983.692913	4.4	-978.209806	-4.155318	-982.219606
conformer_63	-983.835696	0.256393	0.193655	-983.889249	-140.6	-983.632856	-983.692582	5.3	-978.204952	-4.156874	-982.218712
conformer_82	-983.839958	0.256546	0.196523	-983.889095	-129.0	-983.632549	-983.689560	13.2	-978.204182	-4.160930	-982.214714
conformer_5	-983.845055	0.256660	0.196390	-983.889076	-115.6	-983.632416	-983.689674	12.9	-978.210828	-4.160214	-982.215661
conformer_32	-983.842170	0.256505	0.195646	-983.888989	-122.9	-983.632484	-983.690331	11.2	-978.208427	-4.159516	-982.216104
conformer_56	-983.838232	0.256702	0.195531	-983.888870	-132.9	-983.632168	-983.690327	11.2	-978.205634	-4.158328	-982.216057
conformer_128	-983.839266	0.256402	0.193743	-983.888675	-129.7	-983.632273	-983.691920	7.0	-978.211013	-4.155225	-982.218892
conformer_109	-983.837332	0.256422	0.193385	-983.888617	-134.6	-983.632195	-983.692220	6.3	-978.208643	-4.155550	-982.219080
conformer_10	-983.839778	0.256656	0.196448	-983.888594	-128.2	-983.631938	-983.689134	14.4	-978.204636	-4.160954	-982.214946
conformer_4	-983.846612	0.256660	0.195606	-983.888544	-110.1	-983.631884	-983.689926	12.3	-978.212964	-4.159812	-982.216091
conformer_88	-983.836691	0.256337	0.193446	-983.888493	-136.0	-983.632156	-983.692035	6.7	-978.209151	-4.154368	-982.218864
conformer_105	-983.837329	0.256361	0.193009	-983.888337	-133.9	-983.631976	-983.692316	6.0	-978.208668	-4.155723	-982.219379
conformer_117	-983.839056	0.256573	0.194378	-983.888322	-129.3	-983.631749	-983.690932	9.6	-978.210078	-4.155294	-982.217248
conformer_19	-983.840516	0.256559	0.196026	-983.888273	-125.4	-983.631714	-983.689235	14.1	-978.207664	-4.159354	-982.215737
conformer_90	-983.839993	0.256487	0.194732	-983.888090	-126.3	-983.631603	-983.690346	11.2	-978.210485	-4.156181	-982.217019
conformer_65	-983.833847	0.256365	0.193145	-983.888064	-142.3	-983.631699	-983.691907	7.1	-978.204598	-4.155345	-982.218003
conformer_151	-983.835972	0.256235	0.193313	-983.887968	-136.5	-983.631733	-983.691643	7.8	-978.206699	-4.155473	-982.217843
conformer_78	-983.835688	0.256482	0.194501	-983.887936	-137.2	-983.631454	-983.690423	11.0	-978.204081	-4.157209	-982.216025
conformer_125	-983.832726	0.256210	0.192429	-983.887882	-144.8	-983.631672	-983.692441	5.7	-978.203783	-4.155007	-982.218506
conformer_112	-983.838327	0.256382	0.194409	-983.887795	-129.9	-983.631413	-983.690374	11.1	-978.209910	-4.154970	-982.216927
conformer_57	-983.837722	0.256599	0.195222	-983.887782	-131.4	-983.631183	-983.689548	13.3	-978.206981	-4.156710	-982.215518
conformer_24	-983.839561	0.256671	0.194450	-983.887432	-125.7	-983.630761	-983.689970	12.2	-978.210455	-4.155835	-982.216698

conformer_135	-983.839425	0.256436	0.194514	-983.887380	-125.9	-983.630944	-983.689854	12.5	-978.210226	-4.156149	-982.216805
conformer_101	-983.835792	0.256329	0.193067	-983.887316	-135.3	-983.630987	-983.691237	8.8	-978.209126	-4.153548	-982.218118
conformer_11	-983.844088	0.256624	0.195579	-983.887295	-113.4	-983.630671	-983.688704	15.5	-978.211571	-4.158909	-982.215095
conformer_49	-983.839337	0.256554	0.193789	-983.887226	-125.7	-983.630672	-983.690425	11.0	-978.211575	-4.154733	-982.217396
conformer_139	-983.836340	0.256231	0.192513	-983.887226	-133.6	-983.630995	-983.691701	7.6	-978.208415	-4.155102	-982.218878
conformer_87	-983.834549	0.256620	0.193596	-983.887124	-138.0	-983.630504	-983.690516	10.7	-978.202061	-4.159382	-982.217410
conformer_137	-983.839770	0.256360	0.194600	-983.887123	-124.3	-983.630763	-983.689511	13.4	-978.208152	-4.158628	-982.216521
conformer_97	-983.840525	0.256450	0.194463	-983.887120	-122.3	-983.630670	-983.689645	13.0	-978.211905	-4.155665	-982.216690
conformer_40	-983.843215	0.256549	0.195444	-983.886969	-114.9	-983.630420	-983.688513	16.0	-978.209771	-4.159428	-982.214498
conformer_75	-983.836164	0.256518	0.194614	-983.886916	-133.2	-983.630398	-983.689290	13.9	-978.204735	-4.157894	-982.215755
conformer_118	-983.833977	0.256329	0.193209	-983.886880	-138.9	-983.630551	-983.690659	10.4	-978.205251	-4.155049	-982.216982
conformer_124	-983.836938	0.256441	0.194362	-983.886818	-131.0	-983.630377	-983.689444	13.5	-978.206124	-4.157165	-982.215795
conformer_39	-983.841364	0.256545	0.195160	-983.886705	-119.0	-983.630160	-983.688533	15.9	-978.209408	-4.158328	-982.214906
conformer_8	-983.845312	0.256628	0.195567	-983.886513	-108.2	-983.629885	-983.687934	17.5	-978.212933	-4.159000	-982.214555
conformer_6	-983.843132	0.256604	0.195391	-983.886491	-113.8	-983.629887	-983.688088	17.1	-978.211381	-4.158100	-982.214437
conformer_41	-983.836392	0.256455	0.194096	-983.886442	-131.4	-983.629987	-983.689334	13.8	-978.205777	-4.156955	-982.215674
conformer_26	-983.842021	0.256457	0.195048	-983.886441	-116.6	-983.629984	-983.688381	16.3	-978.209732	-4.158500	-982.214591
conformer_111	-983.836377	0.256360	0.193190	-983.886423	-131.4	-983.630063	-983.690221	11.5	-978.210373	-4.153062	-982.217279
conformer_18	-983.840116	0.256555	0.194547	-983.886412	-121.5	-983.629857	-983.688853	15.1	-978.210321	-4.156756	-982.215813
conformer_59	-983.838684	0.256403	0.193417	-983.886280	-125.0	-983.629877	-983.689851	12.5	-978.209954	-4.155672	-982.216793
conformer_74	-983.838866	0.256574	0.194051	-983.886150	-124.1	-983.629576	-983.689087	14.5	-978.210098	-4.155736	-982.216055
conformer_129	-983.836151	0.256377	0.193124	-983.886140	-131.2	-983.629763	-983.690004	12.1	-978.208645	-4.154606	-982.217105
conformer_142	-983.841025	0.256470	0.195104	-983.886105	-118.4	-983.629635	-983.687989	17.4	-978.209621	-4.158167	-982.214753
conformer_31	-983.838039	0.256467	0.194554	-983.886000	-125.9	-983.629533	-983.688434	16.2	-978.209045	-4.155923	-982.215362
conformer_44	-983.838537	0.256786	0.195063	-983.885900	-124.4	-983.629114	-983.687825	17.8	-978.208593	-4.156396	-982.214277
conformer_94	-983.836187	0.256294	0.193926	-983.885857	-130.4	-983.629563	-983.688919	14.9	-978.206420	-4.156685	-982.215838
conformer_12	-983.836231	0.256545	0.195036	-983.885829	-130.2	-983.629284	-983.687781	17.9	-978.204727	-4.157676	-982.213953
conformer_15	-983.837772	0.256570	0.195469	-983.885609	-125.6	-983.629039	-983.687128	19.6	-978.201567	-4.162332	-982.213254
conformer_34	-983.835131	0.256447	0.194269	-983.885584	-132.5	-983.629137	-983.688303	16.5	-978.203754	-4.157581	-982.214507
conformer_89	-983.837765	0.256399	0.193765	-983.885415	-125.1	-983.629016	-983.688638	15.7	-978.209412	-4.155504	-982.215790
conformer_52	-983.842555	0.256558	0.195196	-983.885395	-112.5	-983.628837	-983.687187	19.5	-978.211433	-4.157785	-982.213850
conformer_64	-983.833136	0.256497	0.194760	-983.885329	-137.0	-983.628832	-983.687557	18.5	-978.200916	-4.158398	-982.213736
conformer_29	-983.835218	0.256607	0.194806	-983.885203	-131.2	-983.628596	-983.687385	18.9	-978.203772	-4.157643	-982.213583
conformer_106	-983.831490	0.256553	0.194480	-983.885138	-140.9	-983.628585	-983.687646	18.3	-978.198715	-4.159350	-982.214221
conformer_66	-983.839544	0.256575	0.194417	-983.885011	-119.4	-983.628436	-983.687582	18.4	-978.211479	-4.155522	-982.215038
conformer_43	-983.838396	0.256472	0.194458	-983.884955	-122.2	-983.628483	-983.687485	18.7	-978.210135	-4.155394	-982.214619
conformer_38	-983.838916	0.256697	0.193866	-983.884753	-120.3	-983.628056	-983.687875	17.7	-978.210477	-4.155098	-982.214535
conformer_93	-983.833494	0.256488	0.194201	-983.884715	-134.5	-983.628227	-983.687502	18.6	-978.201337	-4.158932	-982.214277
conformer_42	-983.836011	0.256477	0.194519	-983.884572	-127.5	-983.628095	-983.687041	19.9	-978.205368	-4.157169	-982.213566
conformer_73	-983.835013	0.256820	0.197068	-983.884518	-130.0	-983.627698	-983.684438	26.7	-978.198200	-4.163432	-982.211057
conformer_47	-983.838088	0.256516	0.194363	-983.884460	-121.8	-983.627944	-983.687085	19.7	-978.208350	-4.156376	-982.213723
conformer_27	-983.835621	0.256514	0.194868	-983.884428	-128.1	-983.627914	-983.686548	21.1	-978.204103	-4.157632	-982.212662
conformer_16	-983.840794	0.256559	0.194401	-983.884408	-114.5	-983.627849	-983.686995	20.0	-978.211292	-4.156522	-982.214015
conformer_146	-983.834108	0.256483	0.194287	-983.884318	-131.8	-983.627835	-983.687019	19.9	-978.202340	-4.158818	-982.214068
conformer_83	-983.834244	0.256392	0.194943	-983.884057	-130.8	-983.627665	-983.686102	22.3	-978.198329	-4.162125	-982.212313
conformer_153	-983.834431	0.256482	0.194653	-983.883902	-129.9	-983.627420	-983.686237	22.0	-978.200066	-4.160519	-982.212392
conformer_25	-983.835558	0.256623	0.194752	-983.883579	-126.1	-983.626956	-983.685815	23.1	-978.201295	-4.160963	-982.212515
conformer_23	-983.841198	0.256556	0.194349	-983.883340	-110.6	-983.626784	-983.685979	22.6	-978.212520	-4.156171	-982.213472
conformer_86	-983.834995	0.256618	0.193982	-983.883339	-126.9	-983.626721	-983.686345	21.7	-978.204253	-4.156815	-982.212418
conformer_144	-983.833595	0.256265	0.195252	-983.882232	-127.7	-983.625967	-983.683968	27.9	-978.199652	-4.160898	-982.210922
conformer_30	-983.832750	0.256621	0.195103	-983.881704	-128.5	-983.625083	-983.683589	28.9	-978.197952	-4.161645	-982.210436
conformer_21	-983.833437	0.256659	0.193893	-983.880951	-124.7	-983.624292	-983.684046	27.7	-978.199836	-4.160213	-982.210658
conformer_141	-983.831829	0.256451	0.194647	-983.880851	-128.7	-983.624400	-983.683192	30.0	-978.197875	-4.161300	-982.210538
conformer_9	-983.839528	0.256712	0.195068	-983.880693	-108.1	-983.623981	-983.682613	31.5	-978.209503	-4.157206	-982.209794

[a]: Single-point calculation in aqueous phase with SMD model.

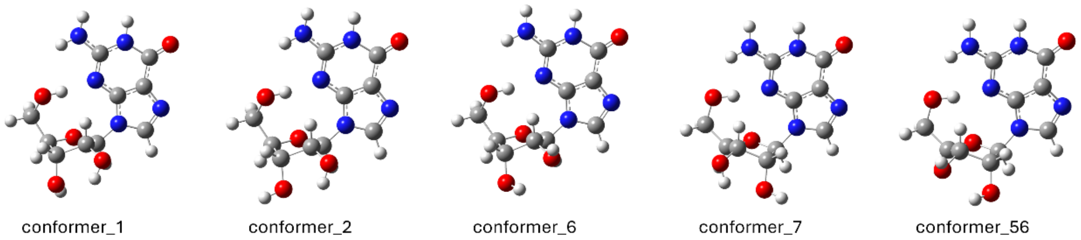

Figure S54. B3LYP-D3/def2-TZVPP optimized geometries of conformers for guanosine (**9rbG**).

Table S52. Conformers of gas-phase optimized guanosine (**9rbG**) at the B3LYP-D3/def2-TZVPP level of theory followed by aqueous phase single-point calculation. The columns display total energy without zero-point correction (E_{Tot}), Gibbs free energy (δG), total energy without zero-point correction ($E_{Tot,W}$), Gibbs free energy ($G_{298,W}$) in water (W), total single-point energy (E_{CBS}) calculated at DLPNO-CCSD(T)/CBS level of theory, and their corresponding free energy G_{CBS}. $G_{298,W}$ and G_{CBS} have been corrected to the standard state of 1 mol/L by addition of +7.908 kJ/mol. ΔG_{Solv} represents the Gibbs free energy of solvation. The data are arranged in the ascending numeric order of $E_{Tot,W}$. $\Delta G_{298,W}$ represents the respective energy difference to the lowest structure. Only conformers within the 24 kJ/mol (6 kcal/mol) energy window above the lowest in CREST are included in initial conformer sampling. Duplicates of the same structure are excluded.

	B3LYP-D3/def2-TZVPP			SMD(H_2O)/B3LYP-D3/def2-TZVPP[a]					DLPNO-CCSD(T)/CBS		
9rbG No.	E_{Tot} (Hartree)	δH (Hartree)	δG (Hartree)	$E_{Tot,W}$ (Hartree)	ΔG_{Solv} (kJ/mol)	$H_{298,W}$ (Hartree)	$G_{298,W}$ (Hartree)	$\Delta G_{298,W}$ (kJ/mol)	$E_{CBS,HF}$ (Hartree)	$E_{CBS,C}$ (Hartree)	$G_{CBS,W}$ (Hartree)
**conformer_1**	**-1039.245115**	**0.274774**	**0.212386**	**-1039.298128**	**-139.2**	**-1039.023354**	**-1039.082730**	**0.0**	**-1033.286208**	**-4.403786**	**-1037.527609**
conformer_6	-1039.247527	0.274914	0.212436	-1039.298004	-132.5	-1039.023090	-1039.082556	0.5	-1033.290704	-4.402575	-1037.528308
conformer_2	-1039.246687	0.274785	0.212320	-1039.297877	-134.4	-1039.023092	-1039.082545	0.5	-1033.289699	-4.402562	-1037.528119
conformer_56	-1039.245030	0.274788	0.212249	-1039.293354	-126.9	-1039.018566	-1039.078093	12.2	-1033.287754	-4.403115	-1037.523933
conformer_7	-1039.242935	0.274615	0.211732	-1039.293179	-131.9	-1039.018564	-1039.078435	11.3	-1033.286025	-4.402395	-1037.523920
conformer_8	-1039.240656	0.274612	0.212168	-1039.293045	-137.5	-1039.018433	-1039.077865	12.8	-1033.281775	-4.403778	-1037.522762
conformer_88	-1039.237474	0.274661	0.210067	-1039.292959	-145.7	-1039.018298	-1039.079880	7.5	-1033.286565	-4.397491	-1037.526462
conformer_78	-1039.236948	0.274736	0.209998	-1039.292697	-146.4	-1039.017961	-1039.079687	8.0	-1033.286786	-4.396932	-1037.526456
conformer_86	-1039.236725	0.274704	0.209942	-1039.292596	-146.7	-1039.017892	-1039.079642	8.1	-1033.286557	-4.396898	-1037.526372
conformer_11	-1039.243030	0.274809	0.210974	-1039.292536	-130.0	-1039.017727	-1039.078550	11.0	-1033.288640	-4.400671	-1037.524830
conformer_9	-1039.239383	0.274907	0.212632	-1039.292254	-138.8	-1039.017347	-1039.076610	16.1	-1033.281133	-4.403787	-1037.522148
conformer_13	-1039.239949	0.274807	0.212320	-1039.292184	-137.1	-1039.017377	-1039.076852	15.4	-1033.283885	-4.402261	-1037.523049
conformer_90	-1039.234622	0.274709	0.210461	-1039.292134	-151.0	-1039.017425	-1039.078661	10.7	-1033.281750	-4.398803	-1037.524592
conformer_38	-1039.239999	0.274724	0.212037	-1039.291618	-135.5	-1039.016894	-1039.076569	16.2	-1033.284372	-4.402151	-1037.523093
conformer_18	-1039.240276	0.274513	0.209525	-1039.291198	-133.7	-1039.016685	-1039.078661	10.7	-1033.287463	-4.399545	-1037.525392
conformer_43	-1039.235865	0.274717	0.209364	-1039.290809	-144.3	-1039.016092	-1039.078433	11.3	-1033.286348	-4.396554	-1037.525471
conformer_47	-1039.235788	0.274703	0.209325	-1039.290749	-144.3	-1039.016046	-1039.078412	11.3	-1033.286230	-4.396504	-1037.525358
conformer_68	-1039.238983	0.274519	0.210151	-1039.290601	-135.5	-1039.016082	-1039.077438	13.9	-1033.285294	-4.400025	-1037.523774
conformer_99	-1039.235282	0.274559	0.208978	-1039.290581	-145.2	-1039.016022	-1039.078591	10.9	-1033.287236	-4.395411	-1037.525956
conformer_100	-1039.235118	0.274531	0.209018	-1039.290493	-145.4	-1039.015962	-1039.078463	11.2	-1033.287048	-4.395413	-1037.525806
conformer_15	-1039.239283	0.274659	0.210115	-1039.290486	-134.4	-1039.015827	-1039.077359	14.1	-1033.287159	-4.398703	-1037.523938
conformer_57	-1039.237290	0.274468	0.209334	-1039.290236	-139.0	-1039.015768	-1039.077890	12.7	-1033.285144	-4.398830	-1037.524574
conformer_58	-1039.237287	0.274455	0.209397	-1039.290070	-138.6	-1039.015615	-1039.077661	13.3	-1033.284992	-4.398991	-1037.524358
conformer_23	-1039.241042	0.274600	0.210086	-1039.289913	-128.3	-1039.015313	-1039.076815	15.5	-1033.288483	-4.399528	-1037.523783
conformer_72	-1039.235332	0.274464	0.208613	-1039.289508	-142.2	-1039.015044	-1039.077883	12.7	-1033.286111	-4.396410	-1037.525072
conformer_48	-1039.233115	0.274531	0.209289	-1039.289500	-148.0	-1039.014969	-1039.077199	14.5	-1033.281898	-4.397746	-1037.523729
conformer_62	-1039.235240	0.274464	0.208814	-1039.289487	-142.4	-1039.015023	-1039.077661	13.3	-1033.286008	-4.396359	-1037.524788
conformer_49	-1039.235456	0.274526	0.209014	-1039.289461	-141.8	-1039.014935	-1039.077435	13.9	-1033.285079	-4.397604	-1037.524661
conformer_65	-1039.238697	0.274609	0.210056	-1039.289385	-133.1	-1039.014776	-1039.076317	16.8	-1033.287192	-4.398416	-1037.523228
conformer_50	-1039.233864	0.274553	0.209695	-1039.289321	-145.6	-1039.014768	-1039.076614	16.1	-1033.284176	-4.396838	-1037.523764

conformer_81	-1039.234660	0.274829	0.210007	-1039.289185	-143.2	-1039.014356	-1039.076166	17.2	-1033.284138	-4.397114	-1037.522758
conformer_71	-1039.234794	0.274836	0.209888	-1039.289156	-142.7	-1039.014320	-1039.076256	17.0	-1033.284302	-4.397194	-1037.522957
conformer_64	-1039.230886	0.274446	0.209137	-1039.289113	-152.9	-1039.014667	-1039.076964	15.1	-1033.278844	-4.398502	-1037.523424
conformer_28	-1039.234747	0.274651	0.210768	-1039.289092	-142.7	-1039.014441	-1039.075312	19.5	-1033.277951	-4.402880	-1037.521395
conformer_21	-1039.235045	0.274717	0.210948	-1039.289055	-141.8	-1039.014338	-1039.075095	20.0	-1033.278265	-4.402952	-1037.521267
conformer_45	-1039.235819	0.274558	0.209424	-1039.289051	-139.8	-1039.014493	-1039.076615	16.1	-1033.284844	-4.398182	-1037.523821
conformer_66	-1039.230772	0.274429	0.208961	-1039.288993	-152.9	-1039.014564	-1039.077020	15.0	-1033.278785	-4.398458	-1037.523491
conformer_16	-1039.232436	0.274603	0.209722	-1039.288817	-148.0	-1039.014214	-1039.076083	17.5	-1033.279872	-4.398904	-1037.522423
conformer_80	-1039.234280	0.274517	0.209447	-1039.288689	-142.9	-1039.014172	-1039.076230	17.1	-1033.285175	-4.396502	-1037.523627
conformer_97	-1039.234321	0.274370	0.207880	-1039.288251	-141.6	-1039.013881	-1039.077359	14.1	-1033.285388	-4.396497	-1037.524924
conformer_46	-1039.231857	0.274673	0.210073	-1039.288023	-147.5	-1039.013350	-1039.074938	20.5	-1033.280294	-4.397980	-1037.521355
conformer_84	-1039.233807	0.274542	0.209370	-1039.287949	-142.2	-1039.013407	-1039.075567	18.8	-1033.285167	-4.396135	-1037.523062
conformer_44	-1039.236770	0.274697	0.209827	-1039.287798	-134.0	-1039.013101	-1039.074959	20.4	-1033.286642	-4.397358	-1037.522188
conformer_73	-1039.234673	0.274613	0.209476	-1039.287796	-139.5	-1039.013183	-1039.075308	19.5	-1033.284377	-4.397222	-1037.522234
conformer_52	-1039.236888	0.274718	0.209983	-1039.287777	-133.6	-1039.013059	-1039.074782	20.9	-1033.286707	-4.397452	-1037.522053
conformer_61	-1039.234528	0.274584	0.209474	-1039.287758	-139.8	-1039.013174	-1039.075272	19.6	-1033.284219	-4.397135	-1037.522097
conformer_35	-1039.232195	0.274547	0.209614	-1039.287677	-145.7	-1039.013130	-1039.075051	20.2	-1033.279976	-4.398526	-1037.521358
conformer_31	-1039.232201	0.274560	0.209782	-1039.287629	-145.5	-1039.013069	-1039.074835	20.7	-1033.279942	-4.398566	-1037.521142
conformer_59	-1039.231706	0.274551	0.209227	-1039.287291	-145.9	-1039.012740	-1039.075052	20.2	-1033.280302	-4.398298	-1037.521946
conformer_51	-1039.232595	0.274658	0.210043	-1039.286908	-142.6	-1039.012250	-1039.073853	23.3	-1033.277588	-4.401680	-1037.520527
conformer_26	-1039.232634	0.274649	0.209967	-1039.286774	-142.1	-1039.012125	-1039.073795	23.5	-1033.277784	-4.401681	-1037.520625
conformer_63	-1039.237212	0.274733	0.210033	-1039.286655	-129.8	-1039.011922	-1039.073610	23.9	-1033.287939	-4.397007	-1037.521345
conformer_83	-1039.236898	0.274678	0.209815	-1039.286652	-130.6	-1039.011974	-1039.073825	23.4	-1033.287705	-4.396910	-1037.521542
conformer_33	-1039.232586	0.274830	0.212172	-1039.285888	-139.9	-1039.011058	-1039.070704	31.6	-1033.273603	-4.404943	-1037.516664
conformer_67	-1039.232041	0.274559	0.211381	-1039.285437	-140.2	-1039.010878	-1039.071044	30.7	-1033.275553	-4.402660	-1037.517216
conformer_30	-1039.229876	0.274666	0.209920	-1039.284892	-144.4	-1039.010226	-1039.071960	28.3	-1033.274338	-4.402484	-1037.518907
conformer_41	-1039.229515	0.274587	0.209628	-1039.284862	-145.3	-1039.010275	-1039.072222	27.6	-1033.274182	-4.402327	-1037.519216
conformer_19	-1039.235585	0.274714	0.210038	-1039.284443	-128.3	-1039.009729	-1039.071393	29.8	-1033.284932	-4.398074	-1037.518814
conformer_85	-1039.235565	0.274700	0.210029	-1039.284433	-128.3	-1039.009733	-1039.071392	29.8	-1033.284728	-4.398239	-1037.518794
conformer_93	-1039.231850	0.274434	0.210821	-1039.284144	-137.3	-1039.009710	-1039.070311	32.6	-1033.275626	-4.402738	-1037.516825
conformer_91	-1039.231734	0.274447	0.210280	-1039.283341	-135.5	-1039.008894	-1039.070049	33.3	-1033.275823	-4.402275	-1037.516414
conformer_24	-1039.234561	0.274811	0.211209	-1039.282816	-126.7	-1039.008005	-1039.068595	37.1	-1033.277824	-4.402849	-1037.514706
conformer_34	-1039.233360	0.274688	0.211212	-1039.281286	-125.8	-1039.006598	-1039.067062	41.1	-1033.276552	-4.402589	-1037.512843
conformer_20	-1039.231869	0.274747	0.211618	-1039.280897	-128.7	-1039.006150	-1039.066267	43.2	-1033.272975	-4.404196	-1037.511569

[a]: Single-point calculation in aqueous phase with SMD model.

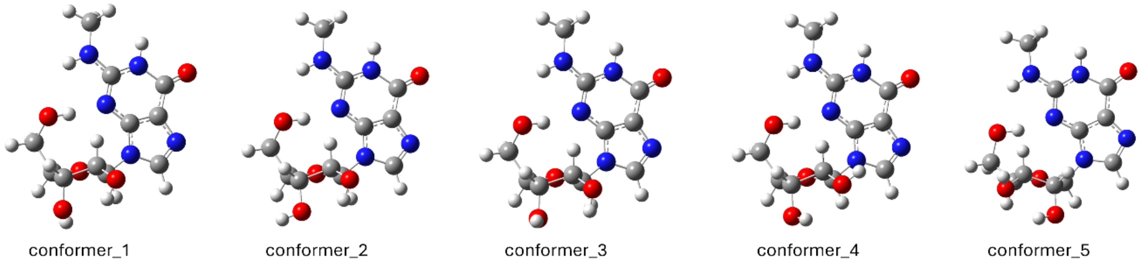

Figure S55. B3LYP-D3/def2-TZVPP optimized geometries of conformers for N^2-methylguanosine (**9rb2mG**).

Table S53. Conformers of gas-phase optimized N^2-methylguanosine (**9rb2mG**) at the B3LYP-D3/def2-TZVPP level of theory followed by aqueous phase single-point calculation. The columns display total energy without zero-point correction (E_{Tot}), Gibbs free energy (δG), total energy without zero-point correction ($E_{Tot,W}$), Gibbs free energy ($G_{298,W}$) in water (W), total single-point energy (E_{CBS}) calculated at DLPNO-CCSD(T)/CBS level of theory, and their corresponding free energy G_{CBS}. $G_{298,W}$ and G_{CBS} have been corrected to the standard state of 1 mol/L by addition of +7.908 kJ/mol. ΔG_{Solv} represents the Gibbs free energy of solvation. The data are arranged in the ascending numeric order of $E_{Tot,W}$. $\Delta G_{298,W}$ represents the respective energy difference to the lowest structure. Only conformers within the 24 kJ/mol (6 kcal/mol) energy window above the lowest in CREST are included in initial conformer sampling. Duplicates of the same structure are excluded.

9rb2mG No.	B3LYP-D3/def2-TZVPP E_{Tot} (Hartree)	δH (Hartree)	δG (Hartree)	SMD(H₂O)/B3LYP-D3/def2-TZVPP[a] $E_{Tot,W}$ (Hartree)	ΔG_{Solv} (kJ/mol)	$H_{298,W}$ (Hartree)	$G_{298,W}$ (Hartree)	$\Delta G_{298,W}$ (kJ/mol)	DLPNO-CCSD(T)/CBS $E_{CBS,HF}$ (Hartree)	$E_{CBS,C}$ (Hartree)	$G_{CBS,W}$ (Hartree)
**conformer_3**	**-1078.570203**	**0.304570**	**0.237662**	**-1078.620814**	**-132.9**	**-1078.316244**	**-1078.380140**	**0.0**	**-1072.330371**	**-4.610180**	**-1076.750488**
conformer_1	-1078.568949	0.304396	0.237629	-1078.620166	-134.5	-1078.315770	-1078.379525	1.6	-1072.327605	-4.611205	-1076.749386
conformer_2	-1078.570415	0.304363	0.237453	-1078.619853	-129.8	-1078.315490	-1078.379388	2.0	-1072.331042	-4.609921	-1076.749937
conformer_4	-1078.571083	0.304400	0.237369	-1078.619195	-126.3	-1078.314795	-1078.378814	3.5	-1072.332540	-4.609529	-1076.749801
conformer_5	-1078.568535	0.304400	0.237916	-1078.615392	-123.0	-1078.310992	-1078.374464	14.9	-1072.328989	-4.610578	-1076.745495
conformer_6	-1078.566685	0.304217	0.237160	-1078.615271	-127.6	-1078.311054	-1078.375099	13.2	-1072.327527	-4.609792	-1076.745733
conformer_7	-1078.564460	0.304242	0.237650	-1078.615044	-132.8	-1078.310802	-1078.374382	15.1	-1072.323282	-4.611292	-1076.744496
conformer_80	-1078.560555	0.304220	0.234711	-1078.614681	-142.1	-1078.310461	-1078.376958	8.4	-1072.327617	-4.604452	-1076.748472
conformer_27	-1078.562262	0.304437	0.237474	-1078.614571	-137.3	-1078.310134	-1078.374085	15.9	-1072.323212	-4.609982	-1076.745017
conformer_9	-1078.566461	0.304316	0.235746	-1078.614435	-126.0	-1078.310119	-1078.375677	11.7	-1072.330010	-4.607902	-1076.747127
conformer_69	-1078.559936	0.304234	0.234920	-1078.614342	-142.8	-1078.310108	-1078.376410	9.8	-1072.327777	-4.604004	-1076.748255
conformer_8	-1078.562928	0.304457	0.238172	-1078.614135	-134.4	-1078.309678	-1078.372951	18.9	-1072.322330	-4.611258	-1076.743611
conformer_13	-1078.563348	0.304338	0.237695	-1078.613906	-132.7	-1078.309568	-1078.373199	18.2	-1072.324933	-4.609780	-1076.744564
conformer_79	-1078.557755	0.304220	0.234871	-1078.613725	-147.0	-1078.309505	-1078.375842	11.3	-1072.322930	-4.605728	-1076.746745
conformer_31	-1078.563546	0.304255	0.237417	-1078.613238	-130.5	-1078.308983	-1078.372809	19.2	-1072.325531	-4.609582	-1076.744375
conformer_16	-1078.563931	0.304175	0.234611	-1078.613065	-129.0	-1078.308890	-1078.375442	12.3	-1072.328857	-4.606820	-1076.747188
conformer_36	-1078.559063	0.304293	0.234486	-1078.612506	-140.3	-1078.308213	-1078.375008	13.5	-1072.327382	-4.603607	-1076.746933
conformer_55	-1078.562375	0.304112	0.235406	-1078.612393	-131.3	-1078.308281	-1078.373975	16.2	-1072.326759	-4.607079	-1076.745438
conformer_14	-1078.562713	0.304143	0.235099	-1078.612113	-129.7	-1078.307970	-1078.374002	16.1	-1072.328622	-4.605847	-1076.745758
conformer_48	-1078.560740	0.304104	0.235172	-1078.612070	-134.8	-1078.307966	-1078.373886	16.4	-1072.326483	-4.606015	-1076.745644
conformer_24	-1078.564400	0.304210	0.235243	-1078.611893	-124.7	-1078.307683	-1078.373638	17.1	-1072.329759	-4.606675	-1076.745672
conformer_45	-1078.558582	0.304033	0.234035	-1078.611289	-138.4	-1078.307256	-1078.374242	15.5	-1072.327304	-4.603406	-1076.746369
conformer_50	-1078.562085	0.304079	0.234776	-1078.611012	-128.5	-1078.306933	-1078.373224	18.2	-1072.328614	-4.605553	-1076.745307
conformer_38	-1078.558839	0.304169	0.235292	-1078.610905	-136.7	-1078.306736	-1078.372601	19.8	-1072.325548	-4.605562	-1076.744872
conformer_51	-1078.554018	0.304104	0.234742	-1078.610803	-149.1	-1078.306699	-1078.373049	18.6	-1072.319658	-4.605866	-1076.744556
conformer_56	-1078.557934	0.304295	0.234763	-1078.610759	-138.7	-1078.306464	-1078.372984	18.8	-1072.325349	-4.604274	-1076.744673
conformer_43	-1078.556941	0.304121	0.234844	-1078.610754	-141.3	-1078.306633	-1078.372898	19.0	-1072.325045	-4.604077	-1076.745080
conformer_19	-1078.558291	0.304245	0.236124	-1078.610737	-137.7	-1078.306492	-1078.371601	22.4	-1072.319375	-4.610115	-1076.742800
conformer_15	-1078.555794	0.304324	0.235568	-1078.610484	-143.6	-1078.306160	-1078.371904	21.6	-1072.321019	-4.606128	-1076.743257

conformer_35	-1078.556260	0.304176	0.234333	-1078.610359	-142.0	-1078.306183	-1078.373014	18.7	-1072.321619	-4.605854	-1076.744228
conformer_63	-1078.557421	0.304072	0.234621	-1078.610332	-138.9	-1078.306260	-1078.372699	19.5	-1072.326061	-4.603780	-1076.745119
conformer_70	-1078.557813	0.304210	0.234018	-1078.610028	-137.1	-1078.305818	-1078.372998	18.8	-1072.327225	-4.602760	-1076.745170
conformer_42	-1078.555002	0.304240	0.234818	-1078.609576	-143.3	-1078.305336	-1078.371746	22.0	-1072.321307	-4.605131	-1076.743182
conformer_81	-1078.556617	0.304104	0.234820	-1078.609503	-138.9	-1078.305399	-1078.371671	22.2	-1072.325765	-4.603423	-1076.744242
conformer_49	-1078.559952	0.304307	0.235515	-1078.609498	-130.1	-1078.305191	-1078.370971	24.1	-1072.327677	-4.604471	-1076.743167
conformer_41	-1078.557907	0.304128	0.234383	-1078.609424	-135.3	-1078.305296	-1078.372029	21.3	-1072.325531	-4.604248	-1076.743901
conformer_29	-1078.555576	0.304135	0.234795	-1078.609248	-140.9	-1078.305113	-1078.371441	22.8	-1072.321296	-4.605535	-1076.742696
conformer_22	-1078.556078	0.304281	0.235101	-1078.608688	-138.1	-1078.304407	-1078.370575	25.1	-1072.319039	-4.608944	-1076.742479
conformer_61	-1078.560144	0.304301	0.235492	-1078.608312	-126.5	-1078.304011	-1078.369808	27.1	-1072.328806	-4.604078	-1076.742548
conformer_30	-1078.556065	0.304409	0.237709	-1078.607426	-134.8	-1078.303017	-1078.366705	35.3	-1072.314684	-4.612557	-1076.737880
conformer_47	-1078.555683	0.304163	0.236827	-1078.606833	-134.3	-1078.302670	-1078.366994	34.5	-1072.316647	-4.610175	-1076.738133
conformer_18	-1078.559110	0.304295	0.234620	-1078.606445	-124.3	-1078.302150	-1078.368813	29.7	-1072.325942	-4.605587	-1076.741231
conformer_76	-1078.555408	0.304099	0.236422	-1078.605598	-131.8	-1078.301499	-1078.366164	36.7	-1072.316638	-4.610193	-1076.737586
conformer_21	-1078.557937	0.304348	0.236630	-1078.604732	-122.9	-1078.300384	-1078.365090	39.5	-1072.318777	-4.610449	-1076.736379
conformer_26	-1078.557099	0.304297	0.236904	-1078.603188	-121.0	-1078.298891	-1078.363272	44.3	-1072.317796	-4.610085	-1076.734054
conformer_17	-1078.555665	0.304354	0.237284	-1078.602870	-123.9	-1078.298516	-1078.362574	46.1	-1072.314318	-4.611666	-1076.732893

[a]: Single-point calculation in aqueous phase with SMD model.

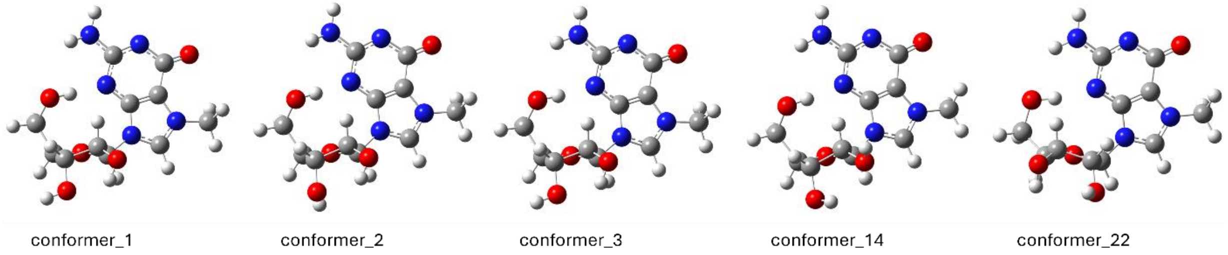

Figure S56. B3LYP-D3/def2-TZVPP optimized geometries of conformers for neutral N^7-methylguanosine (**9rb7mG**).

Table S54. Conformers of gas-phase optimized neutral N^7-methylguanosine (**9rb7mG**) at the B3LYP-D3/def2-TZVPP level of theory followed by aqueous phase single-point calculation. The columns display total energy without zero-point correction (E_{Tot}), Gibbs free energy (δG), total energy without zero-point correction ($E_{Tot,W}$), Gibbs free energy ($G_{298,W}$) in water (W), total single-point energy (E_{CBS}) calculated at DLPNO-CCSD(T)/CBS level of theory, and their corresponding free energy G_{CBS}. $G_{298,W}$ and G_{CBS} have been corrected to the standard state of 1 mol/L by addition of +7.908 kJ/mol. ΔG_{Solv} represents the Gibbs free energy of solvation. The data are arranged in the ascending numeric order of $E_{Tot,W}$. $\Delta G_{298,W}$ represents the respective energy difference to the lowest structure. Only conformers within the 24 kJ/mol (6 kcal/mol) energy window above the lowest in CREST are included in initial conformer sampling. Duplicates of the same structure are excluded.

9rb7mG	B3LYP-D3/def2-TZVPP			SMD(H2O)/B3LYP-D3/def2-TZVPP[a]					DLPNO-CCSD(T)/CBS		
No.	E_{Tot} (Hartree)	δH (Hartree)	δG (Hartree)	$E_{Tot,W}$ (Hartree)	ΔG_{Solv} (kJ/mol)	$H_{298,W}$ (Hartree)	$G_{298,W}$ (Hartree)	$\Delta G_{298,W}$ (kJ/mol)	$E_{CBS,HF}$ (Hartree)	$E_{CBS,C}$ (Hartree)	$G_{CBS,W}$ (Hartree)
conformer_14	-1078.553451	0.304028	0.237119	-1078.609466	-147.1	-1078.305438	-1078.369335	2.0	-1072.307898	-4.615636	-1076.739418
**conformer_1**	**-1078.552889**	**0.303883**	**0.236283**	**-1078.609389**	**-148.3**	**-1078.305506**	**-1078.370094**	**0.0**	**-1072.307027**	**-4.615543**	**-1076.739775**
conformer_2	-1078.550785	0.303874	0.236508	-1078.609262	-153.5	-1078.305388	-1078.369742	0.9	-1072.302793	-4.617061	-1076.738813
conformer_3	-1078.552900	0.303928	0.237030	-1078.609191	-147.8	-1078.305263	-1078.369149	2.5	-1072.306845	-4.615733	-1076.738827
conformer_22	-1078.547161	0.304108	0.236904	-1078.604757	-151.2	-1078.300649	-1078.364841	13.8	-1072.300882	-4.615759	-1076.734320
conformer_5	-1078.551262	0.303839	0.235808	-1078.604349	-139.4	-1078.300510	-1078.365529	12.0	-1072.308493	-4.613804	-1076.736564
conformer_4	-1078.549896	0.303919	0.236740	-1078.604311	-142.9	-1078.300392	-1078.364559	14.5	-1072.303810	-4.616296	-1076.734770
conformer_48	-1078.545233	0.304020	0.236161	-1078.603994	-154.3	-1078.299974	-1078.364821	13.8	-1072.300504	-4.615444	-1076.735535
conformer_15	-1078.544218	0.304019	0.235938	-1078.603950	-156.8	-1078.299931	-1078.365000	13.4	-1072.297211	-4.617062	-1076.735055
conformer_17	-1078.545246	0.304023	0.237151	-1078.603790	-153.7	-1078.299767	-1078.363627	17.0	-1072.300326	-4.615571	-1076.734277
conformer_30	-1078.544225	0.304022	0.236978	-1078.603775	-156.3	-1078.299753	-1078.363785	16.6	-1072.297099	-4.617195	-1076.733855
conformer_11	-1078.550187	0.303792	0.235108	-1078.603558	-140.1	-1078.299766	-1078.365438	12.2	-1072.308622	-4.612656	-1076.736529
conformer_13	-1078.549989	0.303743	0.235423	-1078.603439	-140.3	-1078.299696	-1078.365004	13.4	-1072.308151	-4.612771	-1076.735937
conformer_9	-1078.549338	0.303829	0.235608	-1078.603226	-141.5	-1078.299397	-1078.364606	14.4	-1072.307716	-4.612866	-1076.735850
conformer_10	-1078.548311	0.303821	0.235291	-1078.602968	-143.5	-1078.299147	-1078.364665	14.3	-1072.307927	-4.611693	-1076.735973
conformer_8	-1078.548505	0.303883	0.235256	-1078.602960	-143.0	-1078.299077	-1078.364692	14.2	-1072.308304	-4.611642	-1076.736133
conformer_7	-1078.548087	0.303797	0.235281	-1078.602860	-143.8	-1078.299063	-1078.364567	14.5	-1072.307167	-4.612015	-1076.735662
conformer_6	-1078.548751	0.303868	0.235542	-1078.602678	-141.6	-1078.298810	-1078.364124	15.7	-1072.308117	-4.611794	-1076.735285
conformer_12	-1078.551077	0.303840	0.235070	-1078.602607	-135.3	-1078.298767	-1078.364525	14.6	-1072.310565	-4.612238	-1076.736251
conformer_58	-1078.544355	0.304052	0.235458	-1078.602443	-152.5	-1078.298391	-1078.363973	16.1	-1072.307166	-4.608843	-1076.735627
conformer_63	-1078.542164	0.304161	0.235385	-1078.602420	-158.2	-1078.298259	-1078.364023	15.9	-1072.303599	-4.609928	-1076.735386
conformer_71	-1078.544119	0.304003	0.235409	-1078.602400	-153.0	-1078.298397	-1078.363979	16.1	-1072.306858	-4.608874	-1076.735592
conformer_38	-1078.547489	0.303754	0.235412	-1078.602316	-143.9	-1078.298562	-1078.363892	16.3	-1072.307846	-4.611386	-1076.735635
conformer_74	-1078.541924	0.304086	0.235270	-1078.602274	-158.4	-1078.298188	-1078.363992	16.0	-1072.303273	-4.609958	-1076.735299
conformer_18	-1078.548143	0.303804	0.235312	-1078.601575	-140.3	-1078.297771	-1078.363251	18.0	-1072.308234	-4.611434	-1076.734776
conformer_55	-1078.539454	0.304031	0.234354	-1078.599739	-158.3	-1078.295708	-1078.362373	20.3	-1072.300128	-4.610531	-1076.733579
conformer_44	-1078.540702	0.303889	0.233351	-1078.599352	-154.0	-1078.295463	-1078.362989	18.7	-1072.301427	-4.611010	-1076.734724
conformer_41	-1078.541052	0.303945	0.234732	-1078.599151	-152.5	-1078.295206	-1078.361407	22.8	-1072.301518	-4.611297	-1076.733169
conformer_53	-1078.538345	0.304010	0.233922	-1078.598907	-159.0	-1078.294897	-1078.361973	21.3	-1072.299208	-4.610254	-1076.733090

conformer_66	-1078.537102	0.303635	0.232886	-1078.598660	-161.6	-1078.295025	-1078.362762	19.2	-1072.298549	-4.610467	-1076.734676
conformer_37	-1078.538262	0.303684	0.234354	-1078.598631	-158.5	-1078.294947	-1078.361265	23.2	-1072.299481	-4.610467	-1076.732952
conformer_69	-1078.537097	0.303634	0.232426	-1078.598618	-161.5	-1078.294984	-1078.363180	18.2	-1072.298079	-4.610740	-1076.734903
conformer_65	-1078.535529	0.303825	0.234795	-1078.598575	-165.5	-1078.294750	-1078.360768	24.5	-1072.295029	-4.611398	-1076.731665
conformer_39	-1078.538398	0.303736	0.234493	-1078.598365	-157.4	-1078.294629	-1078.360860	24.2	-1072.299694	-4.610434	-1076.732590
conformer_20	-1078.536162	0.303753	0.234734	-1078.598132	-162.7	-1078.294379	-1078.360386	25.5	-1072.294307	-4.612807	-1076.731338
conformer_21	-1078.536313	0.303786	0.234768	-1078.598010	-162.0	-1078.294224	-1078.360230	25.9	-1072.294637	-4.612623	-1076.731177
conformer_57	-1078.535155	0.303636	0.233470	-1078.597740	-164.3	-1078.294104	-1078.361258	23.2	-1072.293935	-4.612360	-1076.732399
conformer_64	-1078.538565	0.304002	0.234370	-1078.597680	-155.2	-1078.293678	-1078.360298	25.7	-1072.299015	-4.611064	-1076.731812
conformer_24	-1078.539282	0.303924	0.234976	-1078.597184	-152.0	-1078.293260	-1078.359196	28.6	-1072.301445	-4.609885	-1076.731245
conformer_23	-1078.537728	0.303928	0.235967	-1078.596805	-155.1	-1078.292877	-1078.357826	32.2	-1072.291513	-4.617416	-1076.729027
conformer_31	-1078.537501	0.303839	0.235741	-1078.596800	-155.7	-1078.292961	-1078.358047	31.6	-1072.291397	-4.617246	-1076.729188
conformer_42	-1078.541035	0.303922	0.233865	-1078.596799	-146.4	-1078.292877	-1078.359922	26.7	-1072.301619	-4.611069	-1076.731575
conformer_76	-1078.535339	0.303835	0.234765	-1078.596720	-161.2	-1078.292885	-1078.358943	29.3	-1072.290747	-4.615813	-1076.730164
conformer_62	-1078.541033	0.303921	0.234482	-1078.596601	-145.9	-1078.292680	-1078.359107	28.8	-1072.301461	-4.611224	-1076.730758
conformer_29	-1078.540992	0.303876	0.234687	-1078.596541	-145.8	-1078.292665	-1078.358842	29.5	-1072.301342	-4.611238	-1076.730431
conformer_26	-1078.536741	0.303836	0.234484	-1078.596397	-156.6	-1078.292561	-1078.358901	29.4	-1072.296112	-4.612163	-1076.730436
conformer_25	-1078.542065	0.304046	0.234955	-1078.595702	-140.8	-1078.291656	-1078.357735	32.4	-1072.303489	-4.610728	-1076.729888
conformer_40	-1078.533869	0.303801	0.233145	-1078.594832	-160.1	-1078.291031	-1078.358675	30.0	-1072.290660	-4.614429	-1076.729895
conformer_45	-1078.533877	0.303801	0.233706	-1078.594575	-159.4	-1078.290774	-1078.357857	32.1	-1072.290621	-4.614464	-1076.729064

[a]: Single-point calculation in aqueous phase with SMD model.

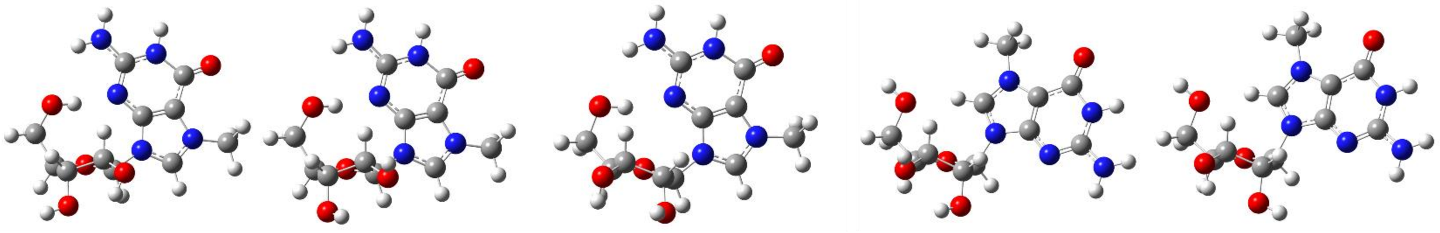

conformer_1 conformer_11 conformer_21 conformer_31 conformer_32

Figure S57. B3LYP-D3/def2-TZVPP optimized geometries of conformers for protonated N^7-methylguanosine cation (**9rb7mG⁺**).

Table S55. Conformers of gas-phase optimized protonated N^7-methylguanosine cation (**9rb7mG$^+$**) at the B3LYP-D3/def2-TZVPP level of theory followed by aqueous phase single-point calculation. The columns display total energy without zero-point correction (E_{Tot}), Gibbs free energy (δG), total energy without zero-point correction ($E_{Tot,W}$), Gibbs free energy ($G_{298,W}$) in water (W), total single-point energy (E_{CBS}) calculated at DLPNO-CCSD(T)/CBS level of theory, and their corresponding free energy G_{CBS}. $G_{298,W}$ and G_{CBS} have been corrected to the standard state of 1 mol/L by addition of +7.908 kJ/mol. ΔG_{Solv} represents the Gibbs free energy of solvation. The data are arranged in the ascending numeric order of $E_{Tot,W}$. $\Delta G_{298,W}$ represents the respective energy difference to the lowest structure. Only conformers within the 24 kJ/mol (6 kcal/mol) energy window above the lowest in CREST are included in initial conformer sampling. Duplicates of the same structure are excluded.

9rb7mG	B3LYP-D3/def2-TZVPP			SMD(H₂O)/B3LYP-D3/def2-TZVPP[a]					DLPNO-CCSD(T)/CBS		
No.	E_{Tot} (Hartree)	δH (Hartree)	δG (Hartree)	$E_{Tot,W}$ (Hartree)	ΔG_{Solv} (kJ/mol)	$H_{298,W}$ (Hartree)	$G_{298,W}$ (Hartree)	$\Delta G_{298,W}$ (kJ/mol)	$E_{CBS,HF}$ (Hartree)	$E_{CBS,C}$ (Hartree)	$G_{CBS,W}$ (Hartree)
conformer_11	-1078.966164	0.317890	0.250949	-1079.075998	-288.4	-1078.758108	-1078.822037	0.8	-1072.733128	-4.598454	-1077.187455
**conformer_1**	**-1078.968371**	**0.317803**	**0.250546**	**-1079.075891**	**-282.3**	**-1078.758088**	**-1078.822333**	**0.0**	**-1072.734998**	**-4.598637**	**-1077.187598**
conformer_32	-1078.967500	0.317272	0.247911	-1079.071798	-273.8	-1078.754526	-1078.820875	3.8	-1072.741061	-4.592994	-1077.187431
conformer_31	-1078.968471	0.317259	0.247860	-1079.071702	-271.0	-1078.754443	-1078.820830	3.9	-1072.741753	-4.593254	-1077.187366
conformer_21	-1078.960692	0.317758	0.250045	-1079.071691	-291.4	-1078.753933	-1078.818634	9.7	-1072.727118	-4.598687	-1077.183747
conformer_23	-1078.964037	0.317291	0.248137	-1079.071254	-281.5	-1078.753963	-1078.820105	5.9	-1072.735430	-4.594398	-1077.185895
conformer_63	-1078.951877	0.317043	0.247025	-1079.070948	-312.6	-1078.753905	-1078.820911	3.7	-1072.723320	-4.594101	-1077.186454
conformer_13	-1078.962993	0.317604	0.249855	-1079.070929	-283.4	-1078.753325	-1078.818062	11.2	-1072.729337	-4.599183	-1077.183589
conformer_26	-1078.964518	0.317316	0.247723	-1079.070862	-279.2	-1078.753546	-1078.820127	5.8	-1072.735993	-4.593919	-1077.185521
conformer_33	-1078.968139	0.317331	0.247789	-1079.070809	-269.6	-1078.753478	-1078.820008	6.1	-1072.741619	-4.592602	-1077.186090
conformer_5	-1078.967085	0.317455	0.248506	-1079.070458	-271.4	-1078.753003	-1078.818940	8.9	-1072.738850	-4.594882	-1077.185587
conformer_40	-1078.956738	0.317296	0.247846	-1079.070306	-298.2	-1078.753010	-1078.819448	7.6	-1072.728005	-4.594962	-1077.185678
conformer_22	-1078.955386	0.317496	0.250047	-1079.070300	-301.7	-1078.752804	-1078.817241	13.4	-1072.719561	-4.600399	-1077.181815
conformer_29	-1078.962401	0.317573	0.250238	-1079.070155	-282.9	-1078.752582	-1078.816905	14.3	-1072.729420	-4.598684	-1077.182608
conformer_50	-1078.959494	0.317456	0.250103	-1079.069826	-289.7	-1078.752370	-1078.816711	14.8	-1072.726788	-4.598565	-1077.182571
conformer_64	-1078.956902	0.317216	0.247884	-1079.069675	-296.1	-1078.752459	-1078.818779	9.3	-1072.728821	-4.594233	-1077.184931
conformer_48	-1078.959152	0.317363	0.248310	-1079.069446	-289.6	-1078.752083	-1078.818124	11.1	-1072.731388	-4.594009	-1077.184369
conformer_61	-1078.955278	0.317272	0.247202	-1079.068954	-298.5	-1078.751682	-1078.818740	9.4	-1072.728863	-4.592457	-1077.184782
conformer_62	-1078.952013	0.317238	0.247575	-1079.068781	-306.6	-1078.751543	-1078.818194	10.9	-1072.723063	-4.594225	-1077.183470
conformer_53	-1078.959164	0.317336	0.247474	-1079.067942	-285.6	-1078.750606	-1078.817456	12.8	-1072.732485	-4.593218	-1077.183995
conformer_87	-1078.948536	0.317302	0.247398	-1079.066123	-308.7	-1078.748821	-1078.815713	17.4	-1072.719021	-4.595025	-1077.181223
conformer_82	-1078.959550	0.317340	0.247392	-1079.065050	-277.0	-1078.747710	-1078.814646	20.2	-1072.733618	-4.593145	-1077.181858

[a]: Single-point calculation in aqueous phase with SMD model.

S3 Results and Discussion

S3.1 Four Reaction Categories

The conformers with the lowest $G_{298,W}$ were taken for each species for the further calculation of thermodynamic driving force of natural decomposition in water. The energies of other basic molecules without conformers were taken directly for the calculation. Based on different types of decomposition, the reactions are divided into four categories: deglycosylation, deamination, deacetylation, desulfurization.

S3.1.1Deglycosylation

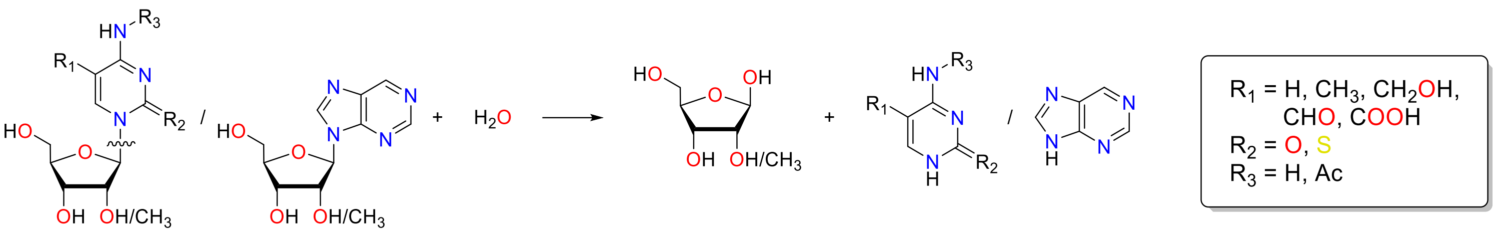

Figure S58. Deglycosylation reaction of modified cytidines and uridines.

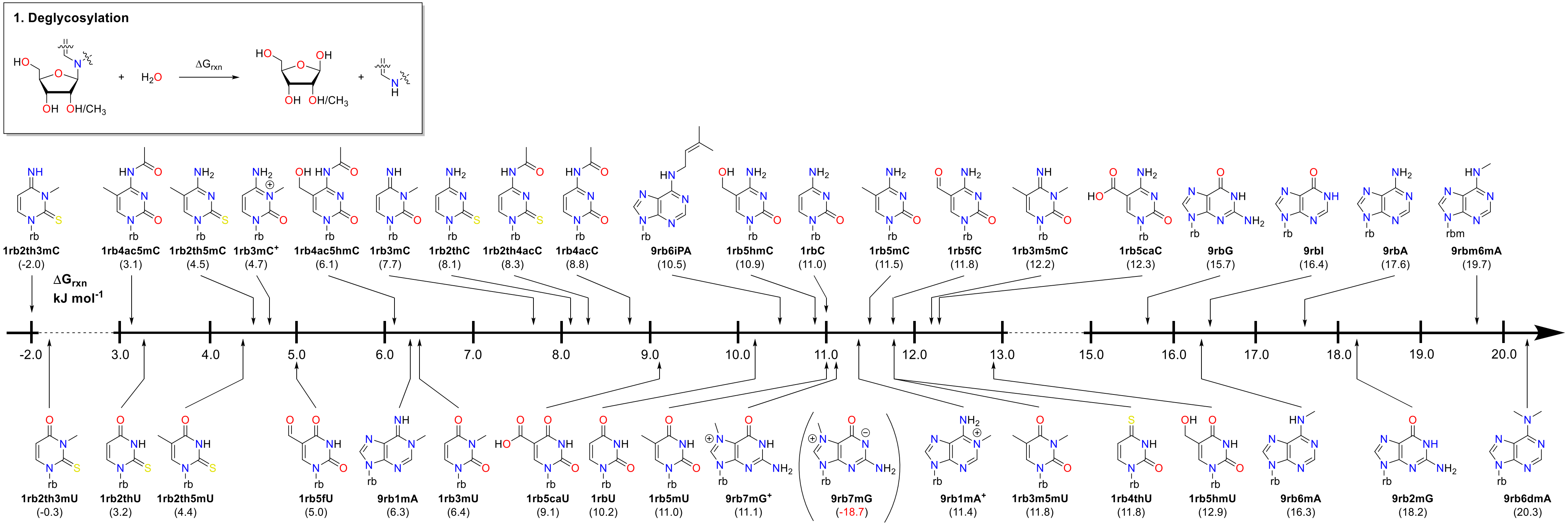

Figure S59. Reaction energy comparison for deglycosylation of different nucleosides calculated at DLPNO-CCSD(T)/CBS//SMD(H_2O)/B3LYP-D3/def2-TZVPP level of theory.

Table S56. Energetics for the deglycosylation. $\Delta E_{Rxn,gas}$, $\Delta G_{Rxn,gas}$, and ΔG_{Rxn} represent the total energy difference in the gas phase and the Gibbs free difference in both gas and aqueous phase for the deglycosylation reaction.

Reactant Nucleotide	Product Nucleobase	$\Delta E_{Rxn,gas}$ (kJ/mol)	$\Delta G_{Rxn,gas}$ (kJ/mol)	ΔG_{Rxn} (kJ/mol)
**Depyrimidination**				
**1rbC**	**C**	+36.9	+29.8	+11.0
**1rb2thC**	**2thC**	+30.5	+25.9	+8.1
**1rb2th3mC**	**2th3mC**	-8.1	-11.9	-2.0
**1rb2th4acC**	**2th4acC**	+29.6	+24.2	+8.3
**1rb2th5mC**	**2th5mC**	+25.6	+18.3	+4.5
**1rb3mC**	**3mC**	+21.5	+12.7	+7.7
**1rb3mC$^+$**	**3mC$^+$**	+19.4	+16.4	+4.7
**1rb3m5mC**	**3m5mC**	+5.7	+5.3	+12.2
**1rb4acC**	**4acC**	+35.8	+26.8	+8.8
**1rb4ac5mC**	**4ac5mC**	+67.4	+51.3	+3.1
**1rb4ac5hmC**	**4ac5hmC**	+58.8	+47.7	+6.1
**1rb5mC**	**5mC**	+38.4	+31.1	+11.5
**1rb5hmC**	**5hmC**	+37.8	+30.5	+10.9
**1rb5fC**	**5fC**	+15.7	+14.8	+11.8
**1rb5caC**	**5caC**	+37.9	+30.6	+12.3
**1rbU**	**U**	+9.0	+5.4	+10.2
**1rb2thU**	**2thU**	-4.4	-6.3	+3.2
**1rb2th3mU**	**2th3mU**	+17.1	+10.8	-0.3
**1rb2th5mU**	**2th5mU**	-3.4	-5.8	+4.4
**1rb3mU**	**3mU**	+21.7	+12.6	+6.4
**1rb3m5mU**	**3m5mU**	+5.2	+6.1	+11.8
**1rb4thU**	**4thU**	+10.0	+7.8	+11.8
**1rb5mU**	**5mU**	+9.8	+6.3	+11.0
**1rb5hmU**	**5hmU**	+9.0	+8.3	+12.9
**1rb5fU**	**5fU**	+21.2	+15.5	+5.0
**1rb5caU**	**5caU**	+14.6	+10.4	+9.1
**Depurination**				
**9rbA**	**A**	+40.9	+29.4	+17.6
**9rb1mA**	**1mA**	+66.0	+54.5	+6.3
**9rb1mA$^+$**	**1mA$^+$**	+39.8	+30.6	+11.4
**9rb6mA**	**6mA**	+41.6	+28.1	+16.3
**9rb6dmA**	**6dmA**	+39.8	+28.7	+20.3
**9rbm6mA**	**6mA**	+43.4	+31.1	+19.7
**9rb6iPA**	**6iPA**	+29.6	+19.2	+10.5
**9rbG**	**G**	+33.2	+21.5	+15.7
**9rb2mG**	**2mG**	+40.2	+26.8	+18.2
**9rb7mG**	**7mG**	-24.6	-33.3	-18.7
**9rb7mG$^+$**	**7mG$^+$**	+53.1	+41.6	+11.1
**9rbI**	**I**	+32.9	+22.8	+16.4

S3.1.2Deamination

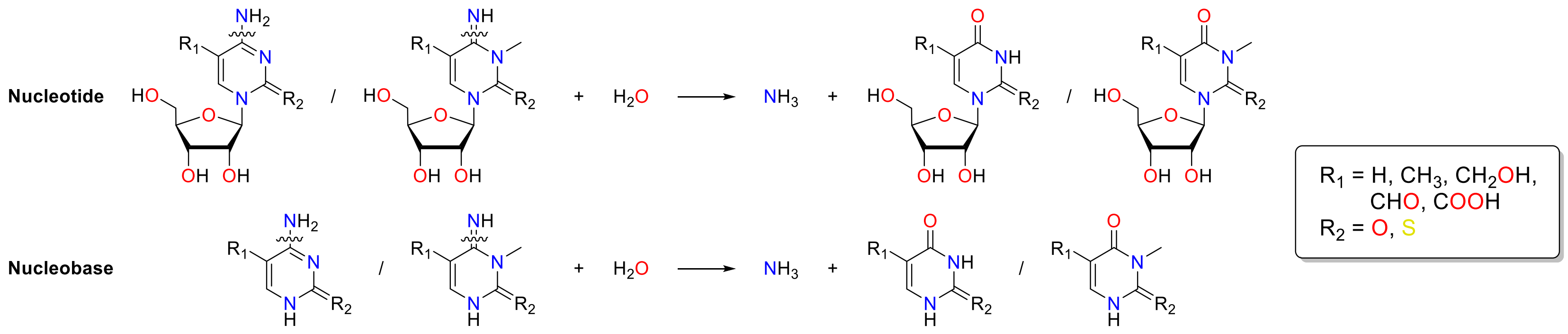

Figure S60. Deamination reaction of modified cytidines.

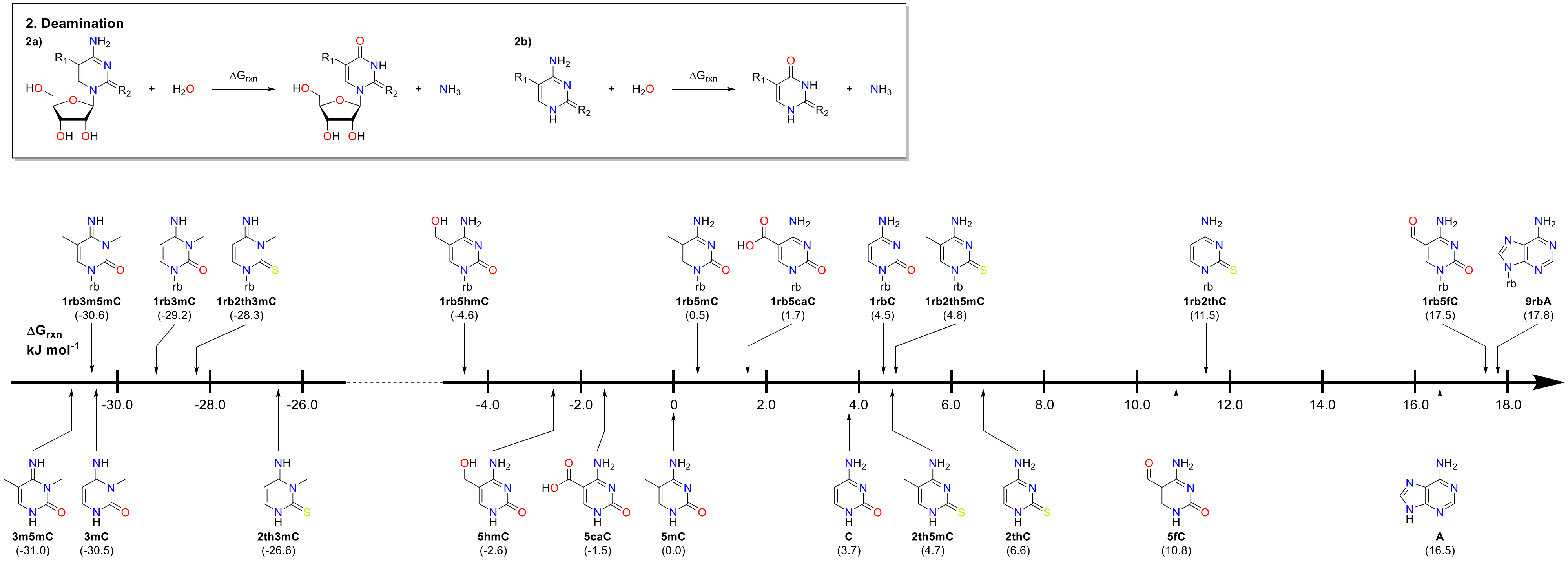

Figure S61. Reaction energy comparison for deamination of different nucleosides and their corresponding nucleobases calculated at DLPNO-CCSD(T)/CBS//SMD(H_2O)/B3LYP-D3/def2-TZVPP level of theory.

Table S57. Energetics for the deamination. $\Delta E_{Rxn,gas}$, $\Delta G_{Rxn,gas}$, and ΔG_{Rxn} represent the total energy difference in the gas phase and the Gibbs free difference in both gas and aqueous phase for the deamination reaction.

Nucleotide		$\Delta E_{Rxn,gas}$	$\Delta G_{Rxn,gas}$	ΔG_{Rxn}
**Reactant**	**Product**	(kJ/mol)	(kJ/mol)	(kJ/mol)
**1rbC**	**1rbU**	-11.3	-12.2	+4.5
**1rb2thC**	**1rb2thU**	-3.3	-2.0	+11.5
**1rb2th5mC**	**1rb2th5mU**	-14	-15.3	+4.8
**1rb5mC**	**1rb5mU**	-15.7	-17.9	+0.5
**1rb5hmC**	**1rb5hmU**	-11.6	-18.8	-4.6
**1rb5fC**	**1rb5fU**	-2.3	-1.6	+17.5
**1rb5caC**	**1rb5caU**	-5.1	-7.2	+1.7
**1rb2th3mC**	**1rb2th3mU**	-69.8	-67.2	-28.3
**1rb3mC**	**1rb3mU**	-42.5	-46.2	-29.2
**1rb3m5mC**	**1rb3m5mU**	-43.5	-49.0	-30.6
**9rbA**	**9rbI**	+22.7	+23.2	+17.8
**Nucleobase**		$\Delta E_{Rxn,gas}$	$\Delta G_{Rxn,gas}$	ΔG_{Rxn}
**Reactant**	**Product**	(kJ/mol)	(kJ/mol)	(kJ/mol)
**C**	**U**	-39.2	-36.6	+3.7
**2thC**	**2thU**	-38.2	-34.2	+6.6
**2th5mC**	**2th5mU**	-43.0	-39.4	+4.7
**5mC**	**5mU**	-44.2	-42.8	+0.1
**5hmC**	**5hmU**	-40.4	-41.1	-2.6
**5fC**	**5fU**	+3.2	-0.8	+10.8
**5caC**	**5caU**	-28.4	-27.3	-1.5
**2th3mC**	**2th3mU**	-44.6	-44.5	-26.6
**3mC**	**3mU**	-42.3	-46.4	-30.5
**3m5mC**	**3m5mU**	-44.0	-48.2	-31.0
**A**	**I**	+14.6	+16.6	+16.5

S3.1.3Deacetylation

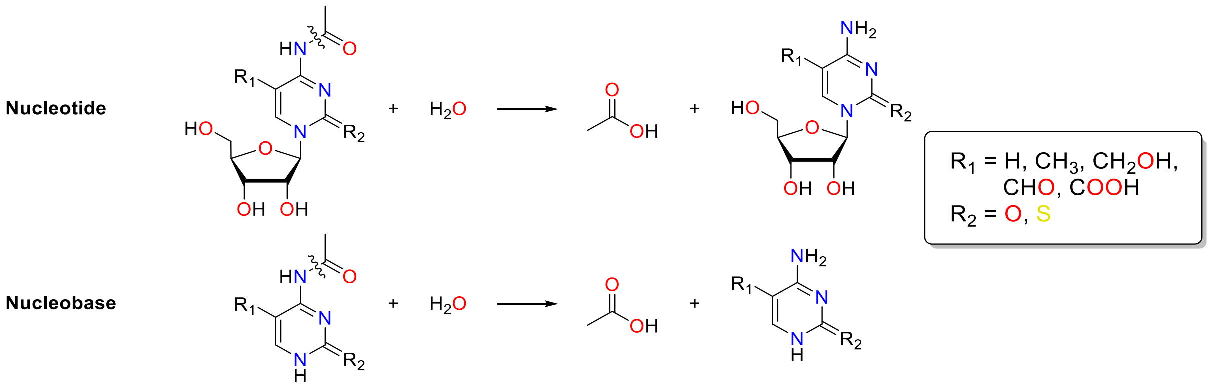

Figure S62. Deamination reaction of modified cytidine.

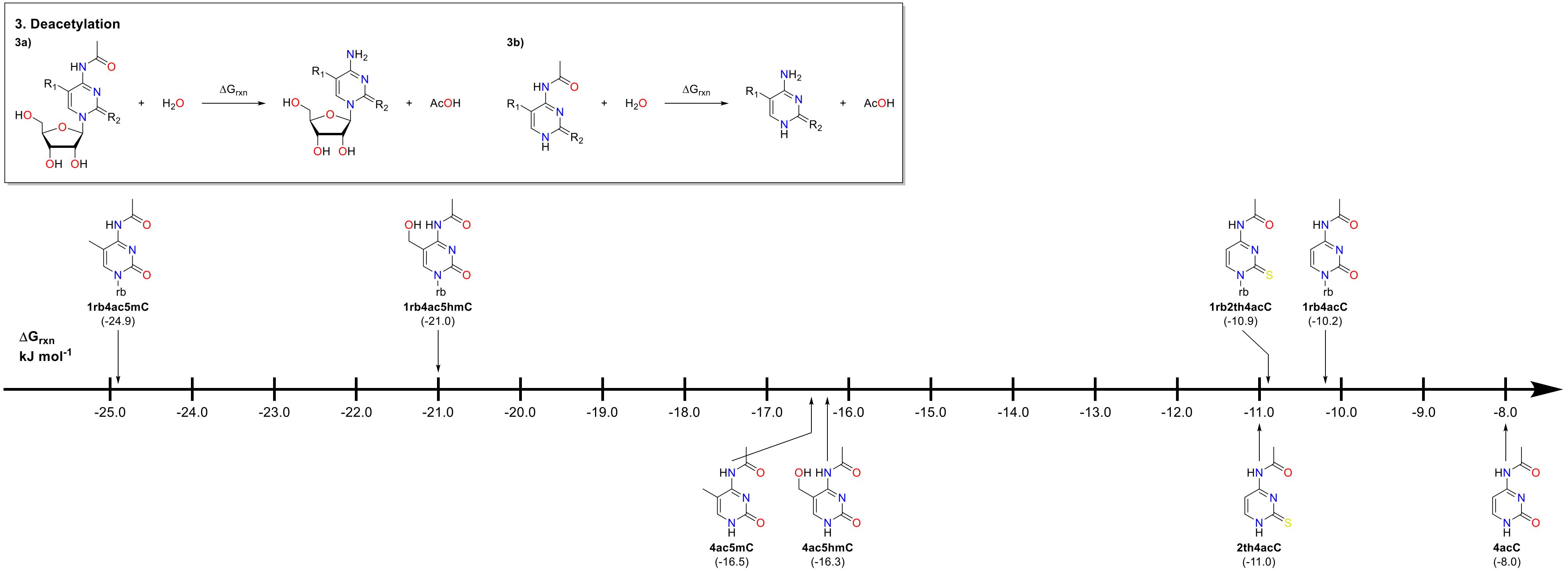

Figure S63. Reaction energy comparison for deacetylation of different nucleosides and their corresponding nucleobases calculated at DLPNO-CCSD(T)/CBS//SMD(H_2O)/B3LYP-D3/def2-TZVPP level of theory.

Table S58. Energetics for the deacetylation. $\Delta E_{Rxn,gas}$, $\Delta G_{Rxn,gas}$, and ΔG_{Rxn} represent the total energy difference in the gas phase and the Gibbs free difference in both gas and aqueous phase for the deacetylation reaction.

Nucleotide		$\Delta E_{Rxn,gas}$	$\Delta G_{Rxn,gas}$	ΔG_{Rxn}
**Reactant**	**Product**	(kJ/mol)	(kJ/mol)	(kJ/mol)
**1rb2th4acC**	**1rb2thC**	-7.4	-15.3	-10.9
**1rb4acC**	**1rbC**	-7.4	-14.8	-10.2
**1rb4ac5mC**	**1rb5mC**	-18.7	-27.6	-24.9
**1rb4ac5hmC**	**1rb5hmC**	-23.9	-28.5	-21.0
**Nucleobase**		$\Delta E_{Rxn,gas}$	$\Delta G_{Rxn,gas}$	ΔG_{Rxn}
**Reactant**	**Product**	(kJ/mol)	(kJ/mol)	(kJ/mol)
**2th4acC**	**2thC**	-6.5	-13.6	-11.0
**4acC**	**C**	-6.2	-11.7	-8.0
**4ac5mC**	**5mC**	-47.7	-47.8	-16.5
**4ac5hmC**	**5hmC**	-44.9	-45.7	-16.3

S3.1.4Desulfurization

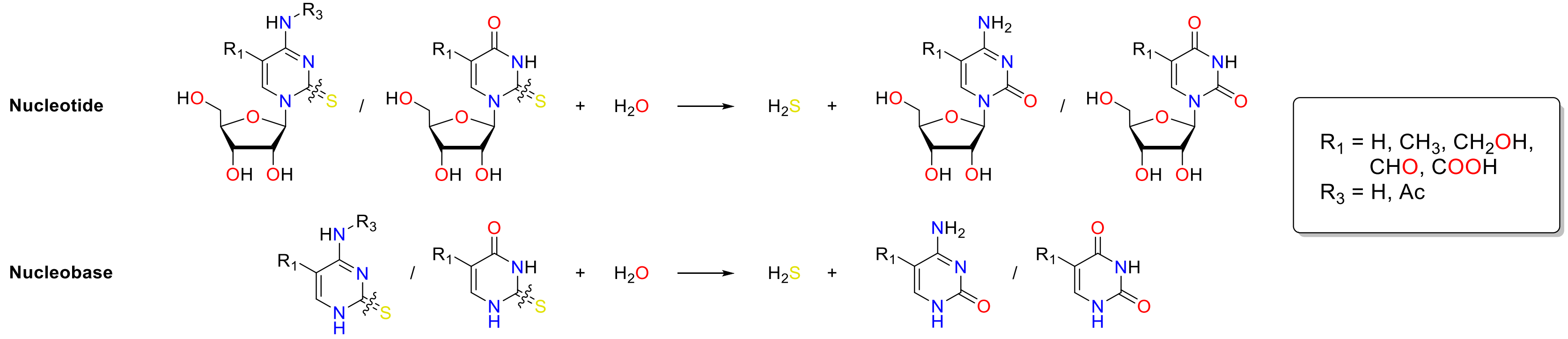

Figure S64. Desulfurization reaction of modified cytidines and uridines.

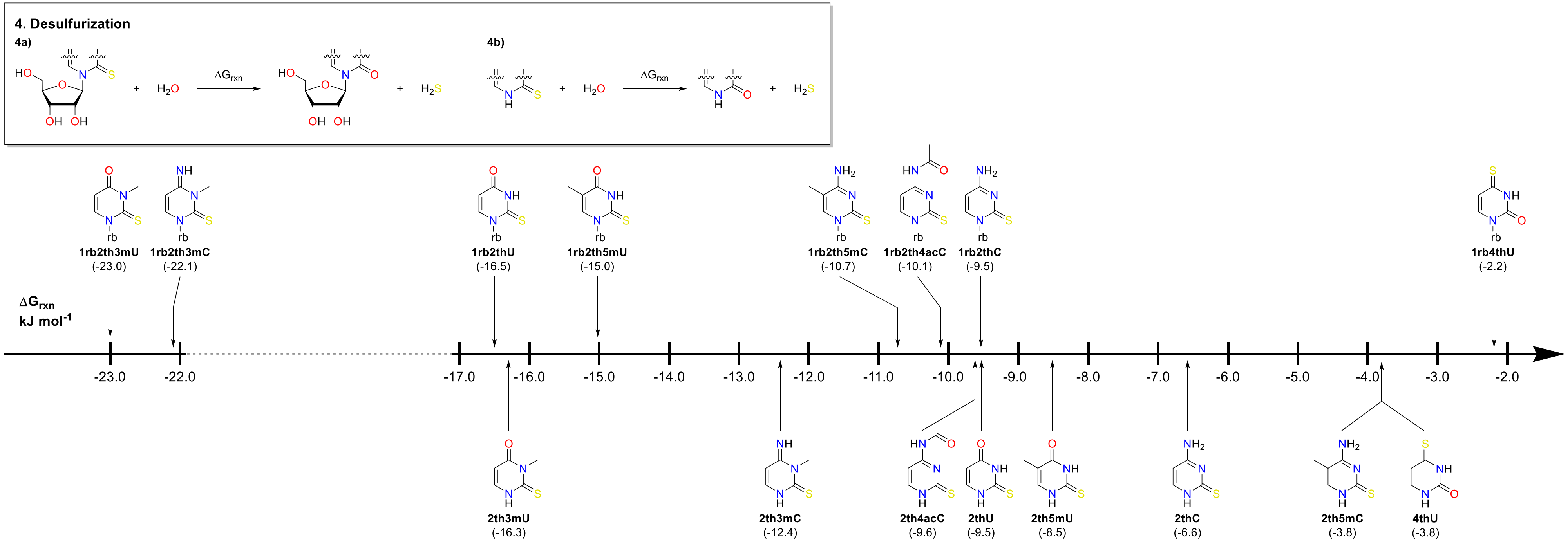

Figure S65. Reaction energy comparison for desulfurization of different nucleosides and their corresponding nucleobases calculated at DLPNO-CCSD(T)/CBS//SMD(H_2O)/B3LYP-D3/def2-TZVPP level of theory.

Table S59. Energetics for the desulfurization. $\Delta E_{Rxn,gas}$, $\Delta G_{Rxn,gas}$, and ΔG_{Rxn} represent the total energy difference in the gas phase and the Gibbs free difference in both gas and aqueous phase for the desulfurization reaction.

Nucleotide		$\Delta E_{Rxn,gas}$	$\Delta G_{Rxn,gas}$	ΔG_{Rxn}
**Reactant**	**Product**	(kJ/mol)	(kJ/mol)	(kJ/mol)
**1rb2thC**	**1rbC**	-24.3	-33.8	-9.5
**1rb2th3mC**	**1rb3mC**	-51.9	-58.8	-22.1
**1rb2th4acC**	**1rb4acC**	-24.3	-34.4	-10.1
**1rb2th5mC**	**1rb5mC**	-28.7	-40.4	-10.7
**1rb2thU**	**1rbU**	-32.3	-44.0	-16.5
**1rb2th3mU**	**1rb3mU**	-24.6	-37.9	-23.0
**1rb2th5mU**	**1rb5mU**	-30.4	-43.0	-15.0
**Nucleobase**		$\Delta E_{Rxn,gas}$	$\Delta G_{Rxn,gas}$	ΔG_{Rxn}
**Reactant**	**Product**	(kJ/mol)	(kJ/mol)	(kJ/mol)
**2thC**	**C**	-17.9	-29.9	-6.6
**2th3mC**	**3mC**	-22.3	-34.3	-12.4
**2th4acC**	**4acC**	-18.2	-31.8	-9.6
**2th5mC**	**5mC**	-16.0	-27.6	-3.8
**2thU**	**U**	-18.9	-32.4	-9.5
**2th3mU**	**3mu**	-20.0	-36.1	-16.3
**2th5mU**	**5mU**	-17.2	-30.9	-8.5

S3.2 Corresponding Computational Results to Experimental Findings

S3.2.1Degradation of 4-thiouridine

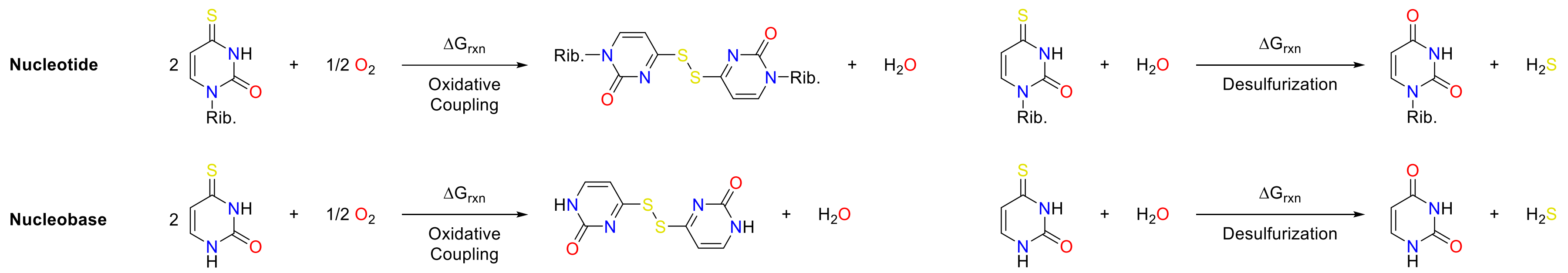

Figure S66. Reaction equation for the degradation of 4-thiouridine (**s⁴U**).

Table S60. Energetics for the degradation of 4-thiouridine (**s⁴U**). $\Delta E_{Rxn,gas}$, $\Delta G_{Rxn,gas}$, and ΔG_{Rxn} represent the total energy difference in the gas phase and the Gibbs free difference in both gas and aqueous phase for the deglycosylation reaction.

Nucleotide			$\Delta E_{Rxn,gas}$	$\Delta G_{Rxn,gas}$	ΔG_{Rxn}
**Reactant**	**Product**	**Reaction Type**	(kJ/mol)	(kJ/mol)	(kJ/mol)
**s⁴U**	**s⁴U_dimer**	Oxidative Coupling	-215.0	-154.4	-121.6
**s⁴U**	**U**	Desulfurization	-11.7	-24.0	-2.2
**Nucleobase**			$\Delta E_{Rxn,gas}$	$\Delta G_{Rxn,gas}$	ΔG_{Rxn}
**Reactant**	**Product**	**Reaction Type**	(kJ/mol)	(kJ/mol)	(kJ/mol)
**s⁴U**	**s⁴U_dimer**	Oxidative Coupling	-108.0	-90.9	-133.4
**s⁴U**	**U**	Desulfurization	-12.8	-26.4	-3.8

S3.2.2Degradation of 3-methylcytidine

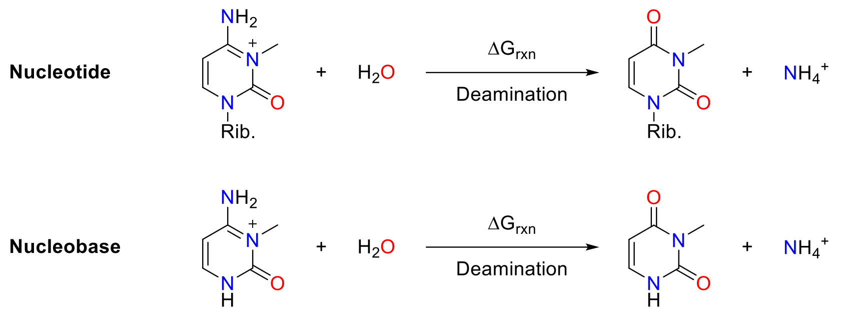

Figure S67. Reaction equation for the degradation of 3-methylcytidine (**m³C**).

Table S61. Energetics for the degradation of 3-methylcytidine (**m³C**). $\Delta E_{Rxn,gas}$, $\Delta G_{Rxn,gas}$, and ΔG_{Rxn} represent the total energy difference in the gas phase and the Gibbs free difference in both gas and aqueous phase for the deglycosylation reaction.

**Nucleotide**			**$\Delta E_{Rxn,gas}$**	**$\Delta G_{Rxn,gas}$**	**ΔG_{Rxn}**
**Reactant**	**Product**	**Reaction Type**	(kJ/mol)	(kJ/mol)	(kJ/mol)
**m³C**	**m³U**	Deamination	71.7	82.7	-21.3
**Nucleobase**			**$\Delta E_{Rxn,gas}$**	**$\Delta G_{Rxn,gas}$**	**ΔG_{Rxn}**
**Reactant**	**Product**	**Reaction Type**	(kJ/mol)	(kJ/mol)	(kJ/mol)
**m³C**	**m³U**	Deamination	74.1	78.9	-19.5

S3.2.3Degradation of N^6-isoprenyladenosine

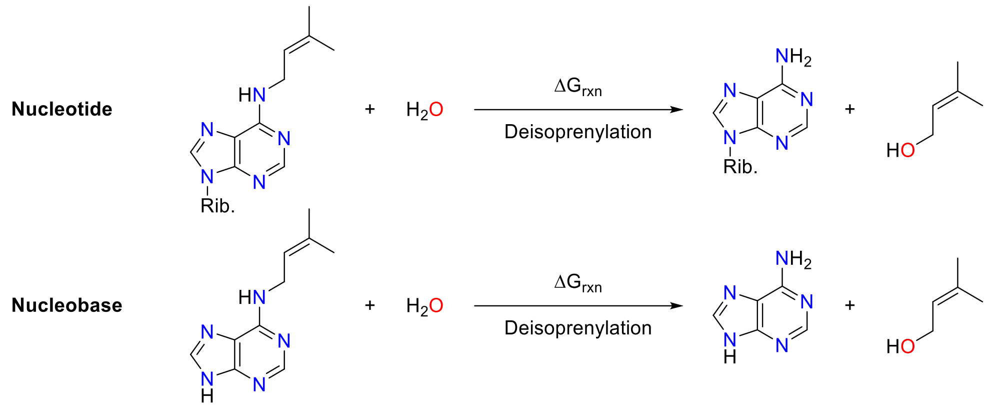

Figure S68. Reaction equation for the degradation of N^6-isoprenyladenosine (**i^{6}A**).

Table S62. Energetics for the degradation of N^6-isoprenyladenosine (**i^{6}A**). $\Delta E_{Rxn,gas}$, $\Delta G_{Rxn,gas}$, and ΔG_{Rxn} represent the total energy difference in the gas phase and the Gibbs free difference in both gas and aqueous phase for the deglycosylation reaction.

**Nucleotide**			**$\Delta E_{Rxn,gas}$**	**$\Delta G_{Rxn,gas}$**	**ΔG_{Rxn}**
**Reactant**	**Product**	**Reaction Type**	(kJ/mol)	(kJ/mol)	(kJ/mol)
**i^{6}A**	**A**	Deisoprenylation	19.5	16.0	24.5
**Nucleobase**			**$\Delta E_{Rxn,gas}$**	**$\Delta G_{Rxn,gas}$**	**ΔG_{Rxn}**
**Reactant**	**Product**	**Reaction Type**	(kJ/mol)	(kJ/mol)	(kJ/mol)
**i^{6}A**	**A**	Deisoprenylation	30.8	26.3	31.6

S3.2.4Degradation of *N*4-acetylcytidine

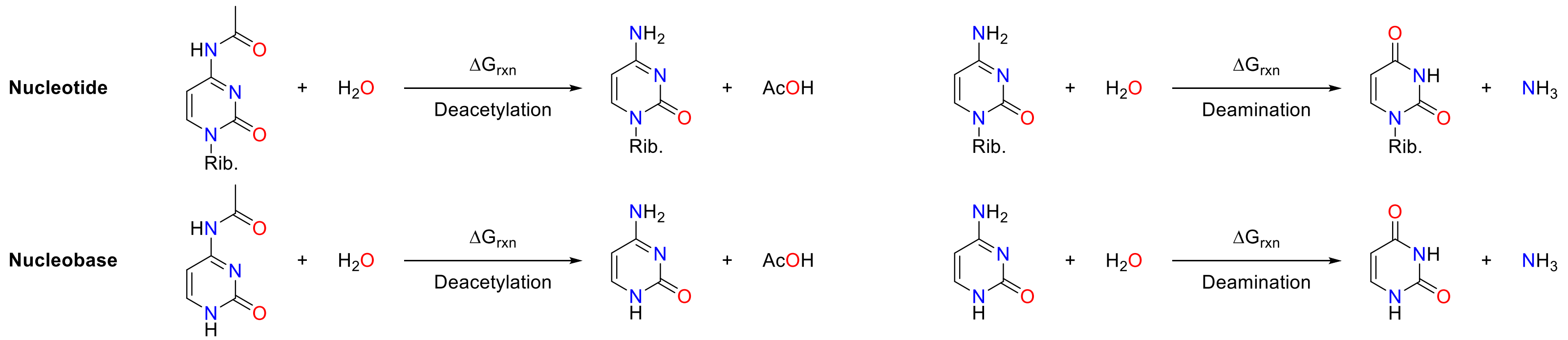

Figure S69. Reaction equation for the degradation of *N*4-acetylcytidine (**ac⁴C**).

Table S63. Energetics for the degradation of *N*4-acetylcytidine (**ac⁴C**). $\Delta E_{Rxn,gas}$, $\Delta G_{Rxn,gas}$, and ΔG_{Rxn} represent the total energy difference in the gas phase and the Gibbs free difference in both gas and aqueous phase for the deglycosylation reaction.

Nucleotide			$\Delta E_{Rxn,gas}$	$\Delta G_{Rxn,gas}$	ΔG_{Rxn}
**Reactant**	**Product**	**Reaction Type**	(kJ/mol)	(kJ/mol)	(kJ/mol)
**ac⁴C**	**C**	Deacetylation	-7.4	-14.8	-10.2
**C**	**U**	Deamination	-11.3	-12.2	4.5
**Nucleobase**			$\Delta E_{Rxn,gas}$	$\Delta G_{Rxn,gas}$	ΔG_{Rxn}
**Reactant**	**Product**	**Reaction Type**	(kJ/mol)	(kJ/mol)	(kJ/mol)
**ac⁴C**	**C**	Deacetylation	-6.2	-11.7	-8.0
**C**	**U**	Deamination	-39.2	-36.6	3.7

S4 Reference

[1] F. Neese, *WIREs Computational Molecular Science* **2022**, *12*, e1606.
[2] P. Pracht, F. Bohle, S. Grimme, *Physical Chemistry Chemical Physics* **2020**, *22*, 7169-7192.
[3] S. Grimme, *Journal of Chemical Theory and Computation* **2019**, *15*, 2847-2862.
[4] C. Bannwarth, S. Ehlert, S. Grimme, *Journal of Chemical Theory and Computation* **2019**, *15*, 1652-1671.
[5] S. Spicher, S. Grimme, *Angewandte Chemie International Edition* **2020**, *59*, 15665-15673.
[6] G. W. T. M. J. Frisch, H. B. Schlegel, G. E. Scuseria, M. A. Robb, J. R. Cheeseman, G. Scalmani, V. Barone, G. A. Petersson, H. Nakatsuji, X. Li, M. Caricato, A. Marenich, J. Bloino, B. G. Janesko, R. Gomperts, B. Mennucci, H. P. Hratchian, J. V. Ortiz, A. F. Izmaylov, J. L. Sonnenberg, D. Williams-Young, F. Ding, F. Lipparini, F. Egidi, J. Goings, B. Peng, A. Petrone, T. Henderson, D. Ranasinghe, V. G. Zakrzewski, J. Gao, N. Rega, G. Zheng, W. Liang, M. Hada, M. Ehara, K. Toyota, R. Fukuda, J. Hasegawa, M. Ishida, T. Nakajima, Y. Honda, O. Kitao, H. Nakai, T. Vreven, K. Throssell, J. A. Montgomery, Jr., J. E. Peralta, F. Ogliaro, M. Bearpark, J. J. Heyd, E. Brothers, K. N. Kudin, V. N. Staroverov, T. Keith, R. Kobayashi, J. Normand, K. Raghavachari, A. Rendell, J. C. Burant, S. S. Iyengar, J. Tomasi, M. Cossi, J. M. Millam, M. Klene, C. Adamo, R. Cammi, J. W. Ochterski, R. L. Martin, K. Morokuma, O. Farkas, J. B. Foresman, and D. J. Fox, Gaussian, Inc., Wallingford CT, **2016**.
[7] A. D. Becke, *The Journal of Chemical Physics* **1993**, *98*, 1372-1377.
[8] S. Grimme, A. Hansen, J. G. Brandenburg, C. Bannwarth, *Chemical Reviews* **2016**, *116*, 5105-5154.
[9] F. Weigend, R. Ahlrichs, *Physical Chemistry Chemical Physics* **2005**, *7*, 3297-3305.
[10] A. V. Marenich, C. J. Cramer, D. G. Truhlar, *The Journal of Physical Chemistry B* **2009**, *113*, 6378-6396.
[11] A. Altun, F. Neese, G. Bistoni, *Beilstein Journal of Organic Chemistry* **2018**, *14*, 919-929.
[12] M. Saitow, U. Becker, C. Riplinger, E. F. Valeev, F. Neese, *The Journal of Chemical Physics* **2017**, *146*, 164105.
[13] T. H. Dunning, Jr., *The Journal of Chemical Physics* **1989**, *90*, 1007-1023.
[14] R. A. Kendall, T. H. Dunning, Jr., R. J. Harrison, *The Journal of Chemical Physics* **1992**, *96*, 6796-6806.
[15] D. E. Woon, T. H. Dunning, Jr., *The Journal of Chemical Physics* **1993**, *98*, 1358-1371.
[16] F. Neese, E. F. Valeev, *Journal of Chemical Theory and Computation* **2011**, *7*, 33-43.
[17] N. K. Kochetkov, E. I. Budovskii, in *Organic Chemistry of Nucleic Acids: Part B* (Eds.: N. K. Kochetkov, E. I. Budovskii), Springer US, Boston, MA, **1972**, pp. 425-448.
[18] R. Shapiro, R. S. Klein, *Biochemistry* **1966**, *5*, 2358-2362.
[19] B. Kim, J. Hong, *Curr Top Med Chem* **2015**, *14*, 2759-2782.
[20] K. A. Gray, O. S. Pogrebinsky, G. T. Mrachko, L. Xi, D. J. Monticello, C. H. Squires, *Nature Biotechnology* **1996**, *14*, 1705-1709.
